# Development of a Novel Phenotypic Roadmap to Improve Blueberry Quality and Storability

**DOI:** 10.3389/fpls.2020.01140

**Published:** 2020-08-14

**Authors:** Brian Farneti, Francesco Emanuelli, Iuliia Khomenko, Matteo Ajelli, Franco Biasioli, Lara Giongo

**Affiliations:** ^1^ Genomics and Biology of Fruit Crop Department, Fondazione Edmund Mach, Trento, Italy; ^2^ Food Quality and Nutrition Department, Fondazione Edmund Mach, Trento, Italy

**Keywords:** *Vaccinium* spp., texture, aroma, Proton Transfer Reaction-Time of Flight-Mass Spectrometry, breeding, storage

## Abstract

Improved fruit quality and prolonged storage capability are key breeding traits for blueberry (*Vaccinium* spp.) fruit. Until now, breeding selection was mostly oriented on the amelioration of agronomic traits, such as flowering time, chilling requirement, or plant structure. Up until now, however, the storage effect on fruit quality has not been extensively studied, mostly because objective and handy phenotyping tools to evaluate quality traits were not available. In this study we are proposing a novel phenotyping protocol to support breeding selection and quality control within the entire blueberry production chain. Volatile organic compounds (VOCs) and texture traits, were measured by Proton Transfer Reaction- Time of Flight- Mass Spectrometry (PTR-ToF-MS) and a texture analyzer respectively, taking into consideration the influence of prolonged storage. The exploitation of the genetic variability existing within the investigated blueberry germplasm collection (including both southern and northern highbush, hybrids, and rabbiteyes) allowed the identification of the best performing cultivars, based on texture and VOCs variability, to be used as superior parental lines for future breeding programs. The comprehensive characterization of blueberry aroma allowed the identification of a wide array of spectrometric features, mostly related to aldehydes, alcohols, terpenoids, and esters, that can be used as putative biomarkers to rapidly evaluate the blueberry aroma variations related to genetic differences and storability. In addition, this study revealed a lack of straightforward relationship between harvest and postharvest quality features, that might be genotype-dependent.

## Introduction

Worldwide blueberry (*Vaccinium* spp.) production, for both processed and fresh market, has increased over the last decade making blueberry becoming the second most important soft fruit species after strawberry (Romo-Muñoz et al., 2019). Fresh market production, for instance, rose from about 270,000 tons to 370,000 tons in only 4 years (2012-2016, https://www.internationalblueberry.org/). Nonetheless, this progression mostly concerns certain production areas, situated for the most part in Peru, Mexico, Spain, Poland, South Africa, and China (Romo-Muñoz et al., 2019). The development of new blueberry accessions, suitable for cultivation under different climate conditions and for prolonged storage, is thus fundamental to guarantee a year-round supply of blueberries to cover the rising market demand, and with shipped fruit being in the best possible conditions upon delivery.

Initially, blueberry breeding programs were focused on developing cultivars to withstand conditions in the northern United States, including, among the major traits, disease resistance and broad ripening times (Hancock, 2009). Later, certain breeding programs became focused on the development of genotypes adapted to climatic conditions in the southern United States by hybridizing *V*. *corymbosum* with other species like *V*. *darrowii* and *V. elliottii* (Lyrene et al., 2003). Today, key breeding traits are a larger harvest window, improved fruit quality, and better storage capability (Gallardo et al., 2018).

Although trait relevance highly depends on supply chain, addressing high and distinguishable fruit quality is essential for capturing consumer preferences. Recent studies (Gilbert et al., 2015; Gallardo et al., 2018) disclosed that flavor, texture, and prolonged shelf-life are the most appreciated quality traits for both blueberry industry and consumers. Notably, consumers identify typical blueberry flavor and sweetness as positive quality traits, and unpleasant texture attributes, such as mealy and pasty, as negative ones. Both textural and flavor attributes decline during storage but a generic aim of shelf-life extension may have unintended negative consequences on other fruit quality traits, for instance aroma, as already suggested for several horticultural products, like strawberry, peach, apple, or tomato (Goff and Klee, 2006; Klee, 2010; Rambla et al., 2014; Farneti et al., 2017a; Tieman et al., 2017). The chance of this quality decline may be heightened by the fact that breeding selection for aroma has occurred almost without analytical assistance, since aroma is still not considered as a discriminating trait in the early breeding selection phase (Klee and Tieman, 2018). This is also strengthened by the complex and time-consuming phenotyping protocols ordinarily used, which make the analytical screening of wide germplasm unfeasible.

Blueberry aroma depends on the interaction of dozens of volatile compounds (VOCs) synthesized by fruit during ripening (Du et al., 2011; Beaulieu et al., 2014; Du and Rouseff, 2014; Gilbert et al., 2015; Farneti et al., 2017b). As most secondary plant metabolites, VOCs are detectable in blueberry fruit with high variability according to genetic and environmental differences and, above all, to the biological ripening stage of the fruit at the time of analysis (Farneti et al., 2017b). Most of compounds responsible for blueberry aroma are synthesized by the fruit in the full ripe stage, such as linalool and majority of monoterpenes, (Z)-2-hexen-1-ol, and hexanal, or at the pink stage of ripening, such as (E)-2-hexenal (Farneti et al., 2017b). Esters, although being present in lower average concentration, strongly affect blueberry aroma, especially “sweet” and “fruity” fragrances. A large fraction of esters, such as ethyl acetate, methyl isovalerate, ethyl isovalerate, methyl 2-methylbutanoate, are exclusively synthesized in the last phase of ripening and magnified in overripe fruit (Farneti et al., 2017b).

In order to satisfy consumer demands more effort and attention need to be devoted, from a scientific and practical background, to improve and optimize blueberry quality upon delivery to the consumers. The key attributes of quality may vary with context and depend on the intended use of the product and the available or affordable technology (Abbott, 1999). Defining and quantifying quality properties of blueberry fruit, in relation to distinct segments of the production chain, needs comprehensive investigations.

While taste traits (sweetness and sourness) are relatively well explained by the sugar content and titratable acidity, the prediction of aroma and texture traits seems more uncertain (Blaker et al., 2014; Gilbert et al., 2015; Ferrão et al., 2020) because of the lack of precision in the instrumental measures and the high interactions among traits (Folta and Klee, 2016). Breeders need selection criteria, both efficient and easy to assess, for supporting organoleptic quality breeding. Physical and chemical traits could be an alternative approach for routine measurements of some of the quality traits, but molecular markers will provide more efficient tool for selecting improved genotypes (Folta and Klee, 2016). The genetic dissection of these complex processes would permit a more systematic approach to plant improvement than has been possible previously (Klee and Tieman, 2018). An important component that has to be studied more accurately, also from a genetic perspective, is the fruit quality deterioration during storage and the resistance of fruit to several postharvest biotic and abiotic disorders. The achievement of this goal will only be possible with a more accurate and objective quality traits phenotyping, ideally combined with multivariate prediction models of quality perception.

In this study, a wide blueberry germplasm collection, including southern and northern highbush, hybrids and rabbiteyes, was employed and assessed for both texture and aroma traits applying advanced phenotyping strategies preliminarily developed in Giongo et al. (2013) and Farneti et al. (2017b). The aims of this work were i) to estimate the potential genetic variability among blueberry cultivars for both quality traits and ii) to evaluate how post-harvest cold storage may influence this quality variability. Knowing of the genetic variability existing within the blueberry germplasm could allow a precise identification of the best performing cultivars to be used as superior parental lines for future breeding program aimed to improve blueberry fruit quality. In addition, results of this study might be useful in defining an objective phenotyping protocol to apply in the selection breeding phases of blueberry.

## Materials and Methods

### Plant Material and Sampling

Forty-six *Vaccinium* accessions (**Table 1**) were chosen from the experimental field of Edmund Mach Foundation Research and Innovation Centre at Pergine (Trento), located in the northern Italy (Trentino Alto Adige region). At the time of the analysis, plants were in the full production phase, between 7 and 10 years old. Bushes were maintained following standard pruning and surface bark mulching renewal. In the plot, each of the accessions was represented by at least five plants. To avoid both misnaming and redundant genotypes each accessions employed in the study was checked with molecular markers.

**Table 1:** List of the *Vaccinium* spp. cultivars employed in this study.

	**CULTIVAR**			**CULTIVAR**	
**1**	Aron	*V. angustifolium, V. uliginosum*	**24**	Jubilee	*V. corymbosum, V. darrowii, V. elliotti*
**2**	Atlantic	*V. corymbosum*	**25**	Legacy	*V. corymbosum, V. darrowii*
**3**	Aurora	*V. corymbosum*	**26**	Liberty	*V. corymbosum*
**4**	Azur	*V. corymbosum*	**27**	Marimba	*V. corymbosum, V. darrowii, V. ashei*
**5**	Berkeley	*V. corymbosum*	**28**	Misty	*V. corymbosum, V. darrowii*
**6**	Biloxi	*V. corymbosum, V. virgatum, V. darrowii*	**29**	Mondo	*-*
**7**	Blue Crop	*V. corymbosum*	**30**	North Blue	*V. corymbosum, V. angustifolium*
**8**	Blue Moon	*V. corymbosum*	**31**	Northland	*V. corymbosum, V. angustifolium*
**9**	Brigitta Blue	*V. corymbosum*	**32**	Nui	*V. corymbosum*
**10**	Centra Blue	*V. virgatum*	**33**	O’Neal	*V. corymbosum, V. darrowii, V. ashei*
**11**	Centurion	*V. virgatum*	**34**	Ozark Blue	*V. corymbosum*
**12**	Chandler	*V. corymbosum*	**35**	Poppins	*V. corymbosum, V. ashei*
**13**	Compact	*V. corymbosum*	**36**	Primadonna	*V. corymbosum hybrid*
**14**	Cosmopolitan	*-*	**37**	Puru	*V. corymbosum*
**15**	Coville	*V. corymbosum*	**38**	Roxy Blue	*V. corymbosum, V. darrowii, V. elliotti*
**16**	Darrow	*V. corymbosum*	**39**	Rubel	*V. corymbosum*
**17**	Early Blue	*V. corymbosum*	**40**	Safir	*V. corymbosum*
**18**	Elizabeth	*V. corymbosum*	**41**	Simultan	*V. corymbosum*
**19**	Elliott	*V. corymbosum*	**42**	Sky Blue	*V. virgatum*
**20**	Emerald	*V. corymbosum, V. darrowii, V. elliotti*	**43**	Southern Belle	*V. corymbosum, V. darrowii*
**21**	Goldtraube	*V. corymbosum, V. lamarkii*	**44**	Star	*V. corymbosum, V. darrowii, V. ashei*
**22**	Jersey	*V. corymbosum*	**45**	Top Hat	*V. angustifolium*
**23**	Jewel	*V. corymbosum, V. darrowii, V. elliotti*	**46**	Toro	*V. corymbosum*

Fruit were harvested at maturity stage assessed according to the method described by Giongo et al. (2013), coincident with the commercial harvest. Homogeneous fruit, free from external damages or irregularities, were sampled immediately at harvest and divided into two batches, of about 80 fruit each. Analyses were carried out at harvest and after 6 weeks of storage, at 2°C (RH 85%). Each fruit batch was subsequently re-divided into two subsets for texture and VOC analysis, respectively.

### DNA Extraction

Plant material of 46 blueberry accessions (**Table 1**) was collected from young leaves, stored at -20°C and then vacuum lyophilized (72 h) prior to DNA extraction. DNA was extracted in triplicate using the DNeasy 96 Plant Kit (Qiagen GmbH, Germany) according to the manufacturer’s protocol. DNA was re-suspended in 200 ul AE buffer (Qiagen GmbH, Germany) and was diluted 1:10 for use in PCR. The quality and concentration of all DNA samples was visually checked on agarose gels and estimated using a NanoDrop ND-8000 spectrophotometer. Six SSRs (**Table S1**) were chosen as proposed by Boches et al. (2006) and were grouped into two separate multiplexed reactions, MVA and MVB, and used to screen all genotypes. Each specific forward primer was connected with one of the 5′ universal primer sequence tails (Missiaggia and Grattapaglia, 2006; Ge et al., 2014) which were T7 (5′-TAATACGACTCACTATAGGG), M13 (5′-TGTAAAACGACGGCCAGT), M13R (5′-CAGGAAACAGCTATGACC), or D12S1090f (5′-CTATAGGGCACGCGTGGT) (**Table S2**).

The stand alone universal primers M13, D12S1090f, M13R and T7 were fluorescently labeled with FAM, VIC, NED, and PET respectively. All primers were ordered from Life Technologies and dissolved in TE (10 mM Tris, 1 mM EDTA, pH 8.0) to produce a 20 μM stock solution for fluorescently-labeled universal primer and locus-specific reverse primer and a 10μM stock solution of locus-specific forward primer with universal tail (moles of tailed forward primer: reverse primer: dye-labeled universal primer= 1:2:2). Primer mix here designated Pmixlocus (three primers were mixed for each specific locus). Each Multiplex included equimolar amounts of the Pmixlocus.

Multiplexed reactions (MVA and MVB) were carried out in 10ul reactions using Type-it™ Microsatellite PCR kit (Qiagen) according to the manufacturer instructions. Thermal cycling conditions for MVA were as follow: initial 3 min denaturation step at 94°C, eight touch-down cycles comprising a 30 s denaturation at 94°C followed by 90 s of annealing starting at 64°C and decreasing, 0.5°C per cycle, down to 60°C and 60 s of extension at 72°C. Subsequently, 25 more identical cycles were conducted with and annealing temperature of 60°C followed by a final 30 min extension step at 60°C. Thermal conditions for MVB changed only for the annealing temperature, starting at 60°C and decreasing 0.5°C per cycle, down to 60°C. Subsequently, 25 more identical cycles were conducted with an annealing temperature of 56°C followed by a final 30 min extension step at 60°C. Products from multiplexed reactions were checked on agarose gels and then diluted 1:50 prior to analysis and separated by electrophoresis on an ABI3730XL genetic analyzer (Applied Biosystems).

Data generated were then acquired and analysed using the GENEMAPPER (Applied Biosystems) software and checked visually. Final allele sizes were thus obtained per locus and per blueberry accession after removing the length of each universal tail sequence.

### Genetic Structure Analysis

To investigate the genetic relationships between blueberry genotypes, the microsatellite dataset was analyzed using the Poppr package (Kamvar et al., 2014) in R (3.1.3 version, https://www.r-project.org).

Initially, the molecular profile for the 6-SSRs was examined by using the discriminant analysis of principal components (DAPC) implemented in the Adegenet package ver. 2.0.1 (Jombart, 2008; Jombart et al., 2010). Prior clusters were identified by a sequential K-means clustering algorithm (“find.clusters” function) after data transformation by Principal Component Analysis (PCA). Then, a discriminant analysis (DA) used part of the principal components (PCs) to describe the clusters. K-means was run with K varying from 1 to 20 and, to ensure convergence, we increased the number of starting points to 200. The number of clusters was chosen based on the Bayesian Information Criterion (BIC) (Schwarz, 1978). To avoid retaining too many dimensions at the DA step, the optimal number of PCs was determined using both “optim.a.score” and “xvalDapc” functions from Adegenet. The final cluster assignment was obtained after the DA step (posterior assignment of the DAPC analysis).

Once each genotype was assigned to a specific cluster, a dendrogram was established using Bruvo’s distance (Bruvo et al., 2004) and Neighbour Joining (NJ) clustering (Paradis et al., 2004). Bruvo’s distance takes into account the mutational process of microsatellite loci and is well adapted to populations with mixed ploidy levels and is therefore suitable for the study of the blueberry collection used in this work that included both autotetraploid (*V.* *corymbosum*) and hexaploid cultivars (*V.* *virgatum*, “Rabbiteye”). The ‘‘bruvo.boot’’ command (Kamvar et al., 2014) with bootstrap support of 1000 replication was used to produce a neighbor joining tree with the ‘‘njs’’ algorithm from the ape package ver. 5.0 (Paradis et al., 2004).

### Texture Analysis

Texture assessment was performed for each cultivar on 20 homogenous fruit at harvest and after storage. Texture was determined by a texture analyser (Zwick Roell, Ulm, Germany), which profiled a mechanical force displacement using a 5 kg loading cell and a cylindrical flat head probe with a diameter of 4 mm entering into the berry flesh from the sagittal side (for more details see Giongo et al., 2013). The mechanical profile was defined by two fundamental variables: force (N) and distance (strain, %). The force was measured with the following instrumental settings: test speed of 100 mm min^-1^, post-test speed of 300 mm min^-1^, auto force trigger of 2 g, and stop plot at target position. Each berry was compressed until deformation of 90%. Data of the mechanical profiles were acquired with a resolution of 500 points per second. On the force displacement profile, seven parameters were computed: maximum force, final force, area, maximum deformation, minimum deformation, maximum force strain, and gradient (or imitative Young’s module, also known as elasticity module). All data were analyzed by TaxtExpertII software (Zwick Roell, Ulm, Germany).

### VOC Analysis by PTR-ToF–MS

Three biological replicates of 1 g of powdered frozen sample, each obtained by five fruit, were inserted into 20 ml glass vials equipped with PTFE/silicone septa (Agilent, Cernusco sul Naviglio, Italy) and mixed with 1 ml of deionized water, 400 mg of sodium chloride, 5 mg of ascorbic acid, and 5 mg of citric acid (Farneti et al., 2017b). Measurements of blueberry VOCs were performed in three biological replicates with a commercial PTR-ToF–MS 8000 apparatus (Ionicon Analytik GmbH, Innsbruck, Austria; Farneti et al., 2017b). The drift tube conditions were as follows: 110°C drift tube temperature, 2.25 mbar drift pressure, 550 V drift voltage. This leads to an E/N ratio of about 140 Townsend (Td), with E corresponding to the electric field strength and N to the gas number density (1 Td = 10‑17 Vcm2). The sampling time per channel of ToF acquisition was 0.1 ns, amounting to 350,000 channels for a mass spectrum ranging up to *m/z* = 400. The sample headspace was withdrawn through PTR-MS inlet with 40 sccm flow for 60 cycles resulting in an analysis time of 60 s/sample. Pure nitrogen was flushed continuously through the vial to prevent pressure drop. Each measurement was conducted automatically after 20 min of sample incubation at 40°C and 2 min between each measurement was applied in order to prevent memory effect. All steps of measurements were automated by an adapted GC autosampler (MPS Multipurpose Sampler, GERSTEL) coupled to PTR-ToF-MS.

The analysis of PTR-ToF–MS spectra proceeded as described in Farneti et al., 2017b.

### Data and Statistical Analysis

The array of masses detected with PTR-ToF-MS was reduced by applying noise and correlation coefficient thresholds. The first removed peaks were not significantly different from blank samples; the latter excluded peaks with over 99% correlation, which correspond for the most part to isotopes of monoisotopic masses (Farneti et al., 2017b).

For all quality parameters, both texture and volatiles, a storage index (SI) was computed using the formula proposed by Giongo et al., 2013, SI = log_2_(Qi_PH_/Qi_H_), where Qi_H_ is the value of the i-th quality parameter measured at harvest, and Qi_PH_ is the value of the same parameter measured after cold storage. Positive SI values indicate a quality trait enhancement, while negative values highlight a loss of the quality trait during storage.

R.3.4.1. R Foundation for Statistical Computing, Vienna, Austria) internal statistical functions and the external packages “mixOmics”, “FactoMineR”, and “ggplot2” were used for the multivariate statistical methods (PCA, MFA, hierarchical clustering, and definition of significant cluster numbers) employed in this work.

## Results

### Genetic Variability of *Vaccinium* Germplasm

**Figure 1:**
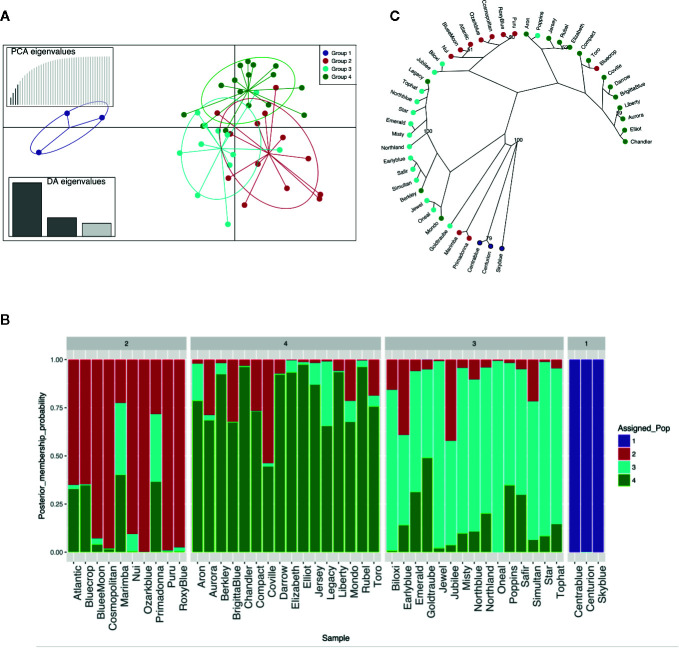
Genetic structure of the 44 blueberry samples evaluated with six SSR. **(A)** Discriminant analysis of principal components (DAPC). **(B)** STRUCTURE-like plot of DAPC analysis for a global picture of the clusters composition. Each individual is represented by a vertical coloured line. Same colour in different individuals indicates that they belong to the same cluster. **(C)** Radial dendrogram based on Bruvo’s distance.

MVA and MVB multiplexed reactions failed for two blueberry accessions [‘Azur’ (#4) and ‘Southern Belle’ (#43)] out of 46 samples and thus these accessions were not included in the genetic analysis. The obtained 44 molecular profiles were further used to identify K-clusters by successive K-means, resulting in the definition of four clusters (**Figure1A**, **Table S3**). Then, SSR profiles were used for the construction of a dendrogram reflecting the genetic proximity between genotypes. The method was based on Bruvo’s distance and NJ and was chosen for being reliable and suitable for populations with mixed ploidy levels. Again, the germplasm collection clustered into four main genetic groups with a good level of accordance to the DAPC grouping (**Figure 1C**). As expected, the hexaploid accessions (“Rabbiteye”) were clustered distinctively (Group 1) and displayed a large genetic distance to the tetraploid cultivars as previously reported by Bian et al. (2014). Tetraploid accessions were clustered into three groups. Those that clustered into three distinct groups by DAPC also displayed a closer genetic proximity in the NJ dendrogram. This result reflects the different and complex pedigree of southern highbush blueberry [i.e. ‘Biloxi’ (#6), ‘Jubilee’ (#24) and ‘Misty’ (#28)], half-high blueberry [i.e. ‘Top Hat’ (#45), ‘Northland’ (#31)] clustering mainly in group 3 and northern highbush blueberry (group 2 and group 4). However, there were some cases of disagreement [‘Legacy’ (#25), ‘Bluecrop’ (#7), ‘Poppins’ (#35), ‘Berkley’ (#5), ‘Mondo’ (#29), ‘Primadonna’ (#36), and ‘Marimba’ (#27)]. These accessions, despite being assigned to a specific group in the DAPC analysis, showed a certain degree of admixture in the assignment plot (**Figure 1B**). In particular, admixture appeared to be the strongest in ‘Marimba’ (#27) and ‘Primadonna’ (#36) which grouped distinctively in the NJ dendrogram.

### Advanced Texture Phenotyping of *Vaccinium* Germplasm

**Figure 2:**
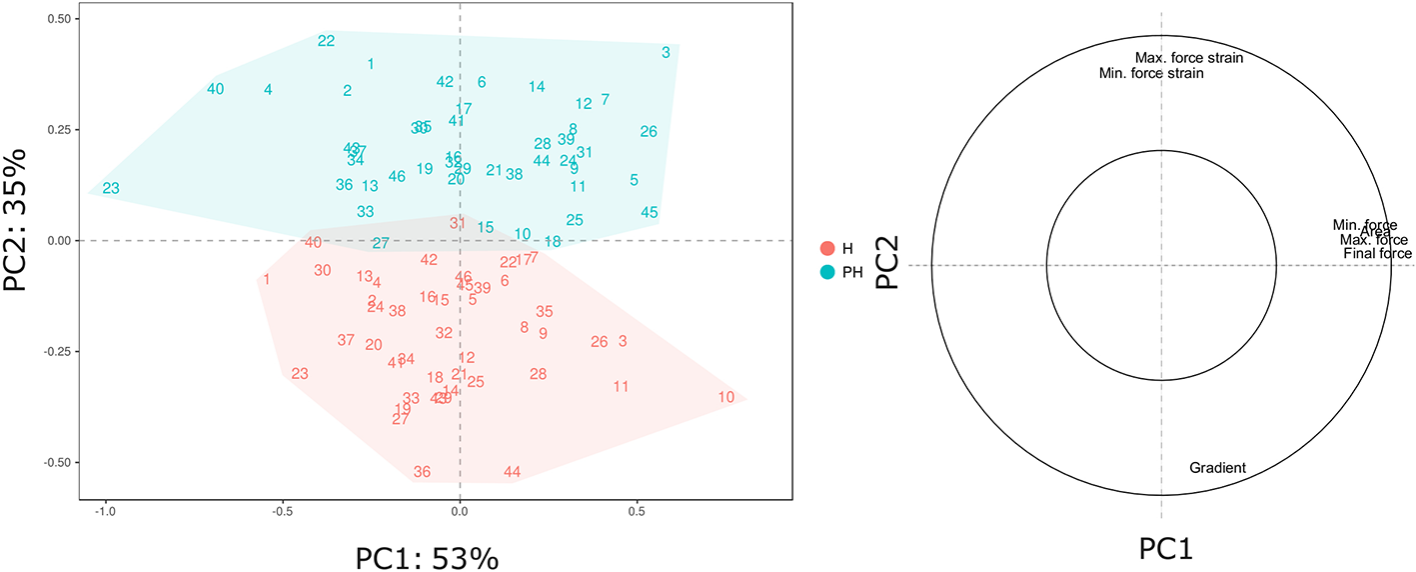
PCA and loading plot of the texture profile of 46 *Vaccinium* spp. cultivars, assessed by texture analyzer at harvest and after storage. Each point of the PCA plot is the average of 20 measurements.

The broad genetic variability revealed in our *Vaccinium* germplasm collection may lead to a high phenotypic variance that can be enhanced and altered by prolonged cold storage. Phenotypic variance for texture qualities and for their alteration during cold storage (**Table 2**), is represented by a PCA plot (**Figure 2**) defined by the first two PCs (PC1: 53% and PC2: 35%). Textural differences related to cold storage are mostly explainable by the second component (PC2) variability for mostly correlated with force strain and gradient (Young’s module). Indeed, storage index values of maximum force strain and gradient were, on average, +0.31 and -0.95, respectively (**Table 2**). Differences among *Vaccinium* genotypes are predominantly related to the first component (PC1) that is highly correlated to the deformation forces, both maximum and minimum. In accordance with the results presented in Giongo et al. (2013) the gradient is orthogonally oriented to the force related parameters and almost oppositely oriented to deformation strains. The perception of a gummy berry is associated with an increased deformation strain at the maximum force caused by a lower turgidity and a high resistance against the force required to break the skin (Paniagua et al., 2013; Blaker et al., 2014). Based on these textural parameters blueberry fruit can be categorized into three main groups. The first group, mostly distinguished based on high gradient values, is characterized by turgid fruit with a high internal turgor pressure while the second group is mostly composed of firm, rather than turgid, fruit. The last group is instead defined by low texture performance berries, for both the deformation modulus and deformation forces, leading to the perception of gumminess.

**Table 2:** Texture parameters detected by a texture analyzer at harvest and after storage, over 46 *Vaccinium* spp. cultivars.

		**HARVEST**			**POST HARVEST**			**STORAGE INDEX**		
		*min*	*max*	*average*	*min*	*max*	*average*	*min*	*max*	*average*
**Gradient**	*MPa*	0.99	2.22	1.46	0.41	1.33	0.77	-1.59	0.01	-0.95
**Maximum force strain**	*%*	2.60	5.08	3.98	3.95	6.41	4.92	0.07	0.70	0.31
**Minimum force strain**	*%*	4.55	6.66	5.83	5.37	9.29	6.89	0.01	0.55	0.24
**Max Force**	*N*	2.58	5.13	3.58	1.94	5.41	3.80	-0.41	0.51	0.07
**Min Force**	*N*	0.50	1.38	0.81	0.44	1.68	0.96	-0.56	0.82	0.21
**Area**	*N%*	114.08	256.81	157.08	82.29	243.76	170.34	-0.53	0.52	0.10
**Final Force**	*N*	0.85	2.39	1.43	0.64	2.31	1.60	-0.43	0.69	0.14

For each parameter the average (20 replicates for each cultivar), minimum and maximum values are reported. In addition, the change fold values between harvest and storage assessments (expressed as SI index) are reported.

**Figure 3:**
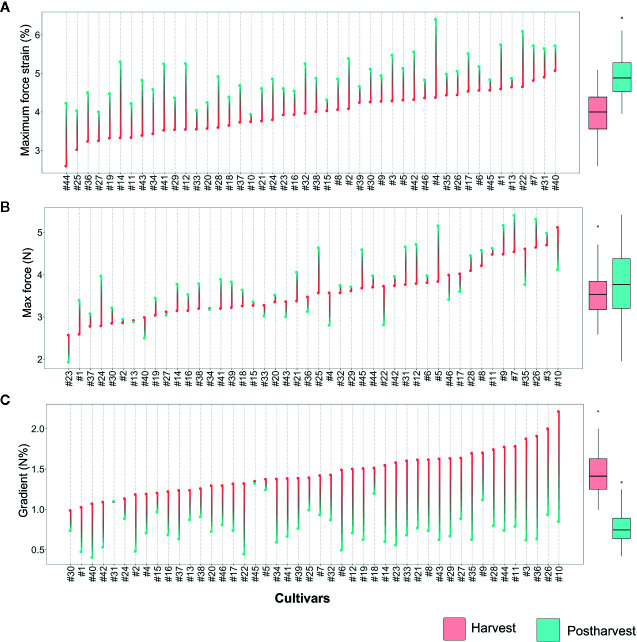
Lollipop graph and box plot of three texture parameters: **(A)** Maximum force strain, **(B)** Maximum force, **(C)** Gradient. Each graph illustrates the average value (of 20 measurements) recorded at harvest and after 6 weeks of storage, for each cultivar (names of the cultivars are reported in **Table 1**). For graphical purpose, cultivars of each graph are ordered based on the trait level recorded at harvest. The box plot, reported next to each lollipop graph, summarise the differences between fruit assessed at harvest and after storage. Lollipop graphs, together with distribution plots and box plots of all 7 texture parameters, are reported in **Figure S1**.

The complexity of the blueberry texture analysis can be reduced to only three variables: deformation strain at maximum force, maximum force, and gradient. All three textural parameters, assessed at harvest and after storage, disclosed a high variability between *Vaccinium* cultivars (**Figure 3**, **Figure S1**). Nevertheless, a strict correlation between texture values assessed at harvest and after storage is not found. This indicated a strong cultivar-storage interaction that cannot be fully estimated considering only the assessment at harvest.

Deformation at maximum force (**Figure 3A**, **Figure S1**) ranged from around 2.6 [‘Star’ (#44)] to 5% [‘Safir’ (#40)] at harvest. After 6 weeks of storage, the deformation of all cultivars increased, without any significant relation with the values recorded at harvest. Several accessions, such as ‘Star’ (#44), ‘Elliott’ (#19), or ‘Chandler’ (#12), were defined by low deformation levels at harvest and remarkably high after storage. Differently, other cultivars characterised by low deformation at harvest, like ‘Centra Blue’ (#10), did not considerably change during storage. Accessions defined by high deformation at harvest also showed the same variability, with cultivars stable during storage, like ‘Biloxi’ (#6) and ‘Compact’ (#13), or very unstable ones like ‘Jersey’ (#22) and ‘Azur’ (#4). The overall increase in deformation caused by prolonged storage can be mostly explained by a turgidity decrement of blueberries that lost between 6 to 15% of water during storage. However, no significant correlation was found between fruit weight loss and deformation fold changes (R^2 =^ 0.12, **Figure S2**).

Maximum force variability of fruit assessed at harvest (**Figure 3B**) ranged from around 2.7 [‘Jewell’ (#23)] to 5.1 N [‘Centra Blue’ (#10)]. Differently from the deformation results, the maximum force variability, assessed after storage, increased. This high variability is mostly explainable by both positive and negative variations during storage. Among the accessions that showed low force values at harvest, “Jewell” (#23) decreased the force after storage, while ‘Jubilee’ (#24) more than doubled its value. Among those accessions defined by high force level at harvest, the force level decreased for ‘Centra Blue’ (#10) fruit, while increased for ‘Brigitta Blue’ (#9). In addition, several accessions, like ‘Biloxi’ (#6) and ‘Centurion’ (#11), showed a very stable force level during storage with minimal modifications.

The gradient values were highly negative correlated with deformation (R^2^: 0.47; **Figure S2**) as it was previously evidenced by the PCA analysis (**Figure 2**). Therefore, changes among blueberry cultivars, at harvest and during storage, were comparable with the deformation ones (**Figure 3C**). Gradient module ranged from around 1.0 [‘Northblue’ (#30)] to 2.2 N% [‘Centra Blue’ (#10)] at harvest. After storage, the gradient module of all cultivars decreased, without any significant relation with the values recorded at harvest. Although several cultivars revealed similar trends for both Young’s module and deformation during storage, such as ‘Coville’ (#15), ‘Top Hat’ (#45), or ‘Star’ (#44), for other cultivars, as ‘Centra Blue’ (#10), ‘Biloxi’ (#6), or ‘O’Neal’ (#33), these values were not comparable. For instance, ‘Centra Blue’ (#10) was the cultivar with the strongest gradient decrement during storage and, at the same time, one with the lowest deformation change. That resulted in a weak correlation (R^2 =^ 0.1) between the storage index values of the deformation strain and deformation (**Figure S2**).

### Cultivar Characterization Based on Storage Textural Modification

**Figure 4:**
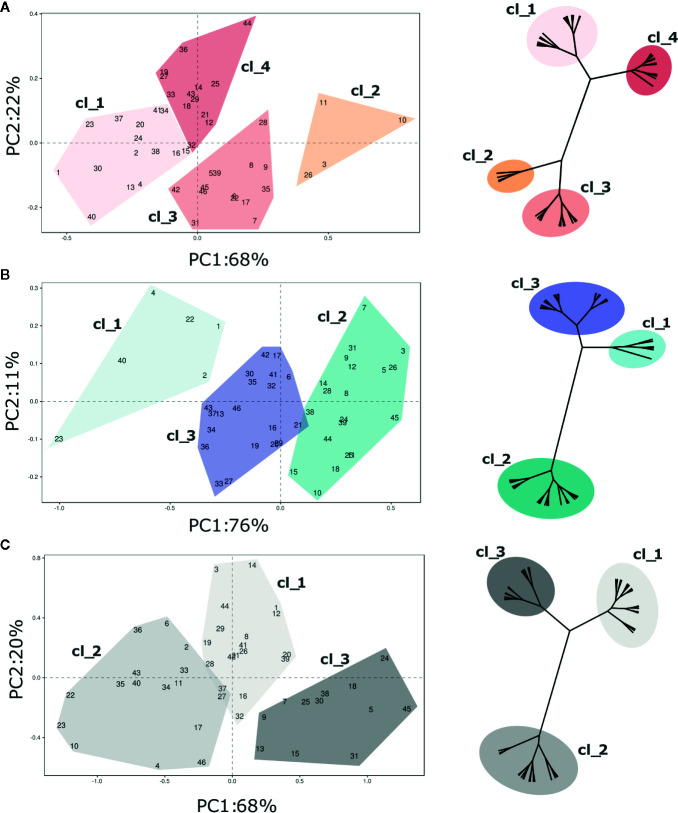
PCA and hierarchical clustering (based on Ward distances) of 46 *Vaccinium* spp cultivars based on texture profile assessed at **(A)** harvest and **(B)** postharvest, and on the calculated storage index **(C)**. Each point of the PCA plot is the average of 20 fruit. Loading plots of each PCA are reported in **Figure S3**. Clusters highlighted in each PCA plot corresponds to the clusters identified by Ward clustering.

Texture profiling of the blueberry germplasm collection was further analysed at harvest and after storage separately, in order to characterize each cultivar more accurately. In addition, cultivar stability during storage was estimated based on the Storage Index (SI) computed on each texture parameter. As for the PCA based on the whole texture database (**Figure 2**), the total variance of each PCA—based on the texture profiles at harvest, storage, and SI- was mostly entirely explained by the first two components (**Figure 4**, **Figure S3**, **Table S3**).

Four distinct clusters were statistically distinguished at harvest based exclusively on texture (**Figure 4A**). Most of variability (68%) is explainable by the first PC, that is highly correlated with the maximum and minimum forces, the total area, and the gradient (**Figure S3**). The deformation at maximum and minimum forces is rather related with the second component, accounting for 22% of the explained variability. Accessions belonging to the first two clusters (“Cl_1”, and “Cl_2”) were mostly distinguished based on forces and gradient. Cultivars of the first cluster, such as ‘Jewell’ (#23), ‘Emerald’ (#20), or ‘Ozark Blue’ (#34), were defined by low forces and gradient while the four accessions of the second cluster [‘Centurion’ (#11), ‘Centra Blue’ (#10), ‘Aurora’ (#2), and ‘Liberty’ (#26)] by high deformation forces and gradient values. The last two clusters (“Cl_3”, “Cl_4”), both characterized by intermediate values of force and gradient, were mostly separated based on deformation. Cultivars of the third cluster, such as ‘Bluecrop’ (#7), ‘Brigitta Blue’ (#9), or ‘Biloxi’ (#6), were defined by high deformation values, while cultivars of the fourth cluster, such as ‘Star’ (#44), ‘O’Neal’ (#33), and ‘Legacy’ (#25), by low deformation.

Three clusters of cultivars were statistically distinguished after storage based on texture assessment (**Figure 4B**). These clusters were distributed according to the first component variation, that is highly correlated with the force displacement and gradient and it explained the 76% of the texture profiling variability. The second PC, mostly related with fruit deformation, explains only 11% of the variance. Based on this classification, the first cluster (“Cl_1”) included few blueberry accessions, such as ‘Jewell’ (#23), ‘Safir’ (#40), or ‘Jersey’ (#22), defined by extremely low force displacement and gradient values. Cultivars of the opposite cluster (“Cl_2”), such as ‘Brigitta Blue’ (#9), ‘Bluecrop’ (#7) and ‘Liberty’ (#26), were defined by positive values of the first component, as a result of higher values of deformation forces and gradient.

The PCA related to the Storage Index (**Figure 4C**) is intended to describe the potential storability of each accession based on textural characteristics. The SI provides valuable information related to the magnitude of the variation of each texture parameter during storage, rather than an absolute value. The 88% of this variability was explained by the first two components (PC1 68%, PC2 20%). Three distinct clusters of cultivars were statistically distinguished mostly based on the PC1 variability. The first cluster (“Cl_1”) was characterized by cultivars, such as ‘Aurora’ (#3), ‘Chandler’ (#12), or ‘Star’ (#44), with high fold changes of deformation at the maximum force, low delta of the gradient, and an intermediate delta of the forces. Accessions of the second cluster (“Cl_2”), such as ‘Biloxi’ (#6), ‘Centra Blue’ (#10), or ‘Jersey’ (#22), showed low fold change values of maximum force, and intermediate ones of the deformation at maximum force and of the gradient. Cultivars of the last cluster (“Cl_3”), such as ‘Jubilee’ (#24), ‘Northland’ (#31), or ‘Top Hat’ (#45), showed high changes of gradient values and forces, and low changes of the deformation at maximum force.

The texture profiling assessed at both harvest and after postharvest storage did not reveal any significant association with the genetic molecular profile based on six SSRs (**Figure S1**; **Figure S3**).

### Phenotyping of *Vaccinium* Germplasm Volatilome

Blueberry VOC profile was assessed at harvest and after storage in triplicate by PTR-ToF-MS analysis as described in Farneti et al. (2017b). Mass peaks from the raw PTR-ToF-MS spectra were reduced from 285 to 134, applying noise and correlation coefficient thresholds. Tentative identification of each mass, detected by PTR-ToF-MS, was based on comparison with pure standards and gas chromatographic results previously presented in Farneti et al., 2017b (**Table 3**).

**Table 3:** Volatile organic compounds detected by PTR-ToF-MS at harvest and after storage, over 46 *Vaccinium* spp. cultivars.

m/z	Formula	tentative Identification	HARVEST			POSTHARVEST			STORAGE INDEX		
			*min*	*Max*	*average*	*Min*	*max*	*average*	*min*	*max*	*average*
27.0263	C2H3+		0.07	1.49	0.22	0.12	2.81	0.58	-1.14	3.4	1.21
28.0184			0.09	0.24	0.16	0.07	0.24	0.15	-1.5	0.72	-0.16
28.0314			0.03	0.13	0.07	0.04	0.95	0.19	-1.36	3.44	1.03
30.0435			0.01	0.19	0.05	0.06	12.08	1.76	0.34	7.76	4.06
30.9952			0.81	1.14	0.93	0.97	1.14	1.05	-0.18	0.46	0.18
31.0455			0	0.35	0.05	0.01	0.17	0.06	-2.8	4.47	0.46
33.0331	CH4OH+	Methanol	502.07	5790.87	2228.26	647.91	11096.53	3956.04	-2.13	3.76	0.77
34.9958	H2SH+	Hydrogen sulfide	0.07	4.79	1.17	0.01	0.35	0.15	-5.49	1.53	-1.82
39.0228	C3H3+	common fragment	2.29	17.68	5.3	3.34	14.16	6.21	-1.31	1.74	0.27
41.0386	C3H5+	common fragment	6.83	51.65	12.26	7.38	46.6	16.97	-1.01	2.01	0.4
42.0101	C2H2O+		0.01	0.14	0.05	0.02	0.83	0.16	-0.81	4.04	1.41
42.0225			0.07	0.82	0.23	0.09	1.39	0.39	-1.41	3.41	0.74
43.0153	C2H3O+	common fragment	9	45.61	21.83	11.32	1388.15	68.48	-1.16	5.24	0.69
43.0576	C3H7+	common fragment	2.6	53.65	6.06	3.58	48.84	9.95	-1.07	3.14	0.71
44.058			0.08	1.71	0.2	0.1	1.53	0.34	-1.13	2.97	0.75
45.0319	C2H4OH+	Acetaldehyde	17.71	515	80.78	158.07	1448.6	651.28	0.09	5.02	3.21
47.0102	CH3O2+	Formic acid	2.93	6.56	4.38	2.29	6.11	3.98	-1.17	0.54	-0.16
47.0193			4.63	5.63	5.08	1.9	5.95	4.49	-1.41	0.36	-0.22
47.0436	C2H6OH+	Ethanol	0.48	21.56	2.44	9.92	2534.48	341.28	2.34	11.32	6.68
49.0112	CH4SH+	Methanethiol	0.03	6.61	0.59	0.02	0.47	0.09	-4.79	1.61	-1.59
49.0277			0.02	0.13	0.06	0.02	0.45	0.12	-1.16	3.44	0.84
49.9991			0.31	0.54	0.42	0.41	0.55	0.48	-0.27	0.66	0.2
51.0059			0.05	0.35	0.1	0.05	0.24	0.11	-1.41	1.74	0.15
51.0431	CH3OH*H3O+	Methanol cluster	10.53	120.38	45.71	13.65	258.95	81.27	-2.12	3.76	0.77
53.0039			0.03	0.3	0.06	0.02	0.12	0.06	-1.75	1.15	-0.07
53.0396	C4H5+	common fragment	0.71	4.81	1.91	0.93	3.49	1.77	-1.49	1.59	-0.02
55.0171	C3H3O+		0.09	0.56	0.32	0.1	1.77	0.33	-1.54	1.83	-0.06
55.0542	C4H7+	common fragment	13.66	110.63	43.32	16.34	75.46	38.92	-1.89	1.54	-0.06
57.0334	C3H4OH+	common fragment	63.22	414.55	205.7	10.81	311.75	149.17	-3.61	1.19	-0.57
57.0697	C4H9+	1-Octanol, high alcohol fragment	2.61	6.59	3.43	2.75	45.53	9.01	-0.45	3.57	1.08
60.0214			0	0.04	0.02	0.01	0.43	0.04	-2.28	8.52	0.9
61.0233	C2H4O2H+	Acetic acid, common ester fragment	4.56	46.19	14.04	6.08	2146.05	87.91	-1.88	7.19	1.24
63.0083			0.17	0.73	0.42	0.28	0.99	0.51	-0.69	2.06	0.39
63.0329	C2H6SH+	Dimethyl sulfide, Ethanethiol	0.75	2.63	1.62	1.27	14.32	2.88	-0.53	3.31	0.79
63.0425	C2H4O*H3O+	Ethanol cluster	0	1.3	0.07	0.09	4.92	1.6	-0.6	15.21	10.79
65.02			0.14	0.31	0.21	0.12	0.34	0.2	-1.01	0.91	-0.11
67.056	C5H7+		0.57	2.21	1.32	0.66	2.04	1.11	-1.16	0.76	-0.23
69.0333	C4H4OH+	Furan	0.15	0.69	0.32	0.15	0.64	0.25	-1.52	1.33	-0.34
69.0699	C5H9+	Aldehyde fragment	2.36	8.22	5.31	2.55	8.04	4.07	-1.28	0.41	-0.37
71.0491	C4H6OH+	Butenal	0.96	2.82	1.74	0.87	5.52	1.63	-1.25	1.15	-0.16
71.0854	C5H11+	3-methyl-1-butanol + 2-methyl-1-butanol, Pentanol	0.66	3.58	1.39	0.59	11.71	2.23	-1.46	2.33	0.48
73.0298	C3H4O2H+		0.88	1.87	1.09	0.8	3.52	1.1	-0.91	1.84	-0.04
73.0646	C4H8OH+	Butanale, isobutyraldehyde	1.07	2.43	1.62	2.13	12.14	4.31	0.1	3.09	1.31
75.0436	C3H6O2H+	Methyl acetate	1.09	21.3	5.6	1.28	244.82	19.06	-3.29	5.08	0.75
75.0803			0	0.14	0.02	0	0.36	0.04	-13.08	9.25	0.32
77.0223			0.07	0.37	0.22	0.17	0.4	0.23	-0.65	1.39	0.09
78.0465	C6H6+		1.61	2.62	2.01	1.7	1.96	1.83	-0.54	0.18	-0.12
79.0374	C6H7+	Benzene	0.02	2.1	0.41	0.05	10.51	0.55	-4.58	6.61	0.55
79.0739			0	0.52	0.1	0.01	2.15	0.37	-2.87	13.2	3.73
80.0559	C5[13]CH7+		0.25	1.64	0.52	0.16	2.19	0.48	-2.51	2.42	-0.23
81.0701	C6H9+	Fragment of aldehydes (Hexenals); fragment of terpenes (Linalool)	25.34	450.16	109.22	4.9	159.48	51.31	-4.93	0.89	-1
83.0492	C5H6OH+	Methylfuran	0.39	1.39	0.88	0.37	1.26	0.75	-1.19	0.88	-0.21
83.0858	C6H11+	(E)-3-Hexen-1-ol, (Z)-3-Hexen-1-ol, (Z)-2-Hexen-1-ol, Hexanal, 2-Hexanone	10.84	85.36	34.12	13.09	65.7	32.36	-1.76	1.65	0.02
85.028	C4H4O2H+	Furanone	0.13	0.23	0.17	0.11	0.21	0.16	-0.96	0.63	-0.12
85.0647	C5H8OH+	(E)-2-Pentenal	1.14	7.94	3.81	1.21	6.98	3.11	-1.37	1.02	-0.27
85.1008	C6H13+	Hexanol	0.2	0.99	0.43	0.7	7.59	2.02	0.34	4.04	2.14
87.0442	C4H6O2H+	Butyrolactone	0.56	18.83	1.77	0.55	37.82	1.99	-3.84	5.2	-0.25
87.081	C5H10OH+	2-methyl butanal+3-methyl butanal	0.5	1.45	0.89	0.5	5.08	1.26	-0.71	2.14	0.29
89.055	C4H8O2H+	Ethyl acetate	0.24	1.76	0.7	0.45	755.64	25.74	-0.96	9.32	2.52
89.1408			0	0.01	0.01	0	0.15	0.01	-2.42	6.32	0.99
89.201			0	0.02	0.01	0	0.16	0.01	-8.61	6.42	-0.11
89.2685			0	0.02	0.01	0	0.34	0.02	-9.71	5.53	0.49
91.068	C7H7+	Benzyl Alcohol	0.33	2.63	0.57	0.44	6.41	1.16	-2.38	3.87	0.76
93.0379	C3H8OSH+	2-(Methylthio)ethanol	1.1	4.01	2.22	1.89	5.72	2.8	-0.62	1.86	0.38
93.9552			0.14	0.3	0.2	0.15	0.23	0.2	-0.72	0.51	-0.03
94.0932			0.01	0.24	0.06	0.01	0.27	0.06	-4.44	2.43	-0.12
95.0188			0.33	1.12	0.62	0.52	1.04	0.72	-0.58	1.35	0.25
95.0489	C6H6OH+	Phenol	1.51	1.87	1.64	1.38	3.04	1.98	-0.32	1	0.23
95.0873	C7H11+	(E)-2-Heptenal, Monoterpene fragment	1	6.75	2.61	0.77	4.53	1.65	-1.82	0.22	-0.61
97.0274	C5H4O2H+	Furfural	0.3	0.49	0.37	0.28	0.47	0.35	-0.44	0.43	-0.06
97.0652	C6H8OH+	(E,Z)-2,4-Hexadienal, (E,E)-2,4-Hexadienal	0.34	2.3	1.01	0.26	1.54	0.57	-2.29	0.78	-0.76
99.0803	C6H10OH+	(Z)-3-Hexenal, (E)-2-Hexenal	28.44	172.73	92.5	3.38	145.1	65.19	-4.05	1.19	-0.61
101.023			0.13	0.2	0.16	0.12	0.18	0.15	-0.49	0.43	-0.13
101.061	C5H8O2H+	2,3-Pentanedione, 2-Butenoic acid methyl ester	0.25	0.58	0.36	0.29	1.38	0.47	-0.39	2.11	0.33
101.096	C6H12OH+	Hexanal	1.4	12.4	4.99	1.26	8.24	3.88	-2.33	1.86	-0.29
103.076	C5H10O2H+	Ethyl Propanoate	0.24	2.88	1.04	0.32	34.21	4.43	-1.83	4.6	1.44
105.064	C8H9+	Phenetyl Alcohol. Styrene	0.1	0.31	0.17	0.14	0.59	0.27	-0.56	2.14	0.69
106.08			0.03	0.41	0.09	0.04	0.63	0.12	-2.48	3.18	0.32
107.07	C7H6OH+	Benzaldehyde	0.1	0.9	0.29	0.2	2.86	0.59	-1.45	3.5	0.91
107.087	C8H10H+	Ethyl Benzene, p-Xylene, m-Xylene	1.93	39.61	7.62	1.58	52.78	8.76	-4.14	3.94	0
108.958			0.65	0.81	0.72	0.67	0.89	0.77	-0.15	0.27	0.1
109.034			0.09	0.29	0.18	0.12	0.19	0.15	-1.31	1.01	-0.16
109.102	C8H13+	2-Octenal (E)	1.31	7.75	3.28	2.17	7.97	3.71	-0.59	1.13	0.2
111.044			0.12	0.24	0.15	0.09	0.15	0.12	-1.13	0.18	-0.36
111.081	C7H10OH+	(E,E)-2,4-Heptadienal	0.26	0.96	0.57	0.22	0.83	0.41	-1.47	0.51	-0.45
111.117	C8H15+	(E)-2-Octenal, Octanal,1-Octen-3-ol	0.31	0.57	0.41	0.3	0.76	0.46	-0.55	1.07	0.14
113.06	C6H8O2H+	Sorbic acid	0.14	1.83	0.53	0.15	0.69	0.25	-3.36	1.01	-0.9
113.098	C7H12OH+	(E)-2-Heptenal	0.21	0.92	0.52	0.31	0.9	0.5	-0.99	0.79	-0.04
115.076	C6H10O2H+	Ethyl Crotonate, Ethyl (2E)-2-butenoate	0.2	0.45	0.29	0.24	1.73	0.57	-0.31	2.79	0.85
115.114	C7H14OH+	2-Heptanone, Heptanal	0.09	2.98	0.88	0.13	1.76	0.55	-2.78	2.07	-0.48
117.092	C6H12O2H+	Ethyl Isobutanoate, Methyl-2-methyl butanoate, Methyl Isovalerate, Ethyl Butyrate, Hexanoic Acid	0.25	5.59	0.85	0.57	56.58	10.09	-0.19	6.65	2.66
118.05			0	0.09	0.02	0	0.29	0.02	-10.62	4.12	-0.42
118.981			0	0.02	0.01	0	0.2	0.04	-0.47	5.03	1.57
119.074	C9H11+	3-Phenylpropanol	0.18	0.33	0.23	0.19	0.56	0.33	-0.25	1.23	0.47
121.068	C8H8OH+	Acetophenone, Phenylacetaldehyde	0.67	1.59	1.14	0.53	3.75	1.83	-1.16	2.48	0.47
123.048			0.11	0.21	0.17	0.14	0.33	0.19	-0.29	0.93	0.18
123.118	C9H15+	2-Nonenal, (E)-2-Nonenal	0.23	0.69	0.41	0.24	0.54	0.37	-0.89	0.65	-0.12
123.946			0.12	0.2	0.16	0.13	0.2	0.16	-0.29	0.43	0.04
125.1	C8H12OH+	6-Methyl-3,5-heptadien-2-one	0.19	0.5	0.34	0.24	1.01	0.4	-0.58	1.46	0.21
125.96			0.15	0.22	0.18	0.17	0.24	0.2	-0.2	0.46	0.12
126.904			0.3	1.84	1	0.55	1.14	0.81	-1.57	1.74	-0.02
127.072			0.08	0.4	0.16	0.07	0.16	0.11	-1.61	0.64	-0.4
127.113	C8H14OH+	1-octen-3-one, 6-methyl-5-hepten-2-one, (E)-2-Octenal.	0.48	2.86	1.39	0.85	2.68	1.48	-0.53	0.92	0.11
129.092	C8H16OH+	2-octanone, Octanal, 1-Octen-3-ol	0.12	0.18	0.15	0.15	0.68	0.26	-0.1	2.21	0.65
131.107	C7H14O2H+	Ethyl-2-methyl butanoate, Ethyl Isovalerate	0.1	2.08	0.23	0.2	32.86	4.04	0.31	7.25	3.14
133.103	C10H13+	Thymol	0.2	4.13	0.7	0.18	1.48	0.52	-2.11	2.04	-0.33
135.115	C10H15+	HO-Trienol	0.26	11.04	2.42	0.21	9.78	1.41	-3.3	0.98	-0.81
136.024			0.09	0.31	0.18	0.09	0.17	0.12	-1.78	0.67	-0.52
136.99			0	0.02	0.01	0.01	0.07	0.02	-1.06	4.18	0.76
137.134	C10H17+	1.8-cineole, Linalool, 4-Terpineol, alpha Terpineol, Nerol, Geraniol* Beta myrcene, Limonene, (E)-Beta Ocimene, Alpha Terpinolene	1.42	22.44	8.30	0.62	16.06	3.63	-3.21	0.51	-1.15
139.076	C8H10O2H+	5,5-Dimethyl-2-cyclohexen-1,4-dione	0.2	0.46	0.31	0.13	1.05	0.49	-1.33	2.36	0.47
139.117	C9H14OH+		0.16	0.43	0.25	0.16	0.56	0.24	-0.86	0.64	-0.05
141.094	C9H16OH+	2-Nonenal, (E)-2-Nonenal, Ethyl sorbate	0.15	0.24	0.19	0.14	0.27	0.2	-0.57	0.65	0.06
143.109	C8H14O2H+	(Z)-3-Hexenyl Acetate, 2-Hexenyl Acetate	0.21	0.34	0.28	0.2	0.39	0.28	-0.55	0.58	0.01
143.145	C9H18OH+	2-Nonanone, Nonanal	0.18	2.13	0.62	0.17	1.23	0.38	-1.93	0.54	-0.62
144.915			0.1	1.5	0.74	0.47	0.92	0.66	-1.53	2.78	0.52
145.124	C8H16O2H+	Ethyl Hexanoate, Hexyl Acetate, Octanoic Acid	0.25	0.51	0.37	0.32	0.66	0.46	-0.41	1	0.34
147.111			0.04	0.13	0.07	0.04	0.18	0.08	-0.66	1.65	0.27
151.113			0.11	0.4	0.21	0.13	0.3	0.18	-1.08	0.67	-0.21
157.122	C9H16O2H+	γ-Nonalactone	0.07	0.15	0.1	0.08	0.47	0.13	-0.39	1.6	0.39
157.16	C10H20OH+	Decanal	0.07	0.18	0.12	0.06	0.22	0.12	-1.12	1.38	-0.09
159.14	C9H18O2H+	Nonanoic Acid	0.42	1.16	0.75	0.61	1.41	1.07	-0.44	1.49	0.54
161.107			0.03	0.08	0.05	0.04	0.1	0.06	-0.66	0.97	0.17
163.088			0.04	0.13	0.06	0.05	0.25	0.08	-0.97	1.99	0.24
165.094			0.05	0.16	0.1	0.09	0.23	0.14	-0.39	1.63	0.45
169.126			0.05	0.11	0.08	0.05	0.11	0.07	-0.59	0.74	-0.04
171.138	C10H18O2H+	Linalool oxide, 2-Octenyl Acetate, gamma-Decalactone	0.06	0.31	0.12	0.06	0.32	0.11	-1.15	0.72	-0.08
171.177			0.05	0.53	0.13	0.02	0.25	0.06	-3.27	1.9	-0.93
173.156	C10H20O2H+	Decanoic Acid	0.07	0.3	0.18	0.11	0.39	0.25	-0.63	1.75	0.55
174.908			0.01	0.24	0.04	0.05	0.37	0.1	-0.41	3.13	1.51
175.116			0.02	0.05	0.03	0.02	0.05	0.03	-0.7	1.14	0.15
177.111	C13H21+	Geranyl Acetone	0.01	0.03	0.02	0.02	0.11	0.03	-0.51	2.32	0.3
179.111			0.02	0.06	0.04	0.03	0.67	0.14	-0.59	4.39	1.2
187.167			0.02	0.05	0.03	0.02	0.07	0.04	-1.08	1.27	0.22

Each mass peak is tentatively identified based on GC-MS analysis reported in Farneti et al. (2017b). Mass peaks values are reported as concentration (μg Kg−1). The average (three replicates for each cultivar). Minimum and maximum values are reported. In addition, the change fold values between harvest and storage assessments (expressed as SI index) are reported.

VOC profile, assessed at harvest, was characterised by mass peaks tentatively identified (t.i.) as methanol (*m/z* 33.033), acetaldehyde (*m/z* 45.031), C6-aldehydes (i.e. *m/z* 99.08; 101.09; 83.08; 81.07), monoterpenes (*m/z* 137.13), benzoic compounds (*m/z* 107.087), butyrolactone (*m/z* 87.04), and sulfuric compounds (*m/z* 34.99; 49.011; 63.033). In agreement with Farneti et al., 2017b, concentration of mass peaks related with ester compounds (i.e. *m/z* 75.043; 89.055; 103.07; 117.09; 131.107; 145.124) was very low.

VOC profile changed significantly during storage; however, any additional mass peaks were not revealed solely after cold storage. Aldehydes and terpenes decreased during storage, as confirmed by the masses *m/z* 81.07 (common fragment of C6-aldehydes and terpenes; SI: -1.00), *m/z* 99.08 (hexenal isomers; SI: -0.61), *m/z* 101.096 (hexanal; SI: -0.29), *m/z* 133.103 (thymol; SI:-033), and *m/z* 137.134 (monoterpenes; SI: -1.15). In contrast, fruit storage significantly enhanced the content of several alcohols (*m/z* 33.033, 47.043, 57.069, 71.085, 85.100) and esters (*m/z* 75.043, 89.055, 103.076, 115.076, 117.092, 131.107). Ethanol (*m/z* 47.043) was one of the compounds that increased most (SI: + 6.68), followed by acetaldehyde (*m/z* 45.031; SI: + 3.21), and by masses related with ester compounds in particular *m/z* 131.107 (t.i. as ethyl isovalerate; SI: + 3.14), *m/z* 117.092 (t.i. as ethyl isobutanoate; SI: + 2.66), or *m/z* 89.055 (t.i as ethyl acetate; SI: + 2.52).

**Figure 5:**
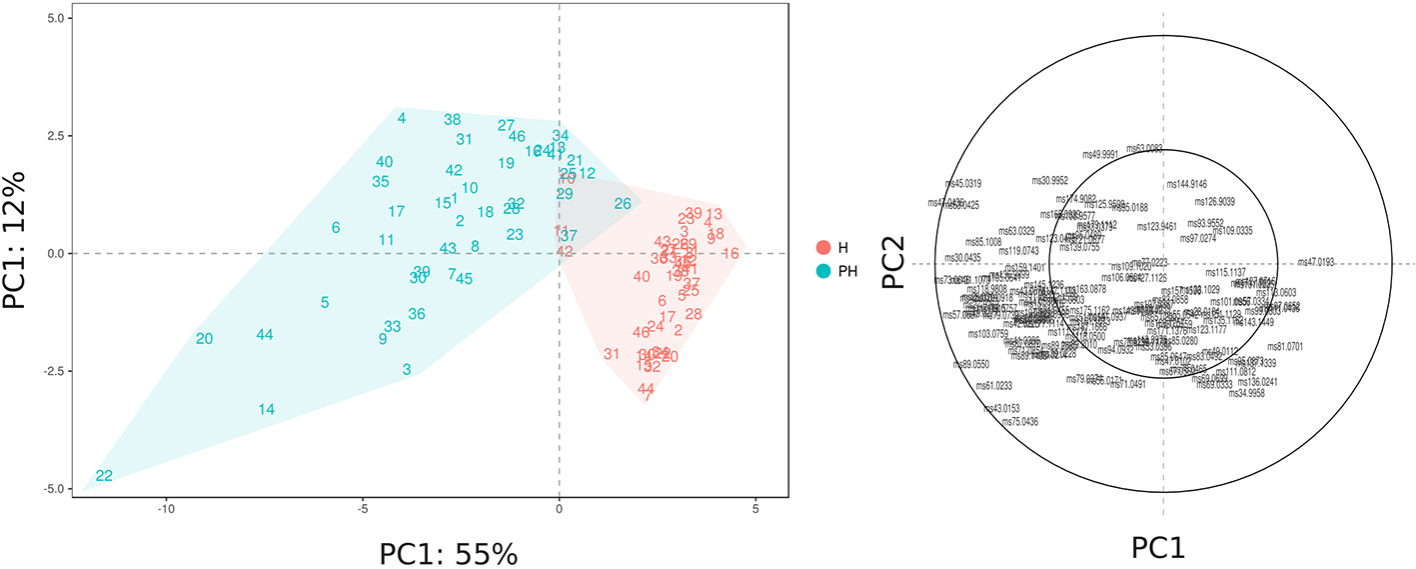
PCA and loading plot of the VOC profile of 46 *Vaccinium* spp. cultivars, assessed by PTR-ToF-MS at harvest and after storage. Each point of the PCA plot is the average of three replicates.

The VOC variability, assessed at harvest and after 6 weeks of 2°C storage, is represented by a PCA plot (**Figure 5**) defined by the first two PCs (PC1: 55% and PC2: 12%). VOC differences related to cold storage are mostly explainable by the first component (PC1) variability, that is for the most part related with differences in concentrations of esters and alcohols. Differences among *Vaccinium* genotypes, especially at harvest, are mostly related to the second component (PC2). In contrast with the outcome of texture assessment (**Figure 2**), that revealed a stronger influence of genetic variability over the storage effect, blueberry VOC profile seemed to be mostly influenced by the storage condition, but still with a significant interaction with genetic variability. As a result, blueberry cultivars, at harvest and after storage, are evidently clustered into two groups (**Figure 5**) based on PC1 variability, with only three cultivars [‘Centra Blue’ (#10), ‘Centurion’ (#11), and ‘Sky Blue’ (#42)] characterized by a VOC profile at harvest more similar to the one detected after postharvest. These cultivars are the only three *Vaccinium* hexaploid accessions (“Rabbiteye”, *V. virgatum*) taken into consideration in this study. These results confirmed that the VOC profile of “Rabbiteye” blueberry cultivars is distinguishable from other *Vaccinium* species, particularly from *V. corymbosum*, for the most part due to a higher content of esters and alcohols (Du et al., 2011; Beaulieu et al., 2014; Farneti et al., 2017b).

Results of this study revealed a strong interaction between genotype and storage treatment. Being the last products of fruit metabolic pathways, VOC emissions are highly controlled by both genetic and environmental factors, such as storage conditions. Indeed, cold storage conservation amplified VOC profile differences between cultivars, as confirmed by the increased variability in PC1 and PC2 values. For instance, cultivars characterized by a similar VOC profile at harvest, like ‘Star’ (#44) or ‘Northland’ (#31), are considerably different after storage. These differences may be the consequence of both ripening and senescence processes.

**Figure 6:**
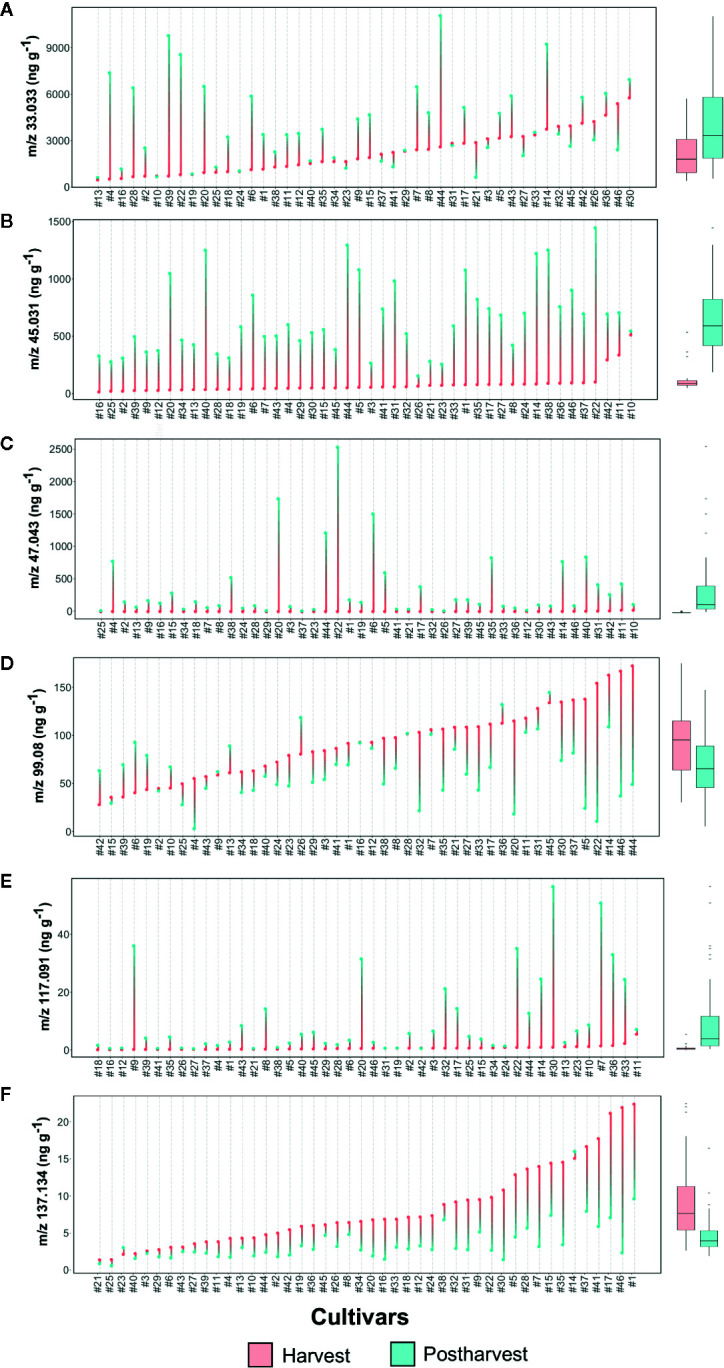
Lollipop graph and box plot of six VOC mass peaks (out of 134 detected in total by PTR-ToF-MS: **(A)** *m/z* 33.033, **(B)** *m/z* 45.031, **(C)** *m/z* 47.043, **(D)** *m/z* 99.08, **(E)** *m/z* 117.091, **(F)** *m/z* 137.134. Each graph illustrates the average value (of three measurements) recorded at harvest and after 6 weeks of storage, for each cultivar (names of the cultivars are reported in **Table 1**). For graphical purpose, cultivars of each graph are ordered based on the trait level recorded at harvest. The box plot, reported next to each lollipop graph, summarize the differences between fruit assessed at harvest and after storage. Lollipop graphs, together with distribution plots and box plots of all 134 VOC mass peaks, are reported in **Figure S4**.

Methanol (*m/z* 33.033, **Figure 6A** and **Figure S3**), being a direct product of cell wall degradation (Dorokhov et al., 2018), is commonly considered as a marker compound for fruit ripening and senescence. The content variability among the *Vaccinium* germplasm is remarkably high at both harvest and storage assessments, without any well-defined trend due to fruit conservation. Several cultivars, such as ‘Compact’ (#13), ‘Centra Blue’ (#10), and ‘Jubilee’ (#24), showed very low concentration of methanol at both stages, while others, such as ‘Azur’ (#4), ‘Jersey’ (#22), and ‘Star’ (#44), are characterized by a severe increase due to storage, independently from the concentration assessed at harvest. On the contrary, several cultivars with high methanol content at harvest, such as ‘Toro’ (#46), ‘Liberty’ (#26), and ‘Top Hat’ (#45), significantly reduced their content after storage.

Ethanol is another VOC ordinarily considered as a reliable marker of fruit senescence (Pesis, 2005). After storage, only 15 cultivars of our germplasm selection had an elevated (over 250 ppb) concentration of ethanol (*m/z* 47.043, **Figure 6C**, **Figure S4**). Among them, ‘Jersey’ (#22), ‘Emerald’ (#20), ‘Biloxy’ (#6), and ‘Star’ (#44), are the cultivars with the highest ethanol concentration.

Variation of methanol and ethanol after storage did not show any significant correlation with the texture parameters previously presented (**Figure S5**). Indeed, among cultivars with the highest ethanol production, only ‘Jersey’ (#22) revealed a higher deterioration of the textural parameters (gradient, maximum force, and force strain) while ‘Emerald’ (#20), ‘Biloxy’ (#6), and ‘Star’ (#44) showed average textural parameters with almost any substantial alterations of Max force values.

Another VOC related to fruit ripening and senescence is acetaldehyde (Pesis, 2005). Results of this study confirmed that acetaldehyde content increased in blueberry fruit during cold storage, as previously demonstrated on a reduced genotype collection by Farneti et al. (2017b). All germplasm accessions of this study increased in acetaldehyde content (*m/z*/45.031, **Figure 6B**, **Figure S4**) during storage. Most of the cultivars, except the *V.* *virgatum* ones [‘Centra Blue’ (#10), ‘Centurion’ (#11), and ‘Sky Blue’ (#42)], presented an extremely low concentration of acetaldehyde at harvest. Variation during storage is related to genotypes: results of this experiment showed cultivars with a low increase, such as ‘Centra Blue’ (#10), ‘Liberty’ (#26), and ‘Aurora’ (#3), and others with a more predominant increase, such as ‘Jersey’ (#22), ‘Star’ (#44), and ‘Safir’ (#40). Cultivars with a high acetaldehyde production had also a high content of methanol and ethanol, but no statistically significant positive correlation was found. Nevertheless, a significant positive correlation is obtained linking SI values of ethanol and acetaldehyde (**Figure S5**), suggesting that acetaldehyde is synthetized in blueberry fruit from ethanol by alcohol dehydrogenases activity as for many other fruit species (Chervin et al., 1999; Tadege et al., 1999; Pesis, 2005).

Postharvest storage significantly improved the concentration of ester compounds, particularly the ones identified with the mass peaks *m/z* 75.043, 89.055, 103.076, 115.076, 117.092, and 131.107. Most of these esters were assessed only in trace amount at harvest; remarkably, only some cultivars significantly enhanced their concentration after storage. All these ester compound mass peaks were not strictly correlated to each other (**Figure S5**), indicating different metabolic pathways involved in their synthesis as well as an evident effect of genotype. Mass peak *m/z* 117.092, tentatively identified as C6 esters, such as ethyl isobutanoate, methyl-2-methyl butanoate, methyl isovalerate, and ethyl butyrate, increased during storage in more than half of the cultivars (**Figure 6E**, **Figure S4**). Notably, for most of the cultivars [i.e. ‘North Blue’ (#30), ‘Bluecrop’ (#7), and ‘Brigitta Blue’ (#9),] the increase of m/z 117.092 concentration was not correlated with the variation of neither ethanol and methanol, nor textural properties (**Figure S5**).

Ethyl acetate (*m/z* 89.055) is another ester compound measured at high concentration after storage, but only in a limited number of accessions (**Figure S4**). As it has already been reported in several fruit species, ethyl acetate content is often highly correlated with ethanol and acetaldehyde levels (Knee and Hatfield, 1981; Larsen, 1995). In this study, high concentrations of *m/z* 89.055 are positively correlated with the content of ethanol and methanol as evidenced in the cultivars ‘Jersey’ (#22), ‘Emerald’ (#20), ‘Cosmopolitan’ (#14), ‘Berkeley’ (#5), and ‘Star’ (#44) (**Figure S5**).

Aldehyde and terpene content normally decrease during the last ripening phases of blueberry fruit, as previously demonstrated by Farneti et al. (2017b). In this study, decay of these compounds was evident also for fruit after storage, with variations in concentration that are evidently cultivar dependent. C6 aldehydes, for most hexenal isomers (*m/z* 99.08) and hexanal (*m/z* 101.096), diminished during storage in a genotype dependent manner with cultivars, such as ‘Jersey’ (#22), ‘Toro’ (#46), ‘Star’ (#44), or ‘Berkeley’ (#5) characterized by a SI lower than -2, and, on the contrary, several nearly stable cultivars, such as ‘Darrow’ (#16), ‘Misty’ (#28), or ‘Bluecrop’ (#7) (**Figure 6D**, **Figure S4**). Hexenal isomers and hexanal contents were highly correlated at both harvest and storage phases, even if these compounds are derived by different free fatty acid precursors, linolenic and linoleic acid respectively (Klee and Tieman, 2018; Ferrão et al., 2020). Based on these outcomes, it is impossible to predict C6 aldehyde content after storage of a blueberry cultivar based solely on the assessment at harvest. Several cultivars, such as ‘Berkeley’ (#5), ‘Emerald’ (#20), ‘Jersey’ (#22), ‘Star’ (#44), or ‘Toro’ (#46), were characterized by the lowest content of hexenal isomers (*m/z* 99.08) despite their high values at harvest. On the contrary, other cultivars with low values at harvest, like ‘Biloxy’ (#6), ‘Centra Blue’ (#10), ‘Eliott’ (#19), ‘Rubel’ (#39), or ‘Sky Blue’ (#42), showed positive SI values and the highest concentrations after storage. In addition, reinforcing the high variability among the *Vaccinium* germplasm, there were also accessions with stable hexenal content during storage, independent of the concentration assessed at harvest, such as ‘Atlantic’ (#2), ‘Bluecrop’ (#7), ‘Brigitta Blue’ (#9), ‘Chandler’ (#12), ‘Darrow’ (#16), or ‘Northblue’ (#30).

Monoterpenes (*m/z* 137.133) were the VOC class most negatively affected by fruit storage (**Figure 6F**, **Figure S4**). The high variability in monoterpenes content assessed at harvest was extremely reduced after storage. Indeed, cultivars, like ‘Aron’ (#1), ‘Toro’ (#46), ‘Early Blue’ (#17), ‘Simultan’ (#41) or ‘Puru’ (#37) showed very low monoterpene content after storage in spite of the high concentration assessed at harvest. Among monoterpene high-ranking cultivars at harvest, only ‘Cosmopolitan’ (#14) was stable after storage.

Another relevant mass peak for the characterization of blueberry VOC profile was *m/z* 107.086 (**Figure S4**), that is the characteristic fragment of compounds containing a benzoic ring, like ethyl benzene or xylene. No significant differences were detectable based on average values obtained at harvest and after storage, suggesting a stability of this trait during cold storage. Nevertheless, SI values showed an extremely high variability between genotypes, with values ranging between -4 to +4. For instance, cultivars like ‘Centurion’ (#11), ‘Aurora’ (#3), ‘Mondo’ (#29), or ‘Elizabeth’ (#18), revealed high concentration of *m/z* 107.086 after storage despite the very low concentration at harvest. On the contrary, cultivars like ‘Southern belle’ (#43), ‘Rubel’ (#39), ‘Nui’ (#32), ‘Hortblue Poppins’ (#35), or ‘Atlantic’ (#2) were highly ranked at harvest but showed very low values after storage.

### Cultivar Characterization Based on Storage VOC Modification

**Figure 7:**
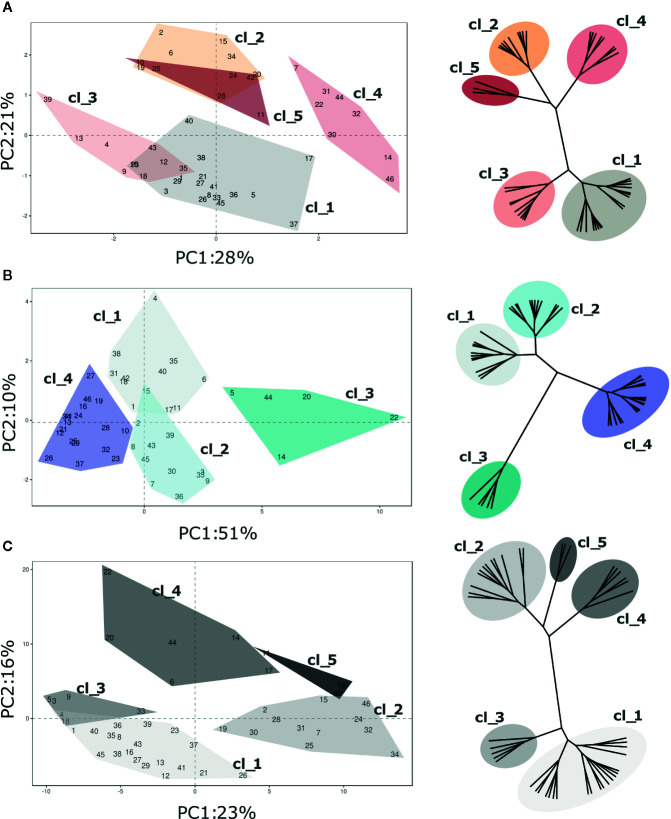
PCA and hierarchical clustering (based on Ward distances) of 46 *Vaccinium* spp cultivars based on VOC profile assessed at **(A)** harvest and **(B)** postharvest, and on the calculated storage index **(C)**. Each point of the PCA plot is the average of three measurements. Loading plots of each PCA are reported in the supplementary **Figure 6**. Clusters highlighted in each PCA plot corresponds to the clusters identified by Ward clustering.

VOC profiling of the blueberry germplasm collection was further analyzed by separately considering the assessments at harvest and after storage, as formerly described for texture. Differently from texture results, the overall VOC profile variability was only partially explained by considering the first two principle components (**Figure 7** and **Figure S6**; Harvest 49%; Postharvest, 61%; SI, 39%).

Nevertheless, *Vaccinium* cultivars were divided into statistically significant clusters, based on Ward hierarchical clustering of the VOC profile, according to the moment of assessment (harvest and post-harvest) and to the storage index (SI). Due to the high multidimensionality of VOC profiles, clusters, based on Euclidean’s distances, were not always properly separated over the bidimensional space produced by PCA analysis. In addition, excluding the three *Vaccinium* virgatum accessions, the profiling based on VOC production did not reveal any significant association with the genetic molecular profile based on six SSRs (**Figure S4** and **Figure S6**).

Five clusters of cultivars were statistically distinguishable at harvest based on VOC composition (**Figure 7A**; **Figure S4** and **Figure S6**). Since only 49% of the overall VOC variability is explained by the first two components, the graphical clusters separation in the PCA plot is not sufficiently effective. For instance, the separation of clusters “Cl_2” and “Cl_5” is clearly distinguishable only after adding the third dimension (data not shown). The variability expressed by the first two components is mostly associated with a reduced VOC array, for the most part related to C6 aldehydes and alcohols (*m/z* 81.07, 83.085, 99.08, 101.096), terpenes (*m/z* 137.134), methanol (*m/z* 33.033) and methyl acetate (*m/z* 75.043). Clusters “Cl_1” and “Cl_4” were composed by cultivars with high content of C6 aldehydes and terpenes, such as ‘Toro’(#46), ‘Northland’(#31), ‘Puru’ (#37), ‘Cosmopolitan’ (#14) or ‘Jersey’(#22). On the contrary, accessions belonging to “Cl_3” and “CL_2”, for instance ‘Atlantic’ (#2), ‘Biloxi’ (#6), ‘Southern Belle’ (#43), or ‘Legacy’ (#25), had a less intense VOC profile. The three *V.* *virgatum* accessions which clustered into “Cl_5”, namely ‘Centra Blue’ (#10), ‘Centurion’ (#11), and ‘Sky Blue’ (#42), were characterized by very low concentration of terpenes and C6 aldehydes, and high content of acetaldehyde (*m/z* 45.031), ethanol (*m/z* 47.043) and esters (*m/z* 61.023 75.043, 117.091, 131.107).

After fruit storage, the VOC variability expressed by the first two components of the PCA analysis increased up to 61% (**Figure 7B**, **Figure S4** and **Figure S6**). Most of this variability was covered by the first component (PC1, 51%), that is highly interrelated with C6 aldehydes (*m/z* 81.070, 99.08, 101.096), C6 alcohols (*m/z* 83.085, 85.100), terpenes (*m/z* 137.134), and with VOCs that were not so relevant to discern cultivars at harvest, principally ethanol (*m/z* 47.043), acetaldehyde (*m/z* 45.031), and several ester compounds (*m/z* 61.023, 75.043, 89.055, 103.076, 117.091, 131.107). Based on that, *Vaccinium* accessions of this study were grouped into four clusters. Accessions with the most modified VOC profile were clustered into “Cl_3”. That cluster is composed only of five accessions, namely ‘Berkeley’(#5), ‘Cosmopolitan’(#14), ‘Emerald’(#20), ‘Jersey’(#22), and ‘Star’(#44), characterized by the highest concentrations of ethanol, acetaldehyde, methyl acetate (*m/z* 75.043) and ethyl acetate (*m/z* 89.055). This alteration of the VOC profile after postharvest storage may be linked to a raise in fermentation processes due to fruit overripening. Differently, VOC profile of “Cl_2” accessions was for the most part characterized by low concentrations of ethanol and acetaldehyde together with high contents of some ester compounds (*m/z* 75.043, 103.076, 117.091, 131.107), especially the cultivars ‘Aurora’(#3), ‘Brigitta Blue’ (#9), ‘North Blue’(#30), ‘O’Neal’ (#33), and ‘Rubel’(#39). Cultivars of the two remaining clusters, “Cl_1” and “Cl_4”, were defined by low concentrations of most compounds, except for C6 aldehydes (*m/z* 81.070, 99.08, 101.096) and C6 alcohols (*m/z* 83.085, 85.100).

The first two PCA components, assessed over the SI values, explained only 39% of the VOC profile variability (**Figure 7C**). This low percentage is mostly due to the high statistical noise of the dataset, introduced by the SI calculation (logarithmic of the molecule concentration ratio assessed before and after fruit storage). Based on that, several molecules that were assessed at extremely low concentration, most probably far below their threshold level, might have a significantly high relevance in the statistical analysis (i.e. minimum and maximum concentration of *m/z* 89.201 ranging between 0.00 and 0.16 ppb, resulting in SI values between -8.61 and +6.42). Nonetheless, *Vaccinium* accessions considered in this study were statistically divided into five clusters based on Euclidean’s distances (**Figure 7C**, **Figure S4** and **Figure S6**). Clusters “Cl_1” and “Cl_2” grouped cultivars with a more stable VOC profile during storage, such as ‘Aron’(#1), ‘Blue Moon’ (#8), ‘Chandler’(#12), ‘Northland’(#31), or ‘Ozark Blue’(#34). Accessions belonging to cluster “Cl_1” differed by a higher average SI value of some ester related masses (*m/z* 43.015, 61.023, 75.043, 89.055, 103.076). Clusters “Cl_3” and “Cl_4” grouped cultivars (i.e. ‘Biloxi’ (#6), ‘Brigitta Blue’ (#9), ‘Emerald’ (#20), ‘Jersey’(#22), or ‘Star’ (#44)) characterized by high SI values of masses related to esters (*m/z* 43.015, 61.023, 75.043, 89.055, 103.076, 117.091, 131.107), acetaldehyde (*m/z* 45.031), and ethanol (*m/z* 47.043), but low SI values of C6 aldehydes (*m/z* 81.070, 99.08, 101.096). The latter cluster, “Cl_5”, included only the three *V.* *virgatum* cultivars [‘Centra Blue’ (#10), ‘Centurion’ (#11), and ‘Sky Blue’ (#42)] that differed from the other accessions for the higher stability of C6 aldehydes (*m/z* 99.08, 101.096), C6 alcohols (*m/z* 83.085 and 85.100), ethanol (*m/z* 47.043), and acetaldehyde (*m/z* 45.031).

## Discussion

Until now, as for many horticultural fruit species (Folta and Klee, 2016; Klee and Tieman, 2018) blueberry breeding selection has been mostly oriented on the amelioration of agronomic traits, such as flowering time, chilling requirements or plant structure, and on standardising the physical-chemical quality traits of the fruit at the time of harvest, ignoring the possible storage effect (Gilbert et al., 2014). Indeed, quality assessments at harvest revealed a limited variability, especially for VOC content, in comparison with the high genetic variability of the *Vaccinium* accessions employed in this study. For this reason, an accurate and objective post-harvest characterization of each accession, based on each quality trait, is necessary for the selection of the optimal parental choice and the best progenies oriented towards distinct market sectors.

The genetic analysis of the plant materials showed that the individuals under investigation are unique genotypes and that the hexaploid *Vaccinium* accessions are genetically diverse and cluster distinctly compared to the tetraploid accessions. On the other hand, there is no evidence of strong genetic structure among the tetraploid cultivars even if distinct clusters for northern and southern highbush blueberry cultivars can be clearly defined. Results of the cluster analysis agreed to what it is reported so far in blueberry (Boches et al., 2006; Bassil et al., 2020). The grouping of highbush blueberry in two main clusters of southern and northern highbush was also visible, as expected, despite some exceptions for which the limiting factor could be also the number of SSRs. In addition, Bassil et al. (2020) recently proposed two new set of markers (5 to 10) including some of the SSRs used in this paper to solve blueberry genotypes, and they showed that the 5-set markers failed in discriminating only two genotypes out of 367 accessions of the USDA germplasm. However, in our study, this genetic clustering could not be correlated, for most of the accessions, either to fruit texture parameters, or to fruit VOCs. Indeed, the choice of parental lines based uniquely on accession’s pedigree or genetic similarities, based on six SSRs, is not enough for a parental choice aimed to improve fruit quality. Moreover, that suggests an overall standardization of blueberry fruit quality that has been reached by breeding activity during these years. However, textural and VOC variability among accessions increased after storage, with clusters of cultivars being more distinguishable based on textural and VOC attributes. For instance, concerning texture results, we could identify cultivars that became more turgid and harder after storage while others lost their turgidity and became softer. Regarding VOCs, instead, several cultivars preserved their profile similarly to the one assessed at harvest, while others considerably altered their VOC profile for the most part enhancing the concentration of esters and other compounds associated with fruit fermentation and deterioration, like ethanol and acetaldehyde (Pesis, 2005). Since blueberry fruit is mostly consumed after storage, often after long transcontinental shipments, these findings raised the importance for breeders to evaluate new varieties’ quality also after a storage period that simulates commercial requirements.

This high variability on quality traits observed after storage might be determined by genetic differences that regulate fruit physiological, chemical and physical features. According to published studies (Giongo et al., 2013) and ongoing experiments on both segregating population and broad germplasm collection, this lack of straightforward relationship between harvest and postharvest quality features seems to be genotype-dependent. Physiological changes associated with ripening, such as firmness decay and flavours and off flavours production, are coordinated by a complex network of endogenous hormones, for the most ethylene and ABA. Nevertheless, there is still no consensus on whether blueberry is a climacteric fruit or not (MacLean and Scott NeSmith, 2011). Although a peak in respiration and ethylene production has been observed in blueberry in some studies (Windus et al., 1976; El-Agamy et al., 1982), this was not conclusive in others (Frenkel, 1972). Recent studies confirmed a complex interaction between ethylene softening and sucrose metabolism in blueberry fruit (Wang et al., 2018; Wang et al., 2020). This complexity is evident in the number and type of cell wall-modifying genes (i.e. VcPE and VcPG) and the different ways in which they are regulated. On the contrary, other studies revealed an important role of ABA on fruit ripening regulation (Karppinen et al., 2018; Oh et al., 2018). Post-harvest ABA treatment during bilberry (*Vaccinium myrtillus*) fruit ripening led to the induction in the expression of genes associated with cell wall modifications (Karppinen et al., 2018). Among these, ABA induced genes encoding pectin-modifying enzymes (i.e. VmPL, VmRGLyase, VmβGAL1, and VmβGAL2) as well as genes involved in depolymerization of hemicellulose (i.e.VmXTH and VmCEL) and expansins (i.e. VmEXP1, VmEXP2, and VmEXP3).

Taking into account the high genetic variability considered in this study, we aimed to uncover most of the blueberry texture and VOC variability. However, without a detailed sensory analysis, quantifying the relevance of each trait might be too speculative, especially for VOCs, bearing also in mind the non-linear interaction of these molecules in determining the consumer preference. For this reason, in order to reduce any possible statistical bias in the result interpretation, all data were analysed with unsupervised multivariate statistical methodologies (PCA and hierarchical clustering). Nonetheless, considering each quality trait independently (i.e. **Figure S1** and **Figure S4**) might be useful for the backcross breeding approach, aimed to introduce, or improve, a distinct quality trait to an elite breeding line. To simplify the application of these results, we limited the number of texture and VOC traits that have to be considered (**Figure S7**), according to the loading plots of the principle component analysis and to the results of previously published articles (Giongo et al., 2013; Farneti et al., 2017b). The content of each trait was grouped based on the distribution quantile (low: 0%‑25%; middle-low: 25%‑50%; middle-high:50%‑75%; high:75%‑100%), calculated for both harvest and postharvest assessment (**Figure S7**). Accessions employed in the study can be consequently sorted and clustered according to the content of the trait of interest, that can be arbitrarily chosen.

Until now only the research of Ferrão et al. (2020) reports results on employing molecular markers in the selection of blueberry fruit for flavour. Indeed, metabolite genome-wide association analysis (GWAS) elucidated the genetic architecture and demonstrated that blueberry VOC synthesis can be accurately predicted using genomic information. Nonetheless results of that investigation were only based on blueberry quality traits recorded at harvest. Moreover VOCs, for which their genomic regions were detected, showed an extremely low (i.e. linalool) or even negative (i.e. eucalyptol) correlation with consumer taste preferences (Gilbert et al., 2015; Ferrão et al., 2020). Considering that no molecular markers are yet available to predict VOC and texture variations during blueberry storage, the application of reliable phenotyping techniques is still essential to support breeding activity.

**Figure 8:**
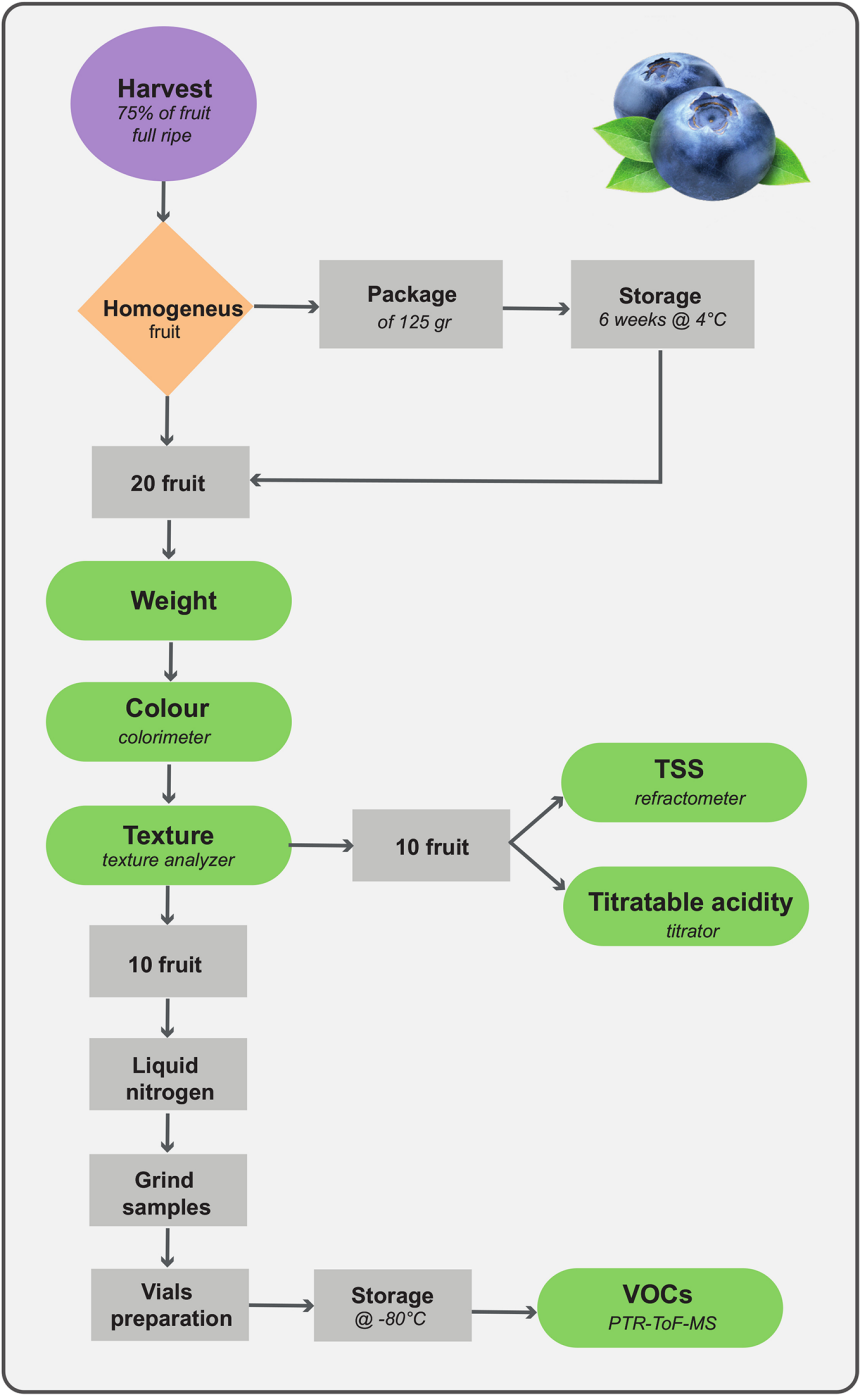
Phenotyping assessment of quality traits applicable for blueberry breeding screening.

Analytical methodologies considered in this study may result in powerful tools for phenotyping quality traits and, at the same time, in developing genetic markers that help to screen blueberry populations. The phenotyping approach suggested in our research, detailed in **Figure 8**, provides the opportunity to use fast and high-throughput techniques to assess a broad number of samples by relatively unskilled personnel, and to follow fruit quality changes during storage. Storage requirements, for instance time length, temperature or atmospheric gas composition, must be optimized according to the breeding objectives (i.e. selection for local market or for overseas exportation). In our case, we established the postharvest assessment after 6 weeks of storage since this prolonged storability is one of the breeding targets requested to extend the Italian blueberry supply.

Analytical detection of traits concerning fruit taste perception, for most part acidity and sweetness, can still be based on refractometer and titrator. Despite the simplicity and lack of sophistication, results obtained by these techniques are properly linked with consumer perception (Gilbert et al., 2015). Nonetheless, it is desirable, in the near future, to use more accurate and high-performance mass spectrometric analytic techniques based, in our opinion, on direct analysis, such as DART-MS (Chen et al., 2007; Guo et al., 2017). Therefore, beside a better identification of compounds determining taste perception, a rapid quantification of blueberry nutraceutical compounds might also be possible.

Fruit texture, is also one of the main quality traits driving consumer preference (Gilbert et al., 2015; Ferrão et al., 2020). In our opinion, being blueberry texture determined by several physical constrains, such as cell turgor, peel elasticity, and cell wall structure, the destructive evaluation of blueberry fruit by texturometer is preferable than the non-destructive assessments based on fruit compression (i.e. Firmtech II; Li et al., 2011; Giongo et al., 2019), laser induced method (Li et al., 2011), hyperspectral imaging (Hu et al., 2015), or Vis-NIR spectroscopy (Hu et al., 2018). Application limits of these non-destructive techniques are still the need of a constant updated calibration of the predictive multivariate algorithm, and the low spatial and spectral resolution (Li et al., 2019). Moreover, for the phenotyping pipeline that we proposed (**Figure 8**) the destructive assessment of texture is not a limiting factor, since analysed fruit can be employed for the analysis of other quality traits, such as total soluble solids, titratable acidity, and VOCs. In that case, the instant freezing of samples using liquid nitrogen, is essential to preserve the organoleptic characteristics of the fruit, that, on the contrary, might be altered by fruit cutting and air exposure.

Bearing in mind that the aim of VOC assessment is to obtain an objective estimation of the aroma perceived by the consumer during fruit consumption, we consider worthless the application of too aggressive chemical extraction methodologies (i.e. liquid-liquid extraction with hexane or dichloromethane). These methodologies are only necessary for the quantification of compounds at extremely low concentrations that, in case of blueberry fruit, might be under the perception threshold of the consumer. Furthermore, we need high resolution analytical techniques, able to identify and quantify, at once, molecules with different polarity and molecular weight (i.e. methanol and sesquiterpenes) present in a broad concentration range (from ppt to ppm). The extreme complexity of food aroma composition is a challenging issue for any existing analytical technology. The rapid development of mass spectrometry (MS) application in metabolomic studies had a significant impact in the field of VOC analysis. Most of the progresses of MS techniques are focused on instrumental improvements of mass resolution, mass accuracy, sensitivity, and enhanced reproducibility. PTR-MS is particularly suited to develop reliable food VOC fingerprints because it provides handier analytical information (concentration estimation and reduced fragmentation) in comparison with the application of MS-e-noses based on electron impact ionization (Biasioli et al., 2011). PTR-ToF-MS, equipped with multipurpose auto-samplers, provides a rich, informative, and high-throughput fingerprint. This study supports the results of Farneti et al. (2017b), confirming that blueberry VOC profile can be accurately assessed by direct injection techniques. One of the aims of this research was the untargeted analysis by PTR-ToF-MS to disclose VOC differences among blueberry accessions due to genetic differences and prolonged fruit storage, while in the previous research (Farneti et al., 2017b) we mostly focused on differences related with blueberry fruit ripening. Pulling together results of these two investigations, the array of mass peaks suitable to describe most of blueberry VOC variability can be considerably reduced. This information can be applied to target VOC assessment for both breeding selection and quality control within the entire production chain, by adopting less performing, but more handy and inexpensive, direct-injection instruments with a quadrupole mass spectrometer (i.e. PTR-MS or SIFT-MS; Vendel et al., 2019).

This investigation, together with recent findings on blueberry flavour (Ferrão et al., 2020), suggests an accurate and objective road map for *Vaccinium* flavor improvement. A better understanding of genes and enzymes involved in the VOC production and textural modification is still needed. This could lead to genetic and environmental manipulations to optimize aroma at the time of consumption, following shipping and marketing. To this end, future breeding programs focused of prolonged fruit post-harvest storage need to consider blueberry VOC modification. This can be achieved only with a more informed and precise identification of the best performing cultivars to be used as superior parental lines in combination with a reliable phenotyping methodology and molecular markers.

## Data Availability Statement

All datasets presented in this study are included in the article/**Supplementary Material**.

## Author Contributions

BF designed the research, analyzed and interpreted data, and wrote the manuscript. FM carried out the genetic structure analysis. IK helped with PTR-ToF-MS data analysis. MA assessed the texture analysis and sampled the blueberries. FB guided the PTR-ToF-MS analysis and edited the manuscript. LG coordinated the work design and edited the manuscript.

## Funding

This work was financially supported by the AdP of the PAT (Provincia Autonoma di Trento) and partially by the project AppleBerry (L6/99 of the PAT).

## Conflict of Interest

The authors declare that the research was conducted in the absence of any commercial or financial relationships that could be construed as a potential conflict of interest.
