# Supplementary material for: Development of a Novel Phenotypic Roadmap to Improve Blueberry Quality and Storability
Source: Front Plant Sci. 2020 Aug 14;11:1140. doi: 10.3389/fpls.2020.01140 (PMC7456834; doi:10.3389/fpls.2020.01140)
Supplement: Supplementary file 1 [file DataSheet_1.pdf]

Figure S1. Lollipop graphs (a). distribution plots (b) and box plots (c) of all texture parameters. Clusters of box plot analysis were determined based on Ward hierarchical clustering presented in **figure 4** and **table S3**

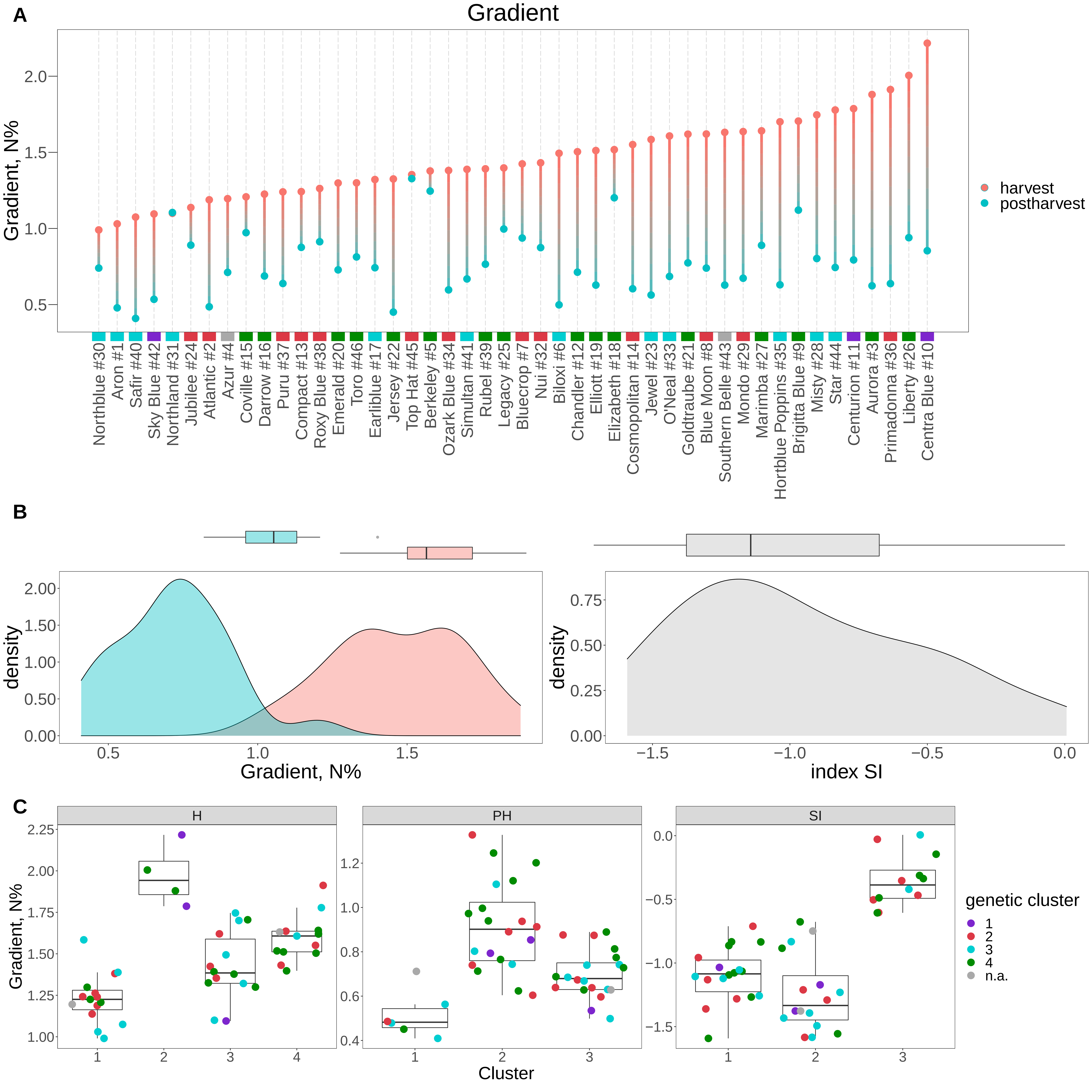
A
Gradient
Gradient, N%
harvest
postharvest
Northblue #30
Aron #1
Safir #40
Sky Blue #42
Northland #31
Jubilee #24
Atlantic #2
Azur #4
Coville #15
Darrow #16
Puru #37
Compact #13
Roxy Blue #38
Emerald #20
Toro #46
Earliblue #17
Jersey #22
Top Hat #45
Berkeley #5
Ozark Blue #34
Simultan #41
Rubel #39
Legacy #25
Bluecrop #7
Nui #32
Biloxi #6
Chandler #12
Elliott #19
Elizabeth #18
Cosmopolitan #14
Jewel #23
O'Neal #33
Goldtraube #21
Blue Moon #8
Southern Belle #43
Mondo #29
Marimba #27
Hortblue Poppins #35
Brigitta Blue #9
Misty #28
Star #44
Centurion #11
Aurora #3
Primadonna #36
Liberty #26
Centra Blue #10
B
density
Gradient, N%
index SI
C
H
PH
SI
Cluster
genetic cluster
n.a.

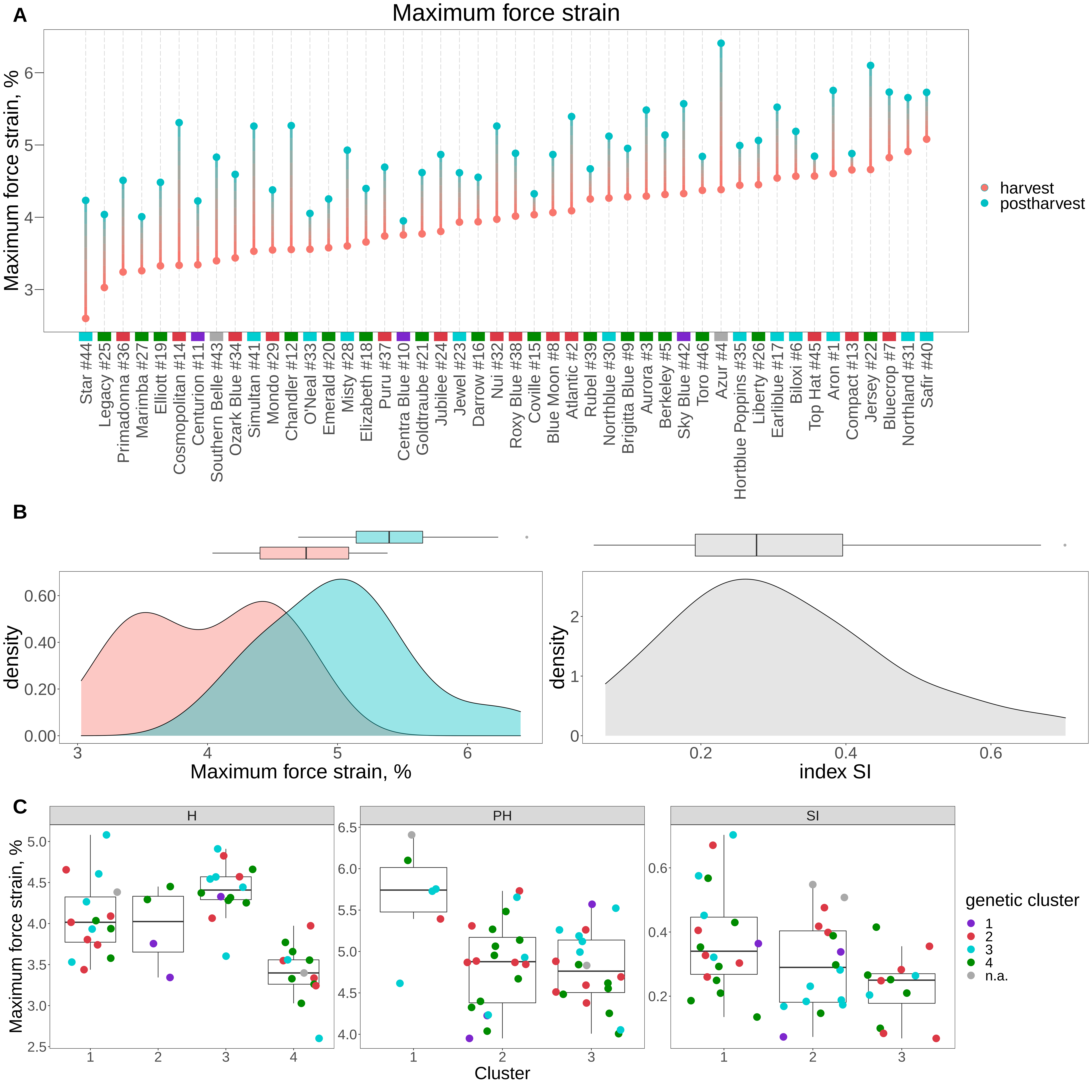
A
Maximum force strain
Maximum force strain, %
harvest
postharvest
Star #44
Legacy #25
Primadonna #36
Marimba #27
Elliott #19
Cosmopolitan #14
Centurion #11
Southern Belle #43
Ozark Blue #34
Simultan #41
Mondo #29
Chandler #12
O'Neal #33
Emerald #20
Misty #28
Elizabeth #18
Puru #37
Centra Blue #10
Goldtraube #21
Jubilee #24
Jewel #23
Darrow #16
Nui #32
Roxy Blue #38
Coville #15
Blue Moon #8
Atlantic #2
Rubel #39
Northblue #30
Brigitta Blue #9
Aurora #3
Berkeley #5
Sky Blue #42
Toro #46
Azur #4
Hortblue Poppins #35
Liberty #26
Earliblue #17
Biloxi #6
Top Hat #45
Aron #1
Compact #13
Jersey #22
Bluecrop #7
Northland #31
Safir #40
B
density
Maximum force strain, %
index SI
C
H
PH
SI
Cluster
genetic cluster
1
2
3
4
n.a.

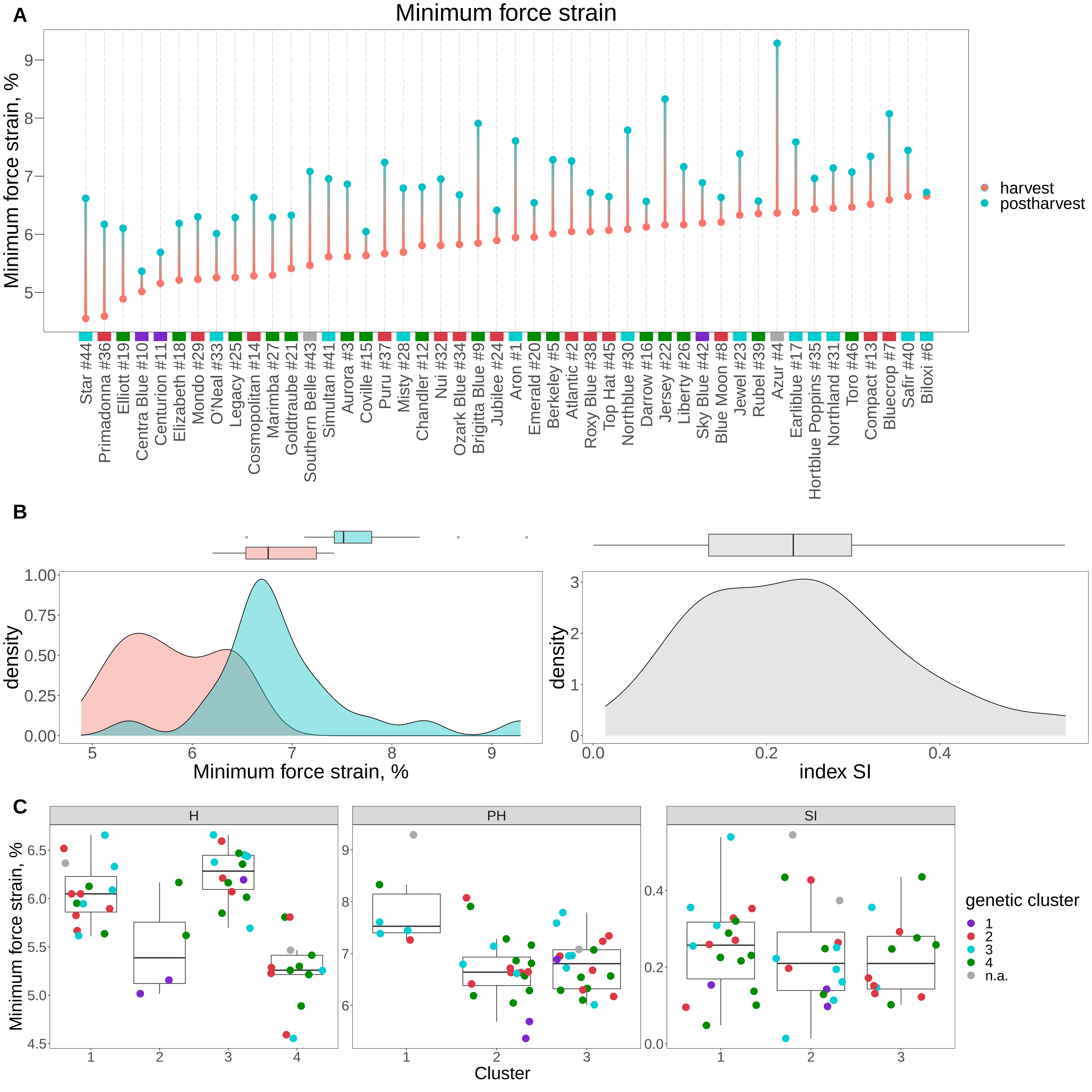
A
Minimum force strain
Minimum force strain, %
harvest
postharvest
Star #44
Primadonna #36
Elliott #19
Centra Blue #10
Centurion #11
Elizabeth #18
Mondo #29
O'Neal #33
Legacy #25
Cosmopolitan #14
Marimba #27
Goldtraube #21
Southern Belle #43
Simultan #41
Aurora #3
Coville #15
Puru #37
Misty #28
Chandler #12
Nui #32
Ozark Blue #34
Brigitta Blue #9
Jubilee #24
Aron #1
Emerald #20
Berkeley #5
Atlantic #2
Roxy Blue #38
Top Hat #45
Northblue #30
Darrow #16
Jersey #22
Liberty #26
Sky Blue #42
Blue Moon #8
Jewel #23
Rubel #39
Azur #4
Earliblue #17
Hortblue Poppins #35
Northland #31
Toro #46
Compact #13
Bluecrop #7
Safir #40
Biloxi #6
B
density
Minimum force strain, %
density
index SI
C
H
PH
SI
Minimum force strain, %
Cluster
genetic cluster
1
2
3
4
n.a.

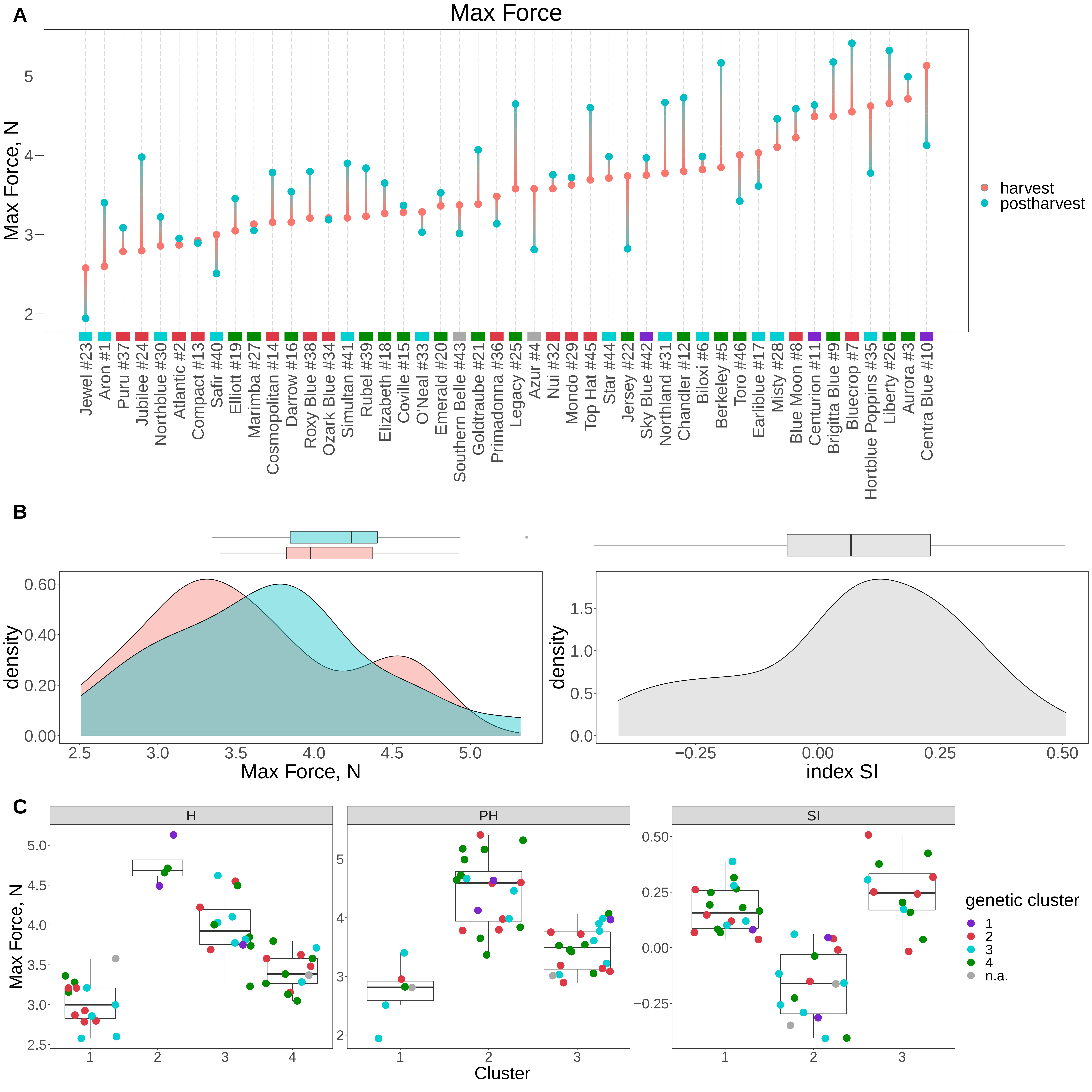
A
Max Force
Max Force, N
harvest
postharvest
Jewel #23
Aron #1
Puru #37
Jubilee #24
Northblue #30
Atlantic #2
Compact #13
Safir #40
Elliott #19
Marimba #27
Cosmopolitan #14
Darrow #16
Roxy Blue #38
Ozark Blue #34
Simultan #41
Rubel #39
Elizabeth #18
Coville #15
O'Neal #33
Emerald #20
Southern Belle #43
Goldtraube #21
Primadonna #36
Legacy #25
Azur #4
Nui #32
Mondo #29
Top Hat #45
Star #44
Jersey #22
Sky Blue #42
Northland #31
Chandler #12
Biloxi #6
Berkeley #5
Toro #46
Earliblue #17
Misty #28
Blue Moon #8
Centurion #11
Brigitta Blue #9
Bluecrop #7
Hortblue Poppins #35
Liberty #26
Aurora #3
Centra Blue #10
B
density
Max Force, N
index SI
C
H
PH
SI
Max Force, N
Cluster
genetic cluster
1
2
3
4
n.a.

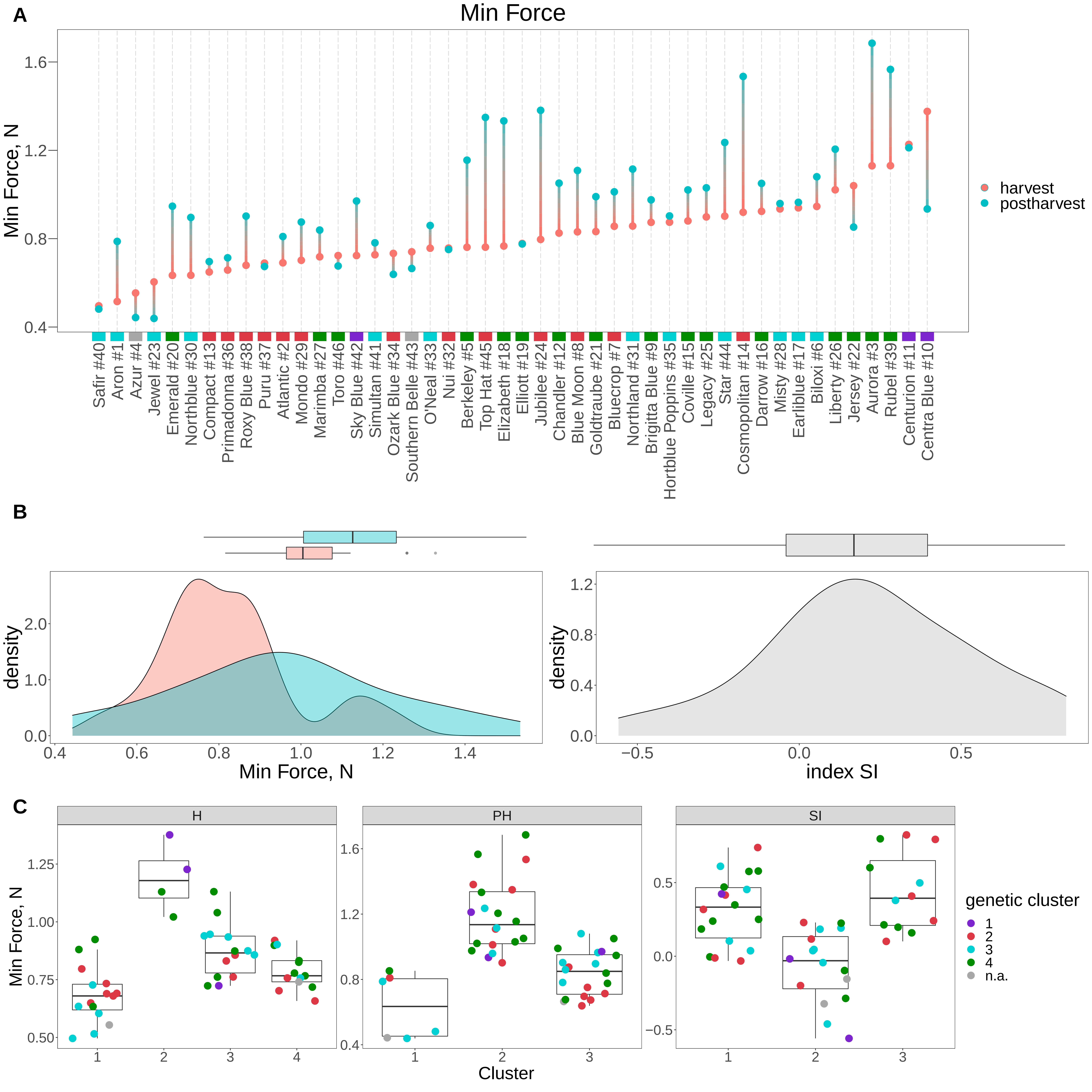

A
Min Force
Min Force, N
harvest
postharvest
Safir #40
Aron #1
Azur #4
Jewel #23
Emerald #20
Northblue #30
Compact #13
Primadonna #36
Roxy Blue #38
Puru #37
Atlantic #2
Mondo #29
Marimba #27
Toro #46
Sky Blue #42
Simultan #41
Ozark Blue #34
Southern Belle #43
O'Neal #33
Nui #32
Berkeley #5
Top Hat #45
Elizabeth #18
Elliott #19
Jubilee #24
Chandler #12
Blue Moon #8
Goldtraube #21
Bluecrop #7
Northland #31
Brigitta Blue #9
Hortblue Poppins #35
Coville #15
Legacy #25
Star #44
Cosmopolitan #14
Darrow #16
Misty #28
Earliblue #17
Biloxi #6
Liberty #26
Jersey #22
Aurora #3
Rubel #39
Centurion #11
Centra Blue #10
B
density
Min Force, N
index SI
C
H
PH
SI
Min Force, N
Cluster
genetic cluster
1
2
3
4
n.a.

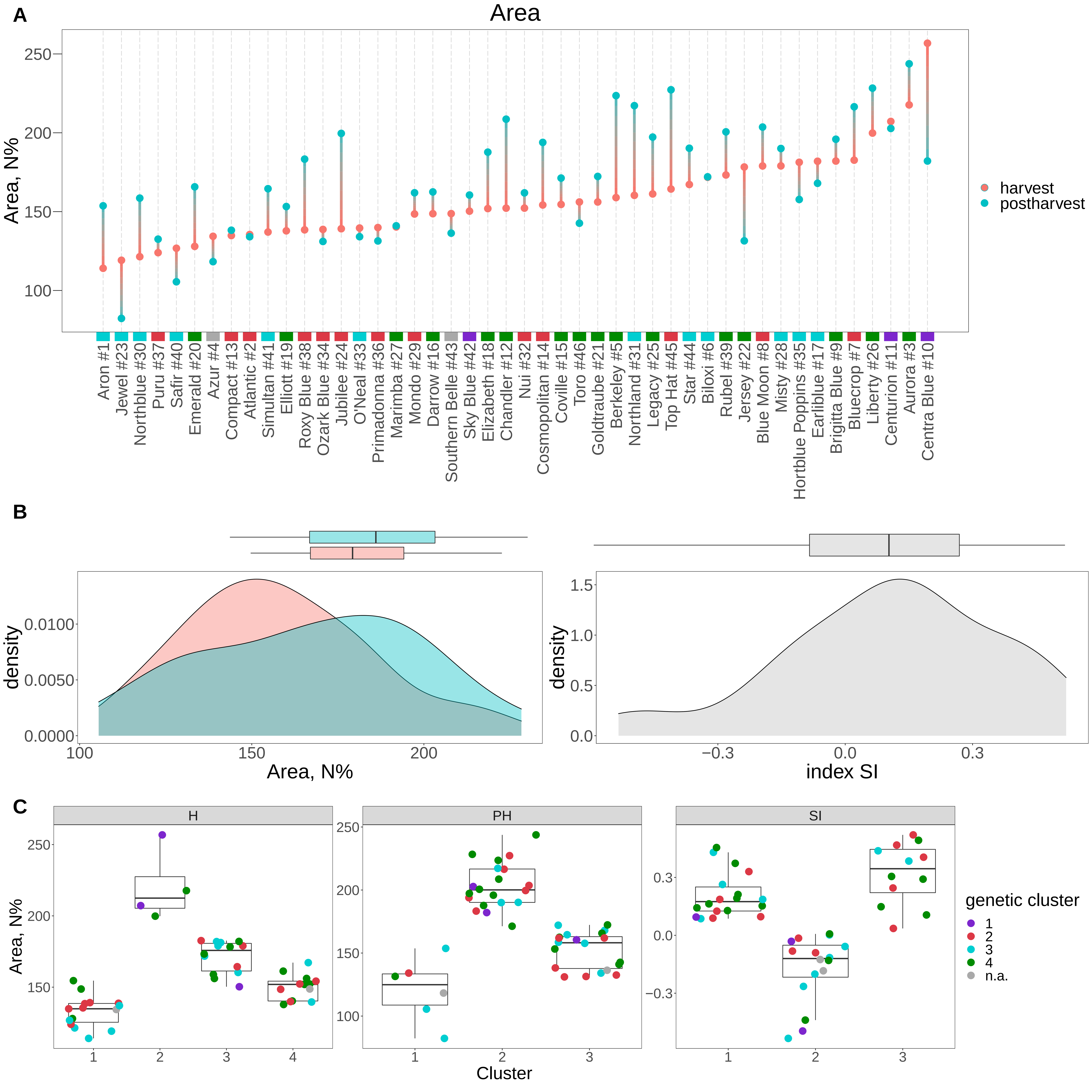

A
Area
Area, N%
250
200
150
100
harvest
postharvest
Aron #1
Jewel #23
Northblue #30
Puru #37
Safir #40
Emerald #20
Azur #4
Compact #13
Atlantic #2
Simultan #41
Elliott #19
Roxy Blue #38
Ozark Blue #34
Jubilee #24
O'Neal #33
Primadonna #36
Marimba #27
Mondo #29
Darrow #16
Southern Belle #43
Sky Blue #42
Elizabeth #18
Chandler #12
Nui #32
Cosmopolitan #14
Coville #15
Toro #46
Goldtraube #21
Berkeley #5
Northland #31
Legacy #25
Top Hat #45
Star #44
Biloxi #6
Rubel #39
Jersey #22
Blue Moon #8
Misty #28
Hortblue Poppins #35
Earliblue #17
Brigitta Blue #9
Bluecrop #7
Liberty #26
Centurion #11
Aurora #3
Centra Blue #10
B
density
0.0100
0.0050
0.0000
100
150
200
Area, N%
density
1.5
1.0
0.5
0.0
−0.3
0.0
0.3
index SI
C
H
PH
SI
Area, N%
Cluster
genetic cluster
1
2
3
4
n.a.

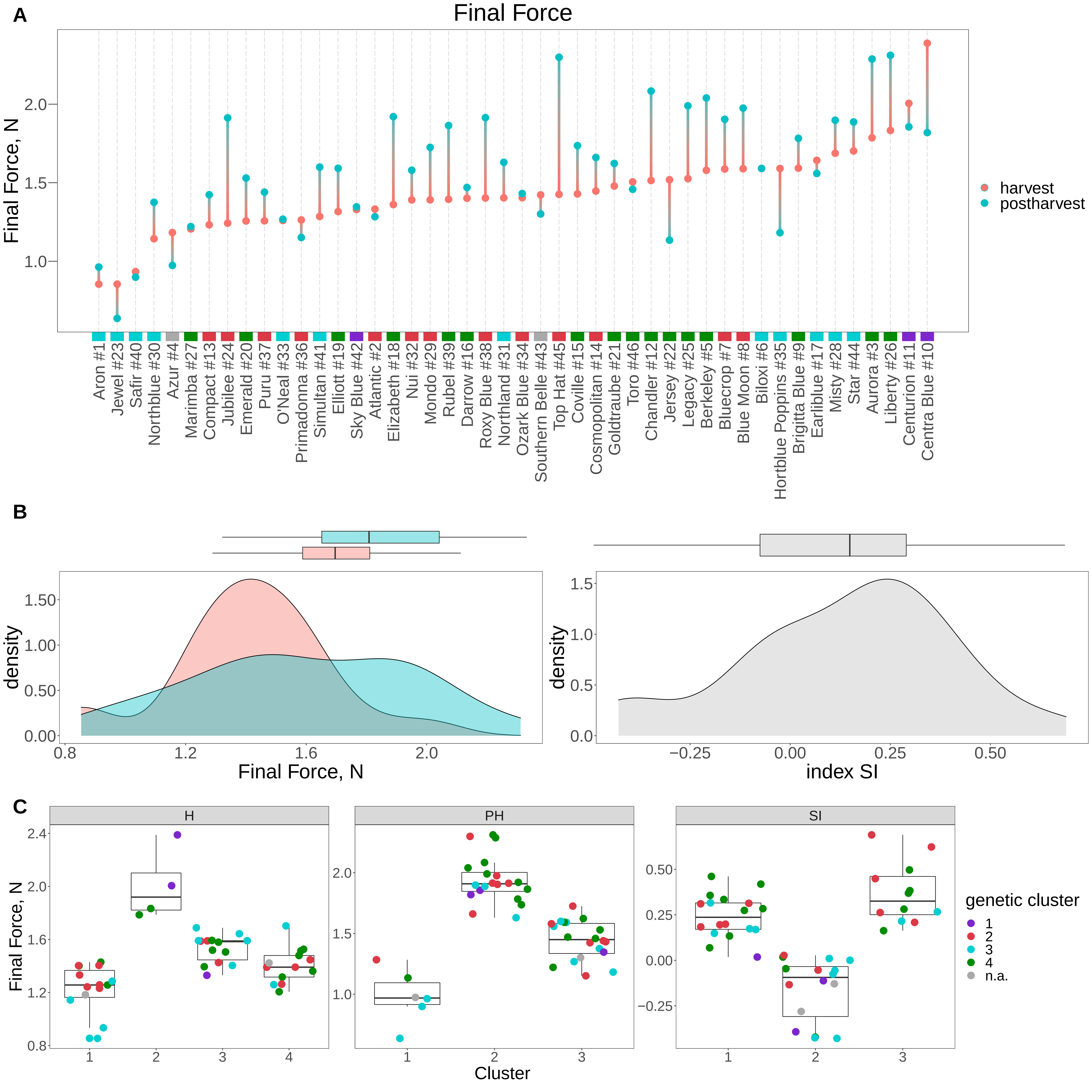
A
Final Force
Final Force, N
2.0
1.5
1.0
harvest
postharvest
Aron #1
Jewel #23
Safir #40
Northblue #30
Azur #4
Marimba #27
Compact #13
Jubilee #24
Emerald #20
Puru #37
O'Neal #33
Primadonna #36
Simultan #41
Elliott #19
Sky Blue #42
Atlantic #2
Elizabeth #18
Nui #32
Mondo #29
Rubel #39
Darrow #16
Roxy Blue #38
Northland #31
Ozark Blue #34
Southern Belle #43
Top Hat #45
Coville #15
Cosmopolitan #14
Goldtraube #21
Toro #46
Chandler #12
Jersey #22
Legacy #25
Berkeley #5
Bluecrop #7
Blue Moon #8
Biloxi #6
Hortblue Poppins #35
Brigitta Blue #9
Earliblue #17
Misty #28
Star #44
Aurora #3
Liberty #26
Centurion #11
Centra Blue #10
B
density
1.50
1.00
0.50
0.00
0.8
1.2
1.6
2.0
Final Force, N
density
1.5
1.0
0.5
0.0
−0.25
0.00
0.25
0.50
index SI
C
H
PH
SI
Final Force, N
2.4
2.0
1.6
1.2
0.8
1
2
3
4
2.0
1.5
1.0
1
2
3
0.50
0.25
0.00
−0.25
1
2
3
Cluster
genetic cluster
1
2
3
4
n.a.
