# Supplementary material for: Development of a Novel Phenotypic Roadmap to Improve Blueberry Quality and Storability
Source: Front Plant Sci. 2020 Aug 14;11:1140. doi: 10.3389/fpls.2020.01140 (PMC7456834; doi:10.3389/fpls.2020.01140)
Supplement: Supplementary file 2 [file DataSheet_2.pdf]

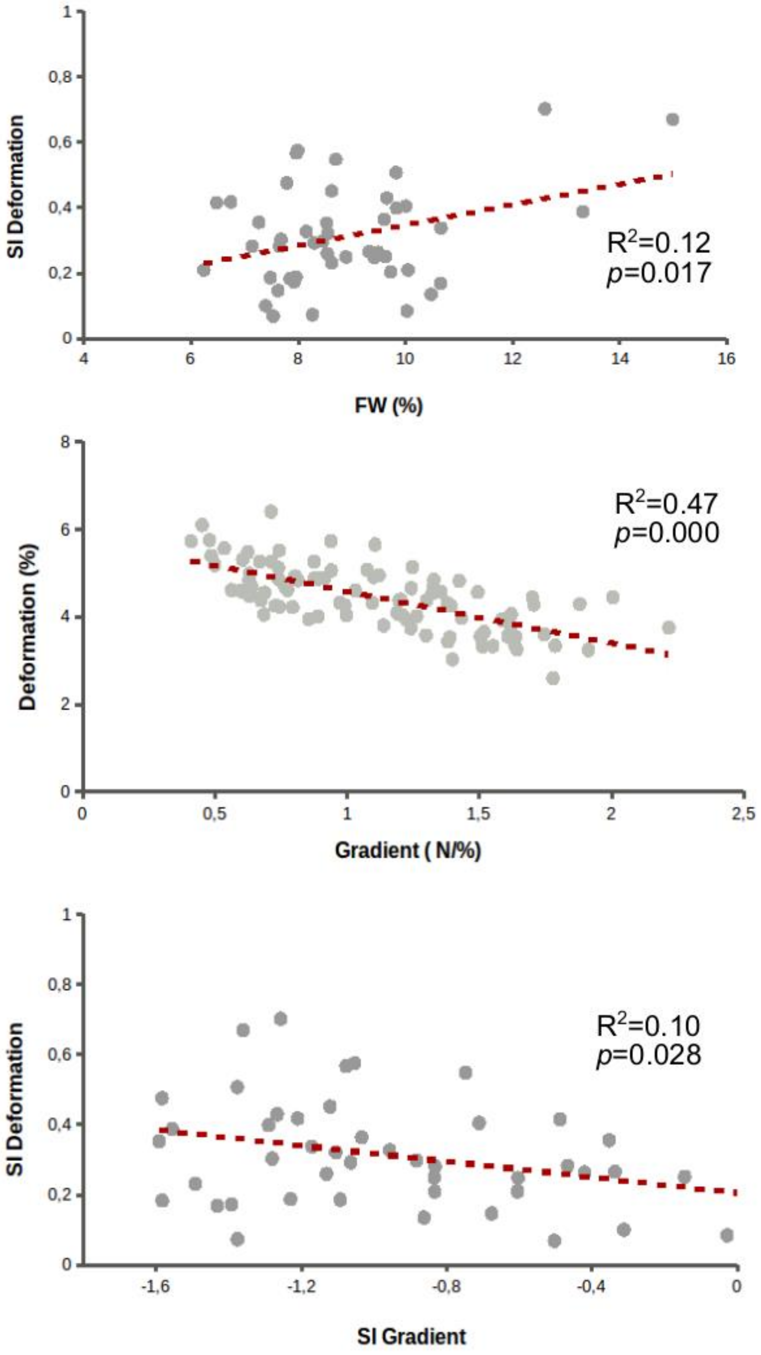

Figure S2. Correlation plots between a) storage index of deformation and percentage of fresh weight loss; b) deformation values and gradient values, both detected at harvest and storage; c) storage index of deformation and storage index of gradient
