# Supplementary material for: Development of a Novel Phenotypic Roadmap to Improve Blueberry Quality and Storability
Source: Front Plant Sci. 2020 Aug 14;11:1140. doi: 10.3389/fpls.2020.01140 (PMC7456834; doi:10.3389/fpls.2020.01140)
Supplement: Supplementary file 4 [file DataSheet_4.pdf]

Figure S4. Lollipop graphs (a), distribution plots (b) and box plots (c) of all VOC mass peaks. Clusters of box plot analysis were determined based on Ward hierarchical clustering presented in **figure 7** and **table S3**.

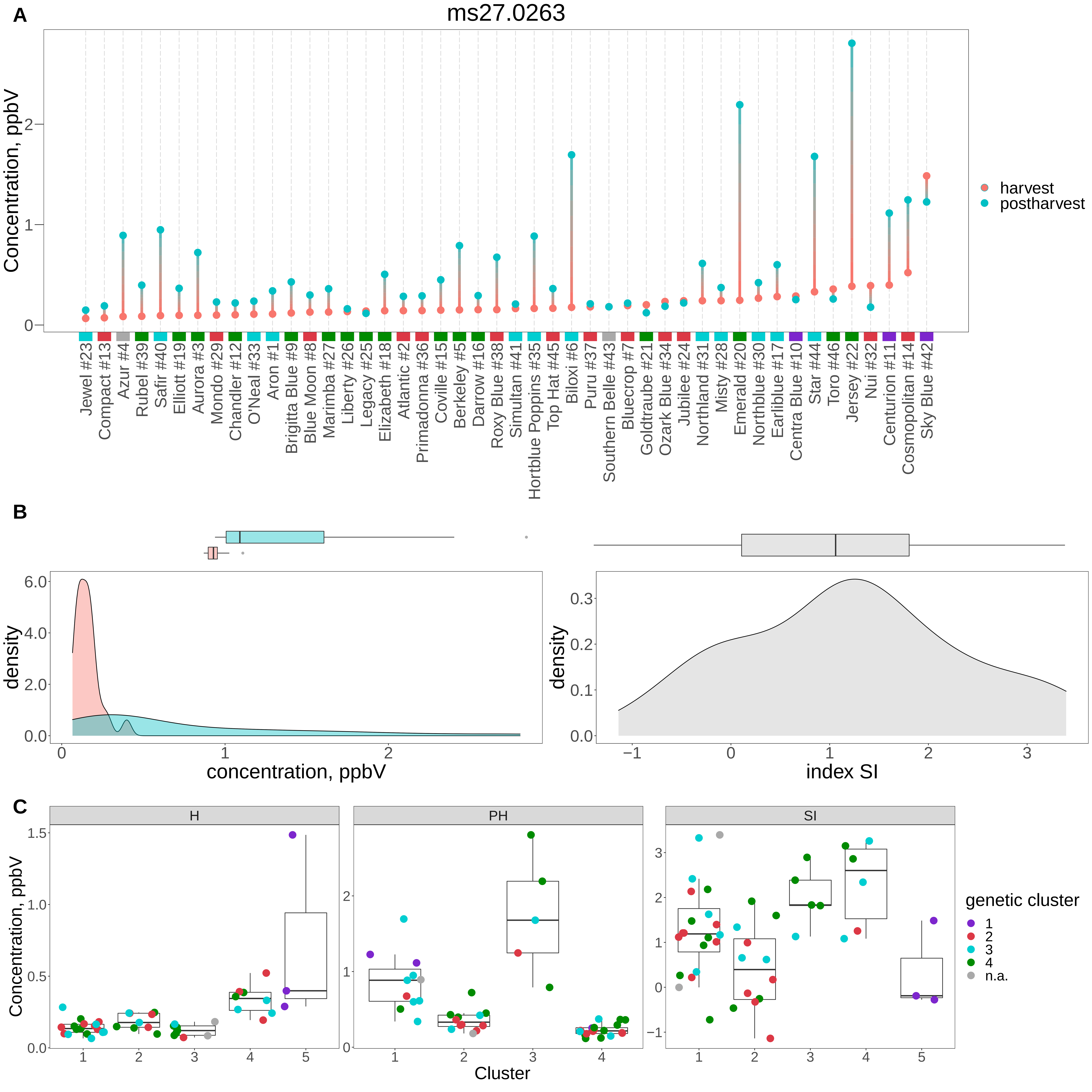

A
ms27.0263
Concentration, ppbV
harvest
postharvest
Jewel #23
Compact #13
Azur #4
Rubel #39
Safir #40
Elliott #19
Aurora #3
Mondo #29
Chandler #12
O'Neal #33
Aron #1
Brigitta Blue #9
Blue Moon #8
Marimba #27
Liberty #26
Legacy #25
Elizabeth #18
Atlantic #2
Primadonna #36
Coville #15
Berkeley #5
Darrow #16
Roxy Blue #38
Simultan #41
Hortblue Poppins #35
Top Hat #45
Biloxi #6
Puru #37
Southern Belle #43
Bluecrop #7
Goldtraube #21
Ozark Blue #34
Jubilee #24
Northland #31
Misty #28
Emerald #20
Northblue #30
Earliblue #17
Centra Blue #10
Star #44
Toro #46
Jersey #22
Nui #32
Centurion #11
Cosmopolitan #14
Sky Blue #42
B
density
concentration, ppbV
index SI
C
H
PH
SI
Concentration, ppbV
Cluster
genetic cluster
1
2
3
4
n.a.

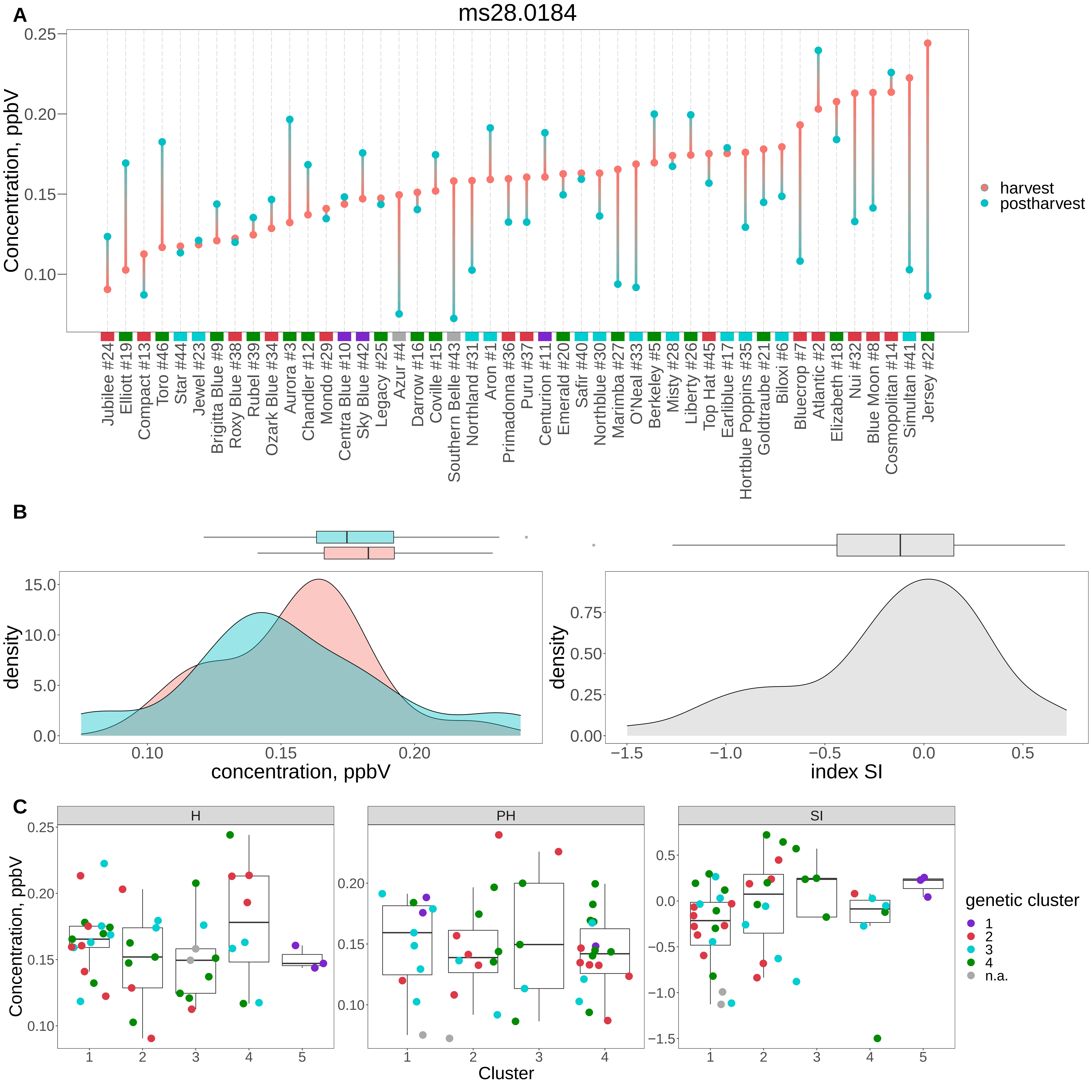

A
ms28.0184
Concentration, ppbV
harvest
postharvest
Jubilee #24
Elliott #19
Compact #13
Toro #46
Star #44
Jewel #23
Brigitta Blue #9
Roxy Blue #38
Rubel #39
Ozark Blue #34
Aurora #3
Chandler #12
Mondo #29
Centra Blue #10
Sky Blue #42
Legacy #25
Azur #4
Darrow #16
Coville #15
Southern Belle #43
Northland #31
Aron #1
Primadonna #36
Puru #37
Centurion #11
Emerald #20
Safir #40
Northblue #30
Marimba #27
O'Neal #33
Berkeley #5
Misty #28
Liberty #26
Top Hat #45
Earliblue #17
Hortblue Poppins #35
Goldtraube #21
Biloxi #6
Bluecrop #7
Atlantic #2
Elizabeth #18
Nui #32
Blue Moon #8
Cosmopolitan #14
Simultan #41
Jersey #22
B
density
concentration, ppbV
density
index SI
C
H
PH
SI
Concentration, ppbV
Cluster
genetic cluster
1
2
3
4
n.a.

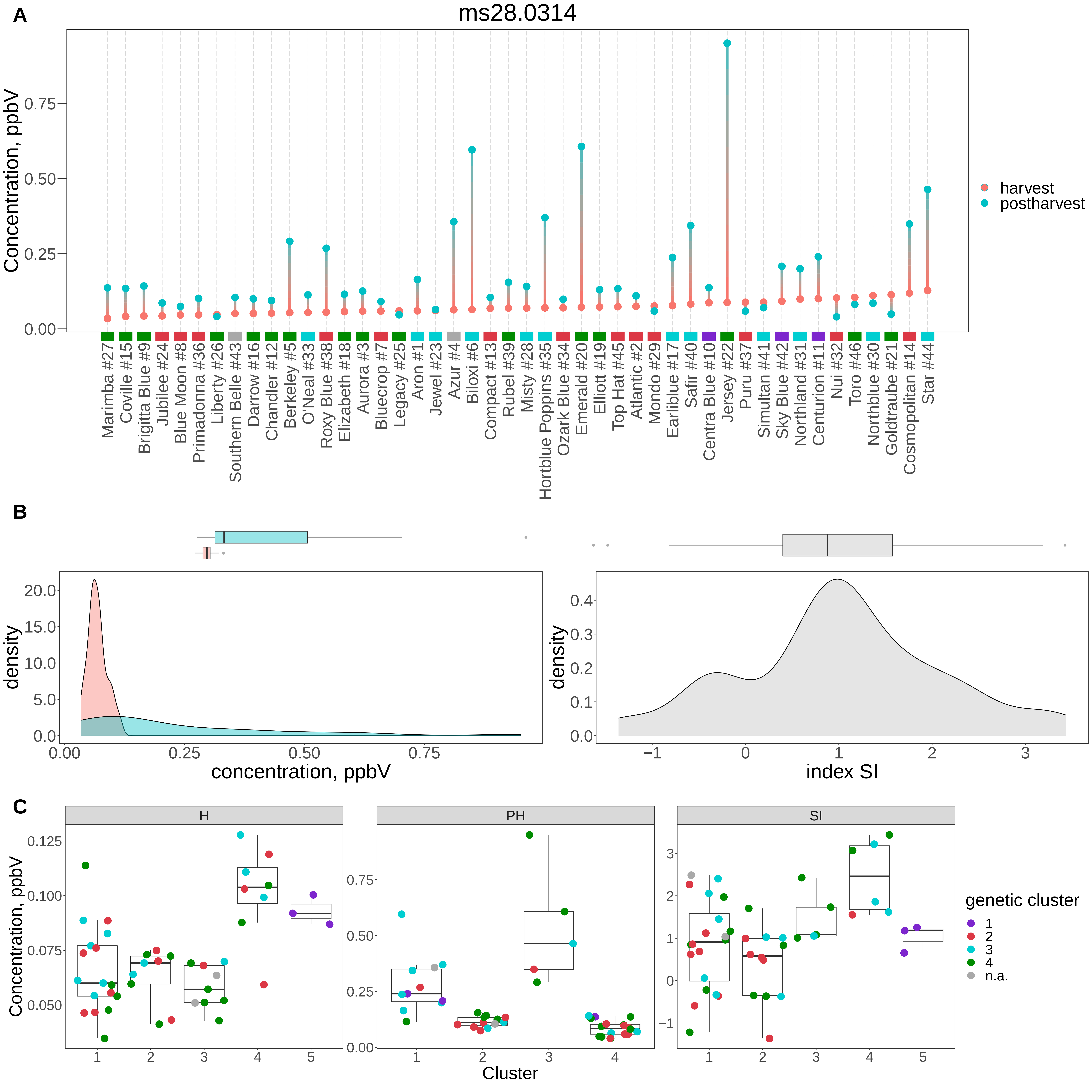

A
ms28.0314
Concentration, ppbV
harvest
postharvest
Marimba #27
Coville #15
Brigitta Blue #9
Jubilee #24
Blue Moon #8
Primadonna #36
Liberty #26
Southern Belle #43
Darrow #16
Chandler #12
Berkeley #5
O'Neal #33
Roxy Blue #38
Elizabeth #18
Aurora #3
Bluecrop #7
Legacy #25
Aron #1
Jewel #23
Azur #4
Biloxi #6
Compact #13
Rubel #39
Misty #28
Hortblue Poppins #35
Ozark Blue #34
Emerald #20
Elliott #19
Top Hat #45
Atlantic #2
Mondo #29
Earliblue #17
Safir #40
Centra Blue #10
Jersey #22
Puru #37
Simultan #41
Sky Blue #42
Northland #31
Centurion #11
Nui #32
Toro #46
Northblue #30
Goldtraube #21
Cosmopolitan #14
Star #44
B
density
concentration, ppbV
index SI
C
H
PH
SI
Concentration, ppbV
Cluster
genetic cluster
1
2
3
4
n.a.

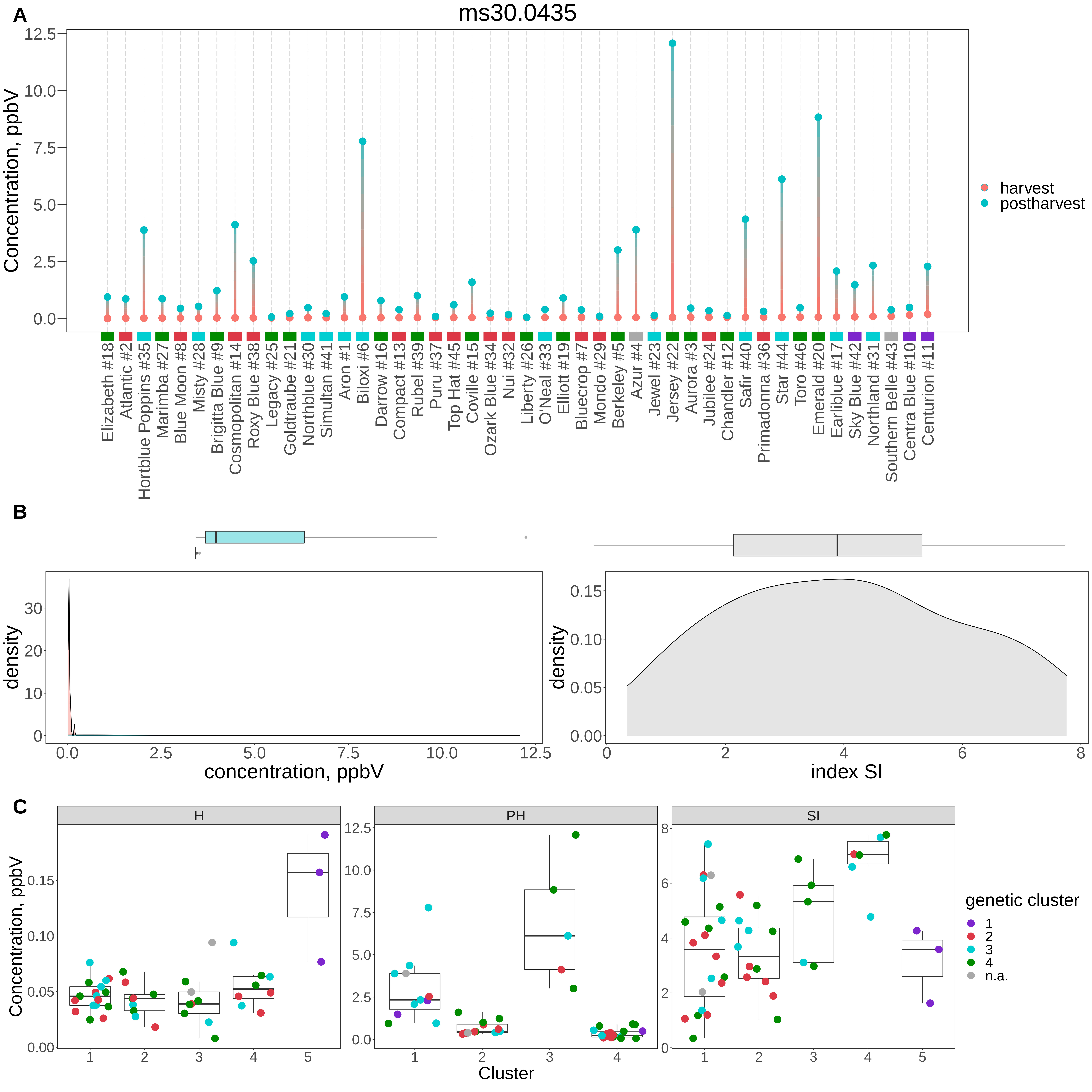
A
ms30.0435
Concentration, ppbV
harvest
postharvest
Elizabeth #18
Atlantic #2
Hortblue Poppins #35
Marimba #27
Blue Moon #8
Misty #28
Brigitta Blue #9
Cosmopolitan #14
Roxy Blue #38
Legacy #25
Goldtraube #21
Northblue #30
Simultan #41
Aron #1
Biloxi #6
Darrow #16
Compact #13
Rubel #39
Puru #37
Top Hat #45
Coville #15
Ozark Blue #34
Nui #32
Liberty #26
O'Neal #33
Elliott #19
Bluecrop #7
Mondo #29
Berkeley #5
Azur #4
Jewel #23
Jersey #22
Aurora #3
Jubilee #24
Chandler #12
Safir #40
Primadonna #36
Star #44
Toro #46
Emerald #20
Earliblue #17
Sky Blue #42
Northland #31
Southern Belle #43
Centra Blue #10
Centurion #11
B
density
concentration, ppbV
index SI
C
H
PH
SI
Concentration, ppbV
Cluster
genetic cluster
1
2
3
4
n.a.

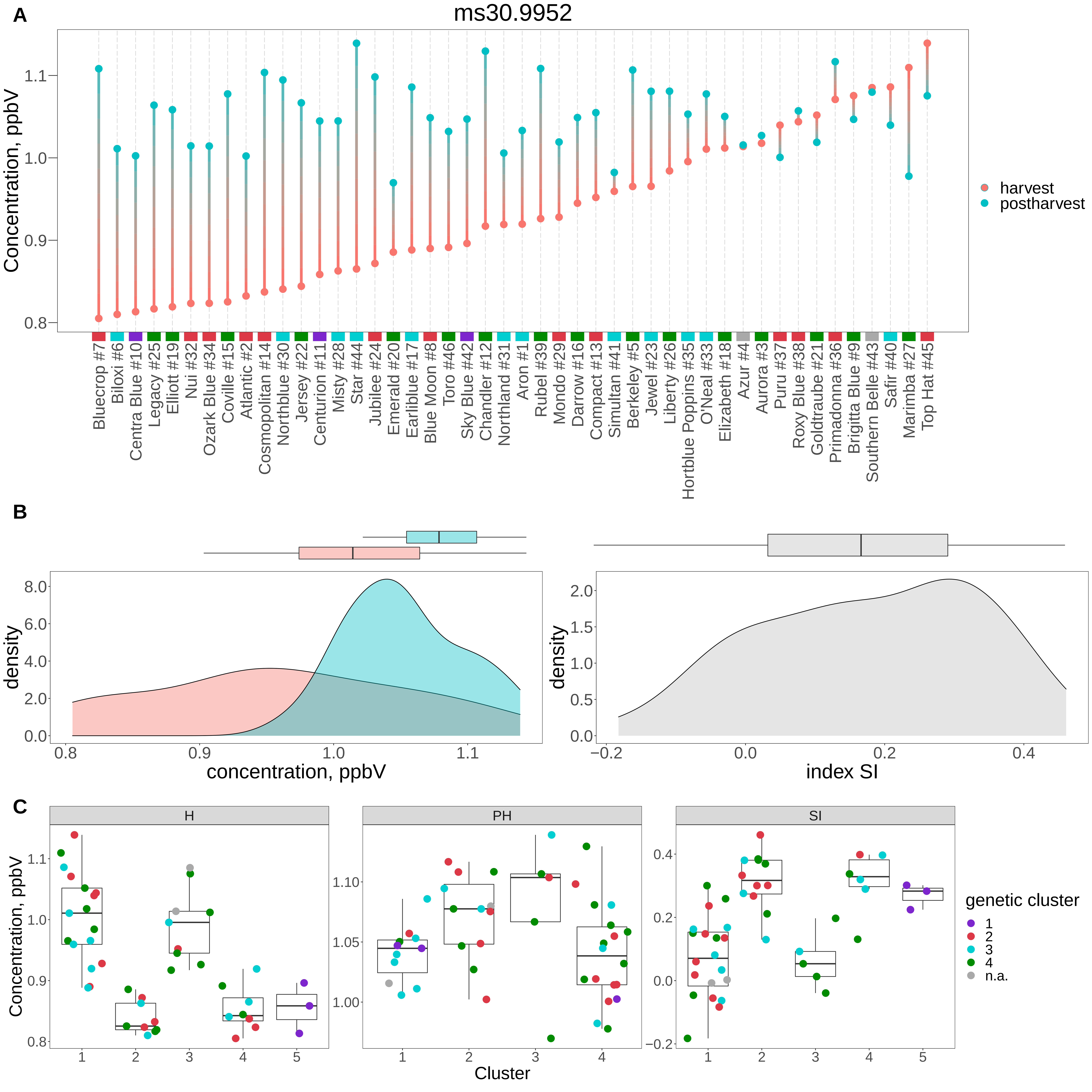

A
ms30.9952
Concentration, ppbV
harvest
postharvest
Bluecrop #7
Biloxi #6
Centra Blue #10
Legacy #25
Elliott #19
Nui #32
Ozark Blue #34
Coville #15
Atlantic #2
Cosmopolitan #14
Northblue #30
Jersey #22
Centurion #11
Misty #28
Star #44
Jubilee #24
Emerald #20
Earliblue #17
Blue Moon #8
Toro #46
Sky Blue #42
Chandler #12
Northland #31
Aron #1
Rubel #39
Mondo #29
Darrow #16
Compact #13
Simultan #41
Berkeley #5
Jewel #23
Liberty #26
Hortblue Poppins #35
O'Neal #33
Elizabeth #18
Azur #4
Aurora #3
Puru #37
Roxy Blue #38
Goldtraube #21
Primadonna #36
Brigitta Blue #9
Southern Belle #43
Safir #40
Marimba #27
Top Hat #45
B
density
concentration, ppbV
density
index SI
C
H
PH
SI
Concentration, ppbV
Cluster
genetic cluster
1
2
3
4
n.a.

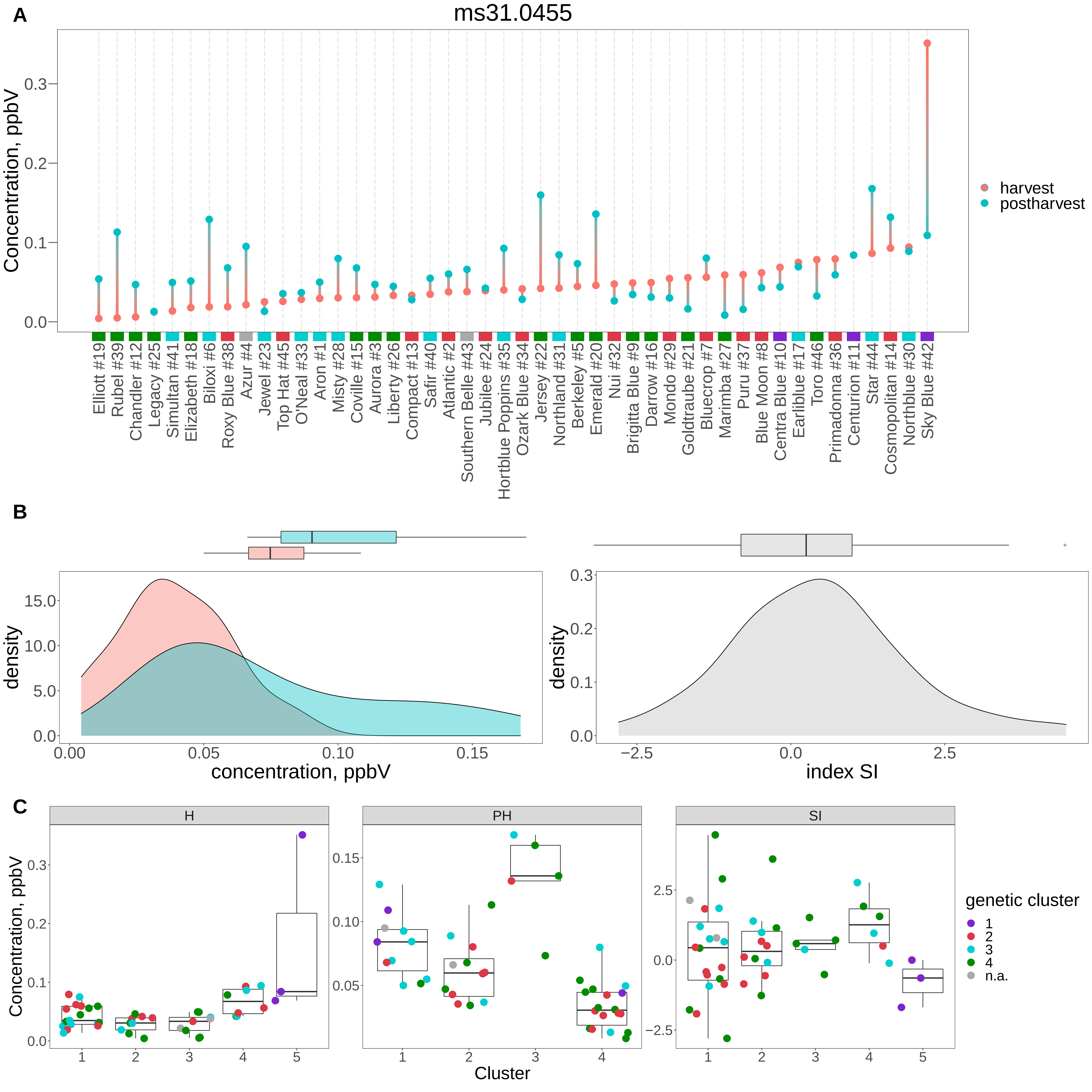
A
ms31.0455
Concentration, ppbV
harvest
postharvest
Elliott #19
Rubel #39
Chandler #12
Legacy #25
Simultan #41
Elizabeth #18
Biloxi #6
Roxy Blue #38
Azur #4
Jewel #23
Top Hat #45
O'Neal #33
Aron #1
Misty #28
Coville #15
Aurora #3
Liberty #26
Compact #13
Safir #40
Atlantic #2
Southern Belle #43
Jubilee #24
Hortblue Poppins #35
Ozark Blue #34
Jersey #22
Northland #31
Berkeley #5
Emerald #20
Nui #32
Brigitta Blue #9
Darrow #16
Mondo #29
Goldtraube #21
Bluecrop #7
Marimba #27
Puru #37
Blue Moon #8
Centra Blue #10
Earliblue #17
Toro #46
Primadonna #36
Centurion #11
Star #44
Cosmopolitan #14
Northblue #30
Sky Blue #42
B
density
concentration, ppbV
index SI
C
H
PH
SI
Concentration, ppbV
Cluster
genetic cluster
1
2
3
4
n.a.

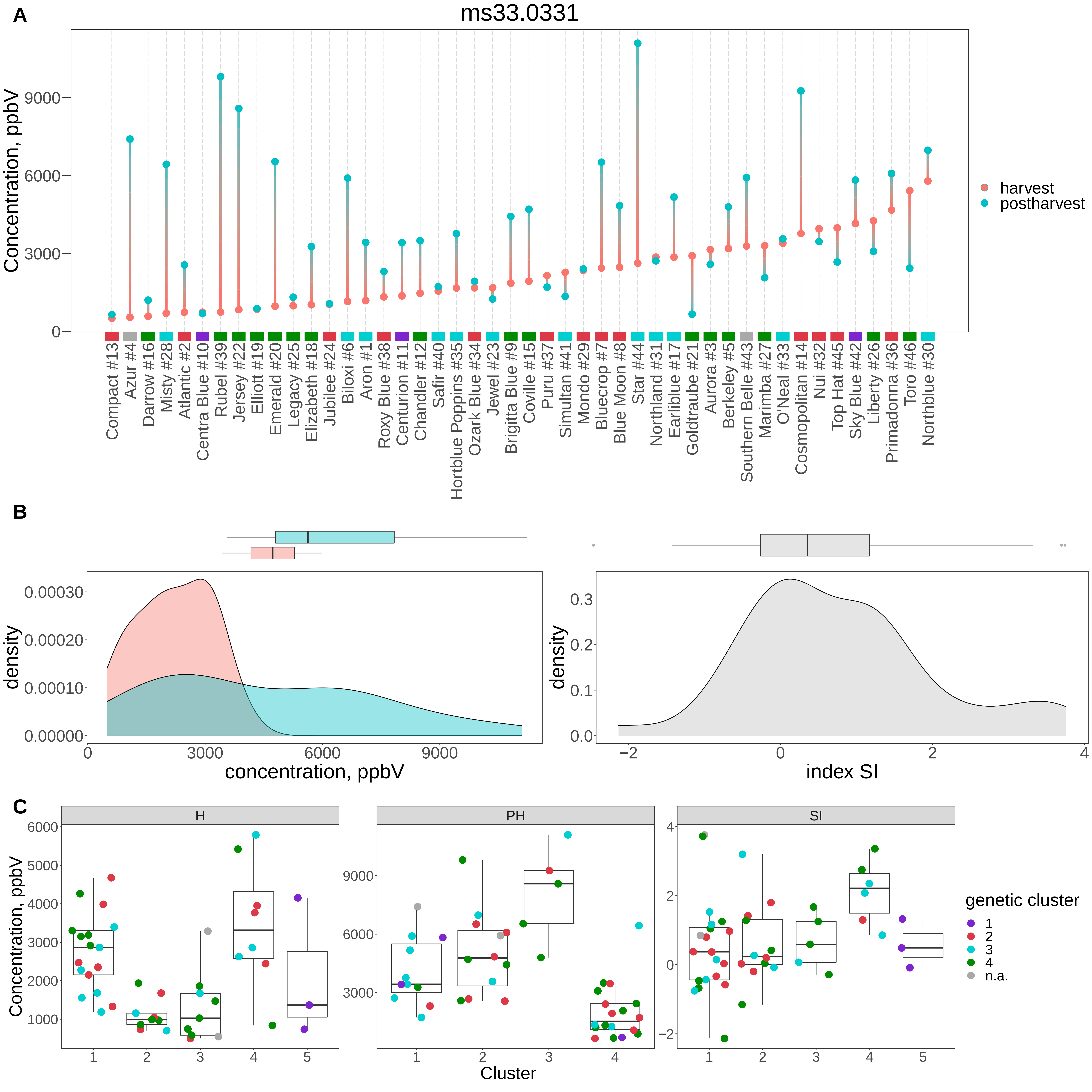
A
ms33.0331
Concentration, ppbV
harvest
postharvest
Compact #13
Azur #4
Darrow #16
Misty #28
Atlantic #2
Centra Blue #10
Rubel #39
Jersey #22
Elliott #19
Emerald #20
Legacy #25
Elizabeth #18
Jubilee #24
Biloxi #6
Aron #1
Roxy Blue #38
Centurion #11
Chandler #12
Safir #40
Hortblue Poppins #35
Ozark Blue #34
Jewel #23
Brigitta Blue #9
Coville #15
Puru #37
Simultan #41
Mondo #29
Bluecrop #7
Blue Moon #8
Star #44
Northland #31
Earliblue #17
Goldtraube #21
Aurora #3
Berkeley #5
Southern Belle #43
Marimba #27
O'Neal #33
Cosmopolitan #14
Nui #32
Top Hat #45
Sky Blue #42
Liberty #26
Primadonna #36
Toro #46
Northblue #30
B
density
concentration, ppbV
index SI
C
H
PH
SI
Concentration, ppbV
Cluster
genetic cluster
1
2
3
4
n.a.

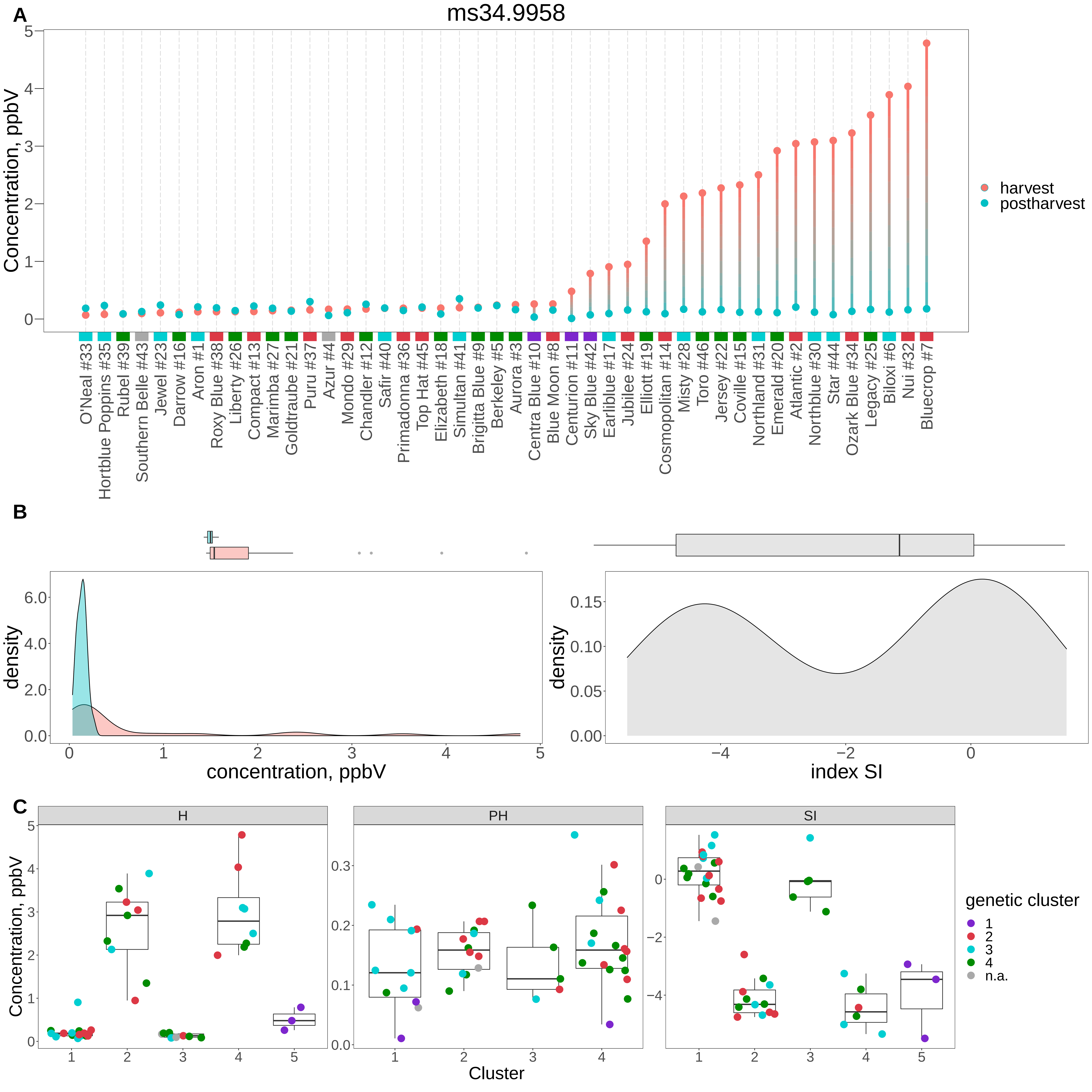
A
ms34.9958
Concentration, ppbV
harvest
postharvest
O'Neal #33
Hortblue Poppins #35
Rubel #39
Southern Belle #43
Jewel #23
Darrow #16
Aron #1
Roxy Blue #38
Liberty #26
Compact #13
Marimba #27
Goldtraube #21
Puru #37
Azur #4
Mondo #29
Chandler #12
Safir #40
Primadonna #36
Top Hat #45
Elizabeth #18
Simultan #41
Brigitta Blue #9
Berkeley #5
Aurora #3
Centra Blue #10
Blue Moon #8
Centurion #11
Sky Blue #42
Earliblue #17
Jubilee #24
Elliott #19
Cosmopolitan #14
Misty #28
Toro #46
Jersey #22
Coville #15
Northland #31
Emerald #20
Atlantic #2
Northblue #30
Star #44
Ozark Blue #34
Legacy #25
Biloxi #6
Nui #32
Bluecrop #7
B
density
concentration, ppbV
density
index SI
C
H
PH
SI
Concentration, ppbV
Cluster
genetic cluster
1
2
3
4
n.a.

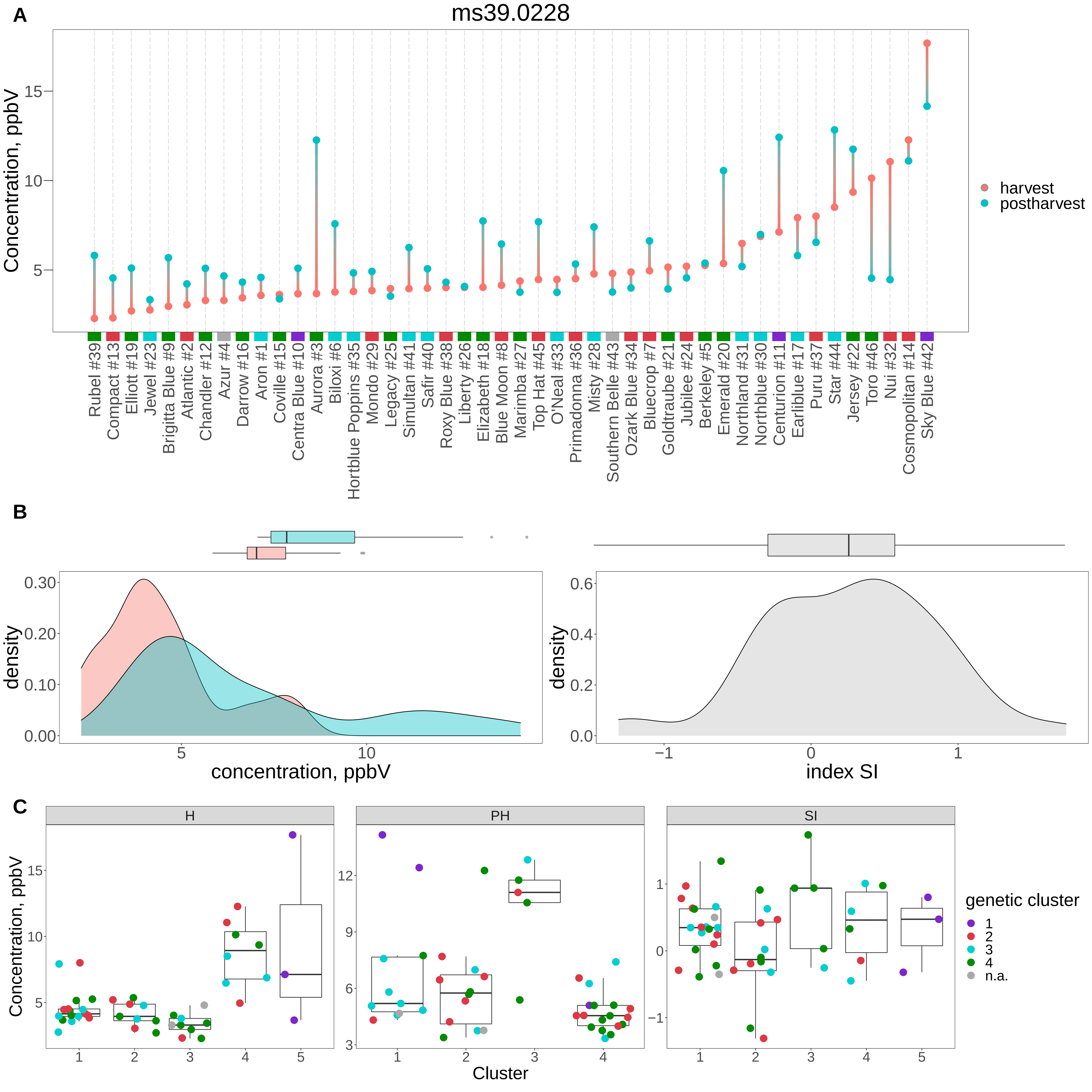
A
ms39.0228
Concentration, ppbV
harvest
postharvest
Rubel #39
Compact #13
Elliott #19
Jewel #23
Brigitta Blue #9
Atlantic #2
Chandler #12
Azur #4
Darrow #16
Aron #1
Coville #15
Centra Blue #10
Aurora #3
Biloxi #6
Hortblue Poppins #35
Mondo #29
Legacy #25
Simultan #41
Safir #40
Roxy Blue #38
Liberty #26
Elizabeth #18
Blue Moon #8
Marimba #27
Top Hat #45
O'Neal #33
Primadonna #36
Misty #28
Southern Belle #43
Ozark Blue #34
Bluecrop #7
Goldtraube #21
Jubilee #24
Berkeley #5
Emerald #20
Northland #31
Northblue #30
Centurion #11
Earliblue #17
Puru #37
Star #44
Jersey #22
Toro #46
Nui #32
Cosmopolitan #14
Sky Blue #42
B
density
concentration, ppbV
index SI
C
H
PH
SI
Concentration, ppbV
Cluster
genetic cluster
1
2
3
4
n.a.

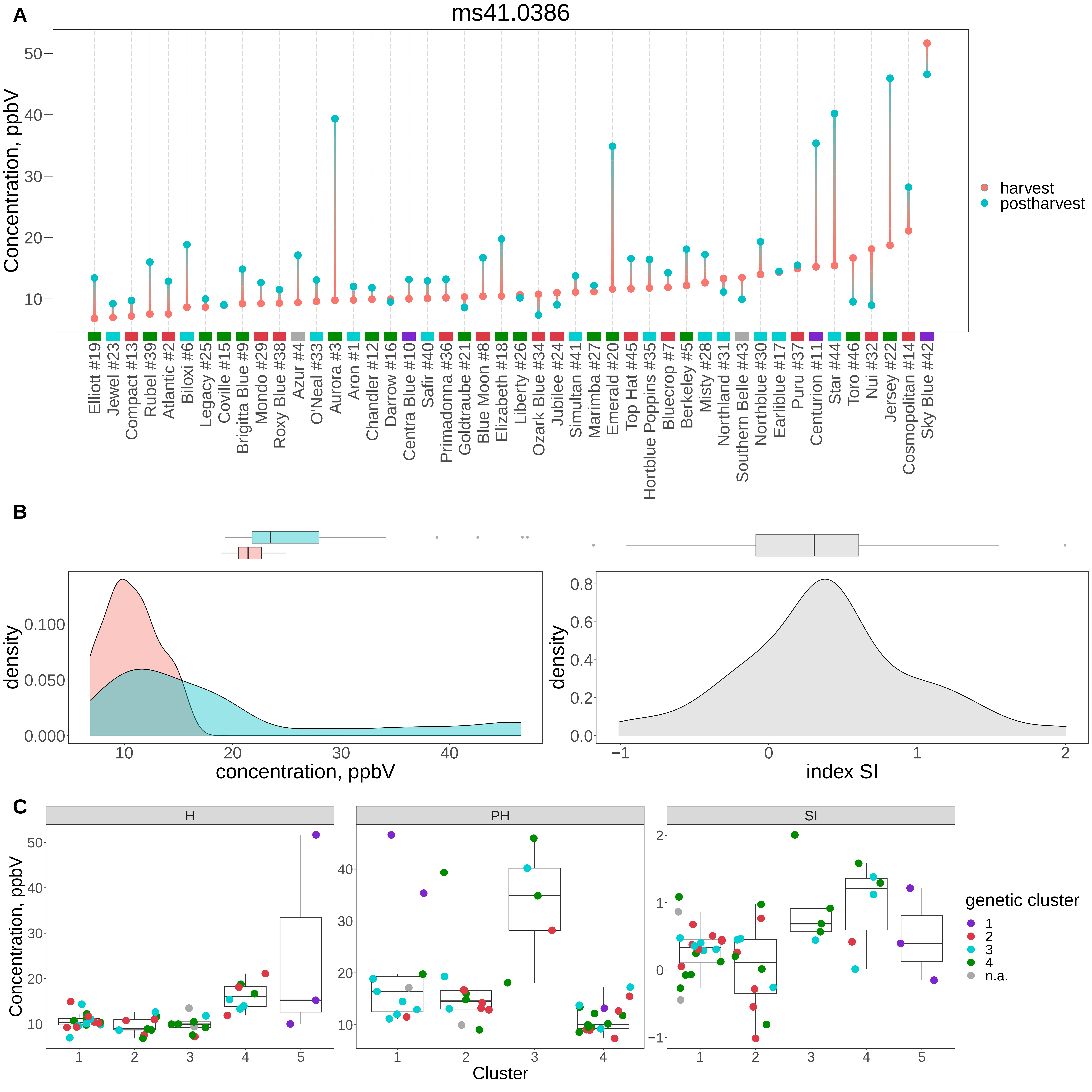

A
ms41.0386
Concentration, ppbV
harvest
postharvest
Elliott #19
Jewel #23
Compact #13
Rubel #39
Atlantic #2
Biloxi #6
Legacy #25
Coville #15
Brigitta Blue #9
Mondo #29
Roxy Blue #38
Azur #4
O'Neal #33
Aurora #3
Aron #1
Chandler #12
Darrow #16
Centra Blue #10
Safir #40
Primadonna #36
Goldtraube #21
Blue Moon #8
Elizabeth #18
Liberty #26
Ozark Blue #34
Jubilee #24
Simultan #41
Marimba #27
Emerald #20
Top Hat #45
Hortblue Poppins #35
Bluecrop #7
Berkeley #5
Misty #28
Northland #31
Southern Belle #43
Northblue #30
Earliblue #17
Puru #37
Centurion #11
Star #44
Toro #46
Nui #32
Jersey #22
Cosmopolitan #14
Sky Blue #42
B
density
concentration, ppbV
density
index SI
C
H
PH
SI
Concentration, ppbV
Cluster
genetic cluster
1
2
3
4
n.a.

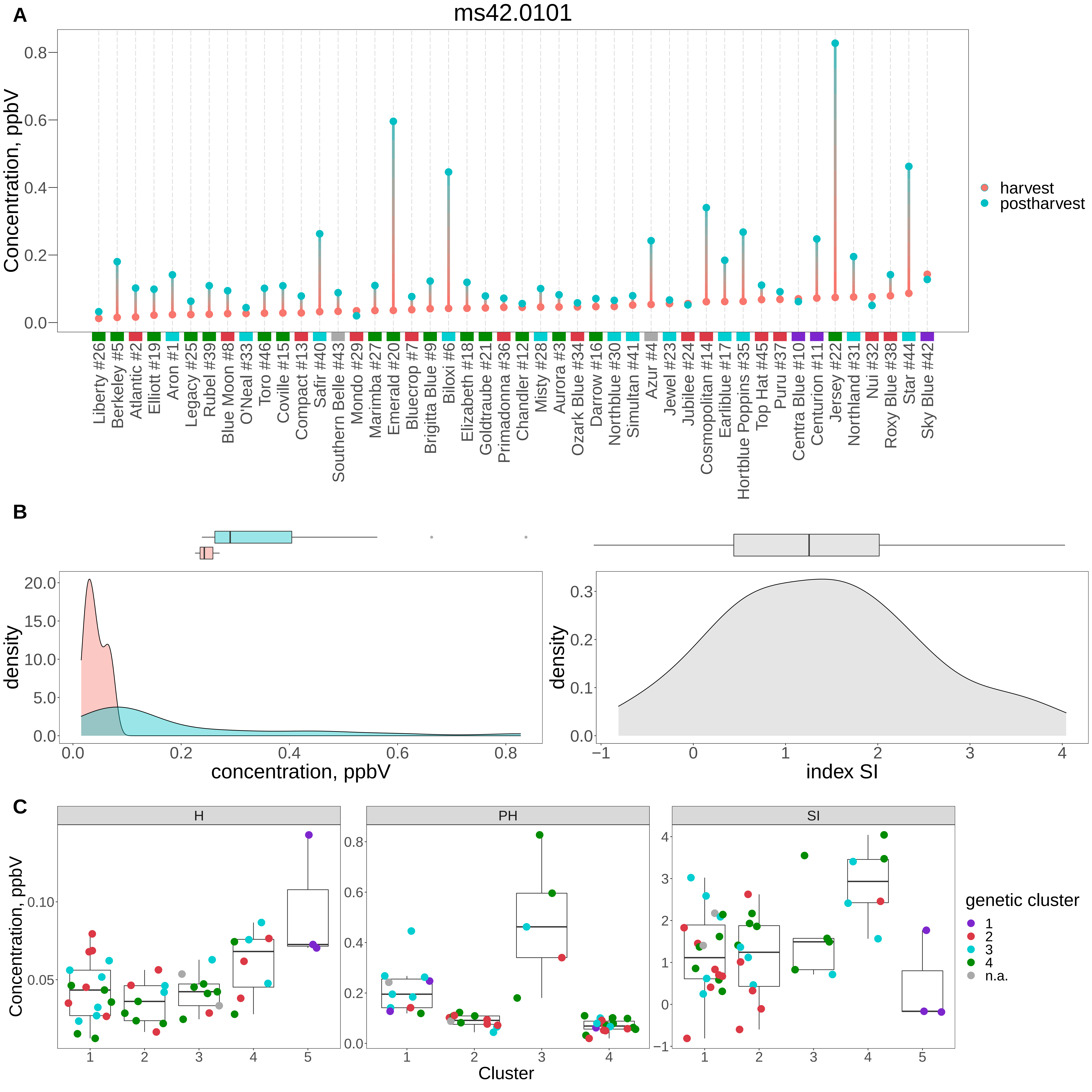
A
ms42.0101
Concentration, ppbV
harvest
postharvest
Liberty #26
Berkeley #5
Atlantic #2
Elliott #19
Aron #1
Legacy #25
Rubel #39
Blue Moon #8
O'Neal #33
Toro #46
Coville #15
Compact #13
Safir #40
Southern Belle #43
Mondo #29
Marimba #27
Emerald #20
Bluecrop #7
Brigitta Blue #9
Biloxi #6
Elizabeth #18
Goldtraube #21
Primadonna #36
Chandler #12
Misty #28
Aurora #3
Ozark Blue #34
Darrow #16
Northblue #30
Simultan #41
Azur #4
Jewel #23
Jubilee #24
Cosmopolitan #14
Earliblue #17
Hortblue Poppins #35
Top Hat #45
Puru #37
Centra Blue #10
Centurion #11
Jersey #22
Northland #31
Nui #32
Roxy Blue #38
Star #44
Sky Blue #42
B
density
concentration, ppbV
index SI
C
H
PH
SI
Concentration, ppbV
Cluster
genetic cluster
1
2
3
4
n.a.

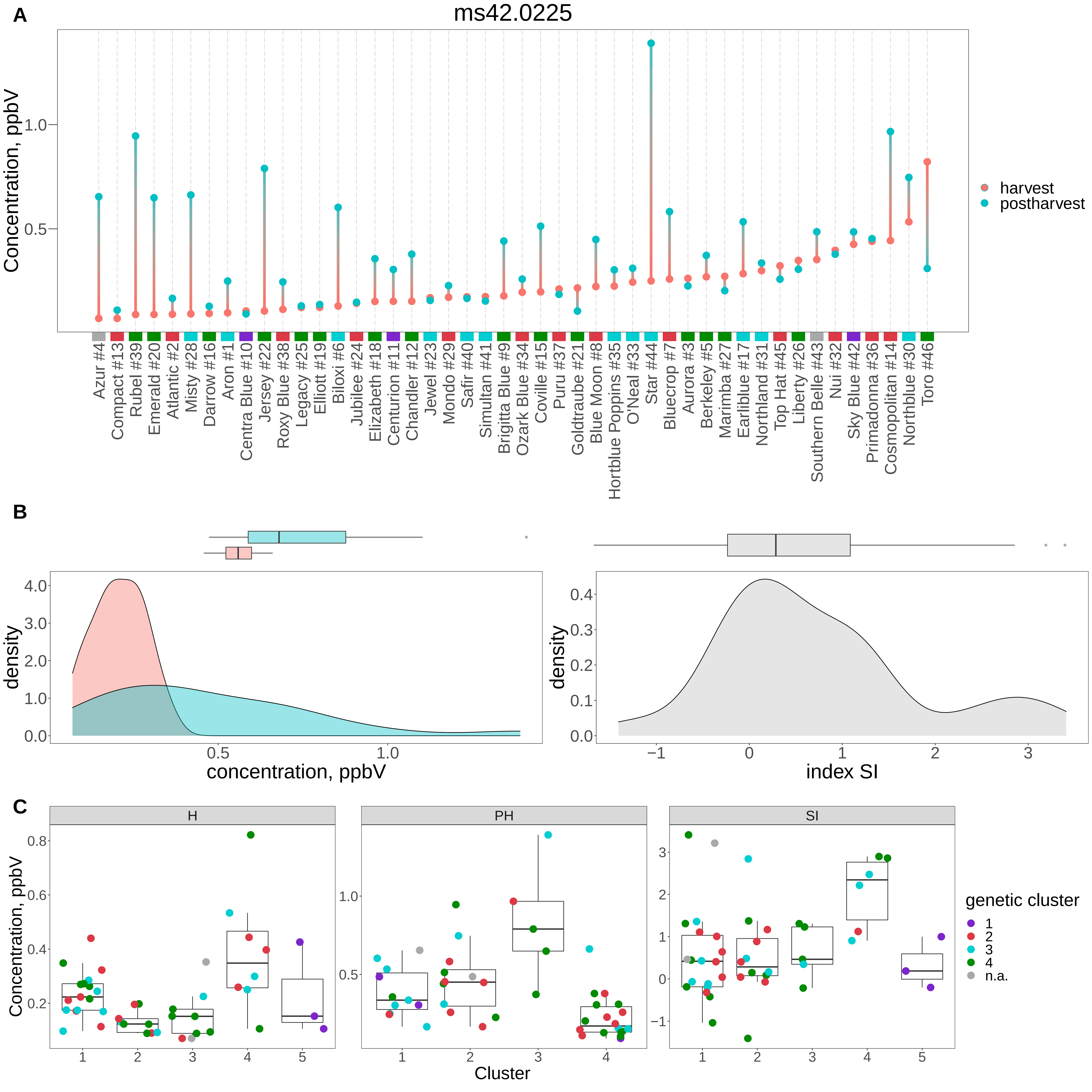

A
ms42.0225
Concentration, ppbV
harvest
postharvest
Azur #4
Compact #13
Rubel #39
Emerald #20
Atlantic #2
Misty #28
Darrow #16
Aron #1
Centra Blue #10
Jersey #22
Roxy Blue #38
Legacy #25
Elliott #19
Biloxi #6
Jubilee #24
Elizabeth #18
Centurion #11
Chandler #12
Jewel #23
Mondo #29
Safir #40
Simultan #41
Brigitta Blue #9
Ozark Blue #34
Coville #15
Puru #37
Goldtraube #21
Blue Moon #8
Hortblue Poppins #35
O'Neal #33
Star #44
Bluecrop #7
Aurora #3
Berkeley #5
Marimba #27
Earliblue #17
Northland #31
Top Hat #45
Liberty #26
Southern Belle #43
Nui #32
Sky Blue #42
Primadonna #36
Cosmopolitan #14
Northblue #30
Toro #46
B
density
concentration, ppbV
index SI
C
H
PH
SI
Cluster
genetic cluster
n.a.

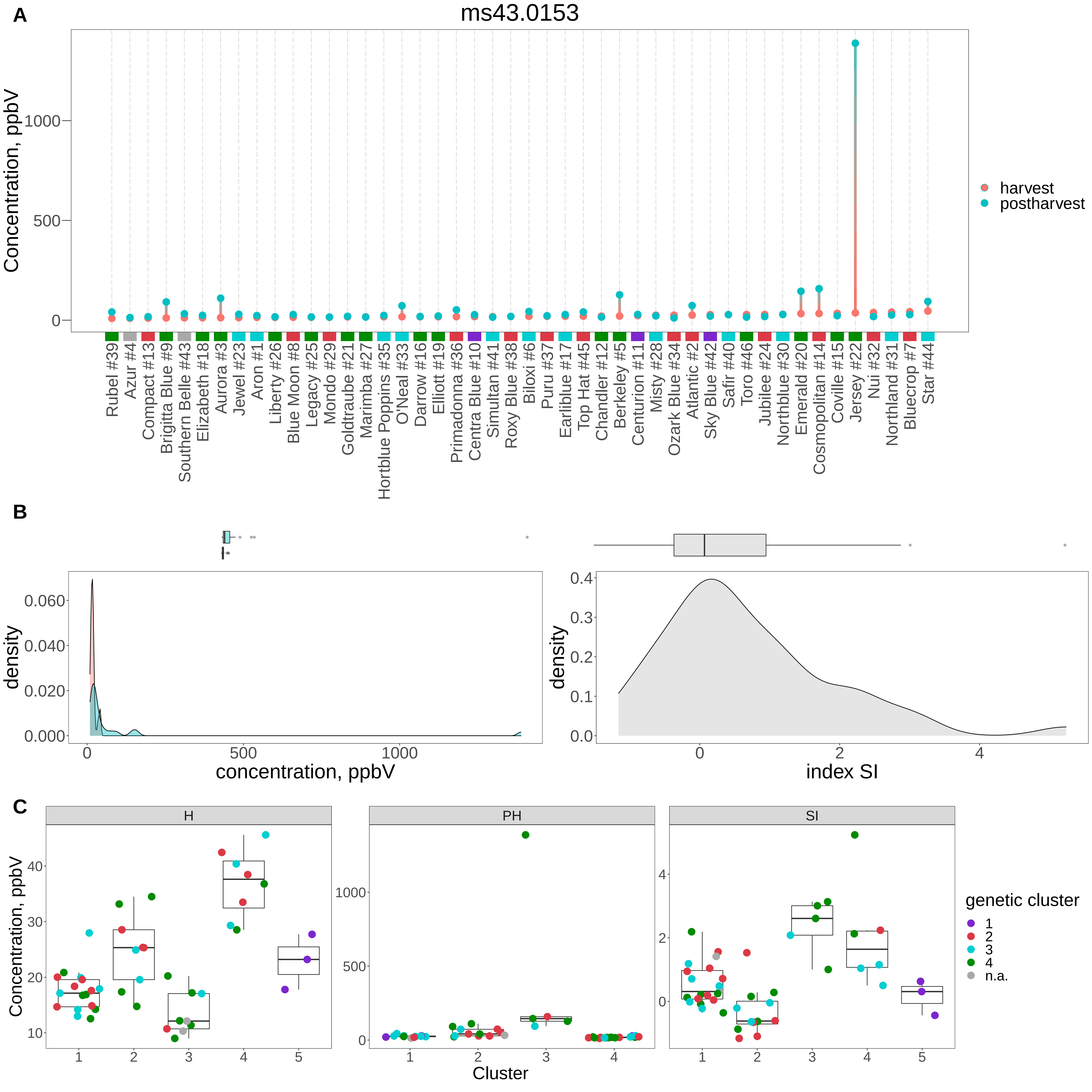
A
ms43.0153
Concentration, ppbV
harvest
postharvest
Rubel #39
Azur #4
Compact #13
Brigitta Blue #9
Southern Belle #43
Elizabeth #18
Aurora #3
Jewel #23
Aron #1
Liberty #26
Blue Moon #8
Legacy #25
Mondo #29
Goldtraube #21
Marimba #27
Hortblue Poppins #35
O'Neal #33
Darrow #16
Elliott #19
Primadonna #36
Centra Blue #10
Simultan #41
Roxy Blue #38
Biloxi #6
Puru #37
Earliblue #17
Top Hat #45
Chandler #12
Berkeley #5
Centurion #11
Misty #28
Ozark Blue #34
Atlantic #2
Sky Blue #42
Safir #40
Toro #46
Jubilee #24
Northblue #30
Emerald #20
Cosmopolitan #14
Coville #15
Jersey #22
Nui #32
Northland #31
Bluecrop #7
Star #44
B
density
concentration, ppbV
index SI
C
H
PH
SI
Concentration, ppbV
Cluster
genetic cluster
n.a.

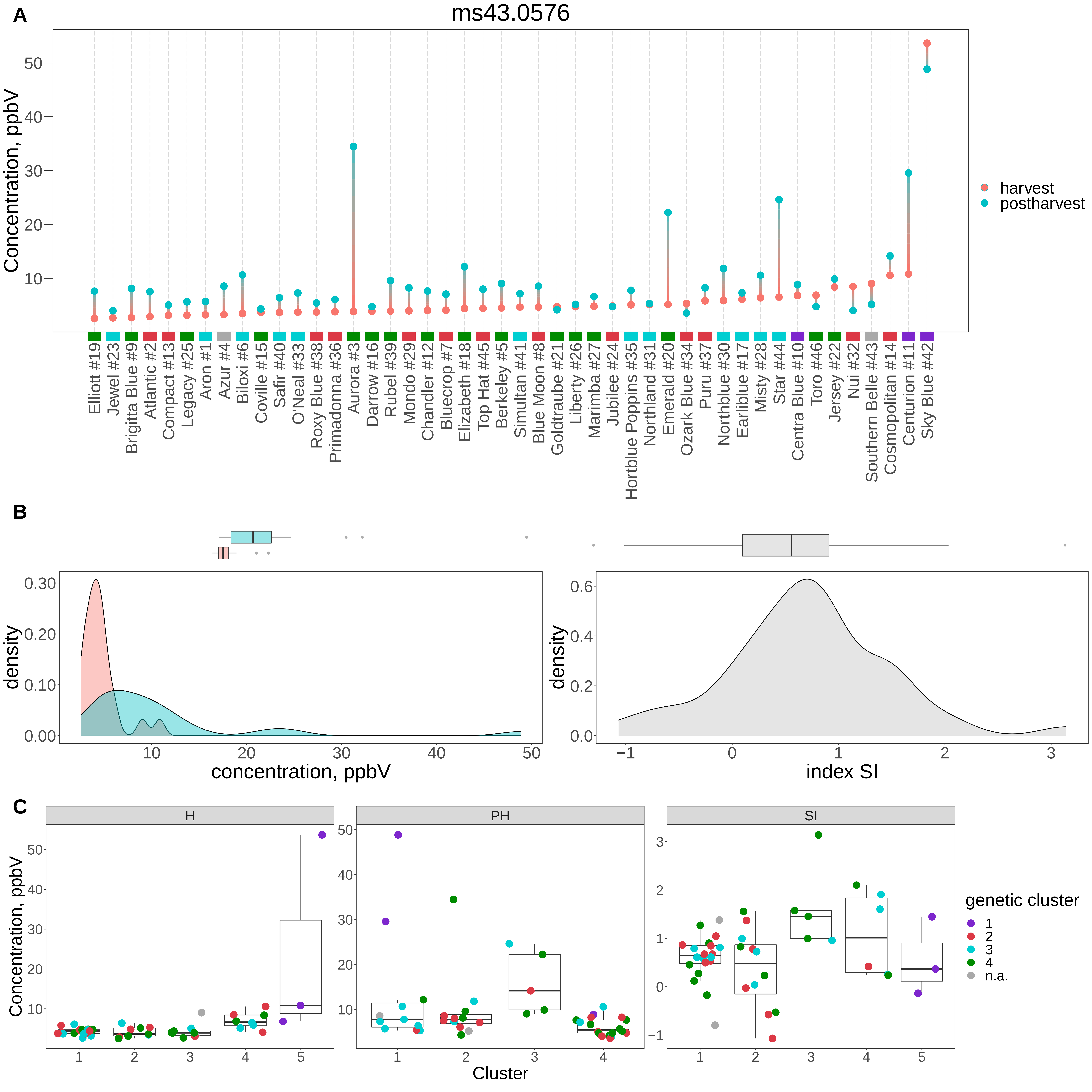

A
ms43.0576
Concentration, ppbV
harvest
postharvest
Elliott #19
Jewel #23
Brigitta Blue #9
Atlantic #2
Compact #13
Legacy #25
Aron #1
Azur #4
Biloxi #6
Coville #15
Safir #40
O'Neal #33
Roxy Blue #38
Primadonna #36
Aurora #3
Darrow #16
Rubel #39
Mondo #29
Chandler #12
Bluecrop #7
Elizabeth #18
Top Hat #45
Berkeley #5
Simultan #41
Blue Moon #8
Goldtraube #21
Liberty #26
Marimba #27
Jubilee #24
Hortblue Poppins #35
Northland #31
Emerald #20
Ozark Blue #34
Puru #37
Northblue #30
Earliblue #17
Misty #28
Star #44
Centra Blue #10
Toro #46
Jersey #22
Nui #32
Southern Belle #43
Cosmopolitan #14
Centurion #11
Sky Blue #42
B
density
concentration, ppbV
density
index SI
C
H
PH
SI
Concentration, ppbV
Cluster
genetic cluster
1
2
3
4
n.a.

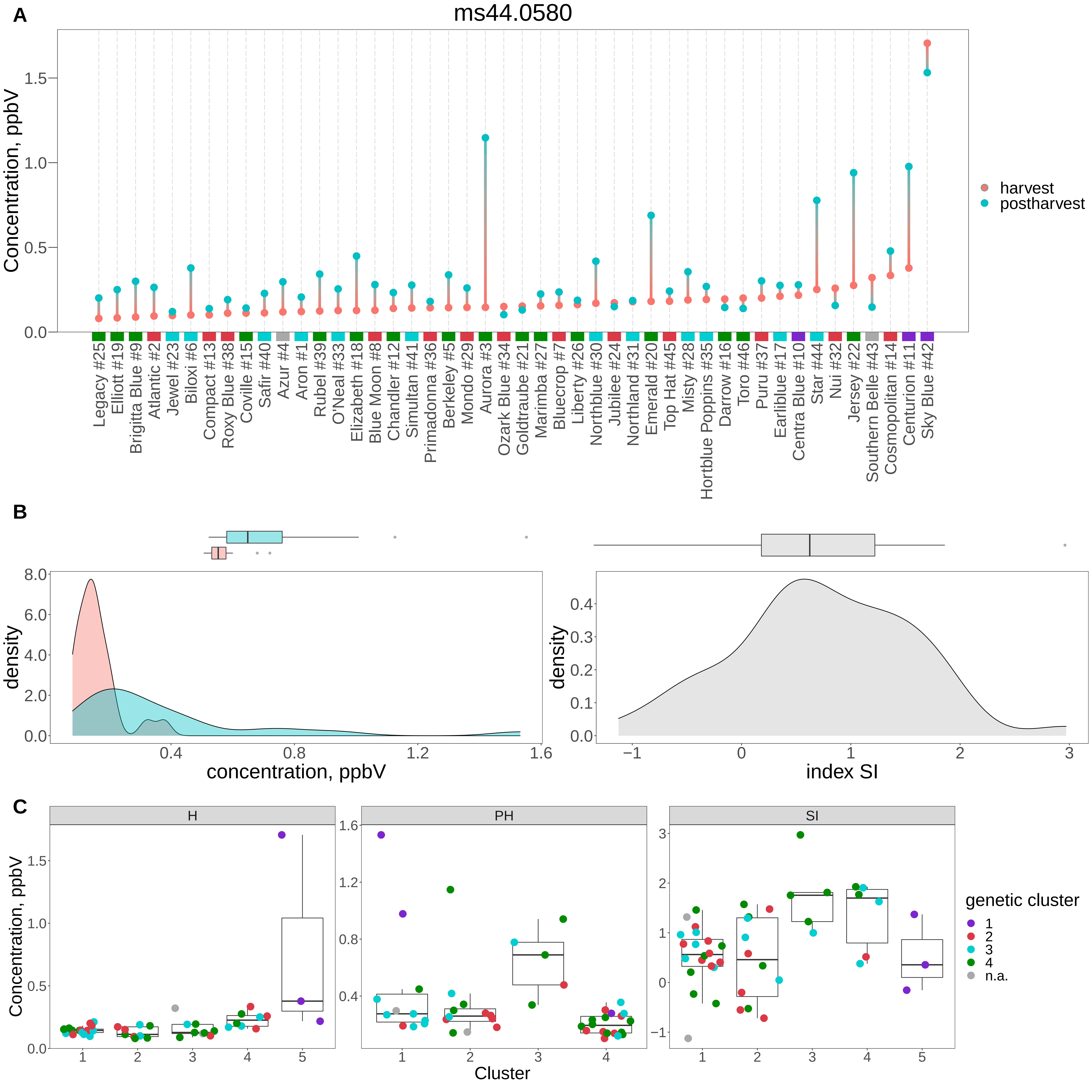
A
ms44.0580
Concentration, ppbV
harvest
postharvest
Legacy #25
Elliott #19
Brigitta Blue #9
Atlantic #2
Jewel #23
Biloxi #6
Compact #13
Roxy Blue #38
Coville #15
Safir #40
Azur #4
Aron #1
Rubel #39
O'Neal #33
Elizabeth #18
Blue Moon #8
Chandler #12
Simultan #41
Primadonna #36
Berkeley #5
Mondo #29
Aurora #3
Ozark Blue #34
Goldtraube #21
Marimba #27
Bluecrop #7
Liberty #26
Northblue #30
Jubilee #24
Northland #31
Emerald #20
Top Hat #45
Misty #28
Hortblue Poppins #35
Darrow #16
Toro #46
Puru #37
Earliblue #17
Centra Blue #10
Star #44
Nui #32
Jersey #22
Southern Belle #43
Cosmopolitan #14
Centurion #11
Sky Blue #42
B
density
concentration, ppbV
density
index SI
C
H
PH
SI
Concentration, ppbV
Cluster
genetic cluster
1
2
3
4
n.a.

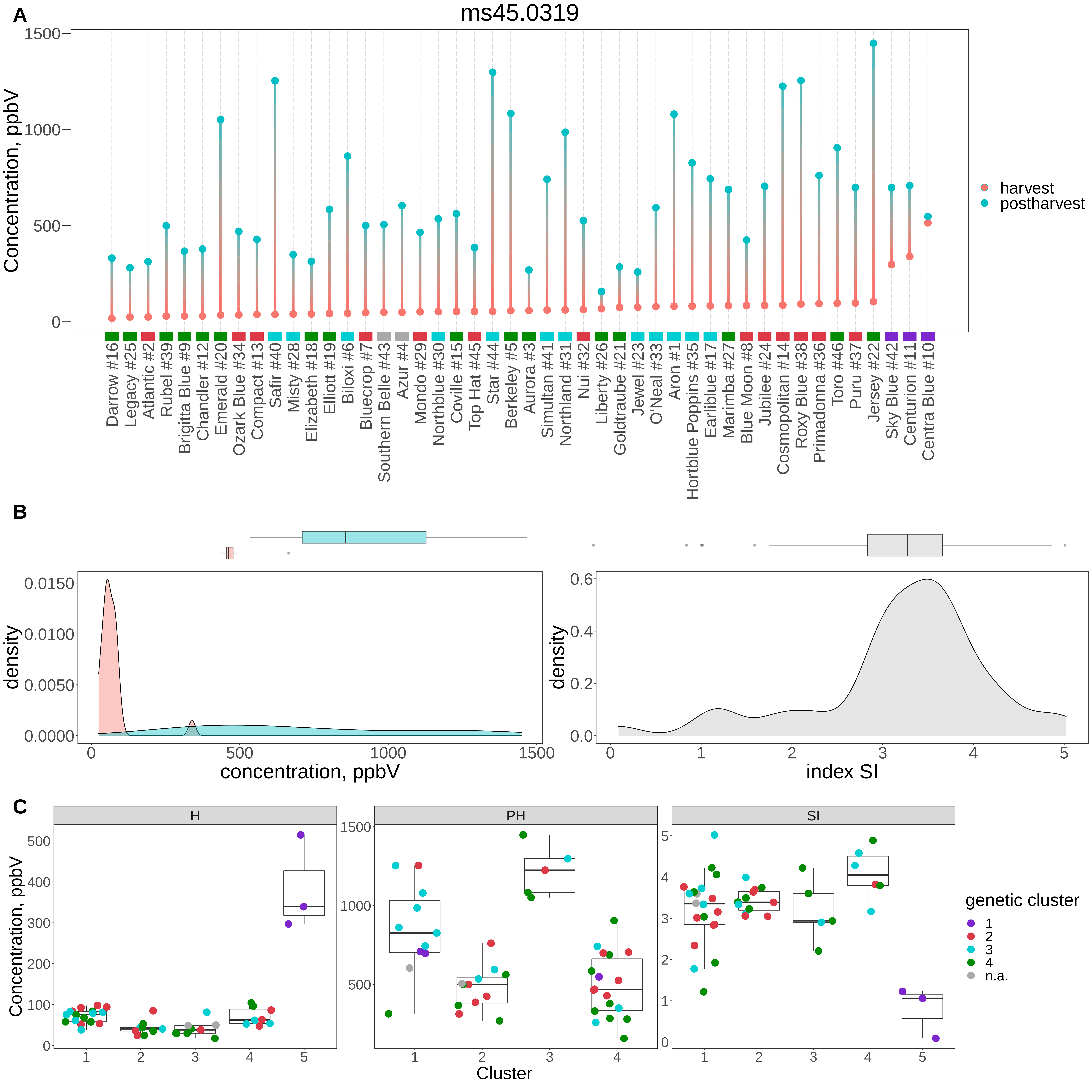

A
ms45.0319
Concentration, ppbV
harvest
postharvest
Darrow #16
Legacy #25
Atlantic #2
Rubel #39
Brigitta Blue #9
Chandler #12
Emerald #20
Ozark Blue #34
Compact #13
Safir #40
Misty #28
Elizabeth #18
Elliott #19
Biloxi #6
Bluecrop #7
Southern Belle #43
Azur #4
Mondo #29
Northblue #30
Coville #15
Top Hat #45
Star #44
Berkeley #5
Aurora #3
Simultan #41
Northland #31
Nui #32
Liberty #26
Goldtraube #21
Jewel #23
O'Neal #33
Aron #1
Hortblue Poppins #35
Earliblue #17
Marimba #27
Blue Moon #8
Jubilee #24
Cosmopolitan #14
Roxy Blue #38
Primadonna #36
Toro #46
Puru #37
Jersey #22
Sky Blue #42
Centurion #11
Centra Blue #10
B
density
concentration, ppbV
density
index SI
C
H
PH
SI
Concentration, ppbV
Cluster
genetic cluster
1
2
3
4
n.a.

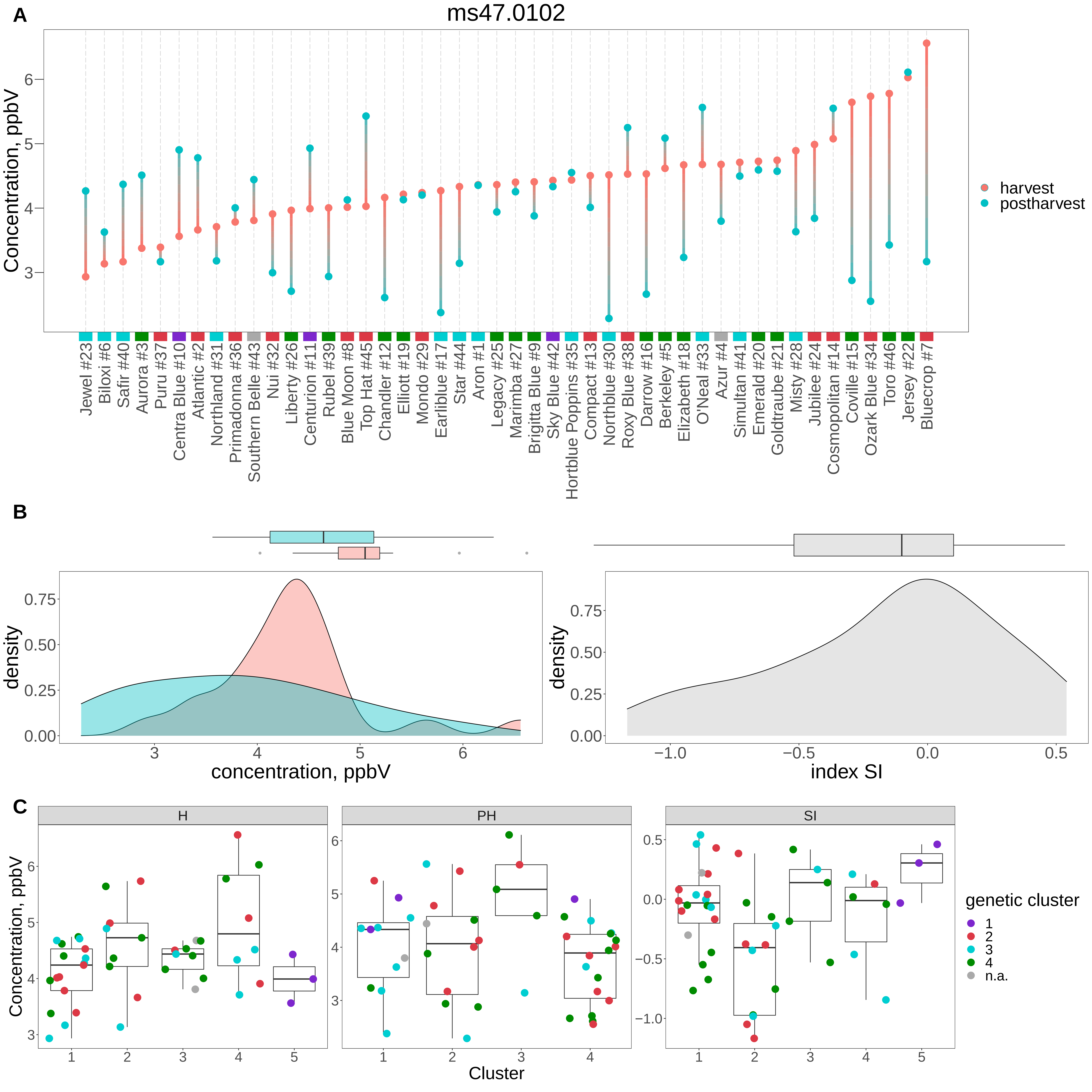
A
ms47.0102
Concentration, ppbV
harvest
postharvest
Jewel #23
Biloxi #6
Safir #40
Aurora #3
Puru #37
Centra Blue #10
Atlantic #2
Northland #31
Primadonna #36
Southern Belle #43
Nui #32
Liberty #26
Centurion #11
Rubel #39
Blue Moon #8
Top Hat #45
Chandler #12
Elliott #19
Mondo #29
Earliblue #17
Star #44
Aron #1
Legacy #25
Marimba #27
Brigitta Blue #9
Sky Blue #42
Hortblue Poppins #35
Compact #13
Northblue #30
Roxy Blue #38
Darrow #16
Berkeley #5
Elizabeth #18
O'Neal #33
Azur #4
Simultan #41
Emerald #20
Goldtraube #21
Misty #28
Jubilee #24
Cosmopolitan #14
Coville #15
Ozark Blue #34
Toro #46
Jersey #22
Bluecrop #7
B
density
concentration, ppbV
index SI
C
H
PH
SI
Concentration, ppbV
Cluster
genetic cluster
1
2
3
4
n.a.

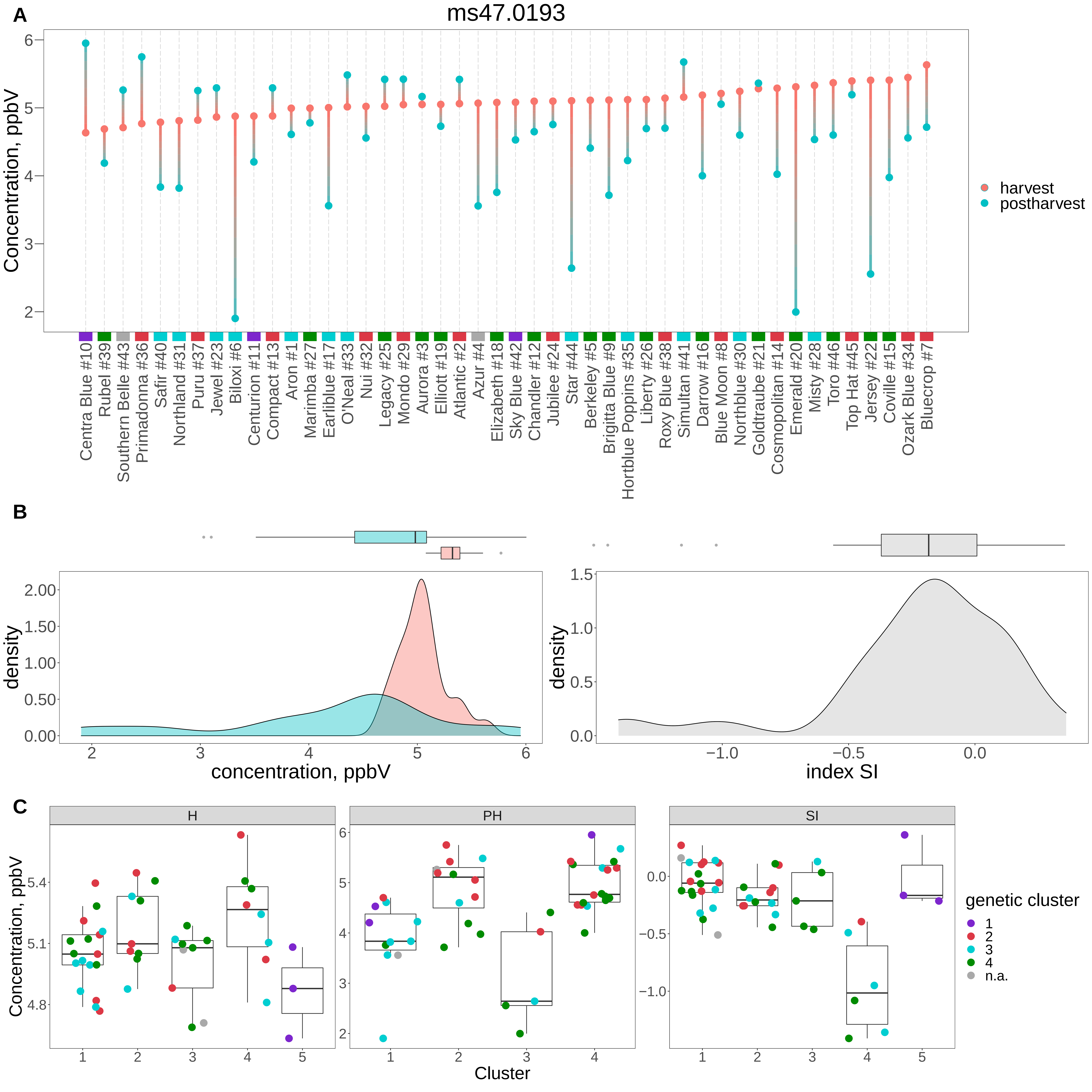
A
ms47.0193
Concentration, ppbV
harvest
postharvest
Centra Blue #10
Rubel #39
Southern Belle #43
Primadonna #36
Safir #40
Northland #31
Puru #37
Jewel #23
Biloxi #6
Centurion #11
Compact #13
Aron #1
Marimba #27
Earliblue #17
O'Neal #33
Nui #32
Legacy #25
Mondo #29
Aurora #3
Elliott #19
Atlantic #2
Azur #4
Elizabeth #18
Sky Blue #42
Chandler #12
Jubilee #24
Star #44
Berkeley #5
Brigitta Blue #9
Hortblue Poppins #35
Liberty #26
Roxy Blue #38
Simultan #41
Darrow #16
Blue Moon #8
Northblue #30
Goldtraube #21
Cosmopolitan #14
Emerald #20
Misty #28
Toro #46
Top Hat #45
Jersey #22
Coville #15
Ozark Blue #34
Bluecrop #7
B
density
concentration, ppbV
index SI
C
H
PH
SI
Concentration, ppbV
Cluster
genetic cluster
1
2
3
4
n.a.

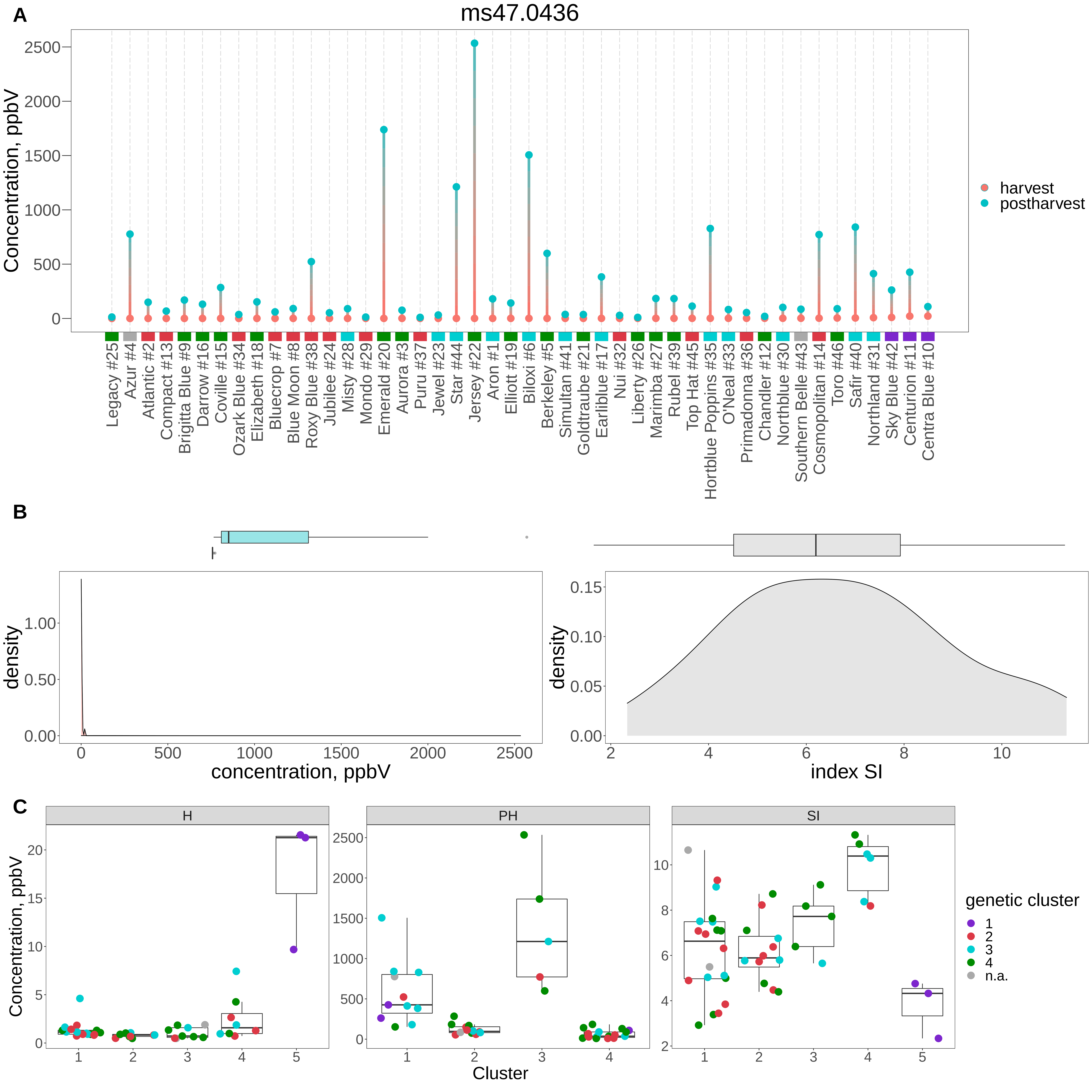

A
ms47.0436
Concentration, ppbV
harvest
postharvest
Legacy #25
Azur #4
Atlantic #2
Compact #13
Brigitta Blue #9
Darrow #16
Coville #15
Ozark Blue #34
Elizabeth #18
Bluecrop #7
Blue Moon #8
Roxy Blue #38
Jubilee #24
Misty #28
Mondo #29
Emerald #20
Aurora #3
Puru #37
Jewel #23
Star #44
Jersey #22
Aron #1
Elliott #19
Biloxi #6
Berkeley #5
Simultan #41
Goldtraube #21
Earliblue #17
Nui #32
Liberty #26
Marimba #27
Rubel #39
Top Hat #45
Hortblue Poppins #35
O'Neal #33
Primadonna #36
Chandler #12
Northblue #30
Southern Belle #43
Cosmopolitan #14
Toro #46
Safir #40
Northland #31
Sky Blue #42
Centurion #11
Centra Blue #10
B
density
concentration, ppbV
index SI
C
H
PH
SI
Cluster
genetic cluster
1
2
3
4
n.a.

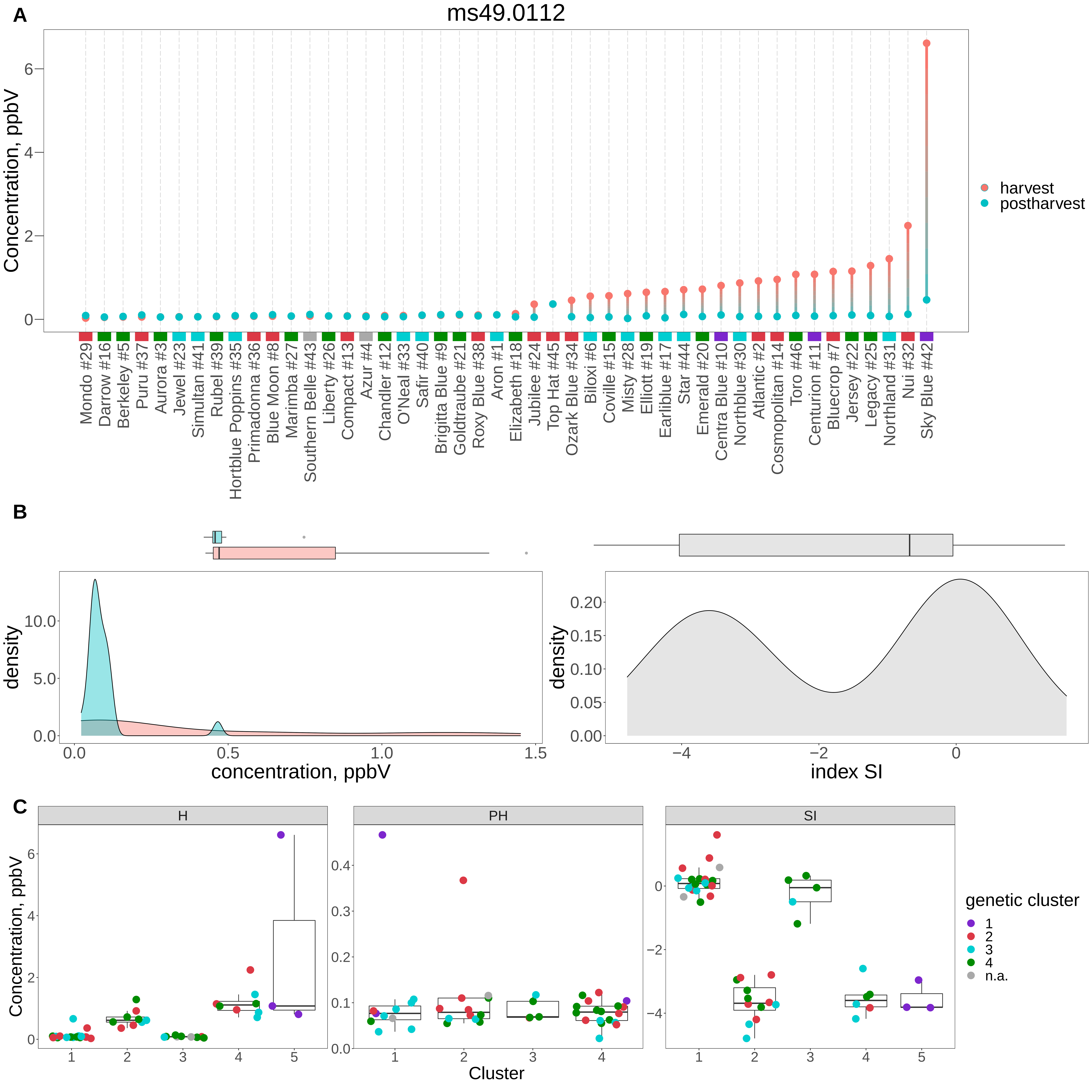

A
ms49.0112
Concentration, ppbV
harvest
postharvest
Mondo #29
Darrow #16
Berkeley #5
Puru #37
Aurora #3
Jewel #23
Simultan #41
Rubel #39
Hortblue Poppins #35
Primadonna #36
Blue Moon #8
Marimba #27
Southern Belle #43
Liberty #26
Compact #13
Azur #4
Chandler #12
O'Neal #33
Safir #40
Brigitta Blue #9
Goldtraube #21
Roxy Blue #38
Aron #1
Elizabeth #18
Jubilee #24
Top Hat #45
Ozark Blue #34
Biloxi #6
Coville #15
Misty #28
Elliott #19
Earliblue #17
Star #44
Emerald #20
Centra Blue #10
Northblue #30
Atlantic #2
Cosmopolitan #14
Toro #46
Centurion #11
Bluecrop #7
Jersey #22
Legacy #25
Northland #31
Nui #32
Sky Blue #42
B
density
concentration, ppbV
index SI
C
H
PH
SI
Concentration, ppbV
Cluster
genetic cluster
1
2
3
4
n.a.

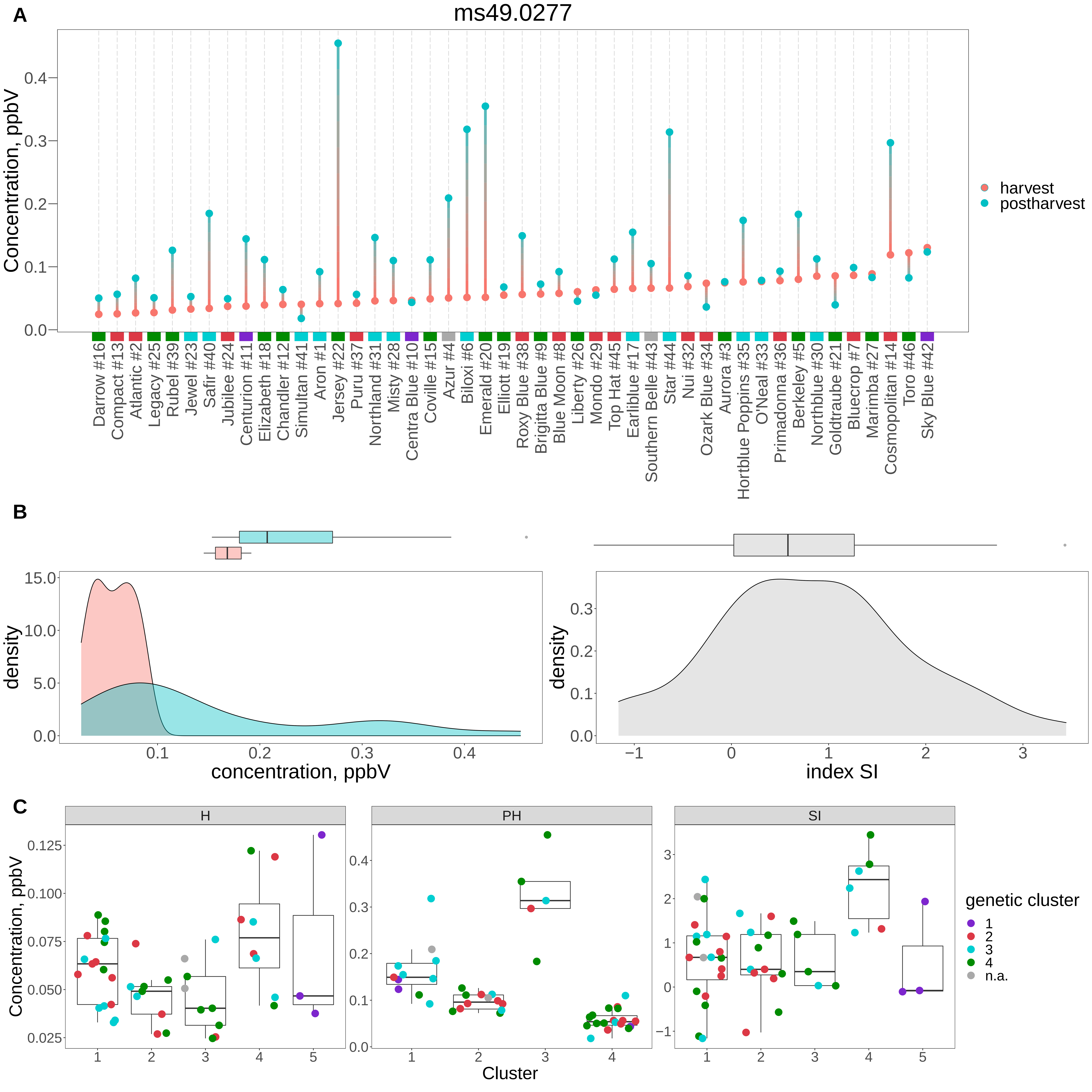
A
ms49.0277
Concentration, ppbV
0.0
0.1
0.2
0.3
0.4
harvest
postharvest
Darrow #16
Compact #13
Atlantic #2
Legacy #25
Rubel #39
Jewel #23
Safir #40
Jubilee #24
Centurion #11
Elizabeth #18
Chandler #12
Simultan #41
Aron #1
Jersey #22
Puru #37
Northland #31
Misty #28
Centra Blue #10
Coville #15
Azur #4
Biloxi #6
Emerald #20
Elliott #19
Roxy Blue #38
Brigitta Blue #9
Blue Moon #8
Liberty #26
Mondo #29
Top Hat #45
Earliblue #17
Southern Belle #43
Star #44
Nui #32
Ozark Blue #34
Aurora #3
Hortblue Poppins #35
O'Neal #33
Primadonna #36
Berkeley #5
Northblue #30
Goldtraube #21
Bluecrop #7
Marimba #27
Cosmopolitan #14
Toro #46
Sky Blue #42
B
density
0.0
5.0
10.0
15.0
0.1
0.2
0.3
0.4
concentration, ppbV
density
0.0
0.1
0.2
0.3
−1
0
1
2
3
index SI
C
H
PH
SI
Concentration, ppbV
0.025
0.050
0.075
0.100
0.125
1
2
3
4
5
0.0
0.1
0.2
0.3
0.4
1
2
3
4
Cluster
−1
0
1
2
3
1
2
3
4
5
genetic cluster
1
2
3
4
n.a.

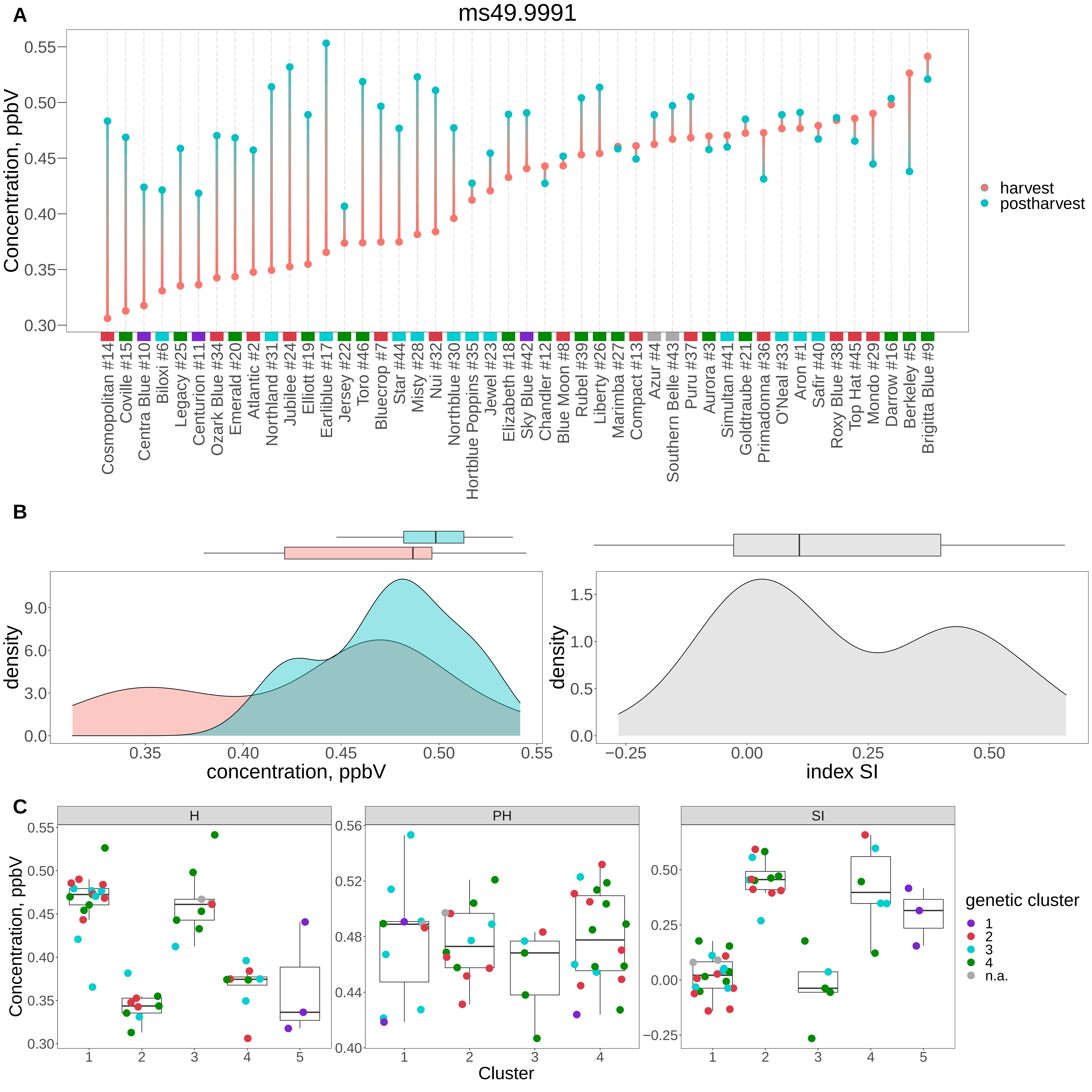
A
ms49.9991
Concentration, ppbV
harvest
postharvest
Cosmopolitan #14
Coville #15
Centra Blue #10
Biloxi #6
Legacy #25
Centurion #11
Ozark Blue #34
Emerald #20
Atlantic #2
Northland #31
Jubilee #24
Elliott #19
Earliblue #17
Jersey #22
Toro #46
Bluecrop #7
Star #44
Misty #28
Nui #32
Northblue #30
Hortblue Poppins #35
Jewel #23
Elizabeth #18
Sky Blue #42
Chandler #12
Blue Moon #8
Rubel #39
Liberty #26
Marimba #27
Compact #13
Azur #4
Southern Belle #43
Puru #37
Aurora #3
Simultan #41
Goldtraube #21
Primadonna #36
O'Neal #33
Aron #1
Safir #40
Roxy Blue #38
Top Hat #45
Mondo #29
Darrow #16
Berkeley #5
Brigitta Blue #9
B
density
concentration, ppbV
index SI
C
H
PH
SI
Concentration, ppbV
Cluster
genetic cluster
1
2
3
4
n.a.

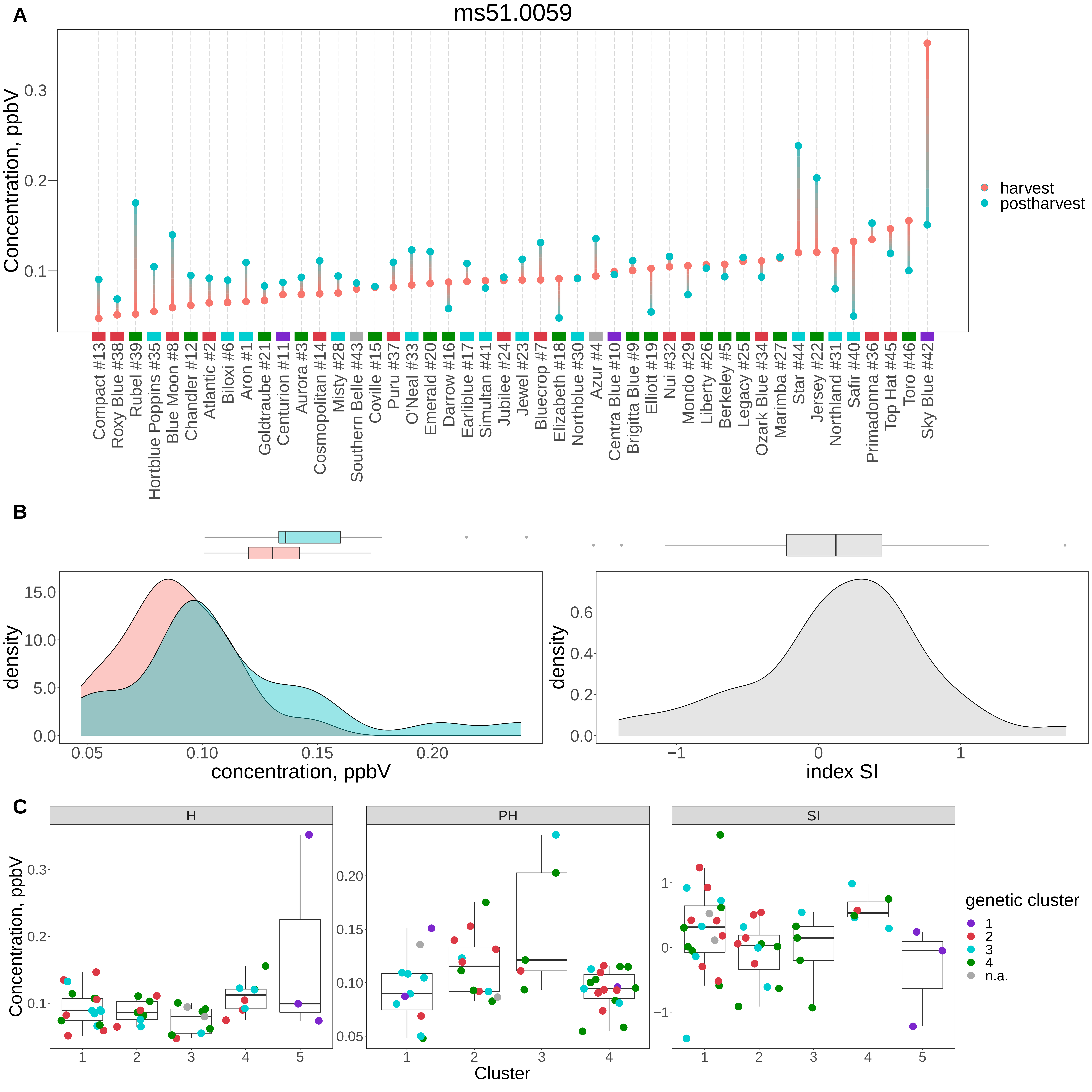

A
ms51.0059
Concentration, ppbV
harvest
postharvest
Compact #13
Roxy Blue #38
Rubel #39
Hortblue Poppins #35
Blue Moon #8
Chandler #12
Atlantic #2
Biloxi #6
Aron #1
Goldtraube #21
Centurion #11
Aurora #3
Cosmopolitan #14
Misty #28
Southern Belle #43
Coville #15
Puru #37
O'Neal #33
Emerald #20
Darrow #16
Earliblue #17
Simultan #41
Jubilee #24
Jewel #23
Bluecrop #7
Elizabeth #18
Northblue #30
Azur #4
Centra Blue #10
Brigitta Blue #9
Elliott #19
Nui #32
Mondo #29
Liberty #26
Berkeley #5
Legacy #25
Ozark Blue #34
Marimba #27
Star #44
Jersey #22
Northland #31
Safir #40
Primadonna #36
Top Hat #45
Toro #46
Sky Blue #42
B
density
concentration, ppbV
index SI
C
H
PH
SI
Cluster
genetic cluster
1
2
3
4
n.a.

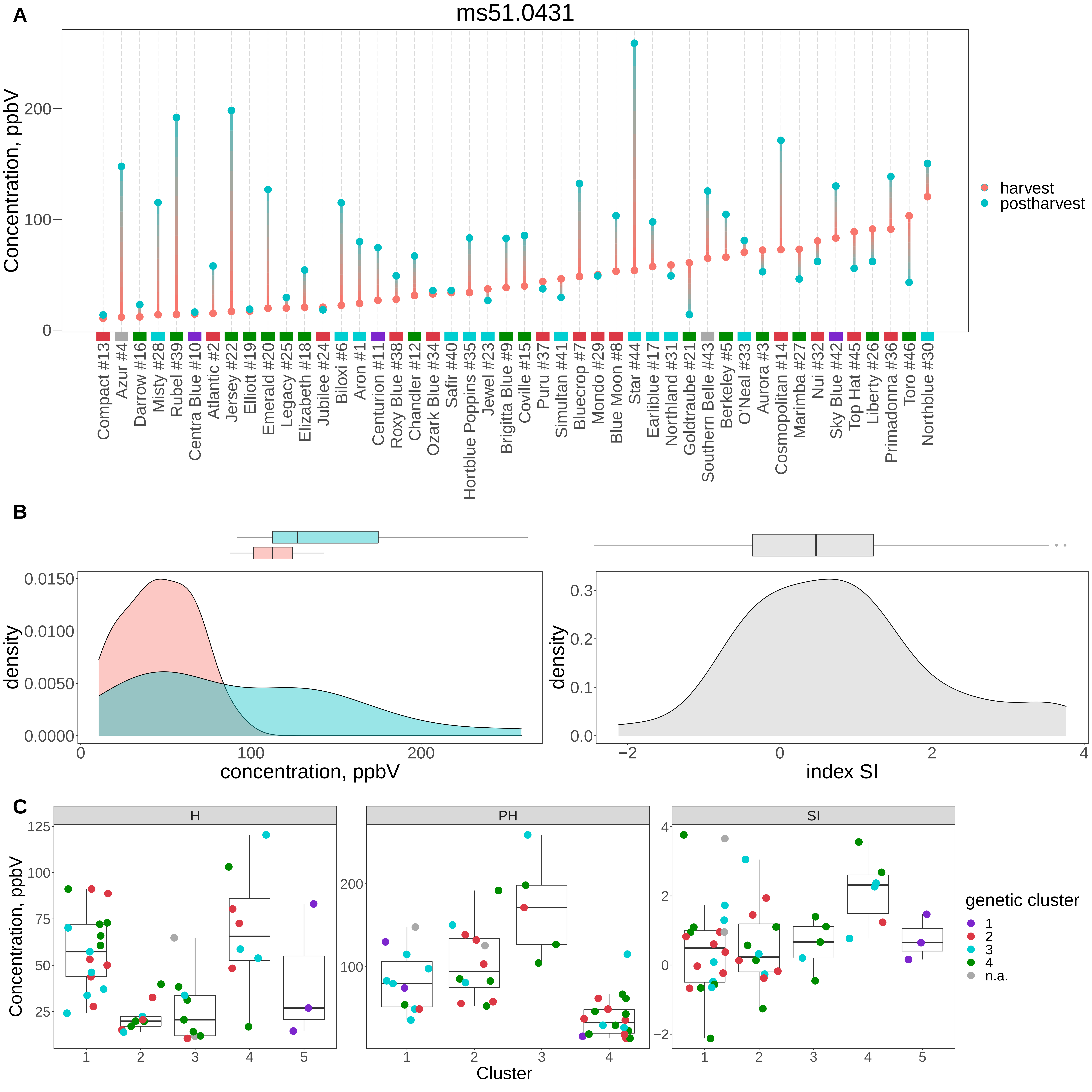
A
ms51.0431
Concentration, ppbV
0
100
200
harvest
postharvest
Compact #13
Azur #4
Darrow #16
Misty #28
Rubel #39
Centra Blue #10
Atlantic #2
Jersey #22
Elliott #19
Emerald #20
Legacy #25
Elizabeth #18
Jubilee #24
Biloxi #6
Aron #1
Centurion #11
Roxy Blue #38
Chandler #12
Ozark Blue #34
Safir #40
Hortblue Poppins #35
Jewel #23
Brigitta Blue #9
Coville #15
Puru #37
Simultan #41
Bluecrop #7
Mondo #29
Blue Moon #8
Star #44
Earliblue #17
Northland #31
Goldtraube #21
Southern Belle #43
Berkeley #5
O'Neal #33
Aurora #3
Cosmopolitan #14
Marimba #27
Nui #32
Sky Blue #42
Top Hat #45
Liberty #26
Primadonna #36
Toro #46
Northblue #30
B
density
0.0000
0.0050
0.0100
0.0150
0
100
200
concentration, ppbV
density
0.0
0.1
0.2
0.3
−2
0
2
4
index SI
C
H
PH
SI
Concentration, ppbV
25
50
75
100
125
100
200
−2
0
2
4
1
2
3
4
5
Cluster
genetic cluster
1
2
3
4
n.a.

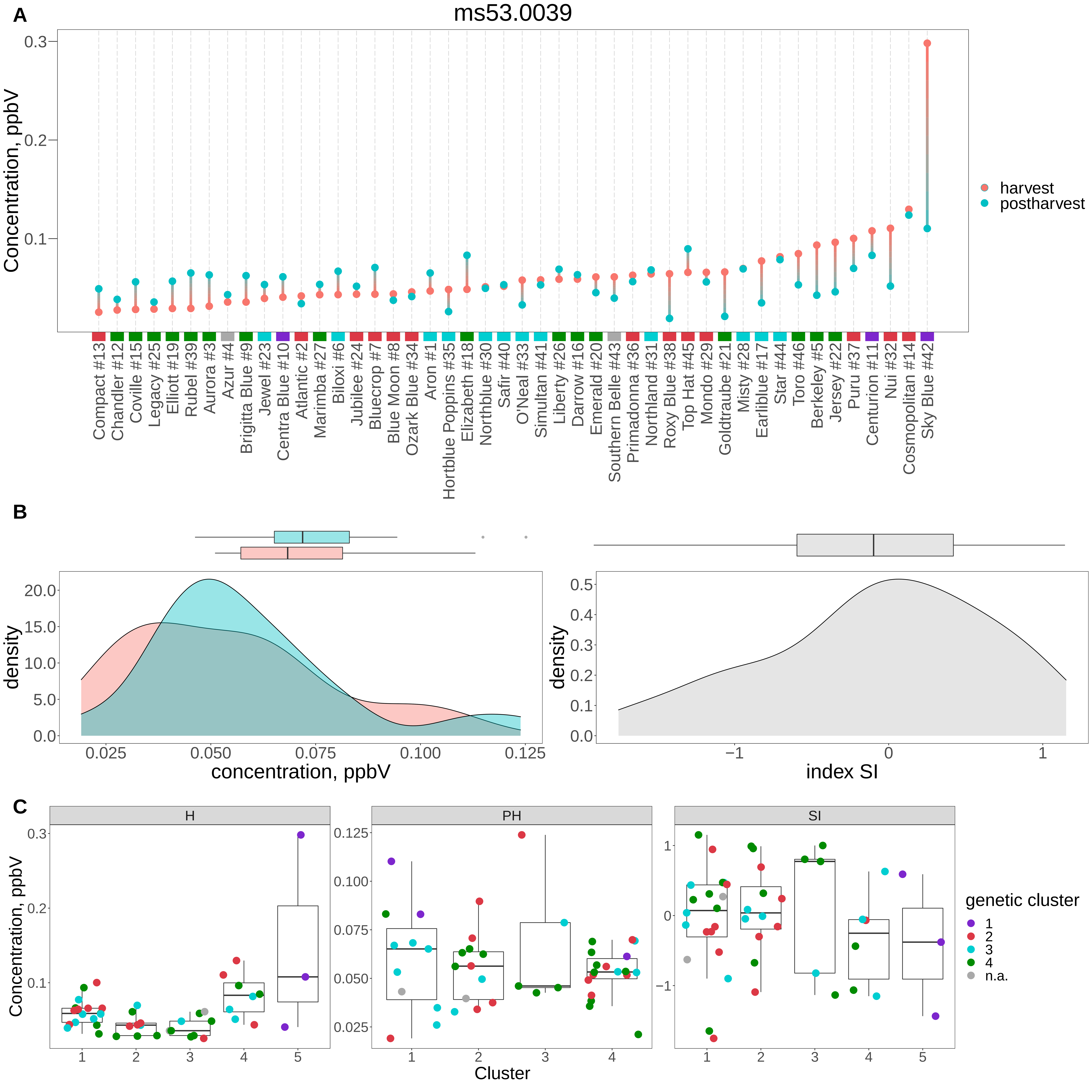
A
ms53.0039
Concentration, ppbV
0.1
0.2
0.3
harvest
postharvest
Compact #13
Chandler #12
Coville #15
Legacy #25
Elliott #19
Rubel #39
Aurora #3
Azur #4
Brigitta Blue #9
Jewel #23
Centra Blue #10
Atlantic #2
Marimba #27
Biloxi #6
Jubilee #24
Bluecrop #7
Blue Moon #8
Ozark Blue #34
Aron #1
Hortblue Poppins #35
Elizabeth #18
Northblue #30
Safir #40
O'Neal #33
Simultan #41
Liberty #26
Darrow #16
Emerald #20
Southern Belle #43
Primadonna #36
Northland #31
Roxy Blue #38
Top Hat #45
Mondo #29
Goldtraube #21
Misty #28
Earliblue #17
Star #44
Toro #46
Berkeley #5
Jersey #22
Puru #37
Centurion #11
Nui #32
Cosmopolitan #14
Sky Blue #42
B
density
0.0
5.0
10.0
15.0
20.0
0.025
0.050
0.075
0.100
0.125
concentration, ppbV
density
0.0
0.1
0.2
0.3
0.4
0.5
−1
0
1
index SI
C
H
PH
SI
Concentration, ppbV
0.1
0.2
0.3
0.025
0.050
0.075
0.100
0.125
−1
0
1
1
2
3
4
5
Cluster
genetic cluster
1
2
3
4
n.a.

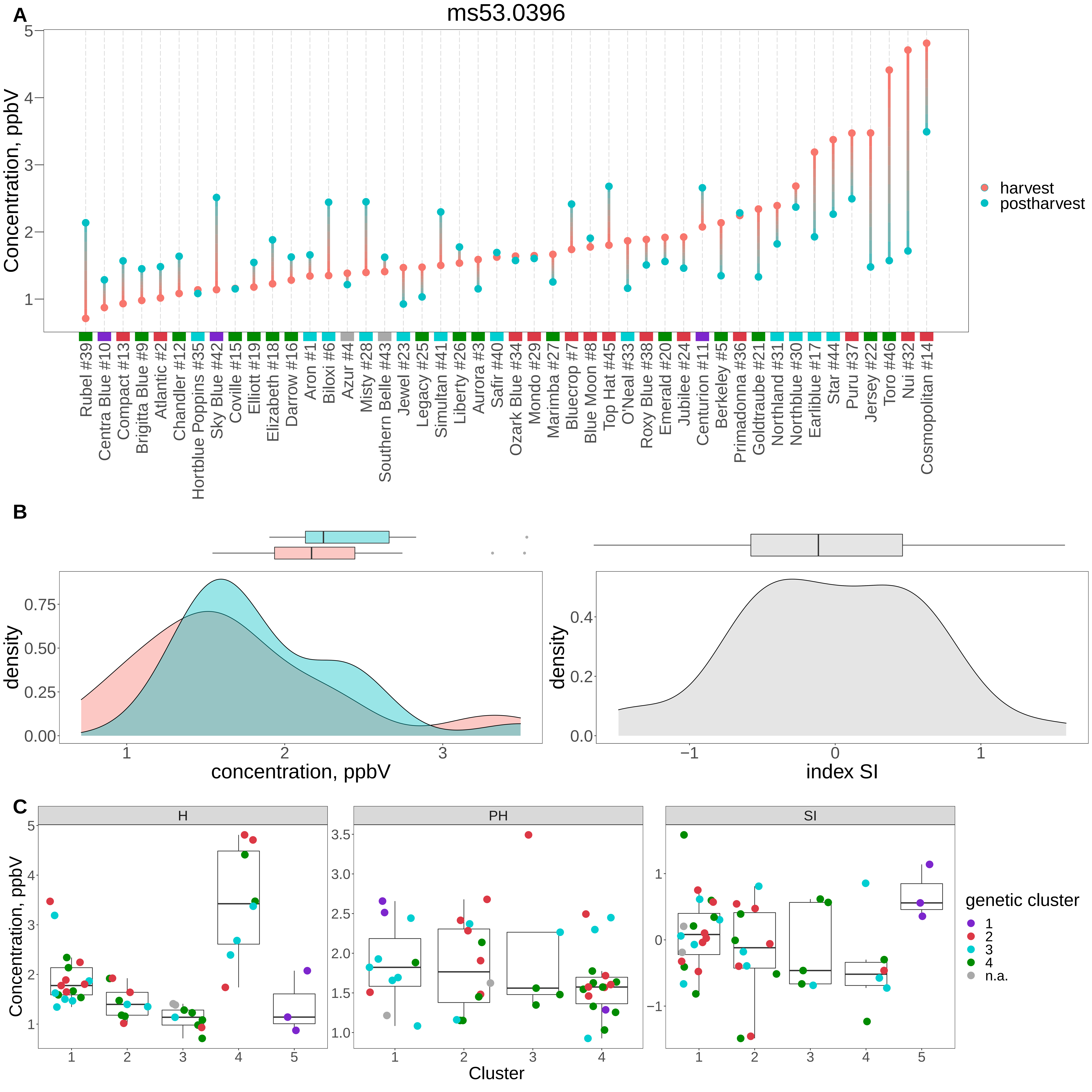
A
ms53.0396
Concentration, ppbV
harvest
postharvest
Rubel #39
Centra Blue #10
Compact #13
Brigitta Blue #9
Atlantic #2
Chandler #12
Hortblue Poppins #35
Sky Blue #42
Coville #15
Elliott #19
Elizabeth #18
Darrow #16
Aron #1
Biloxi #6
Azur #4
Misty #28
Southern Belle #43
Jewel #23
Legacy #25
Simultan #41
Liberty #26
Aurora #3
Safir #40
Ozark Blue #34
Mondo #29
Marimba #27
Bluecrop #7
Blue Moon #8
Top Hat #45
O'Neal #33
Roxy Blue #38
Emerald #20
Jubilee #24
Centurion #11
Berkeley #5
Primadonna #36
Goldtraube #21
Northland #31
Northblue #30
Earliblue #17
Star #44
Puru #37
Jersey #22
Toro #46
Nui #32
Cosmopolitan #14
B
density
concentration, ppbV
density
index SI
C
H
PH
SI
Concentration, ppbV
Cluster
genetic cluster
1
2
3
4
n.a.

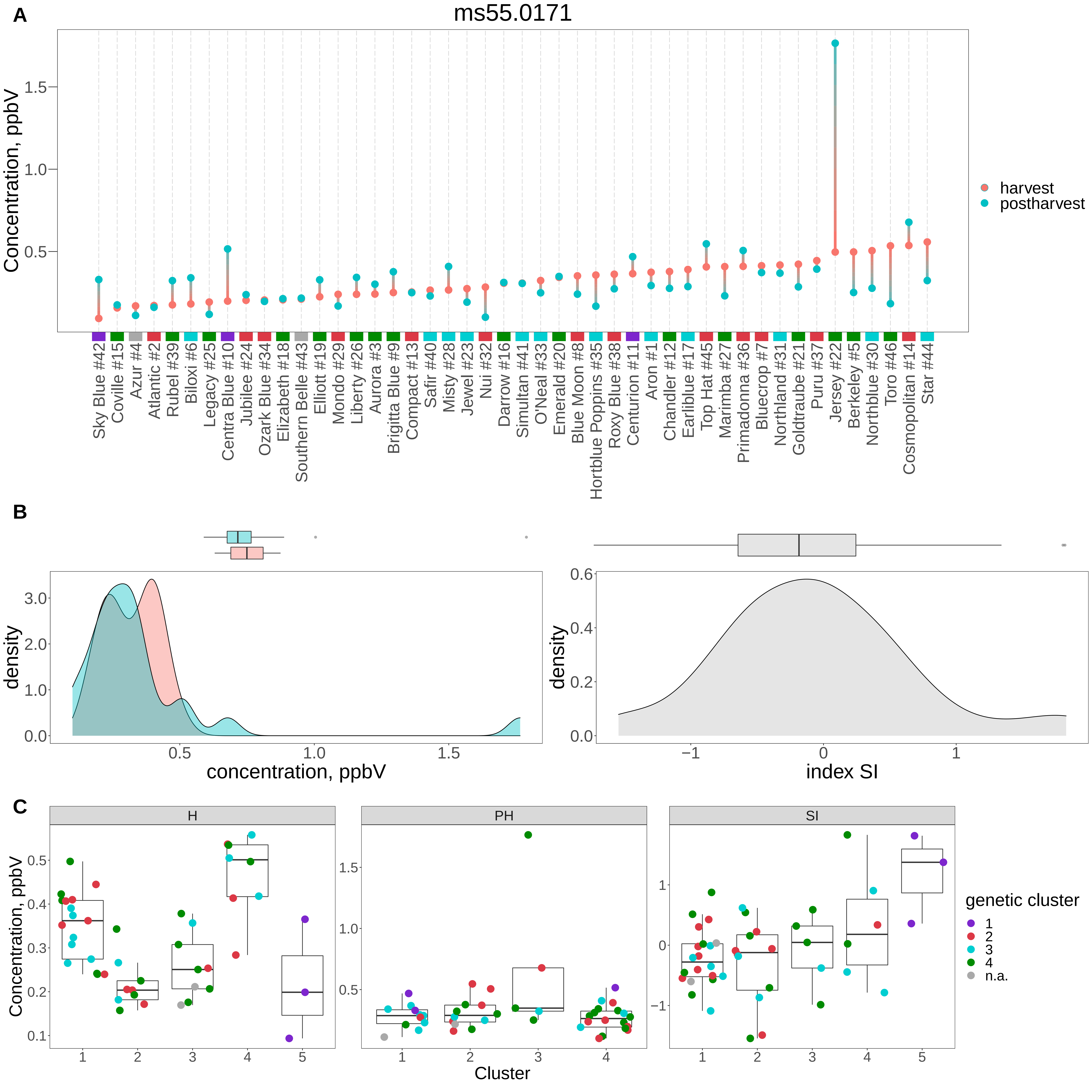

A
ms55.0171
Concentration, ppbV
harvest
postharvest
Sky Blue #42
Coville #15
Azur #4
Atlantic #2
Rubel #39
Biloxi #6
Legacy #25
Centra Blue #10
Jubilee #24
Ozark Blue #34
Elizabeth #18
Southern Belle #43
Elliott #19
Mondo #29
Liberty #26
Aurora #3
Brigitta Blue #9
Compact #13
Safir #40
Misty #28
Jewel #23
Nui #32
Darrow #16
Simultan #41
O'Neal #33
Emerald #20
Blue Moon #8
Hortblue Poppins #35
Roxy Blue #38
Centurion #11
Aron #1
Chandler #12
Earliblue #17
Top Hat #45
Marimba #27
Primadonna #36
Bluecrop #7
Northland #31
Goldtraube #21
Puru #37
Jersey #22
Berkeley #5
Northblue #30
Toro #46
Cosmopolitan #14
Star #44
B
density
concentration, ppbV
density
index SI
C
H
PH
SI
Concentration, ppbV
Cluster
genetic cluster
1
2
3
4
n.a.

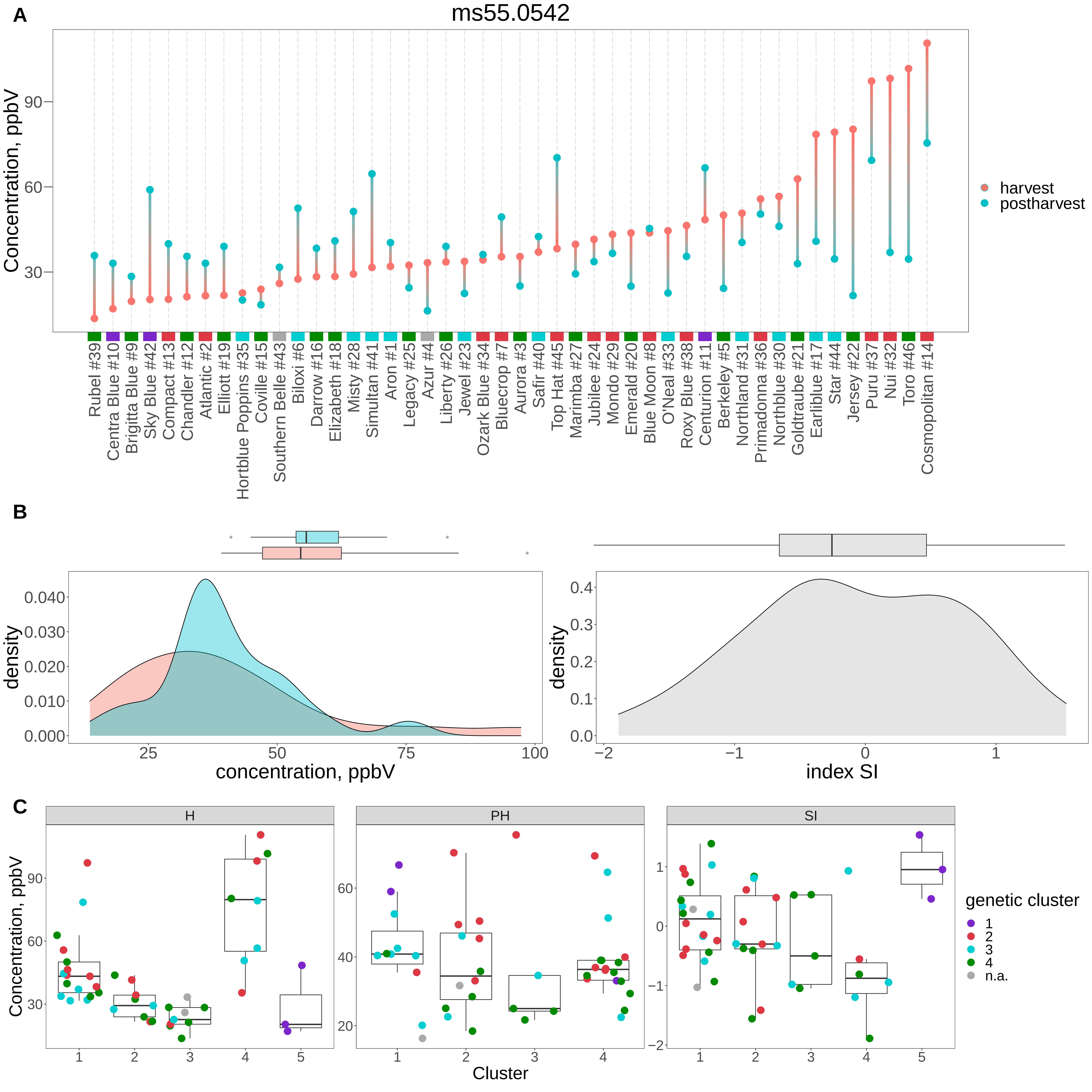

A
ms55.0542
Concentration, ppbV
harvest
postharvest
Rubel #39
Centra Blue #10
Brigitta Blue #9
Sky Blue #42
Compact #13
Chandler #12
Atlantic #2
Elliott #19
Hortblue Poppins #35
Coville #15
Southern Belle #43
Biloxi #6
Darrow #16
Elizabeth #18
Misty #28
Simultan #41
Aron #1
Legacy #25
Azur #4
Liberty #26
Jewel #23
Ozark Blue #34
Bluecrop #7
Aurora #3
Safir #40
Top Hat #45
Marimba #27
Jubilee #24
Mondo #29
Emerald #20
Blue Moon #8
O'Neal #33
Roxy Blue #38
Centurion #11
Berkeley #5
Northland #31
Primadonna #36
Northblue #30
Goldtraube #21
Earliblue #17
Star #44
Jersey #22
Puru #37
Nui #32
Toro #46
Cosmopolitan #14
B
density
concentration, ppbV
index SI
C
H
PH
SI
Concentration, ppbV
Cluster
genetic cluster
1
2
3
4
n.a.

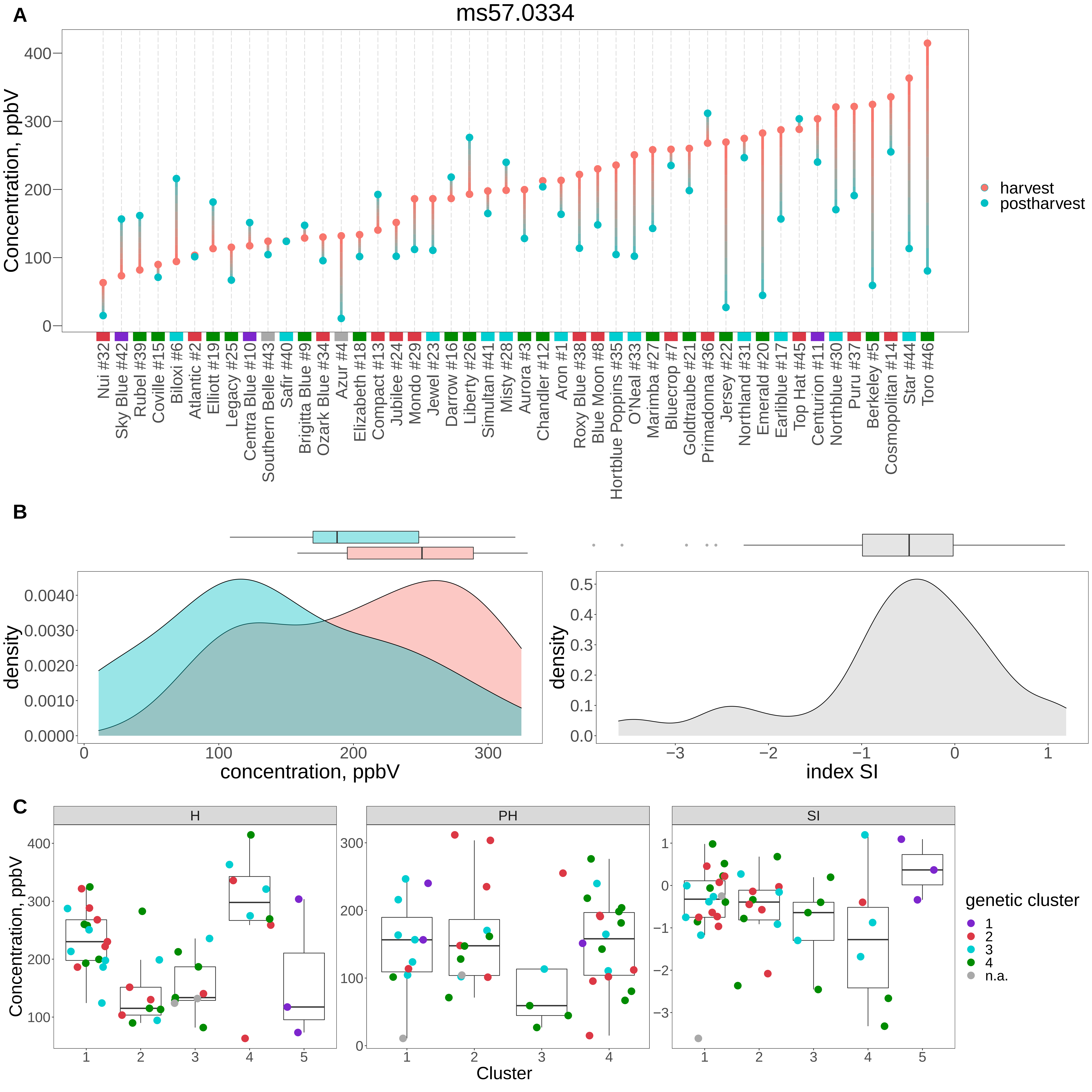

A
ms57.0334
Concentration, ppbV
harvest
postharvest
Nui #32
Sky Blue #42
Rubel #39
Coville #15
Biloxi #6
Atlantic #2
Elliott #19
Legacy #25
Centra Blue #10
Southern Belle #43
Safir #40
Brigitta Blue #9
Ozark Blue #34
Azur #4
Elizabeth #18
Compact #13
Jubilee #24
Mondo #29
Jewel #23
Darrow #16
Liberty #26
Simultan #41
Misty #28
Aurora #3
Chandler #12
Aron #1
Roxy Blue #38
Blue Moon #8
Hortblue Poppins #35
O'Neal #33
Marimba #27
Bluecrop #7
Goldtraube #21
Primadonna #36
Jersey #22
Northland #31
Emerald #20
Earliblue #17
Top Hat #45
Centurion #11
Northblue #30
Puru #37
Berkeley #5
Cosmopolitan #14
Star #44
Toro #46
B
density
concentration, ppbV
index SI
C
H
PH
SI
Concentration, ppbV
Cluster
genetic cluster
1
2
3
4
n.a.

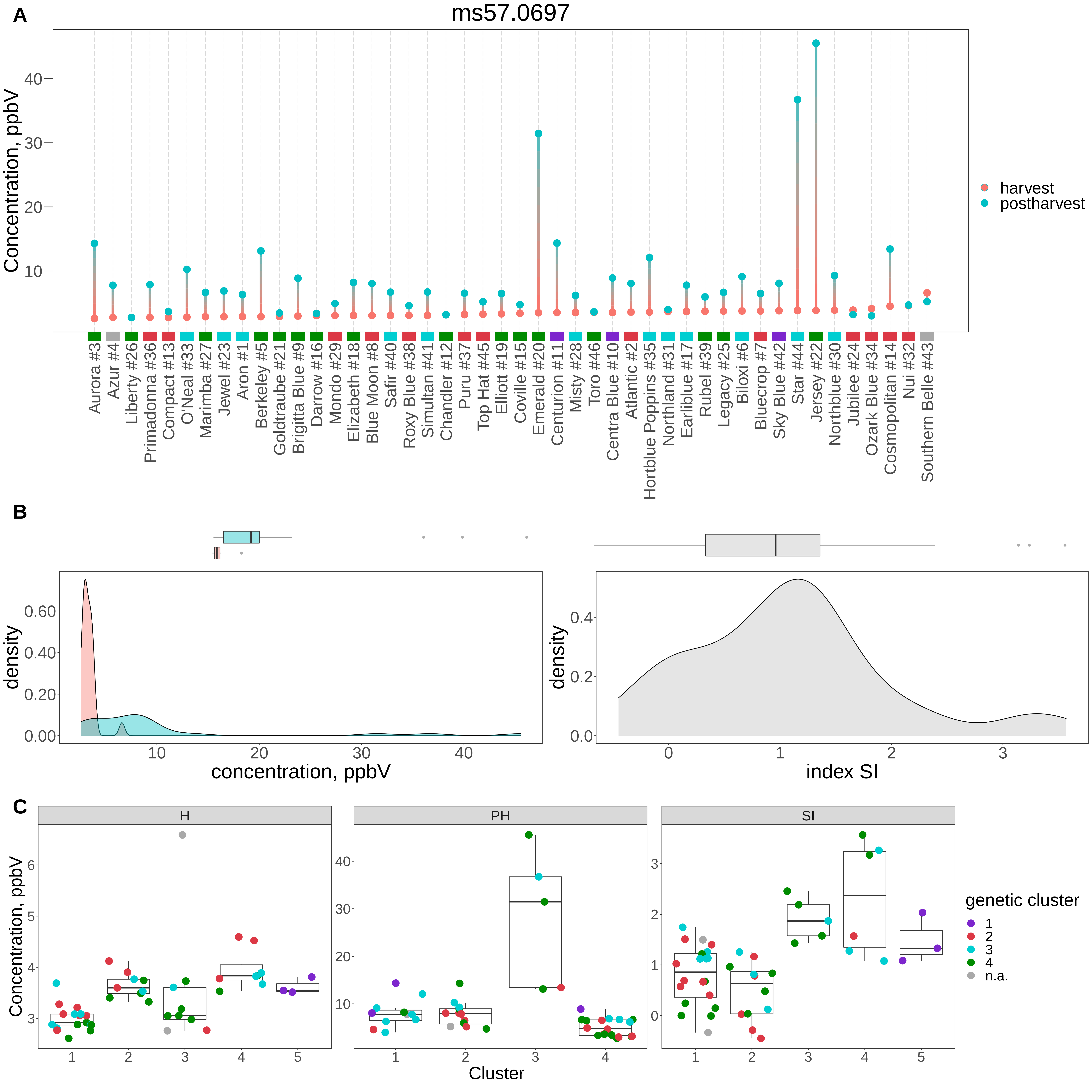
A
ms57.0697
Concentration, ppbV
harvest
postharvest
Aurora #3
Azur #4
Liberty #26
Primadonna #36
Compact #13
O'Neal #33
Marimba #27
Jewel #23
Aron #1
Berkeley #5
Goldtraube #21
Brigitta Blue #9
Darrow #16
Mondo #29
Elizabeth #18
Blue Moon #8
Safir #40
Roxy Blue #38
Simultan #41
Chandler #12
Puru #37
Top Hat #45
Elliott #19
Coville #15
Emerald #20
Centurion #11
Misty #28
Toro #46
Centra Blue #10
Atlantic #2
Hortblue Poppins #35
Northland #31
Earliblue #17
Rubel #39
Legacy #25
Biloxi #6
Bluecrop #7
Sky Blue #42
Star #44
Jersey #22
Northblue #30
Jubilee #24
Ozark Blue #34
Cosmopolitan #14
Nui #32
Southern Belle #43
B
density
concentration, ppbV
index SI
C
H
PH
SI
Concentration, ppbV
Cluster
genetic cluster
1
2
3
4
n.a.

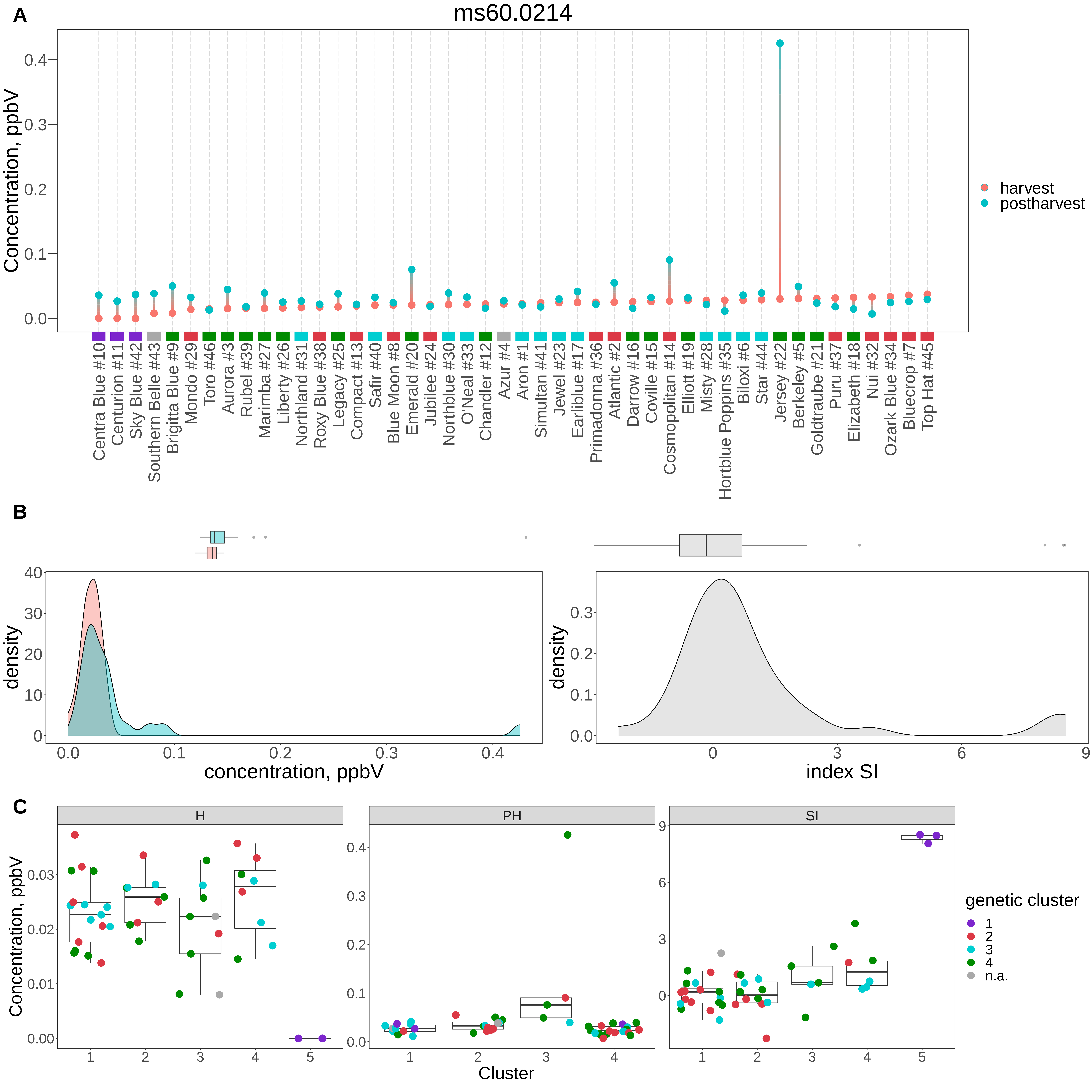

A
ms60.0214
Concentration, ppbV
harvest
postharvest
Centra Blue #10
Centurion #11
Sky Blue #42
Southern Belle #43
Brigitta Blue #9
Mondo #29
Toro #46
Aurora #3
Rubel #39
Marimba #27
Liberty #26
Northland #31
Roxy Blue #38
Legacy #25
Compact #13
Safir #40
Blue Moon #8
Emerald #20
Jubilee #24
Northblue #30
O'Neal #33
Chandler #12
Azur #4
Aron #1
Simultan #41
Jewel #23
Earliblue #17
Primadonna #36
Atlantic #2
Darrow #16
Coville #15
Cosmopolitan #14
Elliott #19
Misty #28
Hortblue Poppins #35
Biloxi #6
Star #44
Jersey #22
Berkeley #5
Goldtraube #21
Puru #37
Elizabeth #18
Nui #32
Ozark Blue #34
Bluecrop #7
Top Hat #45
B
density
concentration, ppbV
index SI
C
H
PH
SI
Concentration, ppbV
Cluster
genetic cluster
1
2
3
4
n.a.

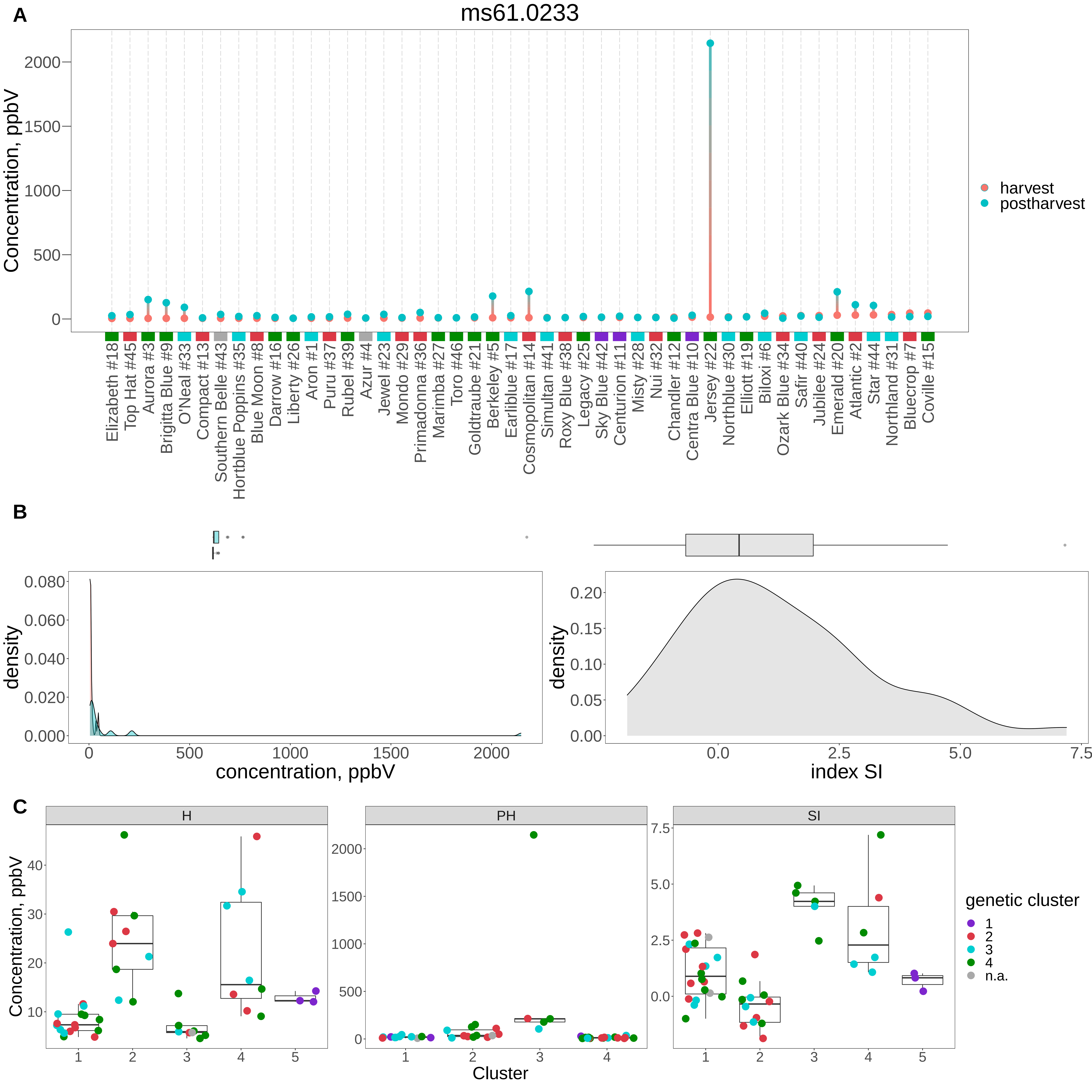

A
ms61.0233
Concentration, ppbV
harvest
postharvest
Elizabeth #18
Top Hat #45
Aurora #3
Brigitta Blue #9
O'Neal #33
Compact #13
Southern Belle #43
Hortblue Poppins #35
Blue Moon #8
Darrow #16
Liberty #26
Aron #1
Puru #37
Rubel #39
Azur #4
Jewel #23
Mondo #29
Primadonna #36
Marimba #27
Toro #46
Goldtraube #21
Berkeley #5
Earliblue #17
Cosmopolitan #14
Simultan #41
Roxy Blue #38
Legacy #25
Sky Blue #42
Centurion #11
Misty #28
Nui #32
Chandler #12
Centra Blue #10
Jersey #22
Northblue #30
Elliott #19
Biloxi #6
Ozark Blue #34
Safir #40
Jubilee #24
Emerald #20
Atlantic #2
Star #44
Northland #31
Bluecrop #7
Coville #15
B
density
concentration, ppbV
index SI
C
H
PH
SI
Concentration, ppbV
Cluster
genetic cluster
1
2
3
4
n.a.

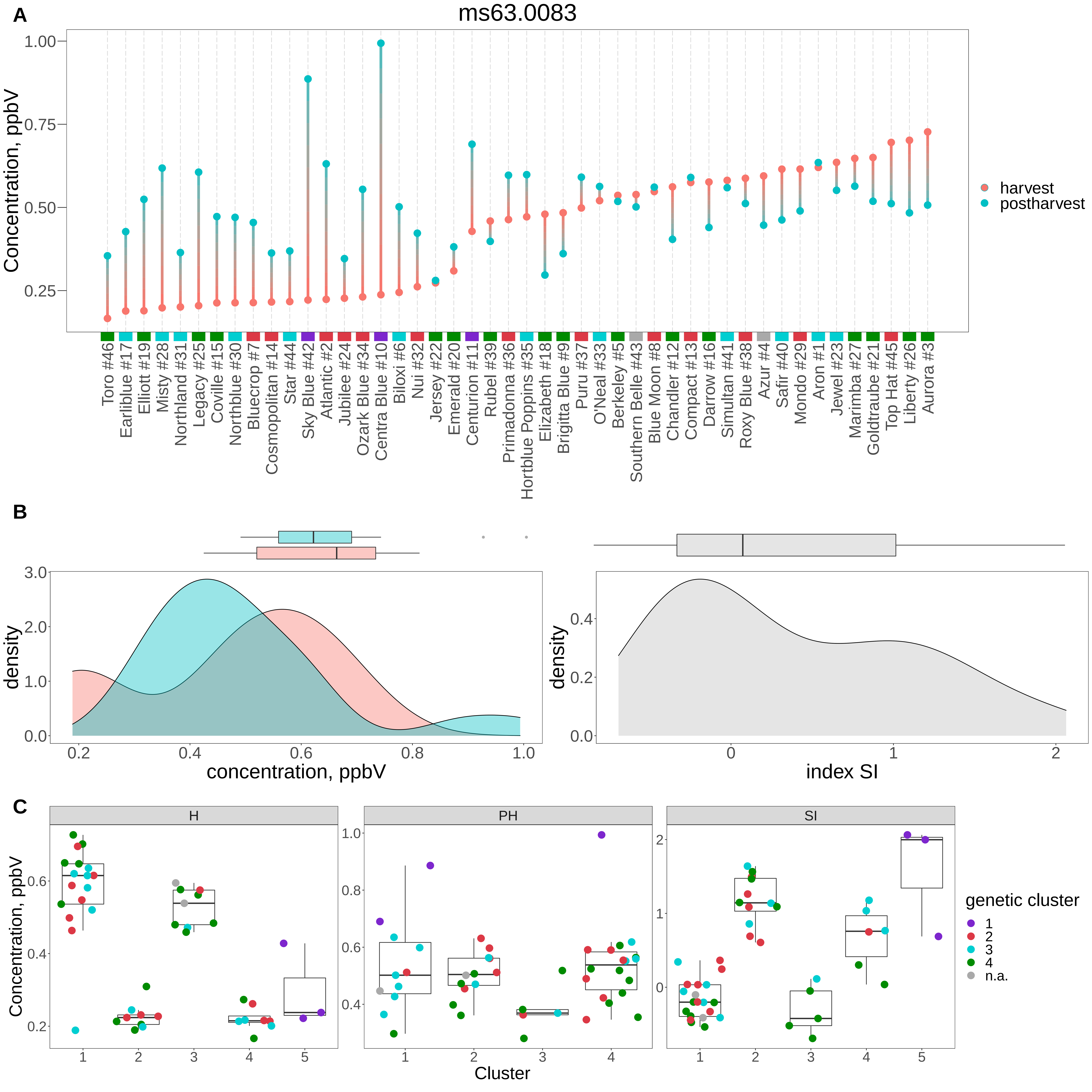

A
ms63.0083
Concentration, ppbV
harvest
postharvest
Toro #46
Earliblue #17
Elliott #19
Misty #28
Northland #31
Legacy #25
Coville #15
Northblue #30
Bluecrop #7
Cosmopolitan #14
Star #44
Sky Blue #42
Atlantic #2
Jubilee #24
Ozark Blue #34
Centra Blue #10
Biloxi #6
Nui #32
Jersey #22
Emerald #20
Centurion #11
Rubel #39
Primadonna #36
Hortblue Poppins #35
Elizabeth #18
Brigitta Blue #9
Puru #37
O'Neal #33
Berkeley #5
Southern Belle #43
Blue Moon #8
Chandler #12
Compact #13
Darrow #16
Simultan #41
Roxy Blue #38
Azur #4
Safir #40
Mondo #29
Aron #1
Jewel #23
Marimba #27
Goldtraube #21
Top Hat #45
Liberty #26
Aurora #3
B
density
concentration, ppbV
index SI
C
H
PH
SI
Concentration, ppbV
Cluster
genetic cluster
1
2
3
4
n.a.

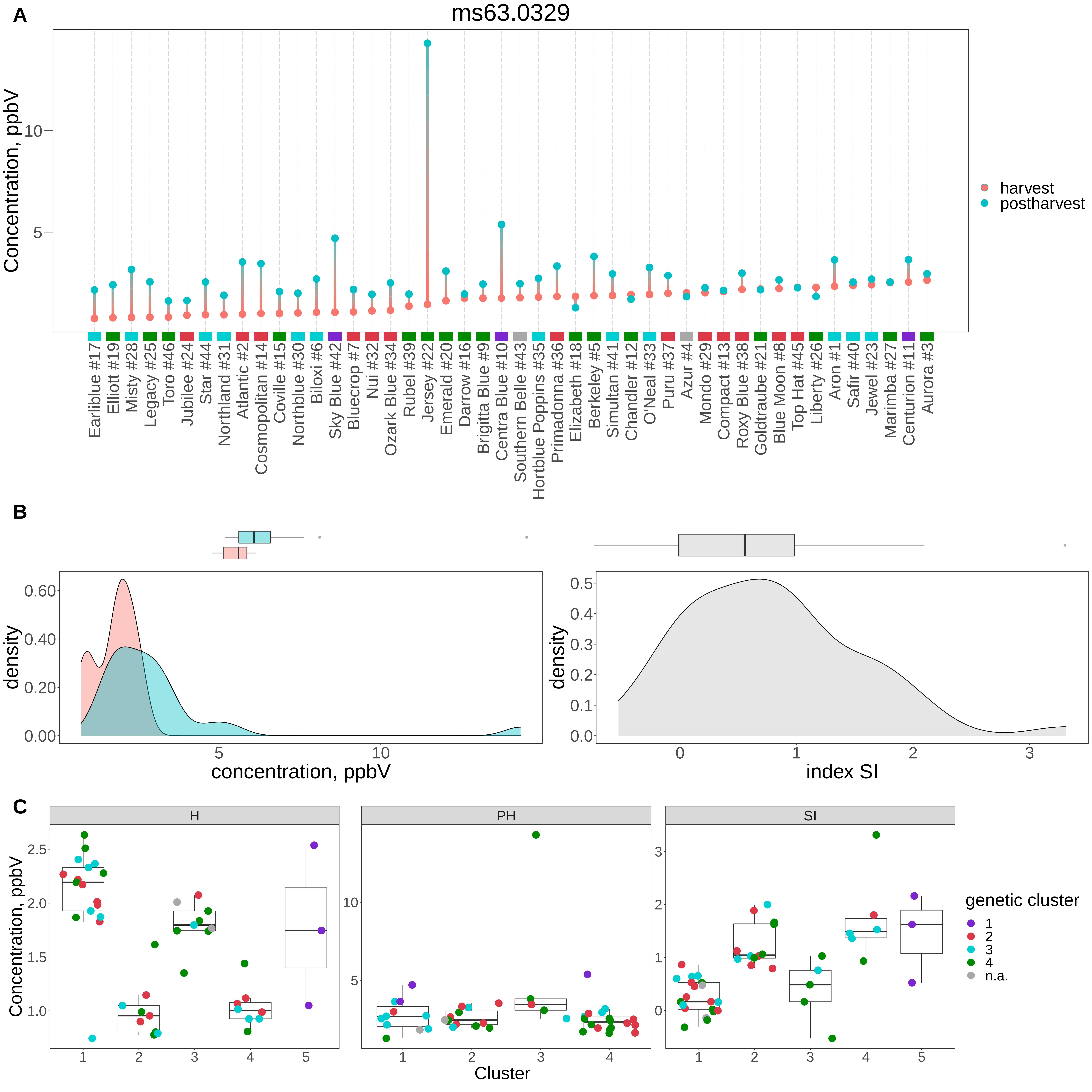

A
ms63.0329
Concentration, ppbV
harvest
postharvest
Earliblue #17
Elliott #19
Misty #28
Legacy #25
Toro #46
Jubilee #24
Star #44
Northland #31
Atlantic #2
Cosmopolitan #14
Coville #15
Northblue #30
Biloxi #6
Sky Blue #42
Bluecrop #7
Nui #32
Ozark Blue #34
Rubel #39
Jersey #22
Emerald #20
Darrow #16
Brigitta Blue #9
Centra Blue #10
Southern Belle #43
Hortblue Poppins #35
Primadonna #36
Elizabeth #18
Berkeley #5
Simultan #41
Chandler #12
O'Neal #33
Puru #37
Azur #4
Mondo #29
Compact #13
Roxy Blue #38
Goldtraube #21
Blue Moon #8
Top Hat #45
Liberty #26
Aron #1
Safir #40
Jewel #23
Marimba #27
Centurion #11
Aurora #3
B
density
concentration, ppbV
index SI
C
H
PH
SI
Concentration, ppbV
Cluster
genetic cluster
1
2
3
4
n.a.

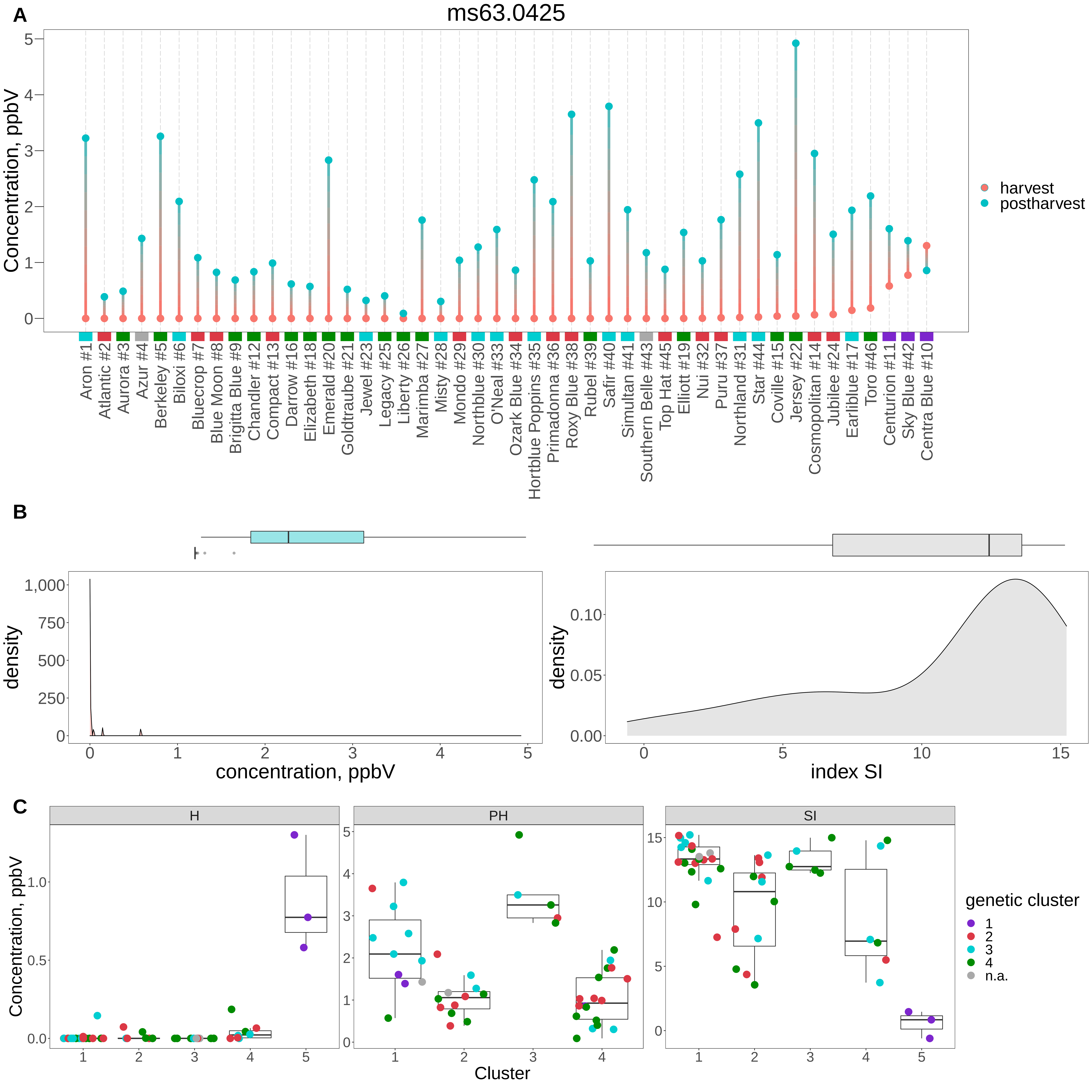

A
ms63.0425
Concentration, ppbV
harvest
postharvest
Aron #1
Atlantic #2
Aurora #3
Azur #4
Berkeley #5
Biloxi #6
Bluecrop #7
Blue Moon #8
Brigitta Blue #9
Chandler #12
Compact #13
Darrow #16
Elizabeth #18
Emerald #20
Goldtraube #21
Jewel #23
Legacy #25
Liberty #26
Marimba #27
Misty #28
Mondo #29
Northblue #30
O'Neal #33
Ozark Blue #34
Hortblue Poppins #35
Primadonna #36
Roxy Blue #38
Rubel #39
Safir #40
Simultan #41
Southern Belle #43
Top Hat #45
Elliott #19
Nui #32
Puru #37
Northland #31
Star #44
Coville #15
Jersey #22
Cosmopolitan #14
Jubilee #24
Earliblue #17
Toro #46
Centurion #11
Sky Blue #42
Centra Blue #10
B
density
concentration, ppbV
index SI
C
H
PH
SI
Concentration, ppbV
Cluster
genetic cluster
n.a.

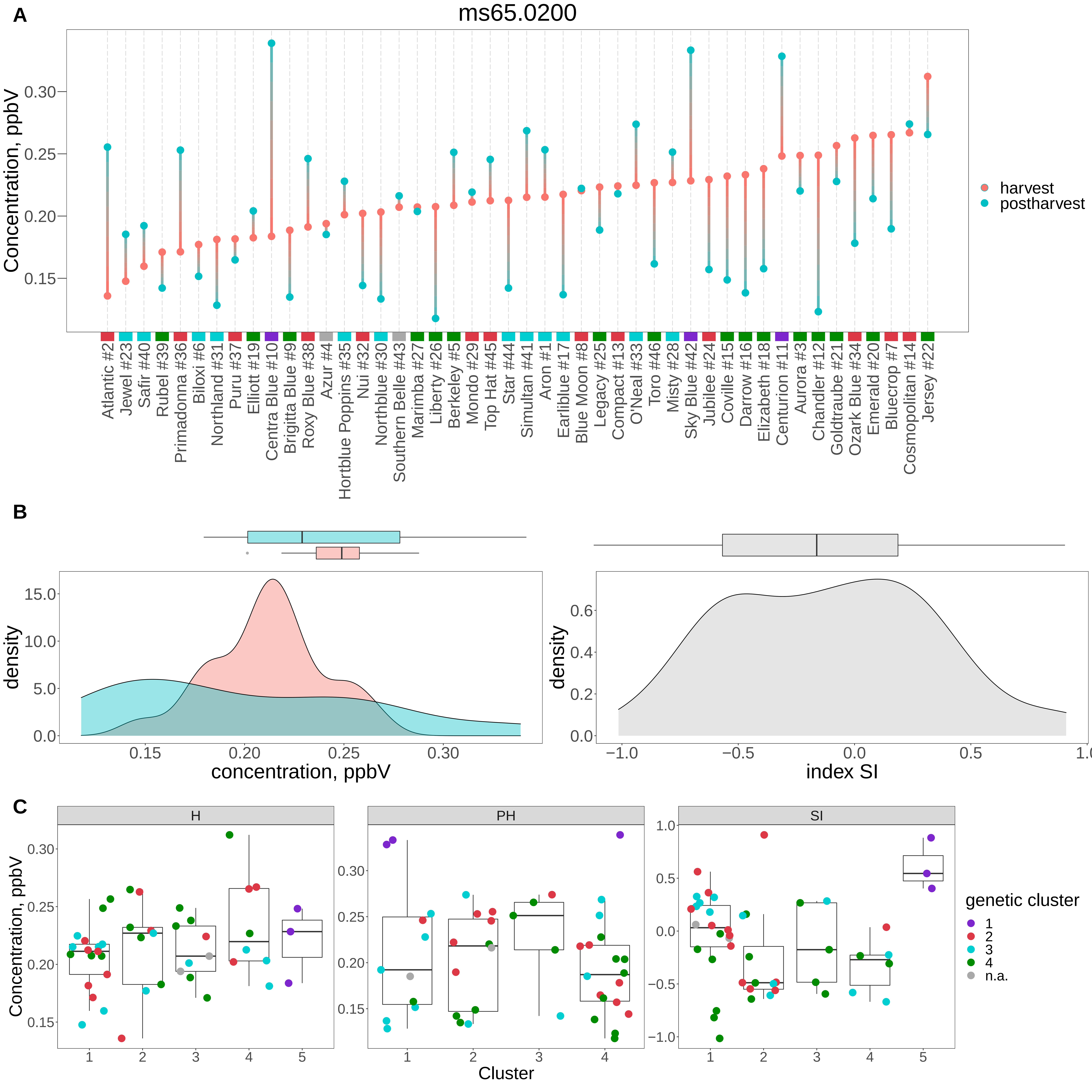

A
ms65.0200
Concentration, ppbV
harvest
postharvest
Atlantic #2
Jewel #23
Safir #40
Rubel #39
Primadonna #36
Biloxi #6
Northland #31
Puru #37
Elliott #19
Centra Blue #10
Brigitta Blue #9
Roxy Blue #38
Azur #4
Hortblue Poppins #35
Nui #32
Northblue #30
Southern Belle #43
Marimba #27
Liberty #26
Berkeley #5
Mondo #29
Top Hat #45
Star #44
Simultan #41
Aron #1
Earliblue #17
Blue Moon #8
Legacy #25
Compact #13
O'Neal #33
Toro #46
Misty #28
Sky Blue #42
Jubilee #24
Coville #15
Darrow #16
Elizabeth #18
Centurion #11
Aurora #3
Chandler #12
Goldtraube #21
Ozark Blue #34
Emerald #20
Bluecrop #7
Cosmopolitan #14
Jersey #22
B
density
concentration, ppbV
index SI
C
H
PH
SI
Cluster
genetic cluster
1
2
3
4
n.a.

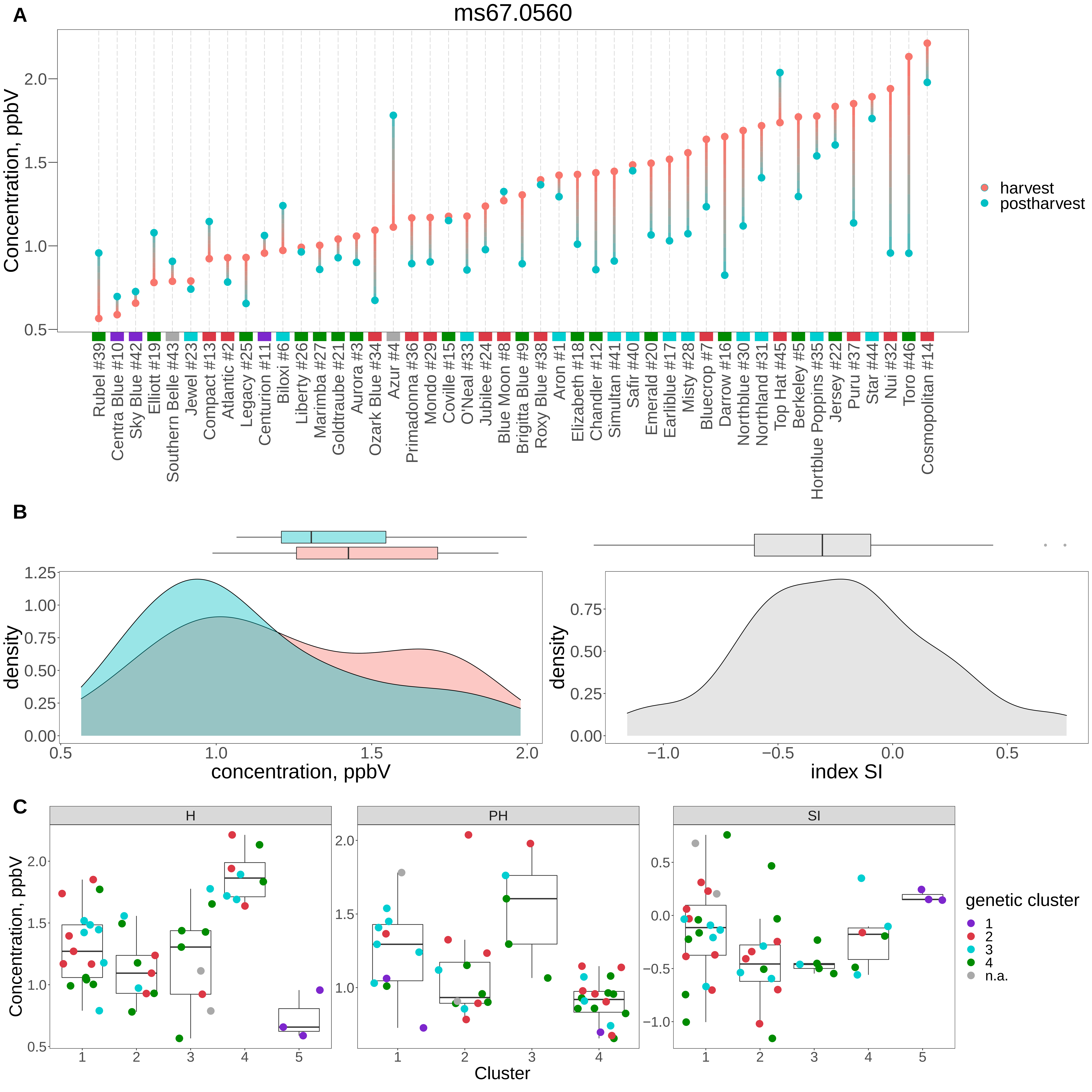
A
ms67.0560
Concentration, ppbV
harvest
postharvest
Rubel #39
Centra Blue #10
Sky Blue #42
Elliott #19
Southern Belle #43
Jewel #23
Compact #13
Atlantic #2
Legacy #25
Centurion #11
Biloxi #6
Liberty #26
Marimba #27
Goldtraube #21
Aurora #3
Ozark Blue #34
Azur #4
Primadonna #36
Mondo #29
Coville #15
O'Neal #33
Jubilee #24
Blue Moon #8
Brigitta Blue #9
Roxy Blue #38
Aron #1
Elizabeth #18
Chandler #12
Simultan #41
Safir #40
Emerald #20
Earliblue #17
Misty #28
Bluecrop #7
Darrow #16
Northblue #30
Northland #31
Top Hat #45
Berkeley #5
Hortblue Poppins #35
Jersey #22
Puru #37
Star #44
Nui #32
Toro #46
Cosmopolitan #14
B
density
concentration, ppbV
index SI
C
H
PH
SI
Concentration, ppbV
Cluster
genetic cluster
1
2
3
4
n.a.

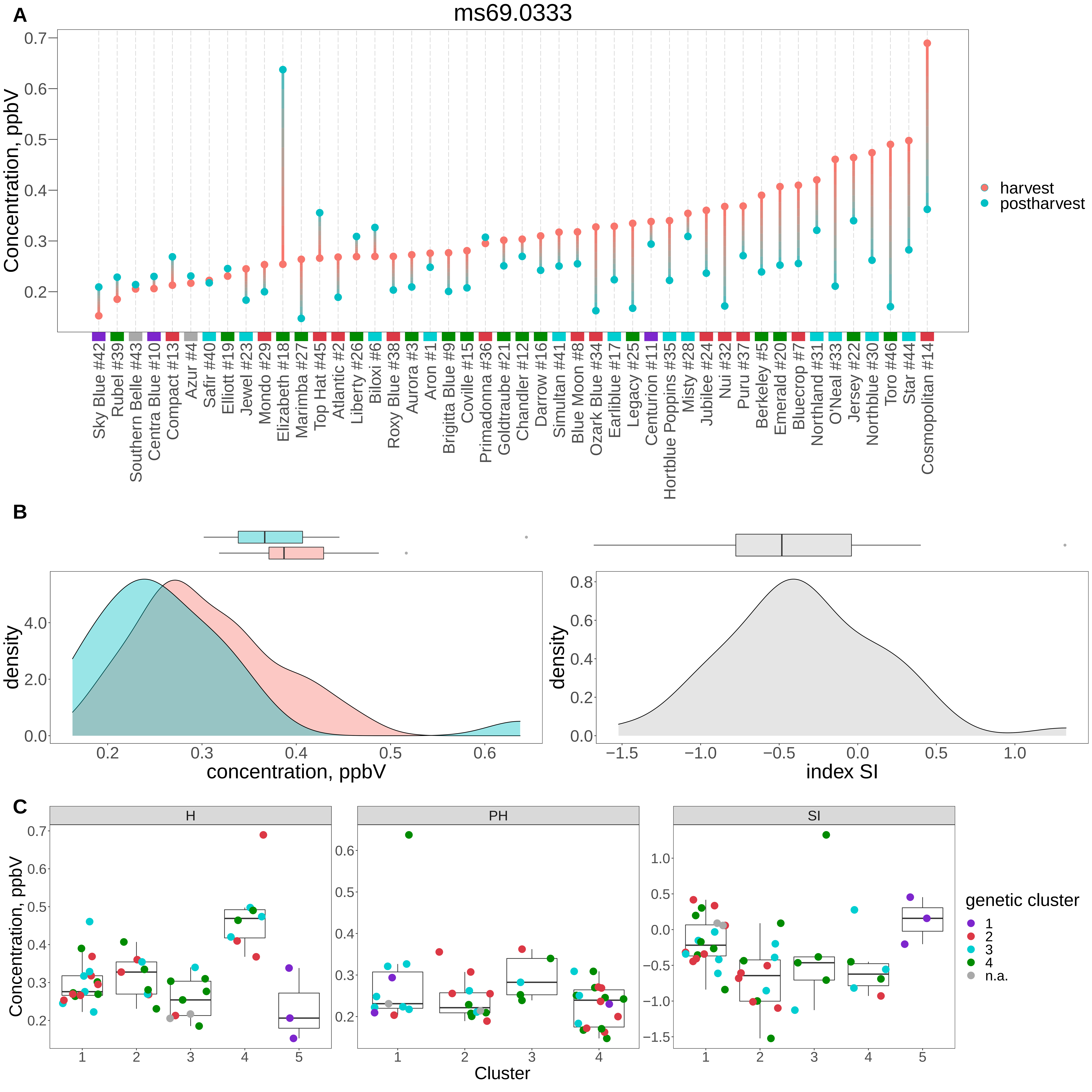
A
ms69.0333
Concentration, ppbV
harvest
postharvest
Sky Blue #42
Rubel #39
Southern Belle #43
Centra Blue #10
Compact #13
Azur #4
Safir #40
Elliott #19
Jewel #23
Mondo #29
Elizabeth #18
Marimba #27
Top Hat #45
Atlantic #2
Liberty #26
Biloxi #6
Roxy Blue #38
Aurora #3
Aron #1
Brigitta Blue #9
Coville #15
Primadonna #36
Goldtraube #21
Chandler #12
Darrow #16
Simultan #41
Blue Moon #8
Ozark Blue #34
Earliblue #17
Legacy #25
Centurion #11
Hortblue Poppins #35
Misty #28
Jubilee #24
Nui #32
Puru #37
Berkeley #5
Emerald #20
Bluecrop #7
Northland #31
O'Neal #33
Jersey #22
Northblue #30
Toro #46
Star #44
Cosmopolitan #14
B
density
concentration, ppbV
index SI
C
H
PH
SI
Concentration, ppbV
Cluster
genetic cluster
1
2
3
4
n.a.

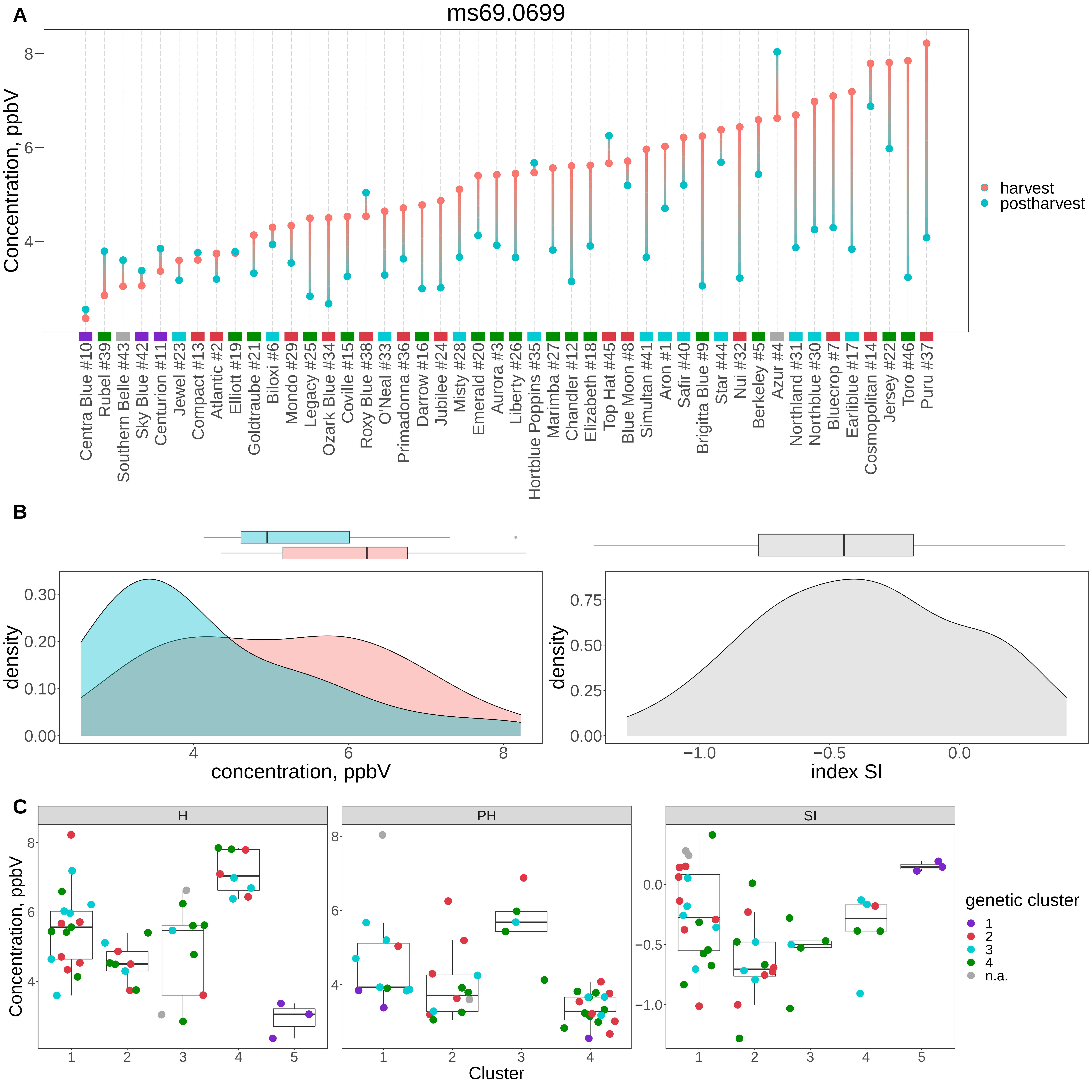

A
ms69.0699
Concentration, ppbV
harvest
postharvest
Centra Blue #10
Rubel #39
Southern Belle #43
Sky Blue #42
Centurion #11
Jewel #23
Compact #13
Atlantic #2
Elliott #19
Goldtraube #21
Biloxi #6
Mondo #29
Legacy #25
Ozark Blue #34
Coville #15
Roxy Blue #38
O'Neal #33
Primadonna #36
Darrow #16
Jubilee #24
Misty #28
Emerald #20
Aurora #3
Liberty #26
Hortblue Poppins #35
Marimba #27
Chandler #12
Elizabeth #18
Top Hat #45
Blue Moon #8
Simultan #41
Aron #1
Safir #40
Brigitta Blue #9
Star #44
Nui #32
Berkeley #5
Azur #4
Northland #31
Northblue #30
Bluecrop #7
Earliblue #17
Cosmopolitan #14
Jersey #22
Toro #46
Puru #37
B
density
concentration, ppbV
index SI
C
H
PH
SI
Cluster
genetic cluster
1
2
3
4
n.a.

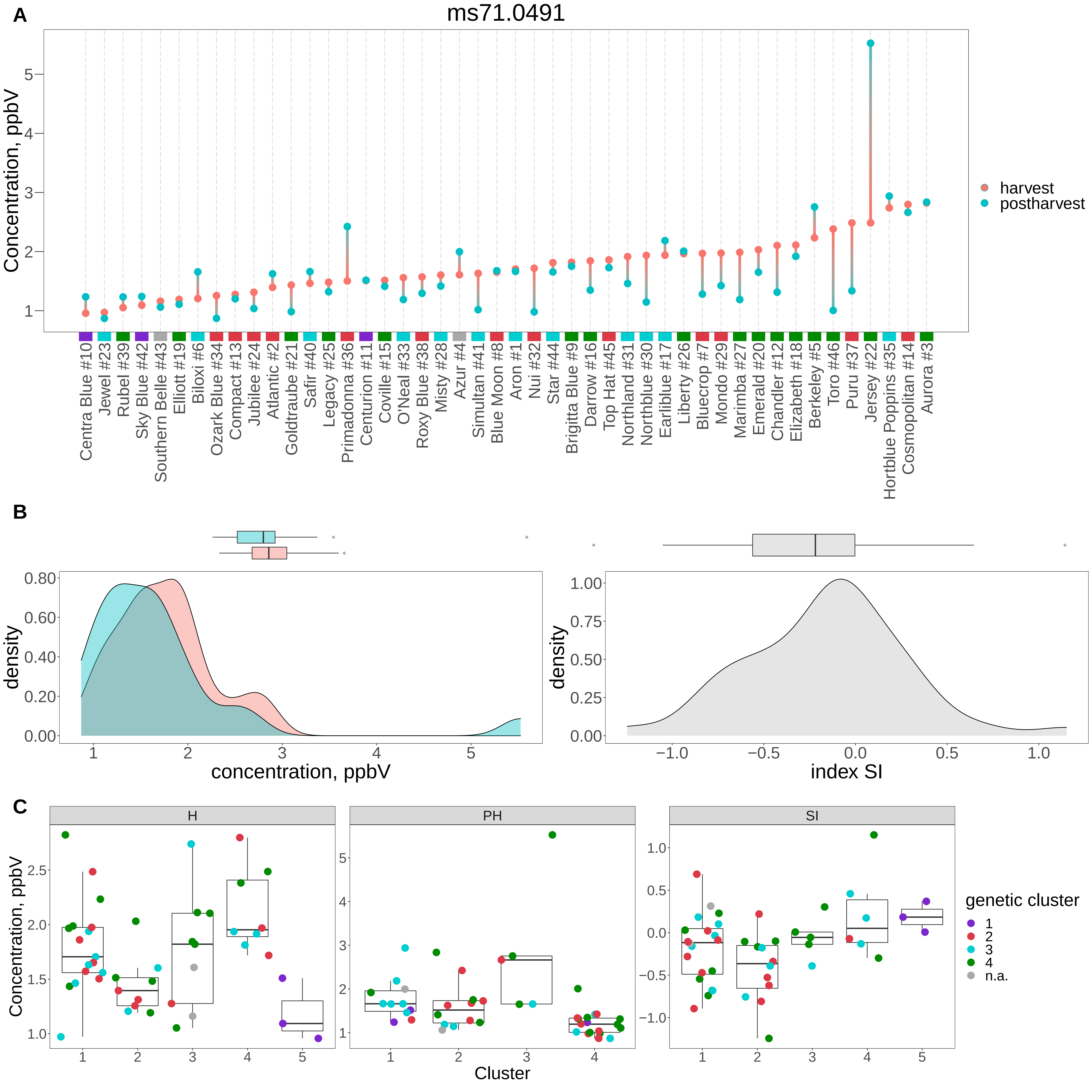
A
ms71.0491
Concentration, ppbV
harvest
postharvest
Centra Blue #10
Jewel #23
Rubel #39
Sky Blue #42
Southern Belle #43
Elliott #19
Biloxi #6
Ozark Blue #34
Compact #13
Jubilee #24
Atlantic #2
Goldtraube #21
Safir #40
Legacy #25
Primadonna #36
Centurion #11
Coville #15
O'Neal #33
Roxy Blue #38
Misty #28
Azur #4
Simultan #41
Blue Moon #8
Aron #1
Nui #32
Star #44
Brigitta Blue #9
Darrow #16
Top Hat #45
Northland #31
Northblue #30
Earliblue #17
Liberty #26
Bluecrop #7
Mondo #29
Marimba #27
Emerald #20
Chandler #12
Elizabeth #18
Berkeley #5
Toro #46
Puru #37
Jersey #22
Hortblue Poppins #35
Cosmopolitan #14
Aurora #3
B
density
concentration, ppbV
index SI
C
H
PH
SI
Concentration, ppbV
Cluster
genetic cluster
1
2
3
4
n.a.

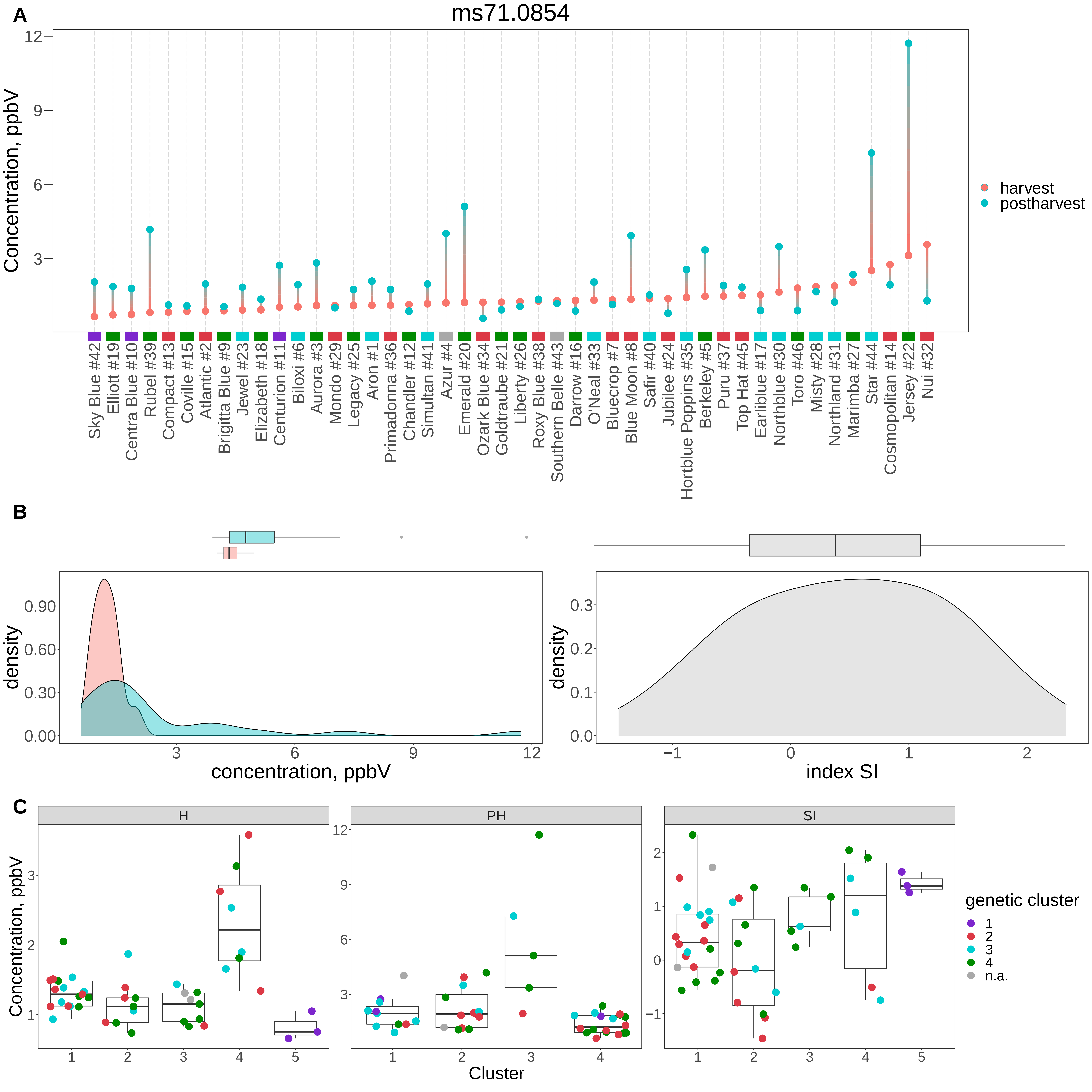

A
ms71.0854
Concentration, ppbV
harvest
postharvest
Sky Blue #42
Elliott #19
Centra Blue #10
Rubel #39
Compact #13
Coville #15
Atlantic #2
Brigitta Blue #9
Jewel #23
Elizabeth #18
Centurion #11
Biloxi #6
Aurora #3
Mondo #29
Legacy #25
Aron #1
Primadonna #36
Chandler #12
Simultan #41
Azur #4
Emerald #20
Ozark Blue #34
Goldtraube #21
Liberty #26
Roxy Blue #38
Southern Belle #43
Darrow #16
O'Neal #33
Bluecrop #7
Blue Moon #8
Safir #40
Jubilee #24
Hortblue Poppins #35
Berkeley #5
Puru #37
Top Hat #45
Earliblue #17
Northblue #30
Toro #46
Misty #28
Northland #31
Marimba #27
Star #44
Cosmopolitan #14
Jersey #22
Nui #32
B
density
concentration, ppbV
index SI
C
H
PH
SI
Concentration, ppbV
Cluster
genetic cluster
1
2
3
4
n.a.

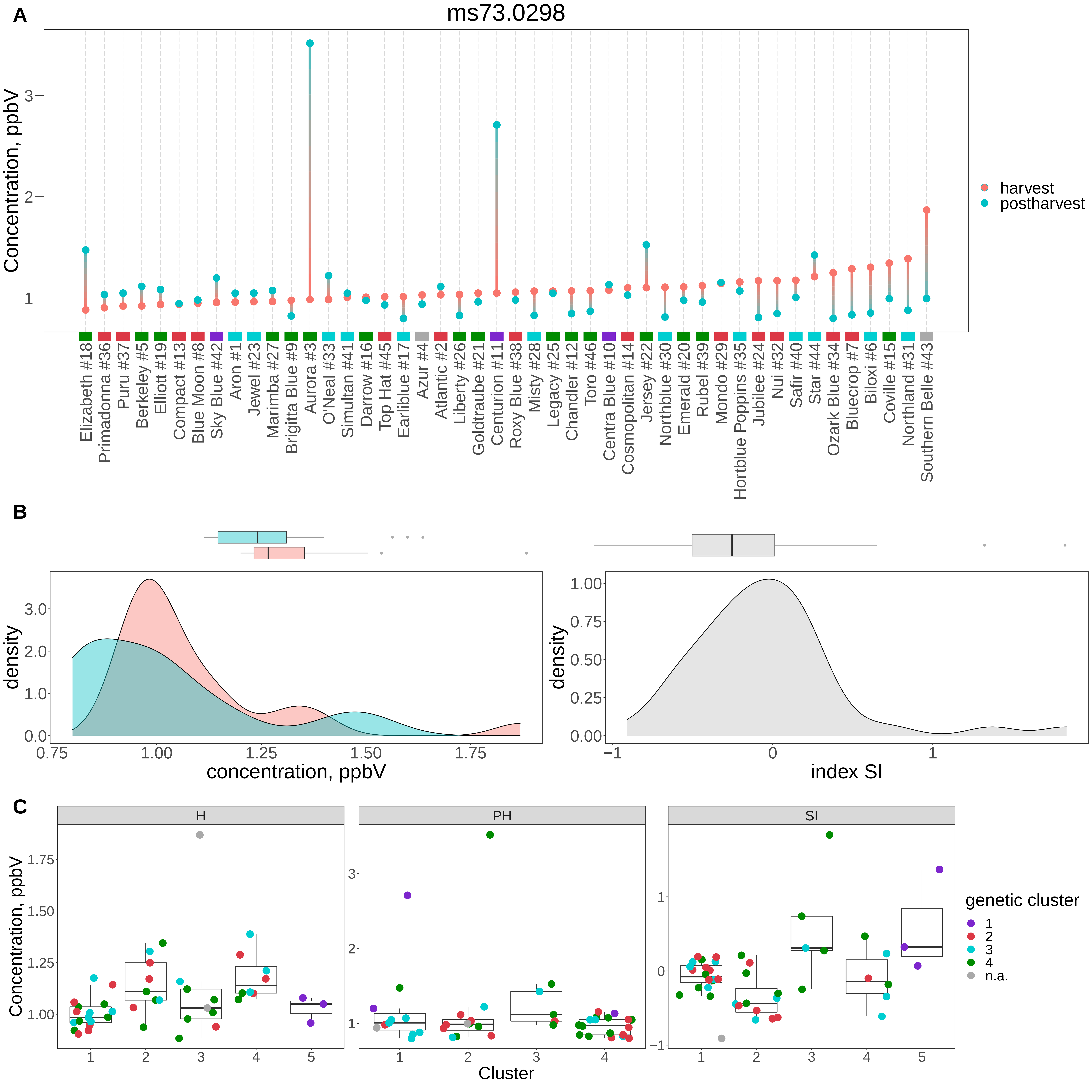
A
ms73.0298
Concentration, ppbV
harvest
postharvest
Elizabeth #18
Primadonna #36
Puru #37
Berkeley #5
Elliott #19
Compact #13
Blue Moon #8
Sky Blue #42
Aron #1
Jewel #23
Marimba #27
Brigitta Blue #9
Aurora #3
O'Neal #33
Simultan #41
Darrow #16
Top Hat #45
Earliblue #17
Azur #4
Atlantic #2
Liberty #26
Goldtraube #21
Centurion #11
Roxy Blue #38
Misty #28
Legacy #25
Chandler #12
Toro #46
Centra Blue #10
Cosmopolitan #14
Jersey #22
Northblue #30
Emerald #20
Rubel #39
Mondo #29
Hortblue Poppins #35
Jubilee #24
Nui #32
Safir #40
Star #44
Ozark Blue #34
Bluecrop #7
Biloxi #6
Coville #15
Northland #31
Southern Belle #43
B
density
concentration, ppbV
index SI
C
H
PH
SI
Cluster
genetic cluster
1
2
3
4
n.a.

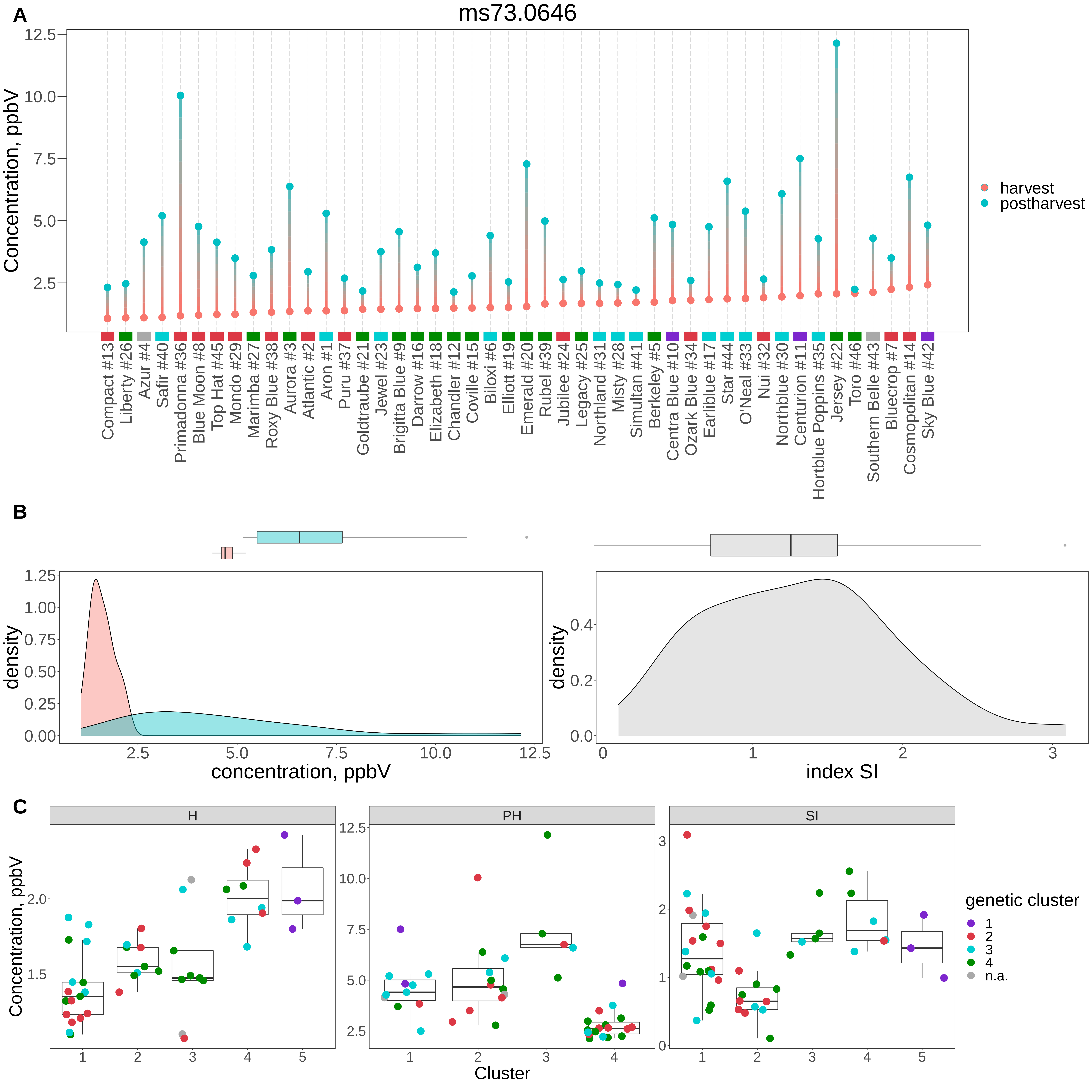
A
ms73.0646
Concentration, ppbV
12.5
10.0
7.5
5.0
2.5
harvest
postharvest
Compact #13
Liberty #26
Azur #4
Safir #40
Primadonna #36
Blue Moon #8
Top Hat #45
Mondo #29
Marimba #27
Roxy Blue #38
Aurora #3
Atlantic #2
Aron #1
Puru #37
Goldtraube #21
Jewel #23
Brigitta Blue #9
Darrow #16
Elizabeth #18
Chandler #12
Coville #15
Biloxi #6
Elliott #19
Emerald #20
Rubel #39
Jubilee #24
Legacy #25
Northland #31
Misty #28
Simultan #41
Berkeley #5
Centra Blue #10
Ozark Blue #34
Earliblue #17
Star #44
O'Neal #33
Nui #32
Northblue #30
Centurion #11
Hortblue Poppins #35
Jersey #22
Toro #46
Southern Belle #43
Bluecrop #7
Cosmopolitan #14
Sky Blue #42
B
density
1.25
1.00
0.75
0.50
0.25
0.00
2.5
5.0
7.5
10.0
12.5
concentration, ppbV
density
0.4
0.2
0.0
0
1
2
3
index SI
C
H
PH
SI
Concentration, ppbV
2.0
1.5
12.5
10.0
7.5
5.0
2.5
3
2
1
0
1
2
3
4
5
Cluster
genetic cluster
1
2
3
4
n.a.

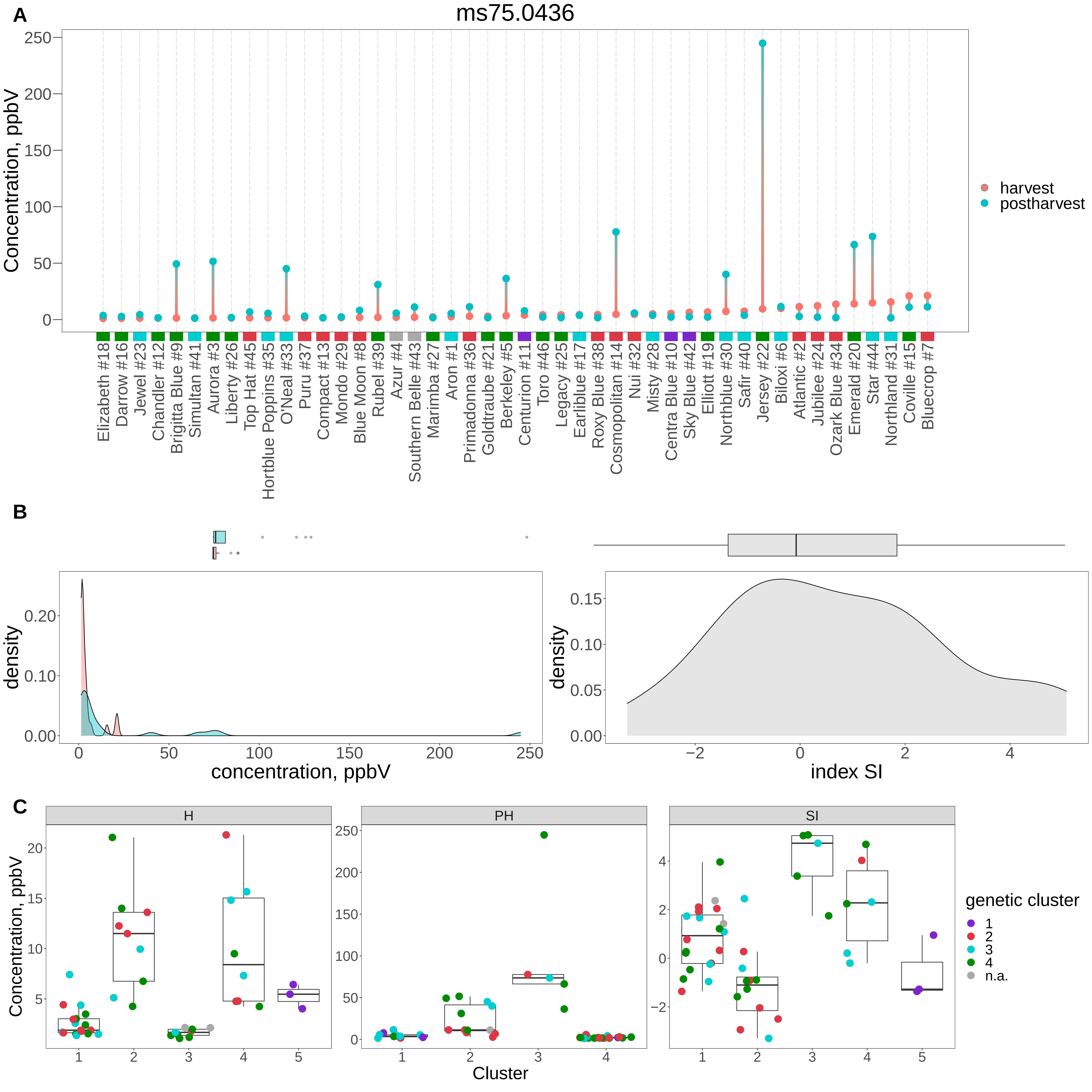
A
ms75.0436
Concentration, ppbV
harvest
postharvest
Elizabeth #18
Darrow #16
Jewel #23
Chandler #12
Brigitta Blue #9
Simultan #41
Aurora #3
Liberty #26
Top Hat #45
Hortblue Poppins #35
O'Neal #33
Puru #37
Compact #13
Mondo #29
Blue Moon #8
Rubel #39
Azur #4
Southern Belle #43
Marimba #27
Aron #1
Primadonna #36
Goldtraube #21
Berkeley #5
Centurion #11
Toro #46
Legacy #25
Earliblue #17
Roxy Blue #38
Cosmopolitan #14
Nui #32
Misty #28
Centra Blue #10
Sky Blue #42
Elliott #19
Northblue #30
Safir #40
Jersey #22
Biloxi #6
Atlantic #2
Jubilee #24
Ozark Blue #34
Emerald #20
Star #44
Northland #31
Coville #15
Bluecrop #7
B
density
concentration, ppbV
index SI
C
H
PH
SI
Concentration, ppbV
Cluster
genetic cluster
1
2
3
4
n.a.

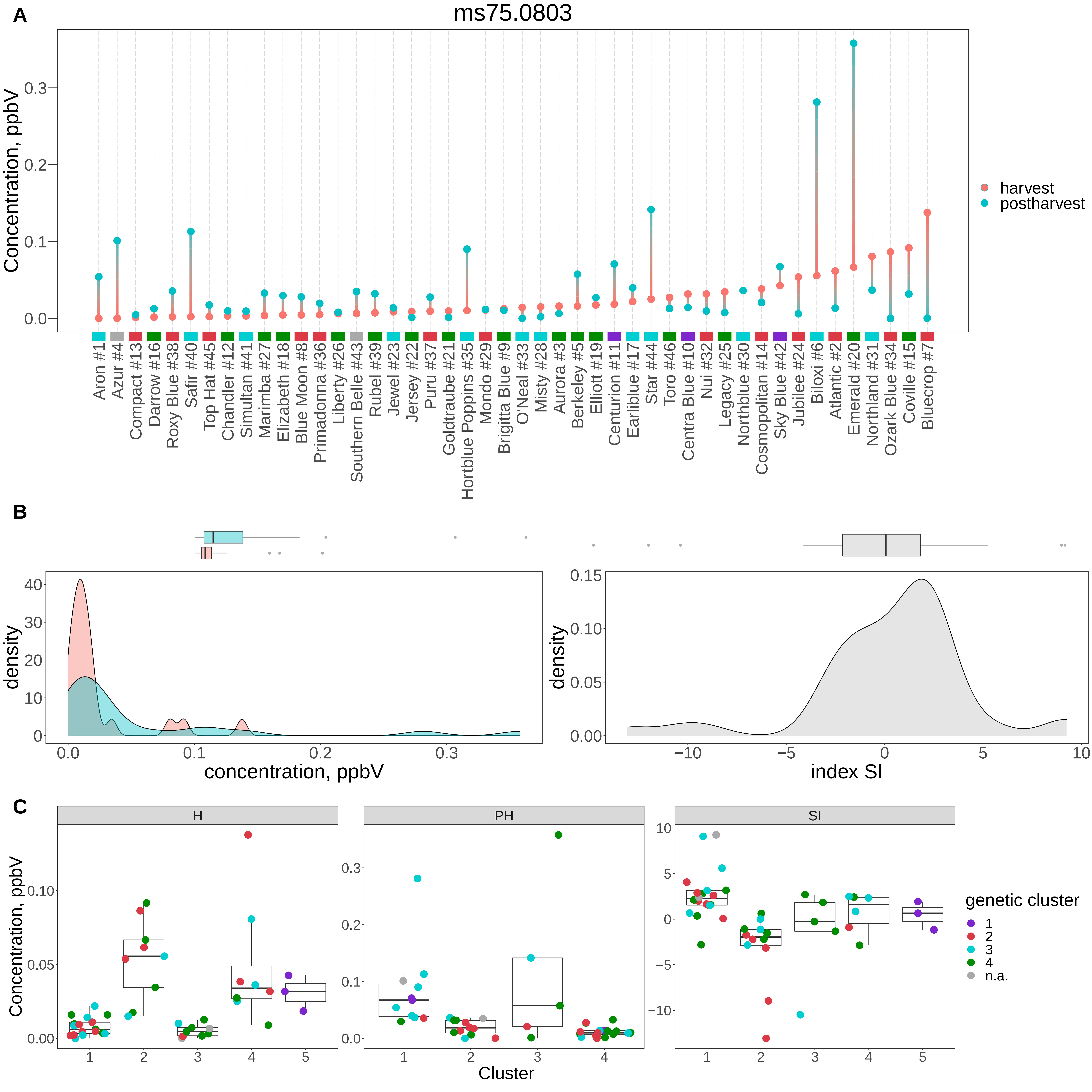
A
ms75.0803
Concentration, ppbV
harvest
postharvest
Aron #1
Azur #4
Compact #13
Darrow #16
Roxy Blue #38
Safir #40
Top Hat #45
Chandler #12
Simultan #41
Marimba #27
Elizabeth #18
Blue Moon #8
Primadonna #36
Liberty #26
Southern Belle #43
Rubel #39
Jewel #23
Jersey #22
Puru #37
Goldtraube #21
Hortblue Poppins #35
Mondo #29
Brigitta Blue #9
O'Neal #33
Misty #28
Aurora #3
Berkeley #5
Elliott #19
Centurion #11
Earliblue #17
Star #44
Toro #46
Centra Blue #10
Nui #32
Legacy #25
Northblue #30
Cosmopolitan #14
Sky Blue #42
Jubilee #24
Biloxi #6
Atlantic #2
Emerald #20
Northland #31
Ozark Blue #34
Coville #15
Bluecrop #7
B
density
concentration, ppbV
index SI
C
H
PH
SI
Concentration, ppbV
Cluster
genetic cluster
1
2
3
4
n.a.

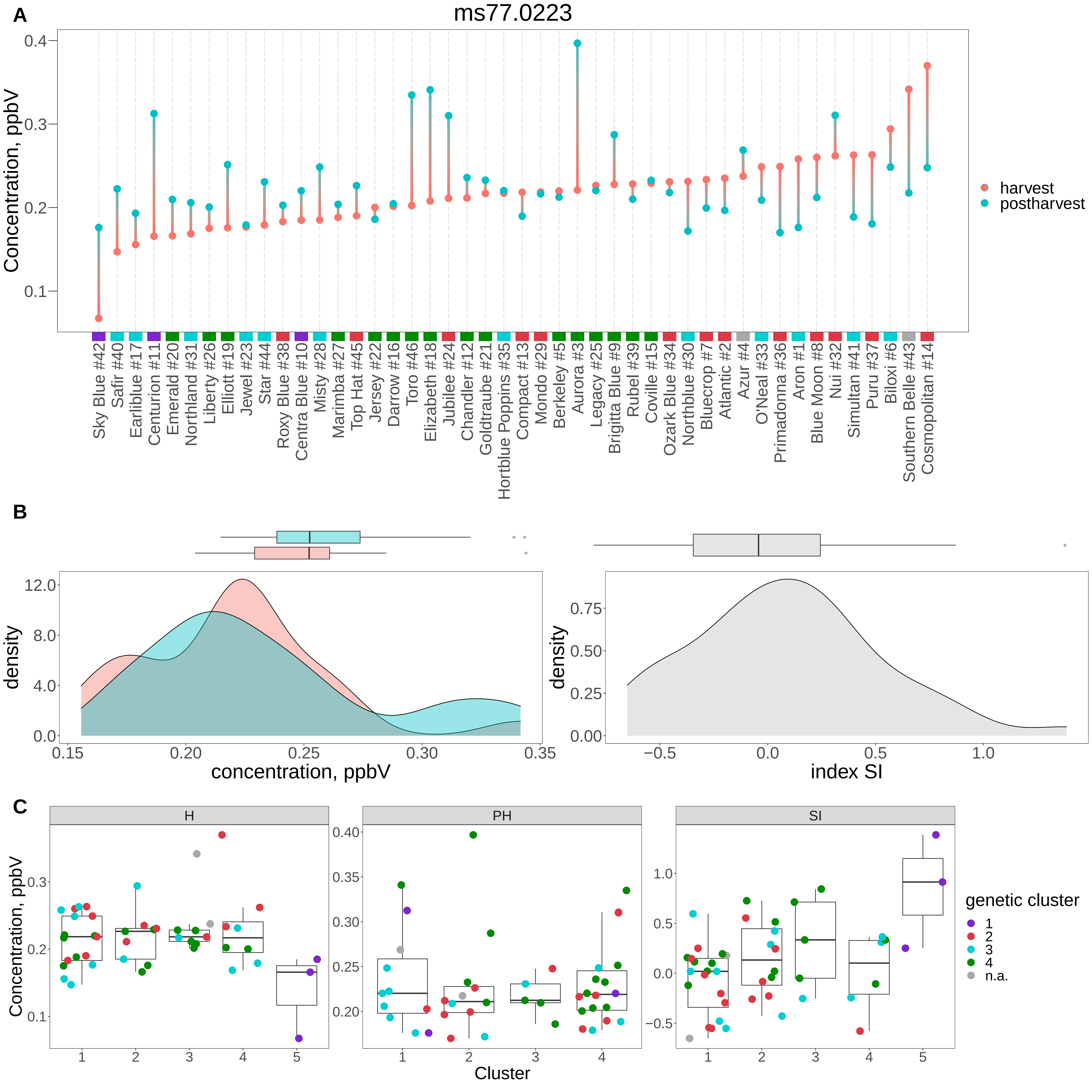
A
ms77.0223
Concentration, ppbV
harvest
postharvest
Sky Blue #42
Safir #40
Earliblue #17
Centurion #11
Emerald #20
Northland #31
Liberty #26
Elliott #19
Jewel #23
Star #44
Roxy Blue #38
Centra Blue #10
Misty #28
Marimba #27
Top Hat #45
Jersey #22
Darrow #16
Toro #46
Elizabeth #18
Jubilee #24
Chandler #12
Goldtraube #21
Hortblue Poppins #35
Compact #13
Mondo #29
Berkeley #5
Aurora #3
Legacy #25
Brigitta Blue #9
Rubel #39
Coville #15
Ozark Blue #34
Northblue #30
Bluecrop #7
Atlantic #2
Azur #4
O'Neal #33
Primadonna #36
Aron #1
Blue Moon #8
Nui #32
Simultan #41
Puru #37
Biloxi #6
Southern Belle #43
Cosmopolitan #14
B
density
concentration, ppbV
index SI
C
H
PH
SI
Concentration, ppbV
Cluster
genetic cluster
1
2
3
4
n.a.

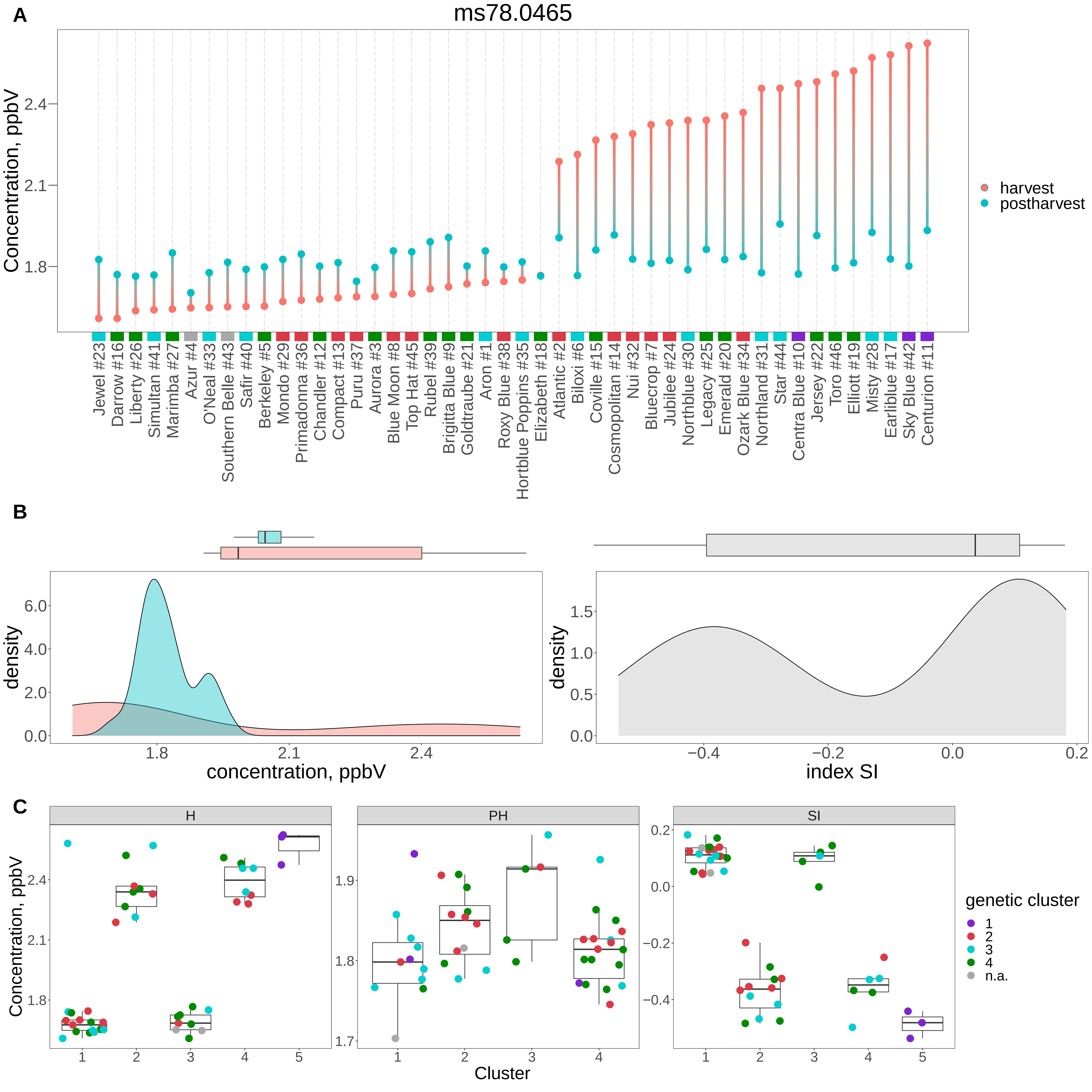
A
ms78.0465
Concentration, ppbV
2.4
2.1
1.8
harvest
postharvest
Jewel #23
Darrow #16
Liberty #26
Simultan #41
Marimba #27
Azur #4
O'Neal #33
Southern Belle #43
Safir #40
Berkeley #5
Mondo #29
Primadonna #36
Chandler #12
Compact #13
Puru #37
Aurora #3
Blue Moon #8
Top Hat #45
Rubel #39
Brigitta Blue #9
Goldtraube #21
Aron #1
Roxy Blue #38
Hortblue Poppins #35
Elizabeth #18
Atlantic #2
Biloxi #6
Coville #15
Cosmopolitan #14
Nui #32
Bluecrop #7
Jubilee #24
Northblue #30
Legacy #25
Emerald #20
Ozark Blue #34
Northland #31
Star #44
Centra Blue #10
Jersey #22
Toro #46
Elliott #19
Misty #28
Earliblue #17
Sky Blue #42
Centurion #11
B
density
6.0
4.0
2.0
0.0
1.8
2.1
2.4
concentration, ppbV
density
1.5
1.0
0.5
0.0
−0.4
−0.2
0.0
0.2
index SI
C
H
PH
SI
Concentration, ppbV
Cluster
genetic cluster
1
2
3
4
n.a.

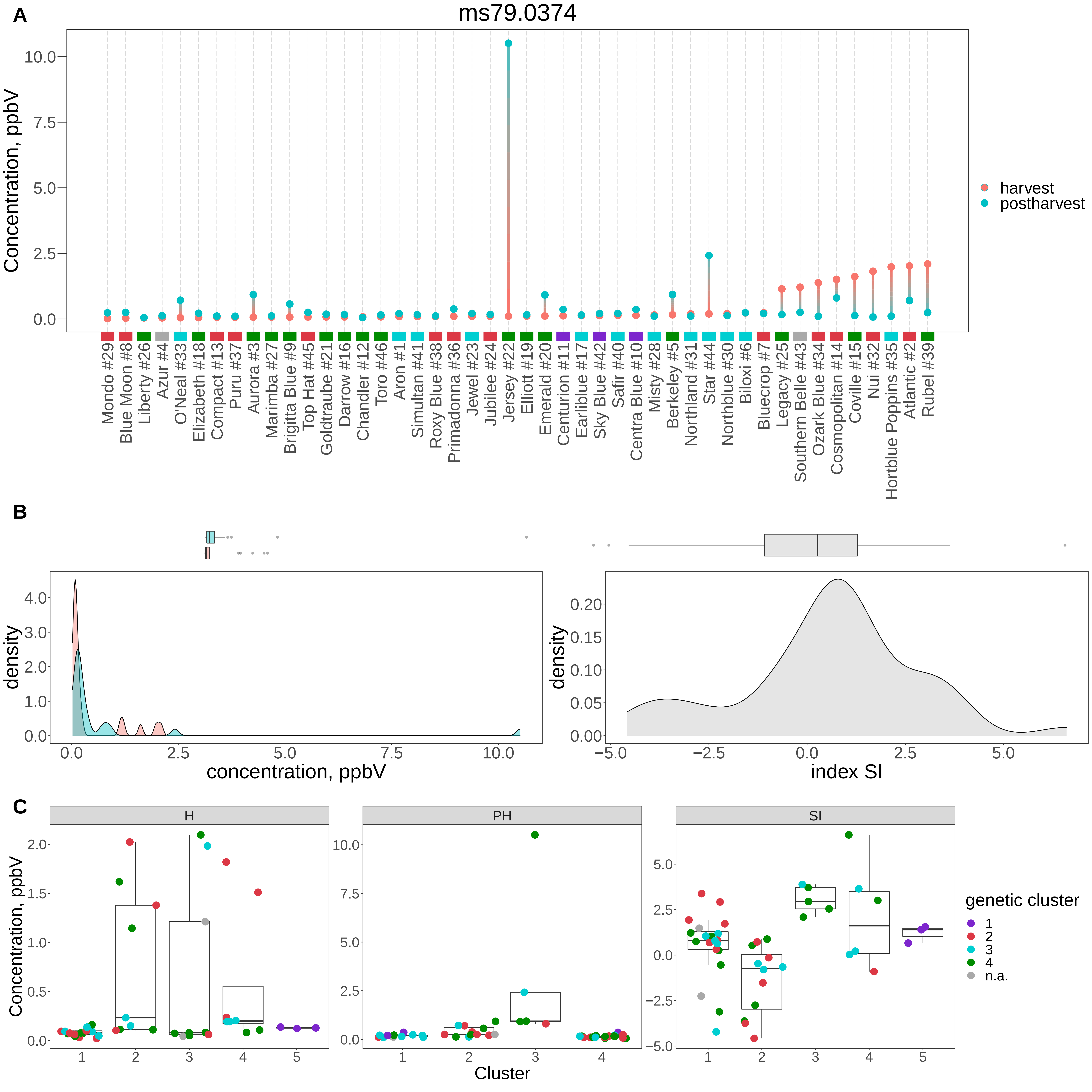

A
ms79.0374
Concentration, ppbV
harvest
postharvest
Mondo #29
Blue Moon #8
Liberty #26
Azur #4
O'Neal #33
Elizabeth #18
Compact #13
Puru #37
Aurora #3
Marimba #27
Brigitta Blue #9
Top Hat #45
Goldtraube #21
Darrow #16
Chandler #12
Toro #46
Aron #1
Simultan #41
Roxy Blue #38
Primadonna #36
Jewel #23
Jubilee #24
Jersey #22
Elliott #19
Emerald #20
Centurion #11
Earliblue #17
Sky Blue #42
Safir #40
Centra Blue #10
Misty #28
Berkeley #5
Northland #31
Star #44
Northblue #30
Biloxi #6
Bluecrop #7
Legacy #25
Southern Belle #43
Ozark Blue #34
Cosmopolitan #14
Coville #15
Nui #32
Hortblue Poppins #35
Atlantic #2
Rubel #39
B
density
concentration, ppbV
index SI
C
H
PH
SI
Concentration, ppbV
Cluster
genetic cluster
1
2
3
4
n.a.

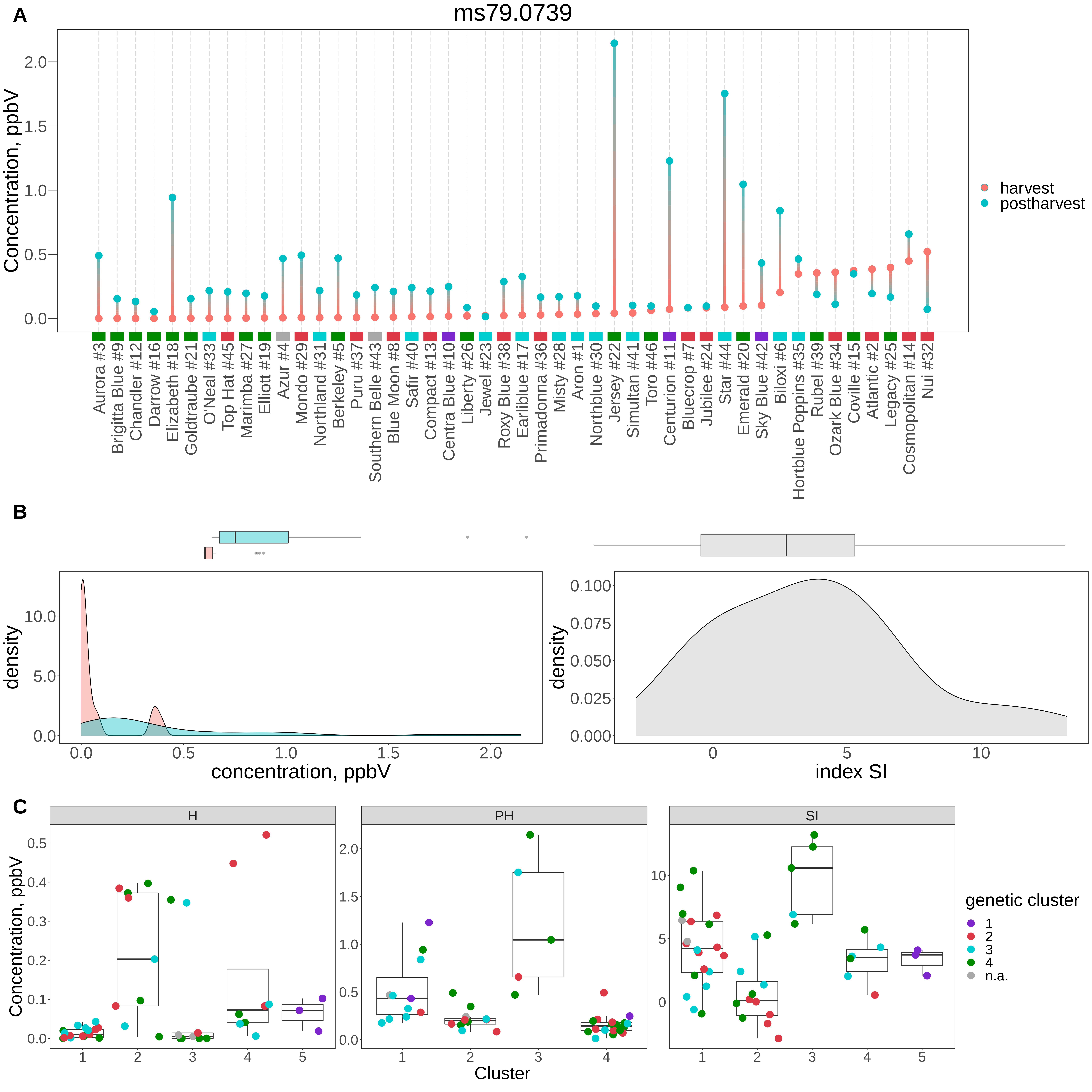

A
ms79.0739
Concentration, ppbV
harvest
postharvest
Aurora #3
Brigitta Blue #9
Chandler #12
Darrow #16
Elizabeth #18
Goldtraube #21
O'Neal #33
Top Hat #45
Marimba #27
Elliott #19
Azur #4
Mondo #29
Northland #31
Berkeley #5
Puru #37
Southern Belle #43
Blue Moon #8
Safir #40
Compact #13
Centra Blue #10
Liberty #26
Jewel #23
Roxy Blue #38
Earliblue #17
Primadonna #36
Misty #28
Aron #1
Northblue #30
Jersey #22
Simultan #41
Toro #46
Centurion #11
Bluecrop #7
Jubilee #24
Star #44
Emerald #20
Sky Blue #42
Biloxi #6
Hortblue Poppins #35
Rubel #39
Ozark Blue #34
Coville #15
Atlantic #2
Legacy #25
Cosmopolitan #14
Nui #32
B
density
concentration, ppbV
index SI
C
H
PH
SI
Cluster
genetic cluster
1
2
3
4
n.a.

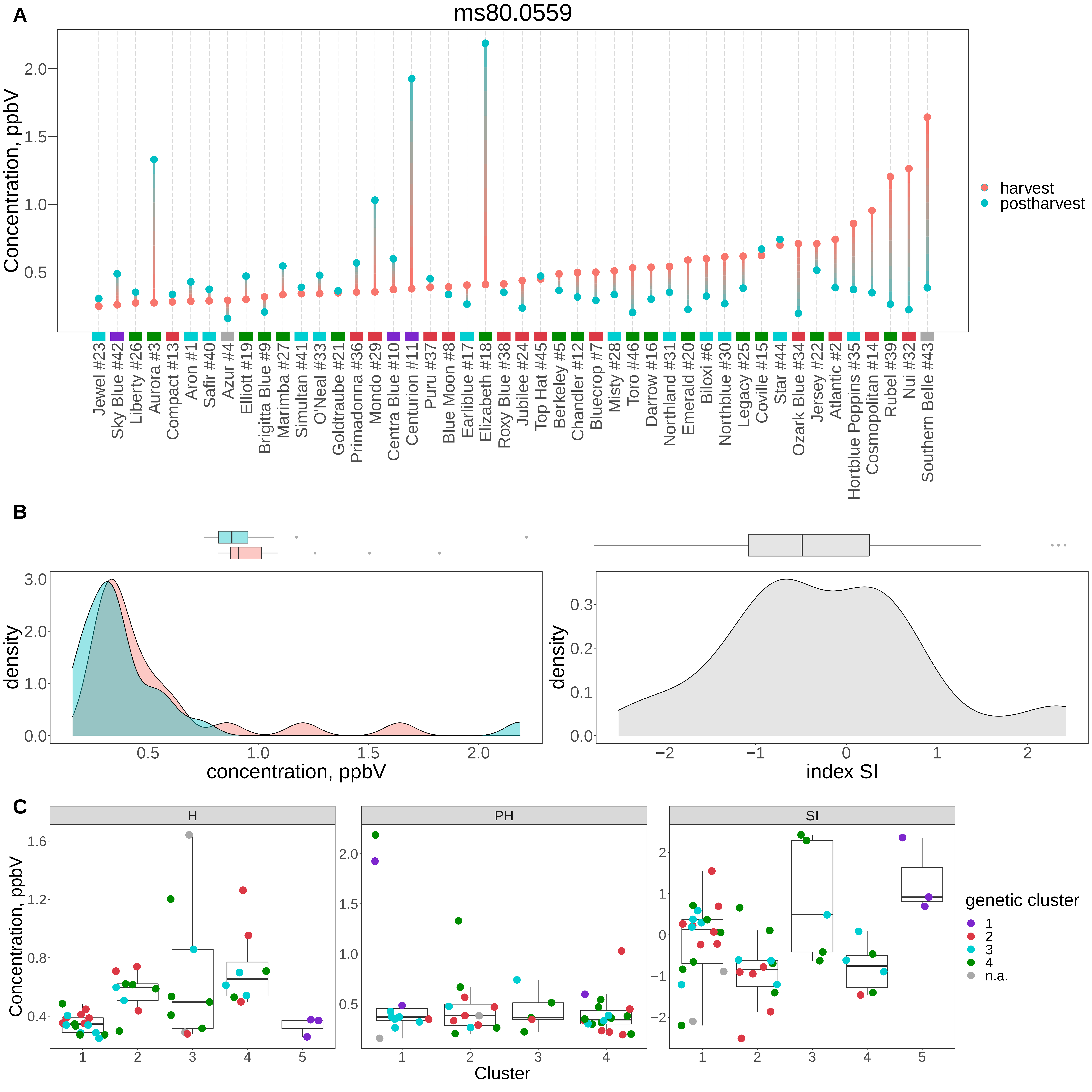
A
ms80.0559
Concentration, ppbV
0.5
1.0
1.5
2.0
harvest
postharvest
Jewel #23
Sky Blue #42
Liberty #26
Aurora #3
Compact #13
Aron #1
Safir #40
Azur #4
Elliott #19
Brigitta Blue #9
Marimba #27
Simultan #41
O'Neal #33
Goldtraube #21
Primadonna #36
Mondo #29
Centra Blue #10
Centurion #11
Puru #37
Blue Moon #8
Earliblue #17
Elizabeth #18
Roxy Blue #38
Jubilee #24
Top Hat #45
Berkeley #5
Chandler #12
Bluecrop #7
Misty #28
Toro #46
Darrow #16
Northland #31
Emerald #20
Biloxi #6
Northblue #30
Legacy #25
Coville #15
Star #44
Ozark Blue #34
Jersey #22
Atlantic #2
Hortblue Poppins #35
Cosmopolitan #14
Rubel #39
Nui #32
Southern Belle #43
B
density
0.0
1.0
2.0
3.0
0.5
1.0
1.5
2.0
concentration, ppbV
density
0.0
0.1
0.2
0.3
−2
−1
0
1
2
index SI
C
H
PH
SI
Concentration, ppbV
0.4
0.8
1.2
1.6
0.5
1.0
1.5
2.0
−2
−1
0
1
2
1
2
3
4
5
Cluster
genetic cluster
1
2
3
4
n.a.

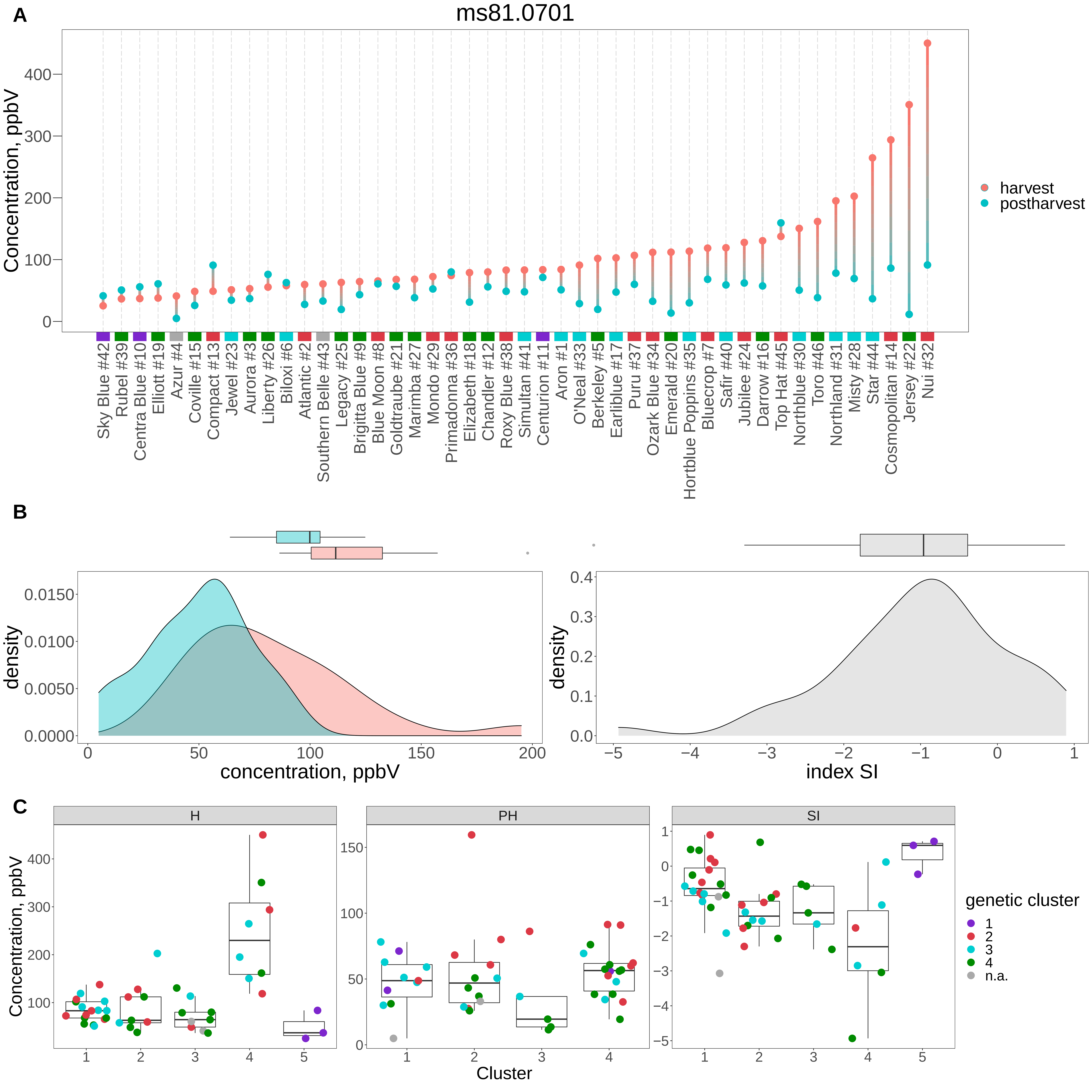
A
ms81.0701
Concentration, ppbV
harvest
postharvest
Sky Blue #42
Rubel #39
Centra Blue #10
Elliott #19
Azur #4
Coville #15
Compact #13
Jewel #23
Aurora #3
Liberty #26
Biloxi #6
Atlantic #2
Southern Belle #43
Legacy #25
Brigitta Blue #9
Blue Moon #8
Goldtraube #21
Marimba #27
Mondo #29
Primadonna #36
Elizabeth #18
Chandler #12
Roxy Blue #38
Simultan #41
Centurion #11
Aron #1
O'Neal #33
Berkeley #5
Earliblue #17
Puru #37
Ozark Blue #34
Emerald #20
Hortblue Poppins #35
Bluecrop #7
Safir #40
Jubilee #24
Darrow #16
Top Hat #45
Northblue #30
Toro #46
Northland #31
Misty #28
Star #44
Cosmopolitan #14
Jersey #22
Nui #32
B
density
concentration, ppbV
density
index SI
C
H
PH
SI
Concentration, ppbV
Cluster
genetic cluster
1
2
3
4
n.a.

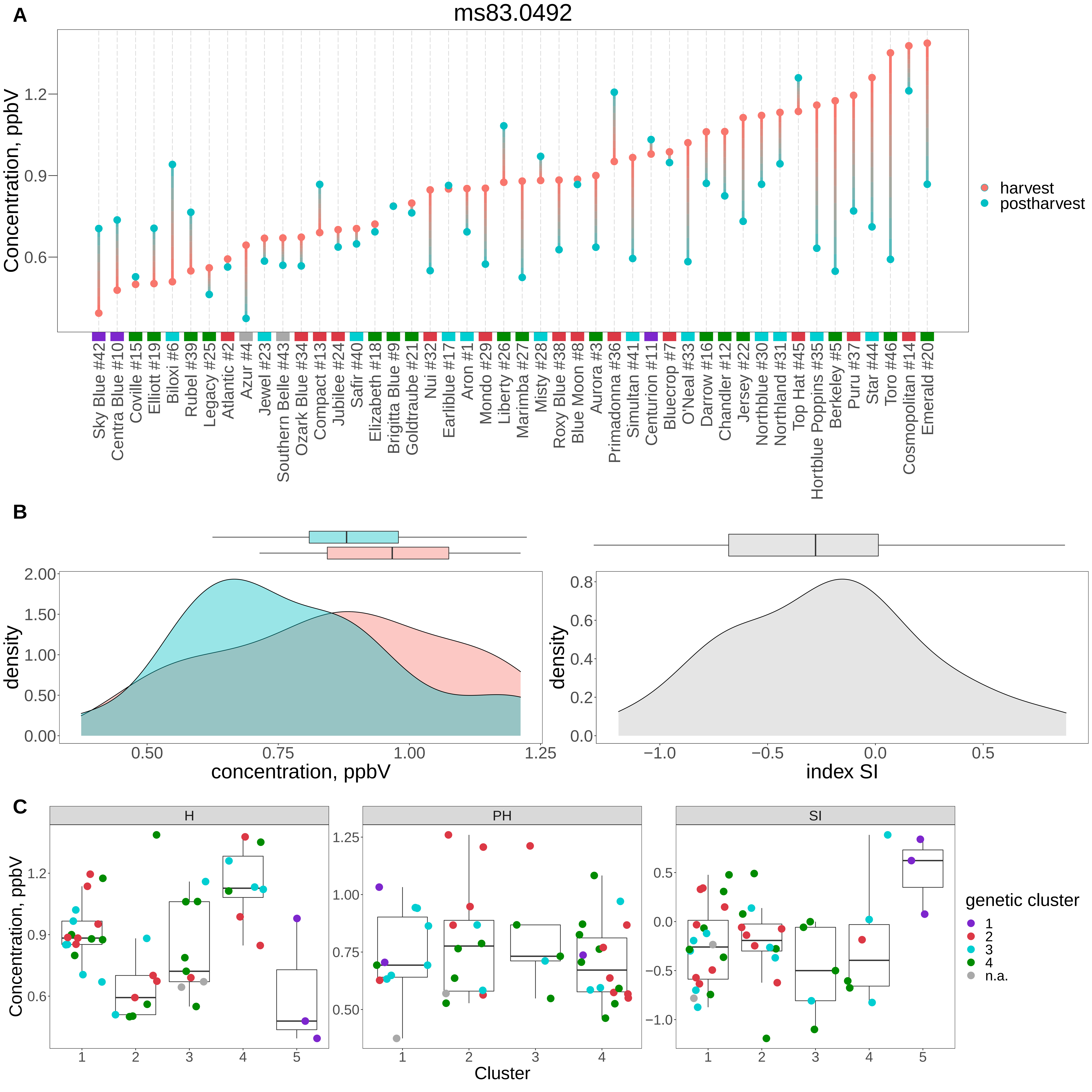

A
ms83.0492
Concentration, ppbV
harvest
postharvest
Sky Blue #42
Centra Blue #10
Coville #15
Elliott #19
Biloxi #6
Rubel #39
Legacy #25
Atlantic #2
Azur #4
Jewel #23
Southern Belle #43
Ozark Blue #34
Compact #13
Jubilee #24
Safir #40
Elizabeth #18
Brigitta Blue #9
Goldtraube #21
Nui #32
Earliblue #17
Aron #1
Mondo #29
Liberty #26
Marimba #27
Misty #28
Roxy Blue #38
Blue Moon #8
Aurora #3
Primadonna #36
Simultan #41
Centurion #11
Bluecrop #7
O'Neal #33
Darrow #16
Chandler #12
Jersey #22
Northblue #30
Northland #31
Top Hat #45
Hortblue Poppins #35
Berkeley #5
Puru #37
Star #44
Toro #46
Cosmopolitan #14
Emerald #20
B
density
concentration, ppbV
index SI
C
H
PH
SI
Concentration, ppbV
Cluster
genetic cluster
1
2
3
4
n.a.

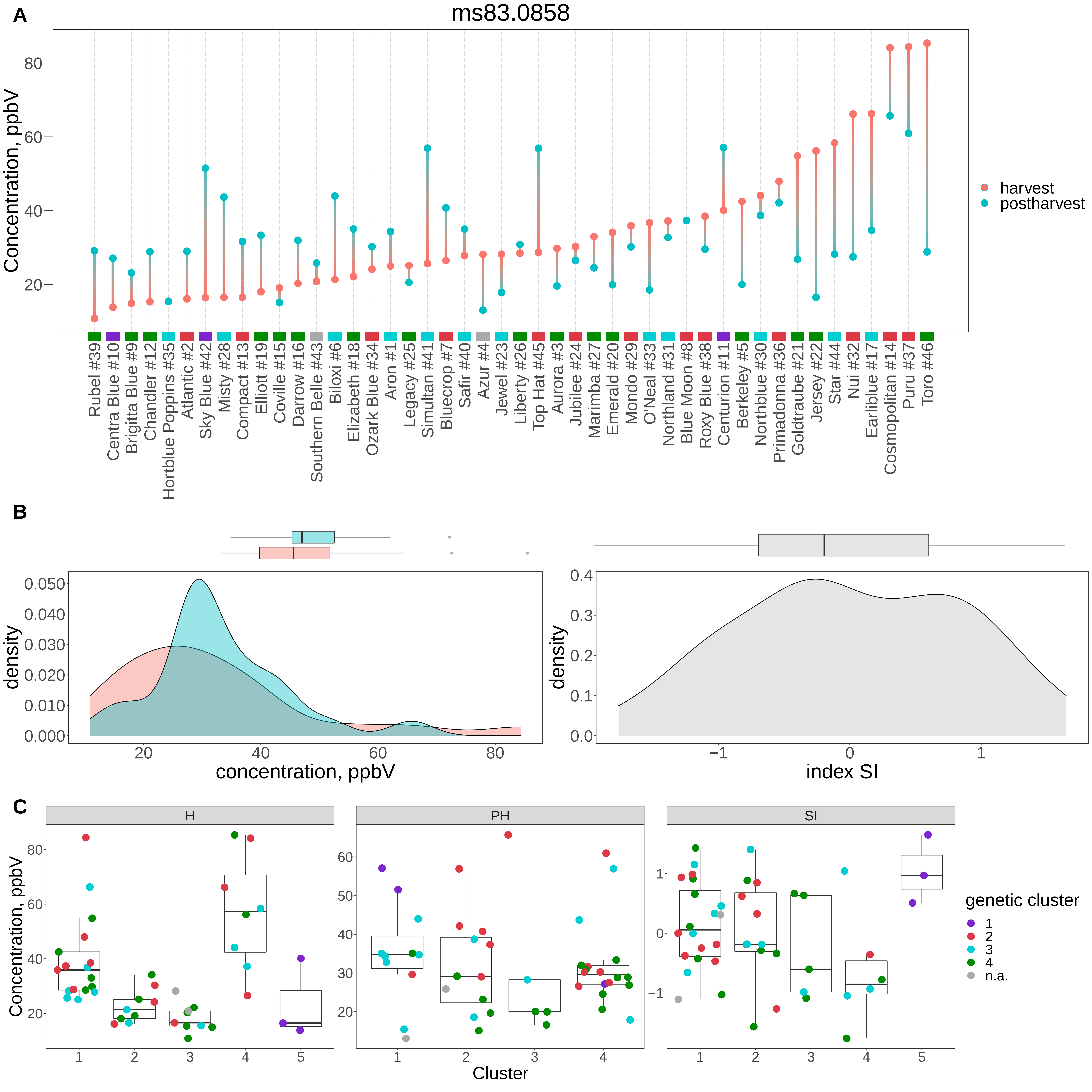
A
ms83.0858
Concentration, ppbV
harvest
postharvest
Rubel #39
Centra Blue #10
Brigitta Blue #9
Chandler #12
Hortblue Poppins #35
Atlantic #2
Sky Blue #42
Misty #28
Compact #13
Elliott #19
Coville #15
Darrow #16
Southern Belle #43
Biloxi #6
Elizabeth #18
Ozark Blue #34
Aron #1
Legacy #25
Simultan #41
Bluecrop #7
Safir #40
Azur #4
Jewel #23
Liberty #26
Top Hat #45
Aurora #3
Jubilee #24
Marimba #27
Emerald #20
Mondo #29
O'Neal #33
Northland #31
Blue Moon #8
Roxy Blue #38
Centurion #11
Berkeley #5
Northblue #30
Primadonna #36
Goldtraube #21
Jersey #22
Star #44
Nui #32
Earliblue #17
Cosmopolitan #14
Puru #37
Toro #46
B
density
concentration, ppbV
index SI
C
H
PH
SI
Concentration, ppbV
Cluster
genetic cluster
1
2
3
4
n.a.

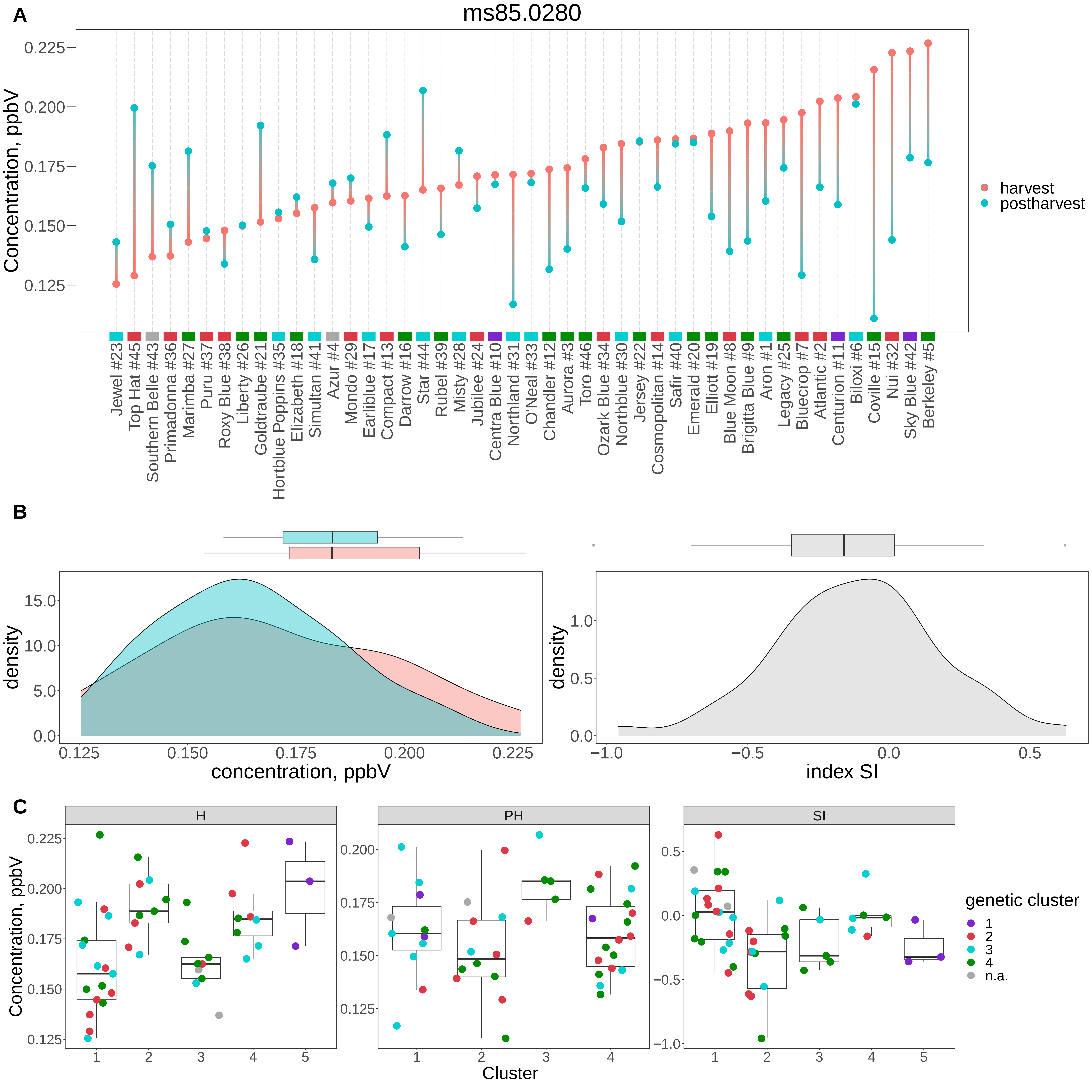
A
ms85.0280
Concentration, ppbV
harvest
postharvest
Jewel #23
Top Hat #45
Southern Belle #43
Primadonna #36
Marimba #27
Puru #37
Roxy Blue #38
Liberty #26
Goldtraube #21
Hortblue Poppins #35
Elizabeth #18
Simultan #41
Azur #4
Mondo #29
Earliblue #17
Compact #13
Darrow #16
Star #44
Rubel #39
Misty #28
Jubilee #24
Centra Blue #10
Northland #31
O'Neal #33
Chandler #12
Aurora #3
Toro #46
Ozark Blue #34
Northblue #30
Jersey #22
Cosmopolitan #14
Safir #40
Emerald #20
Elliott #19
Blue Moon #8
Brigitta Blue #9
Aron #1
Legacy #25
Bluecrop #7
Atlantic #2
Centurion #11
Biloxi #6
Coville #15
Nui #32
Sky Blue #42
Berkeley #5
B
density
concentration, ppbV
index SI
C
H
PH
SI
Concentration, ppbV
Cluster
genetic cluster
1
2
3
4
n.a.

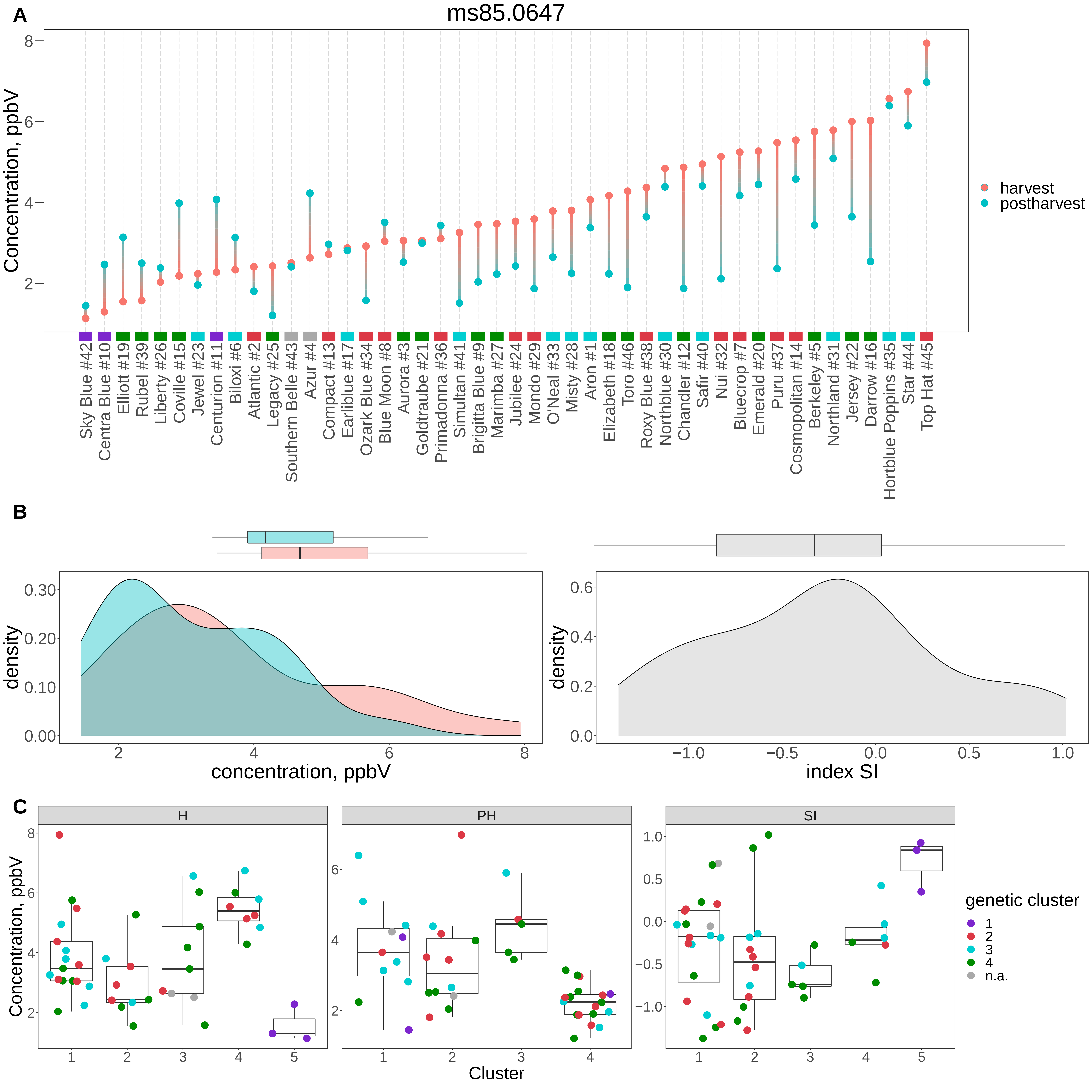
A
ms85.0647
Concentration, ppbV
harvest
postharvest
Sky Blue #42
Centra Blue #10
Elliott #19
Rubel #39
Liberty #26
Coville #15
Jewel #23
Centurion #11
Biloxi #6
Atlantic #2
Legacy #25
Southern Belle #43
Azur #4
Compact #13
Earliblue #17
Ozark Blue #34
Blue Moon #8
Aurora #3
Goldtraube #21
Primadonna #36
Simultan #41
Brigitta Blue #9
Marimba #27
Jubilee #24
Mondo #29
O'Neal #33
Misty #28
Aron #1
Elizabeth #18
Toro #46
Roxy Blue #38
Northblue #30
Chandler #12
Safir #40
Nui #32
Bluecrop #7
Emerald #20
Puru #37
Cosmopolitan #14
Berkeley #5
Northland #31
Jersey #22
Darrow #16
Hortblue Poppins #35
Star #44
Top Hat #45
B
density
concentration, ppbV
index SI
C
H
PH
SI
Concentration, ppbV
Cluster
genetic cluster
1
2
3
4
n.a.

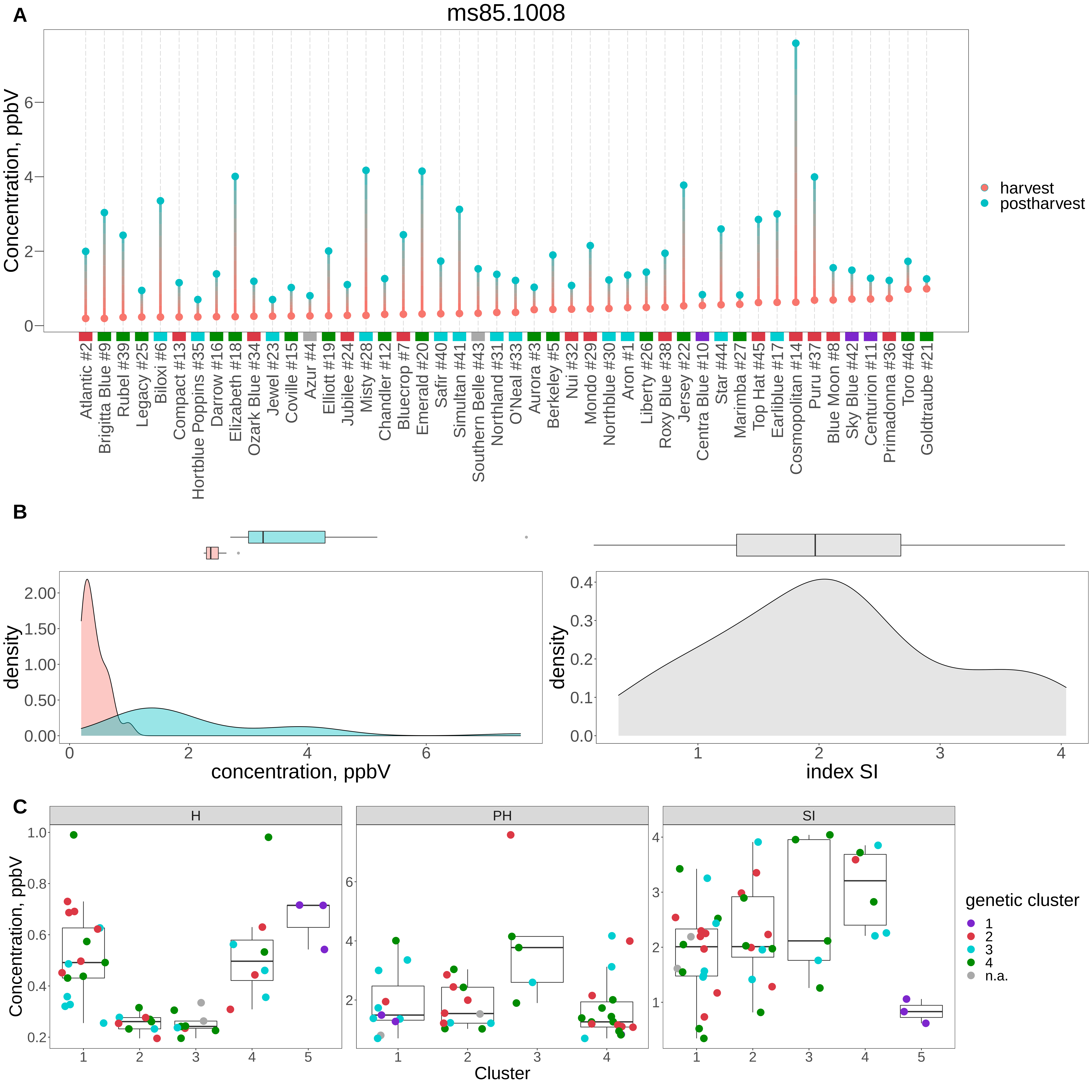

A
ms85.1008
Concentration, ppbV
harvest
postharvest
Atlantic #2
Brigitta Blue #9
Rubel #39
Legacy #25
Biloxi #6
Compact #13
Hortblue Poppins #35
Darrow #16
Elizabeth #18
Ozark Blue #34
Jewel #23
Coville #15
Azur #4
Elliott #19
Jubilee #24
Misty #28
Chandler #12
Bluecrop #7
Emerald #20
Safir #40
Simultan #41
Southern Belle #43
Northland #31
O'Neal #33
Aurora #3
Berkeley #5
Nui #32
Mondo #29
Northblue #30
Aron #1
Liberty #26
Roxy Blue #38
Jersey #22
Centra Blue #10
Star #44
Marimba #27
Top Hat #45
Earliblue #17
Cosmopolitan #14
Puru #37
Blue Moon #8
Sky Blue #42
Centurion #11
Primadonna #36
Toro #46
Goldtraube #21
B
density
concentration, ppbV
index SI
C
H
PH
SI
Concentration, ppbV
Cluster
genetic cluster
1
2
3
4
n.a.

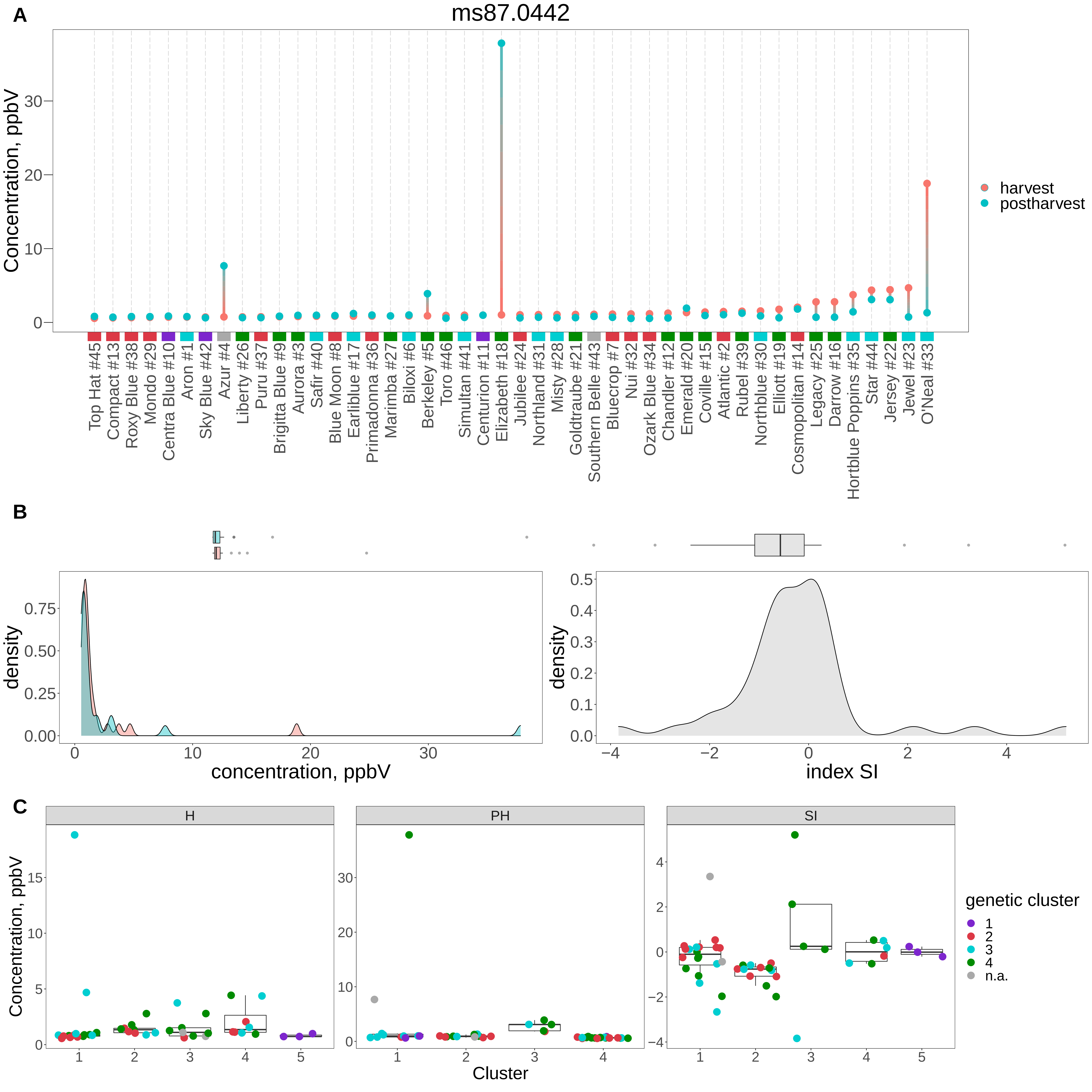

A
ms87.0442
Concentration, ppbV
harvest
postharvest
Top Hat #45
Compact #13
Roxy Blue #38
Mondo #29
Centra Blue #10
Aron #1
Sky Blue #42
Azur #4
Liberty #26
Puru #37
Brigitta Blue #9
Aurora #3
Safir #40
Blue Moon #8
Earliblue #17
Primadonna #36
Marimba #27
Biloxi #6
Berkeley #5
Toro #46
Simultan #41
Centurion #11
Elizabeth #18
Jubilee #24
Northland #31
Misty #28
Goldtraube #21
Southern Belle #43
Bluecrop #7
Nui #32
Ozark Blue #34
Chandler #12
Emerald #20
Coville #15
Atlantic #2
Rubel #39
Northblue #30
Elliott #19
Cosmopolitan #14
Legacy #25
Darrow #16
Hortblue Poppins #35
Star #44
Jersey #22
Jewel #23
O'Neal #33
B
density
concentration, ppbV
index SI
C
H
PH
SI
Cluster
genetic cluster
1
2
3
4
n.a.

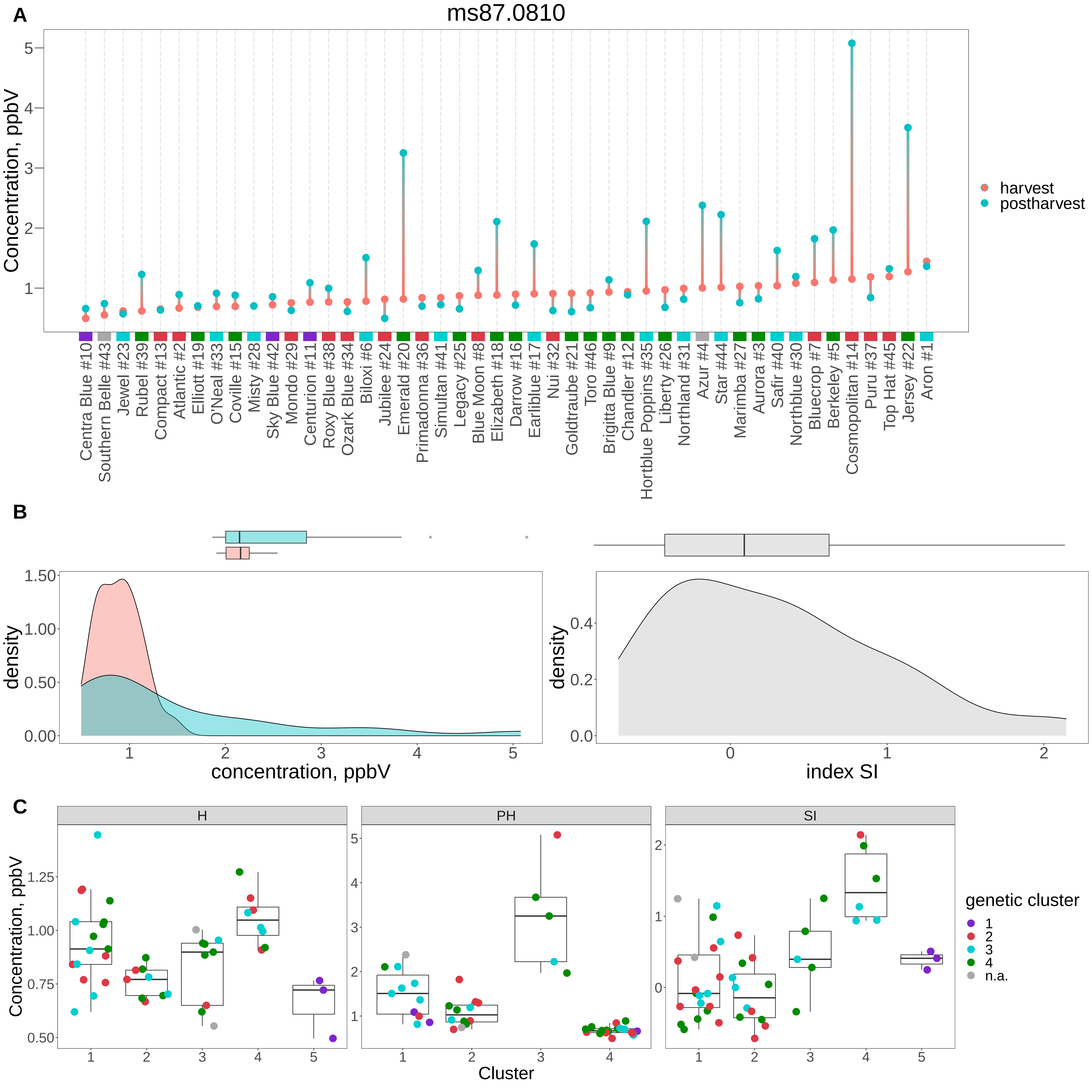
A
ms87.0810
Concentration, ppbV
harvest
postharvest
Centra Blue #10
Southern Belle #43
Jewel #23
Rubel #39
Compact #13
Atlantic #2
Elliott #19
O'Neal #33
Coville #15
Misty #28
Sky Blue #42
Mondo #29
Centurion #11
Roxy Blue #38
Ozark Blue #34
Biloxi #6
Jubilee #24
Emerald #20
Primadonna #36
Simultan #41
Legacy #25
Blue Moon #8
Elizabeth #18
Darrow #16
Earliblue #17
Nui #32
Goldtraube #21
Toro #46
Brigitta Blue #9
Chandler #12
Hortblue Poppins #35
Liberty #26
Northland #31
Azur #4
Star #44
Marimba #27
Aurora #3
Safir #40
Northblue #30
Bluecrop #7
Berkeley #5
Cosmopolitan #14
Puru #37
Top Hat #45
Jersey #22
Aron #1
B
density
concentration, ppbV
index SI
C
H
PH
SI
Concentration, ppbV
Cluster
genetic cluster
1
2
3
4
n.a.

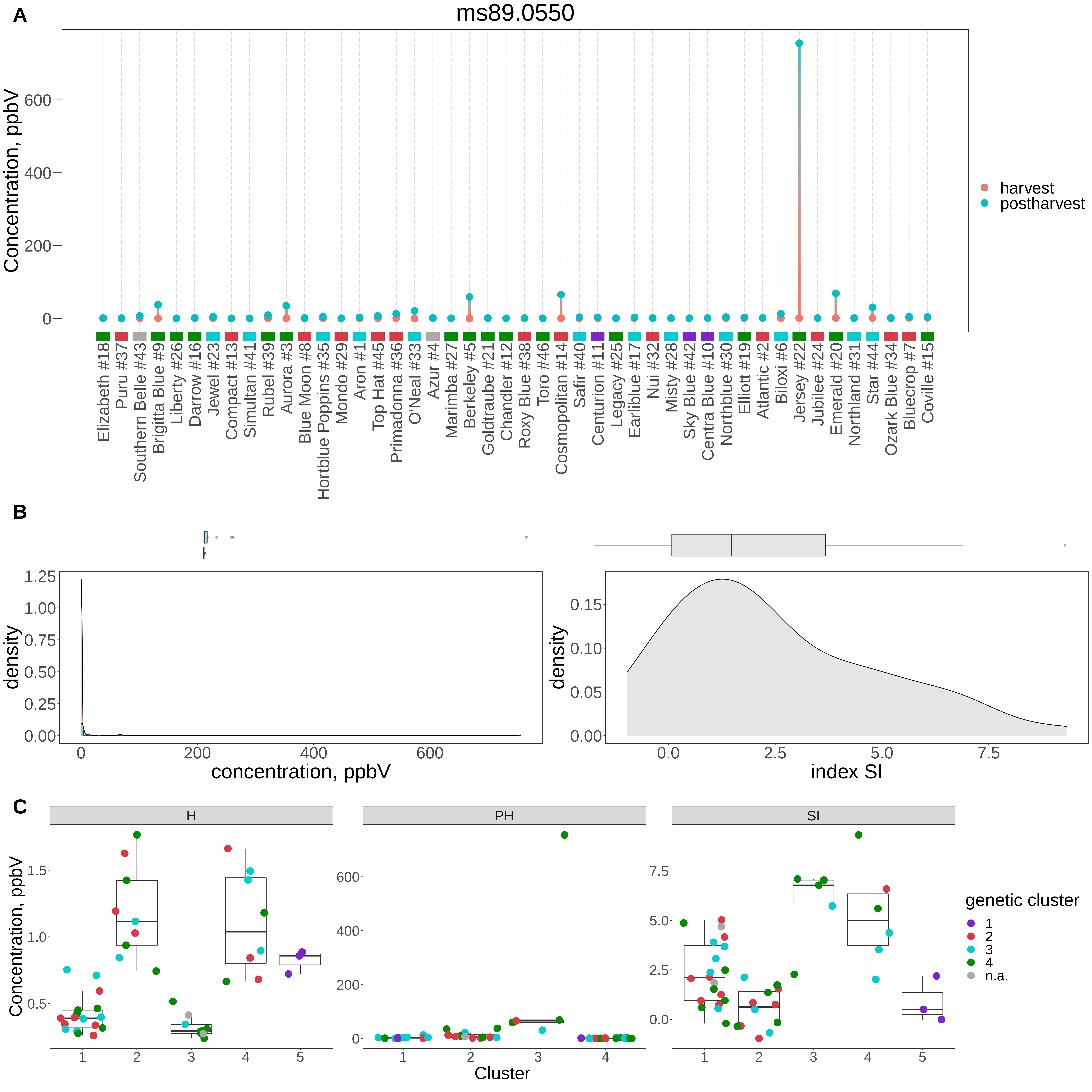
A
ms89.0550
Concentration, ppbV
harvest
postharvest
Elizabeth #18
Puru #37
Southern Belle #43
Brigitta Blue #9
Liberty #26
Darrow #16
Jewel #23
Compact #13
Simultan #41
Rubel #39
Aurora #3
Blue Moon #8
Hortblue Poppins #35
Mondo #29
Aron #1
Top Hat #45
Primadonna #36
O'Neal #33
Azur #4
Marimba #27
Berkeley #5
Goldtraube #21
Chandler #12
Roxy Blue #38
Toro #46
Cosmopolitan #14
Safir #40
Centurion #11
Legacy #25
Earliblue #17
Nui #32
Misty #28
Sky Blue #42
Centra Blue #10
Northblue #30
Elliott #19
Atlantic #2
Biloxi #6
Jersey #22
Jubilee #24
Emerald #20
Northland #31
Star #44
Ozark Blue #34
Bluecrop #7
Coville #15
B
density
concentration, ppbV
density
index SI
C
H
PH
SI
Concentration, ppbV
Cluster
genetic cluster
1
2
3
4
n.a.

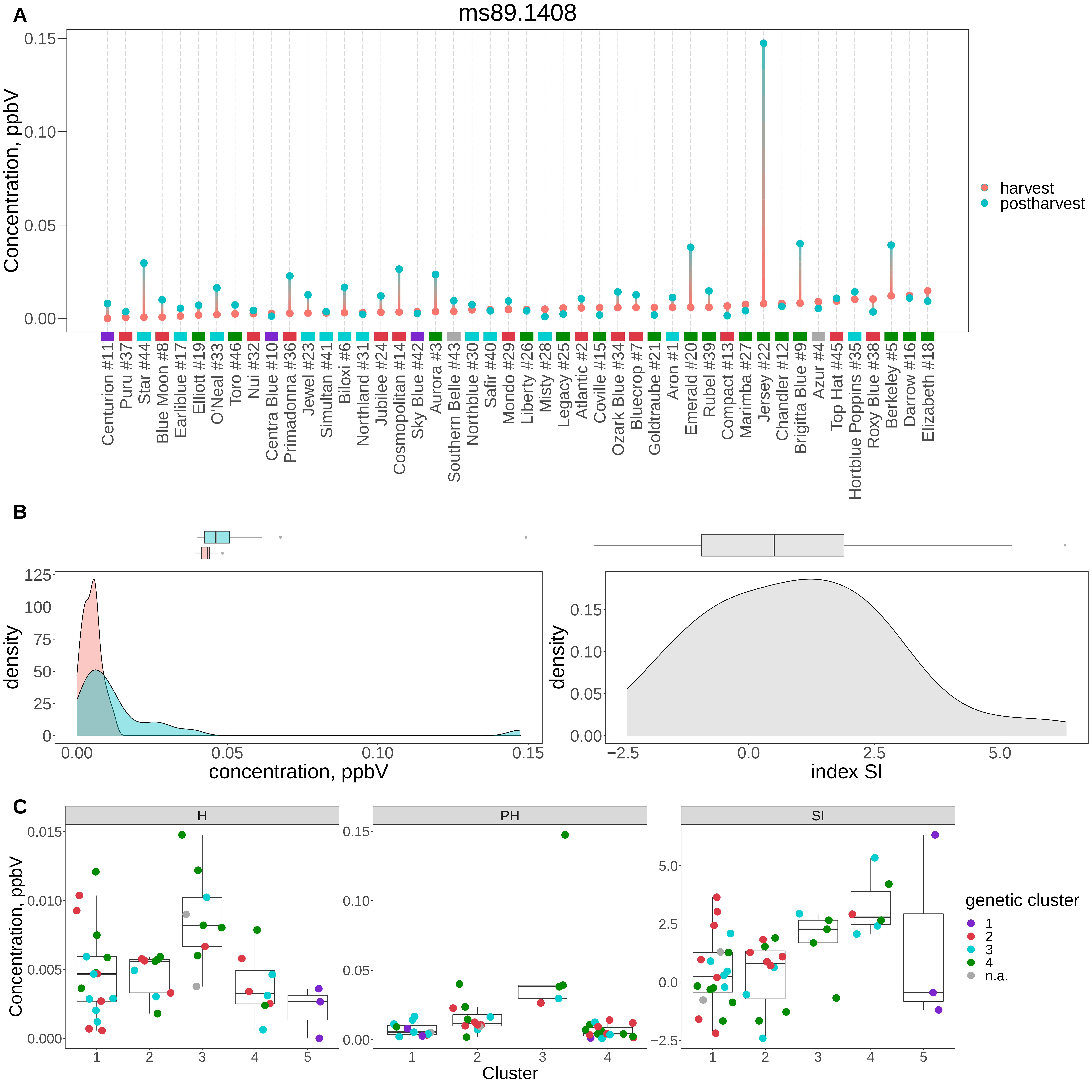

A
ms89.1408
Concentration, ppbV
harvest
postharvest
Centurion #11
Puru #37
Star #44
Blue Moon #8
Earliblue #17
Elliott #19
O'Neal #33
Toro #46
Nui #32
Centra Blue #10
Primadonna #36
Jewel #23
Simultan #41
Biloxi #6
Northland #31
Jubilee #24
Cosmopolitan #14
Sky Blue #42
Aurora #3
Southern Belle #43
Northblue #30
Safir #40
Mondo #29
Liberty #26
Misty #28
Legacy #25
Atlantic #2
Coville #15
Ozark Blue #34
Bluecrop #7
Goldtraube #21
Aron #1
Emerald #20
Rubel #39
Compact #13
Marimba #27
Jersey #22
Chandler #12
Brigitta Blue #9
Azur #4
Top Hat #45
Hortblue Poppins #35
Roxy Blue #38
Berkeley #5
Darrow #16
Elizabeth #18
B
density
concentration, ppbV
index SI
C
H
PH
SI
Cluster
genetic cluster
1
2
3
4
n.a.

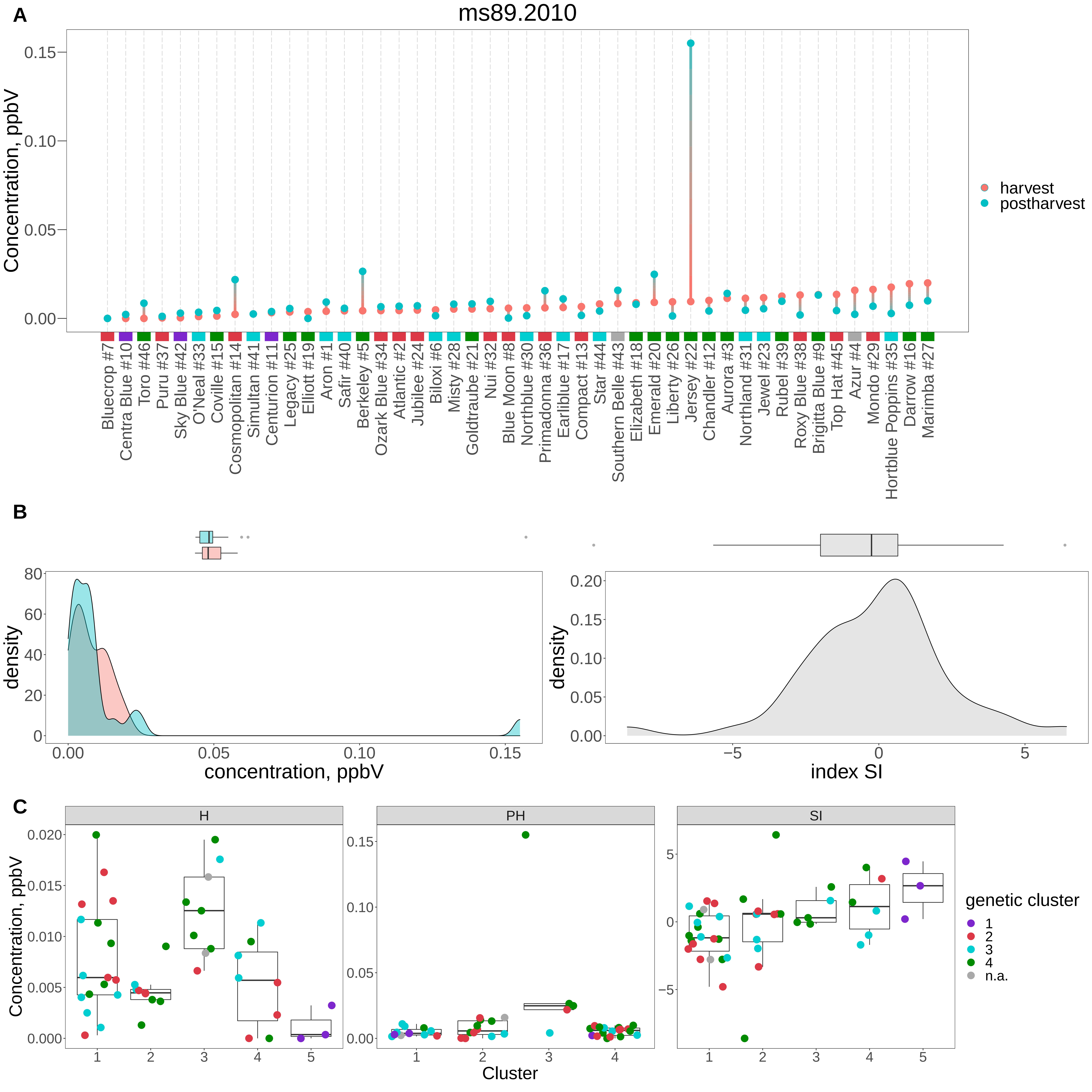
A
ms89.2010
Concentration, ppbV
0.00
0.05
0.10
0.15
harvest
postharvest
Bluecrop #7
Centra Blue #10
Toro #46
Puru #37
Sky Blue #42
O'Neal #33
Coville #15
Cosmopolitan #14
Simultan #41
Centurion #11
Legacy #25
Elliott #19
Aron #1
Safir #40
Berkeley #5
Ozark Blue #34
Atlantic #2
Jubilee #24
Biloxi #6
Misty #28
Goldtraube #21
Nui #32
Blue Moon #8
Northblue #30
Primadonna #36
Earliblue #17
Compact #13
Star #44
Southern Belle #43
Elizabeth #18
Emerald #20
Liberty #26
Jersey #22
Chandler #12
Aurora #3
Northland #31
Jewel #23
Rubel #39
Roxy Blue #38
Brigitta Blue #9
Top Hat #45
Azur #4
Mondo #29
Hortblue Poppins #35
Darrow #16
Marimba #27
B
density
0
20
40
60
80
0.00
0.05
0.10
0.15
concentration, ppbV
density
0.00
0.05
0.10
0.15
0.20
−5
0
5
index SI
C
H
PH
SI
Concentration, ppbV
0.000
0.005
0.010
0.015
0.020
1
2
3
4
5
0.00
0.05
0.10
0.15
1
2
3
4
Cluster
−5
0
5
1
2
3
4
5
genetic cluster
1
2
3
4
n.a.

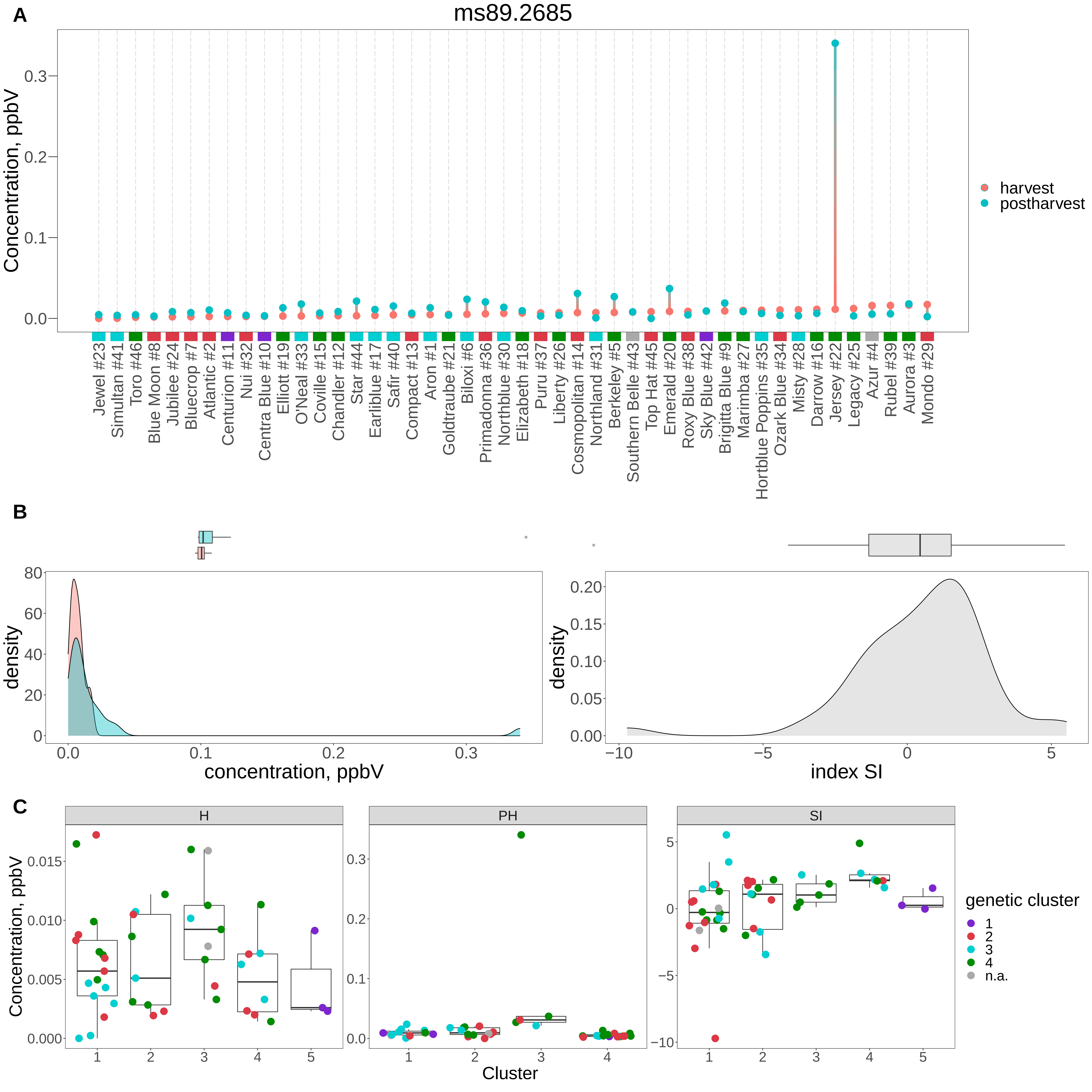
A
ms89.2685
Concentration, ppbV
harvest
postharvest
Jewel #23
Simultan #41
Toro #46
Blue Moon #8
Jubilee #24
Bluecrop #7
Atlantic #2
Centurion #11
Nui #32
Centra Blue #10
Elliott #19
O'Neal #33
Coville #15
Chandler #12
Star #44
Earliblue #17
Safir #40
Compact #13
Aron #1
Goldtraube #21
Biloxi #6
Primadonna #36
Northblue #30
Elizabeth #18
Puru #37
Liberty #26
Cosmopolitan #14
Northland #31
Berkeley #5
Southern Belle #43
Top Hat #45
Emerald #20
Roxy Blue #38
Sky Blue #42
Brigitta Blue #9
Marimba #27
Hortblue Poppins #35
Ozark Blue #34
Misty #28
Darrow #16
Jersey #22
Legacy #25
Azur #4
Rubel #39
Aurora #3
Mondo #29
B
density
concentration, ppbV
index SI
C
H
PH
SI
Cluster
genetic cluster
1
2
3
4
n.a.

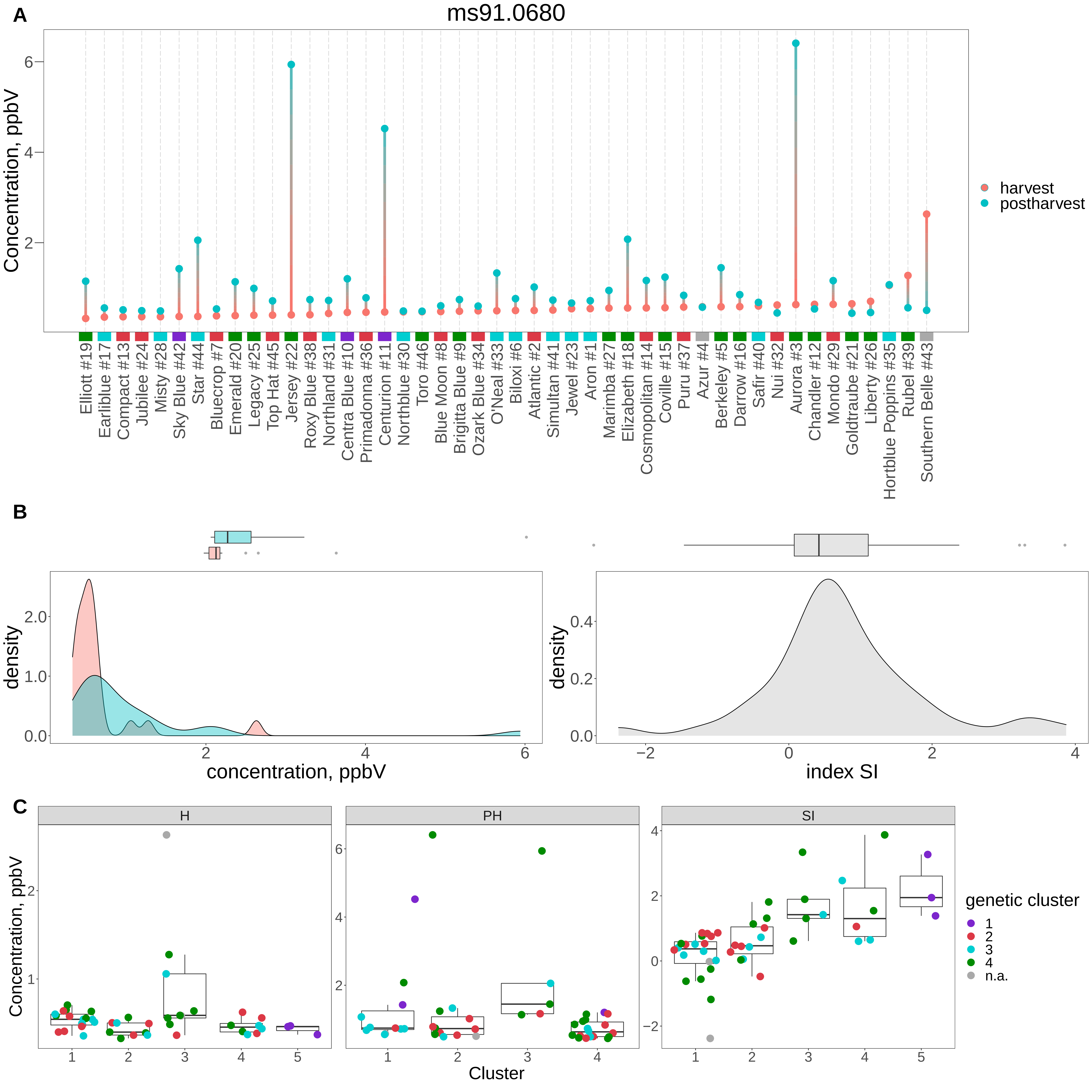

A
ms91.0680
Concentration, ppbV
harvest
postharvest
Elliott #19
Earliblue #17
Compact #13
Jubilee #24
Misty #28
Sky Blue #42
Star #44
Bluecrop #7
Emerald #20
Legacy #25
Top Hat #45
Jersey #22
Roxy Blue #38
Northland #31
Centra Blue #10
Primadonna #36
Centurion #11
Northblue #30
Toro #46
Blue Moon #8
Brigitta Blue #9
Ozark Blue #34
O'Neal #33
Biloxi #6
Atlantic #2
Simultan #41
Jewel #23
Aron #1
Marimba #27
Elizabeth #18
Cosmopolitan #14
Coville #15
Puru #37
Azur #4
Berkeley #5
Darrow #16
Safir #40
Nui #32
Aurora #3
Chandler #12
Mondo #29
Goldtraube #21
Liberty #26
Hortblue Poppins #35
Rubel #39
Southern Belle #43
B
density
concentration, ppbV
index SI
C
H
PH
SI
Concentration, ppbV
Cluster
genetic cluster
1
2
3
4
n.a.

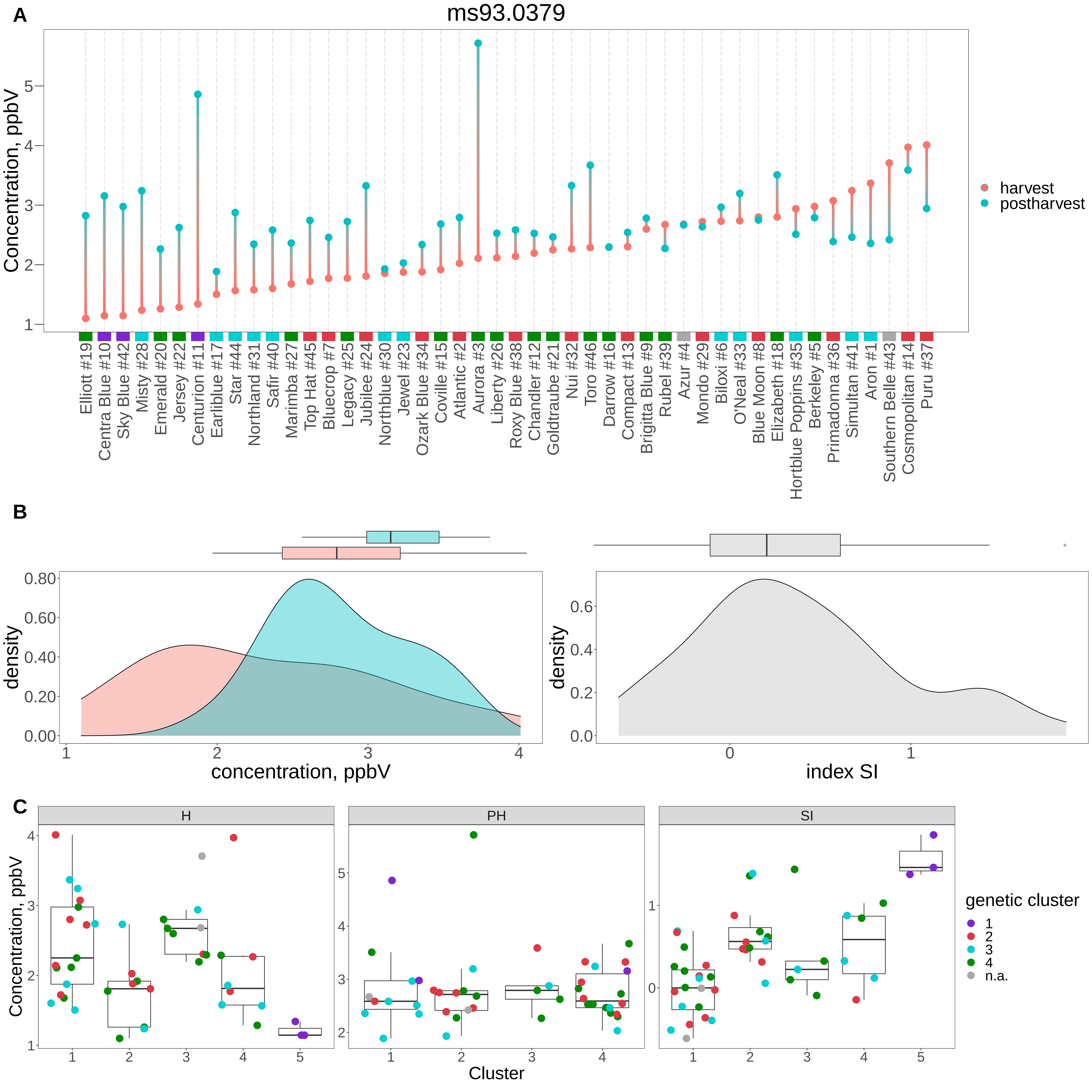

A
ms93.0379
Concentration, ppbV
harvest
postharvest
Elliott #19
Centra Blue #10
Sky Blue #42
Misty #28
Emerald #20
Jersey #22
Centurion #11
Earliblue #17
Star #44
Northland #31
Safir #40
Marimba #27
Top Hat #45
Bluecrop #7
Legacy #25
Jubilee #24
Northblue #30
Jewel #23
Ozark Blue #34
Coville #15
Atlantic #2
Aurora #3
Liberty #26
Roxy Blue #38
Chandler #12
Goldtraube #21
Nui #32
Toro #46
Darrow #16
Compact #13
Brigitta Blue #9
Rubel #39
Azur #4
Mondo #29
Biloxi #6
O'Neal #33
Blue Moon #8
Elizabeth #18
Hortblue Poppins #35
Berkeley #5
Primadonna #36
Simultan #41
Aron #1
Southern Belle #43
Cosmopolitan #14
Puru #37
B
density
concentration, ppbV
density
index SI
C
H
PH
SI
Concentration, ppbV
Cluster
genetic cluster
1
2
3
4
n.a.

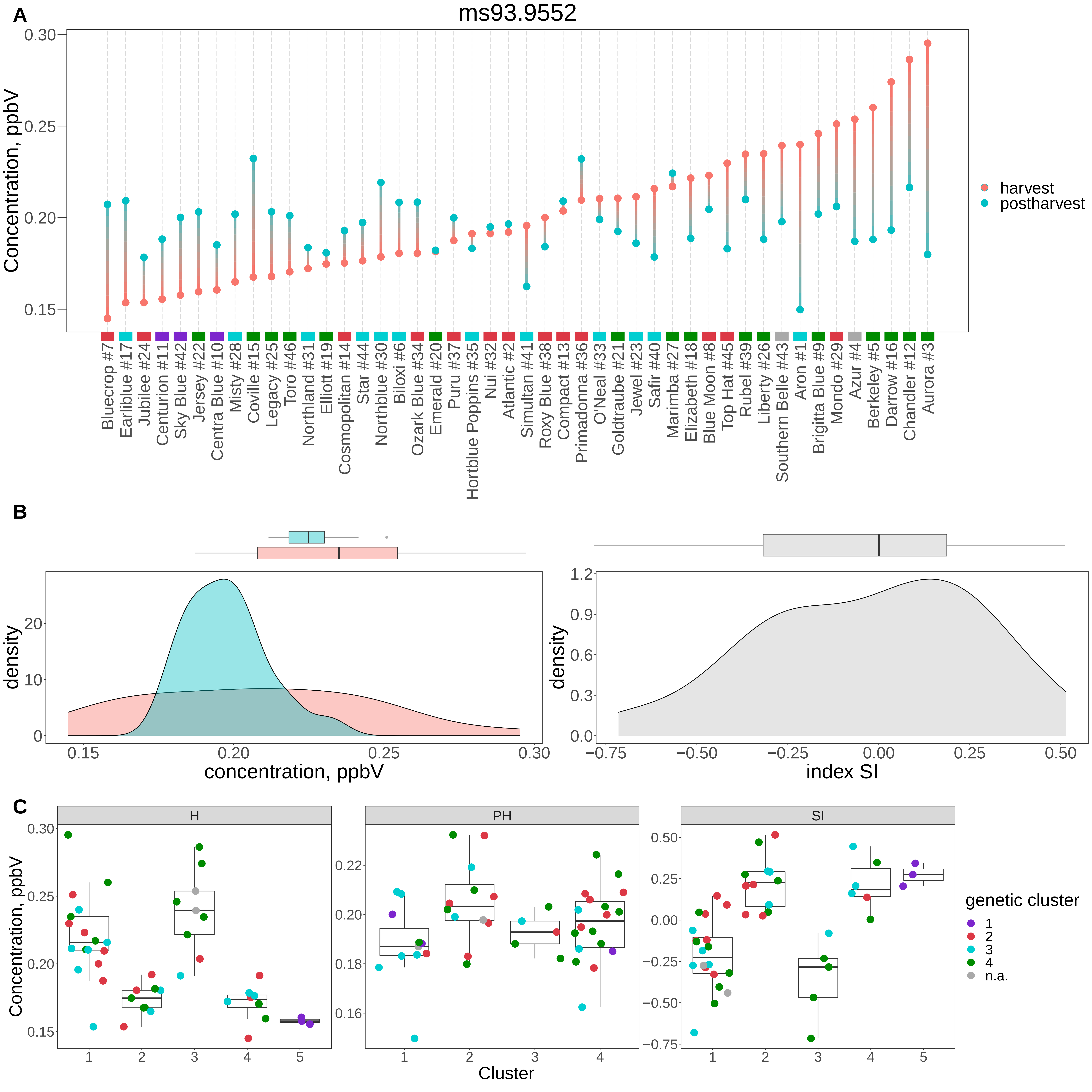

A
ms93.9552
Concentration, ppbV
0.30
0.25
0.20
0.15
harvest
postharvest
Bluecrop #7
Earliblue #17
Jubilee #24
Centurion #11
Sky Blue #42
Jersey #22
Centra Blue #10
Misty #28
Coville #15
Legacy #25
Toro #46
Northland #31
Elliott #19
Cosmopolitan #14
Star #44
Northblue #30
Biloxi #6
Ozark Blue #34
Emerald #20
Puru #37
Hortblue Poppins #35
Nui #32
Atlantic #2
Simultan #41
Roxy Blue #38
Compact #13
Primadonna #36
O'Neal #33
Goldtraube #21
Jewel #23
Safir #40
Marimba #27
Elizabeth #18
Blue Moon #8
Top Hat #45
Rubel #39
Liberty #26
Southern Belle #43
Aron #1
Brigitta Blue #9
Mondo #29
Azur #4
Berkeley #5
Darrow #16
Chandler #12
Aurora #3
B
density
20
10
0
0.15
0.20
0.25
0.30
concentration, ppbV
density
1.2
0.9
0.6
0.3
0.0
−0.75
−0.50
−0.25
0.00
0.25
0.50
index SI
C
H
PH
SI
Concentration, ppbV
0.30
0.25
0.20
0.15
1
2
3
4
5
0.22
0.20
0.18
0.16
1
2
3
4
0.50
0.25
0.00
−0.25
−0.50
−0.75
1
2
3
4
5
Cluster
genetic cluster
1
2
3
4
n.a.

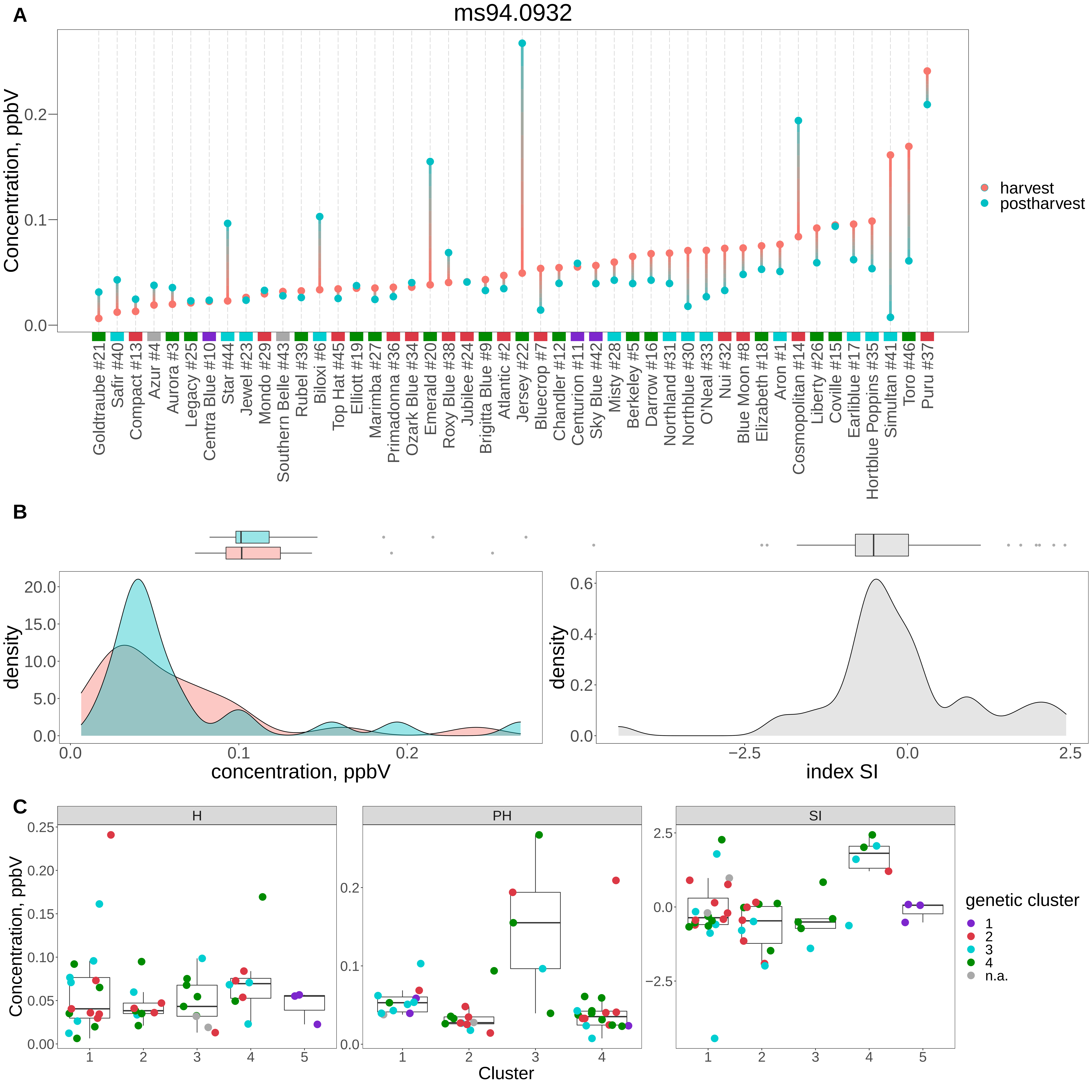

A
ms94.0932
Concentration, ppbV
harvest
postharvest
Goldtraube #21
Safir #40
Compact #13
Azur #4
Aurora #3
Legacy #25
Centra Blue #10
Star #44
Jewel #23
Mondo #29
Southern Belle #43
Rubel #39
Biloxi #6
Top Hat #45
Elliott #19
Marimba #27
Primadonna #36
Ozark Blue #34
Emerald #20
Roxy Blue #38
Jubilee #24
Brigitta Blue #9
Atlantic #2
Jersey #22
Bluecrop #7
Chandler #12
Centurion #11
Sky Blue #42
Misty #28
Berkeley #5
Darrow #16
Northland #31
Northblue #30
O'Neal #33
Nui #32
Blue Moon #8
Elizabeth #18
Aron #1
Cosmopolitan #14
Liberty #26
Coville #15
Earliblue #17
Hortblue Poppins #35
Simultan #41
Toro #46
Puru #37
B
density
concentration, ppbV
index SI
C
H
PH
SI
Concentration, ppbV
Cluster
genetic cluster
1
2
3
4
n.a.

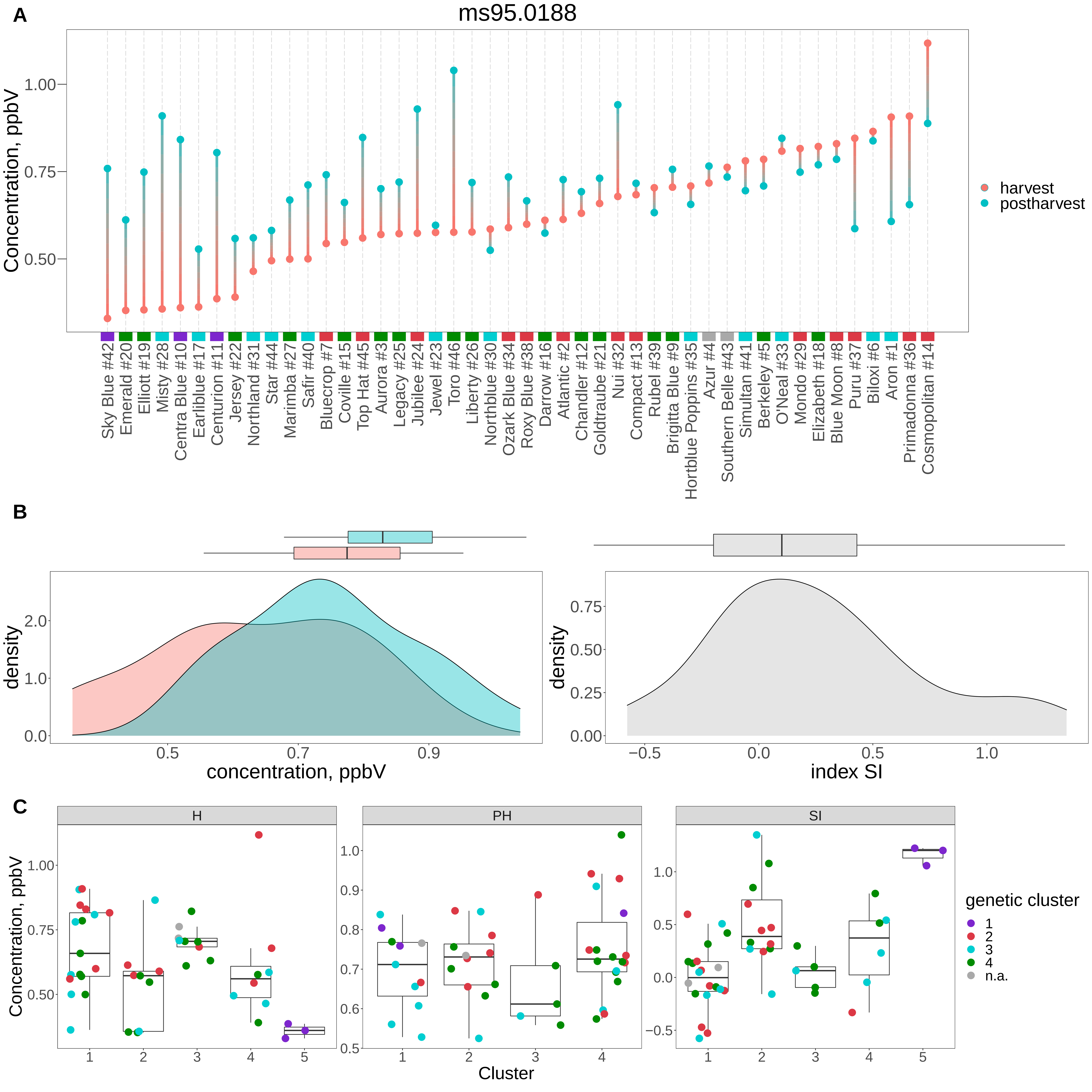
A
ms95.0188
Concentration, ppbV
harvest
postharvest
Sky Blue #42
Emerald #20
Elliott #19
Misty #28
Centra Blue #10
Earliblue #17
Centurion #11
Jersey #22
Northland #31
Star #44
Marimba #27
Safir #40
Bluecrop #7
Coville #15
Top Hat #45
Aurora #3
Legacy #25
Jubilee #24
Jewel #23
Toro #46
Liberty #26
Northblue #30
Ozark Blue #34
Roxy Blue #38
Darrow #16
Atlantic #2
Chandler #12
Goldtraube #21
Nui #32
Compact #13
Rubel #39
Brigitta Blue #9
Hortblue Poppins #35
Azur #4
Southern Belle #43
Simultan #41
Berkeley #5
O'Neal #33
Mondo #29
Elizabeth #18
Blue Moon #8
Puru #37
Biloxi #6
Aron #1
Primadonna #36
Cosmopolitan #14
B
density
concentration, ppbV
index SI
C
H
PH
SI
Concentration, ppbV
Cluster
genetic cluster
1
2
3
4
n.a.

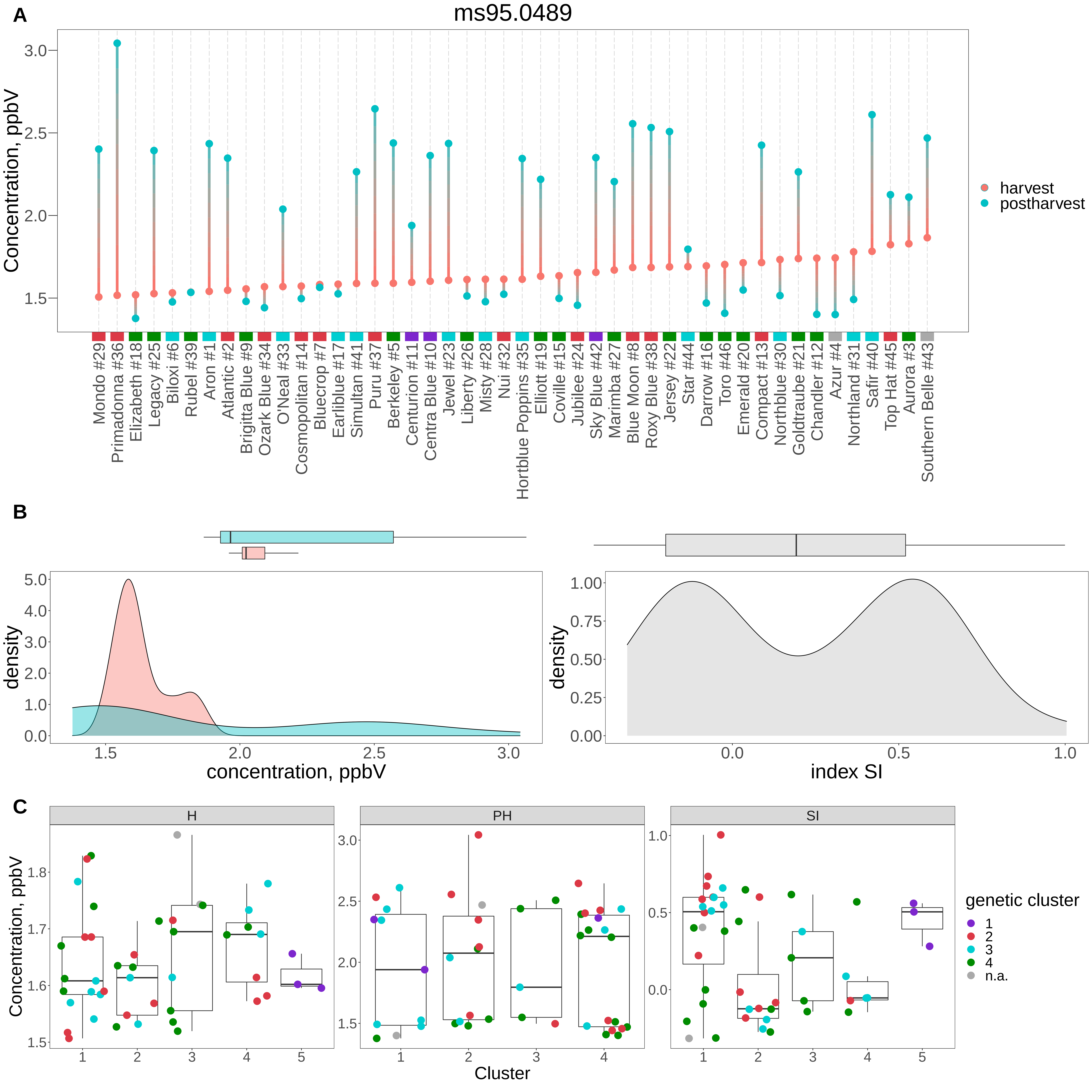

A
ms95.0489
Concentration, ppbV
harvest
postharvest
Mondo #29
Primadonna #36
Elizabeth #18
Legacy #25
Biloxi #6
Rubel #39
Aron #1
Atlantic #2
Brigitta Blue #9
Ozark Blue #34
O'Neal #33
Cosmopolitan #14
Bluecrop #7
Earliblue #17
Simultan #41
Puru #37
Berkeley #5
Centurion #11
Centra Blue #10
Jewel #23
Liberty #26
Misty #28
Nui #32
Hortblue Poppins #35
Elliott #19
Coville #15
Jubilee #24
Sky Blue #42
Marimba #27
Blue Moon #8
Roxy Blue #38
Jersey #22
Star #44
Darrow #16
Toro #46
Emerald #20
Compact #13
Northblue #30
Goldtraube #21
Chandler #12
Azur #4
Northland #31
Safir #40
Top Hat #45
Aurora #3
Southern Belle #43
B
density
concentration, ppbV
index SI
C
H
PH
SI
Cluster
genetic cluster
1
2
3
4
n.a.

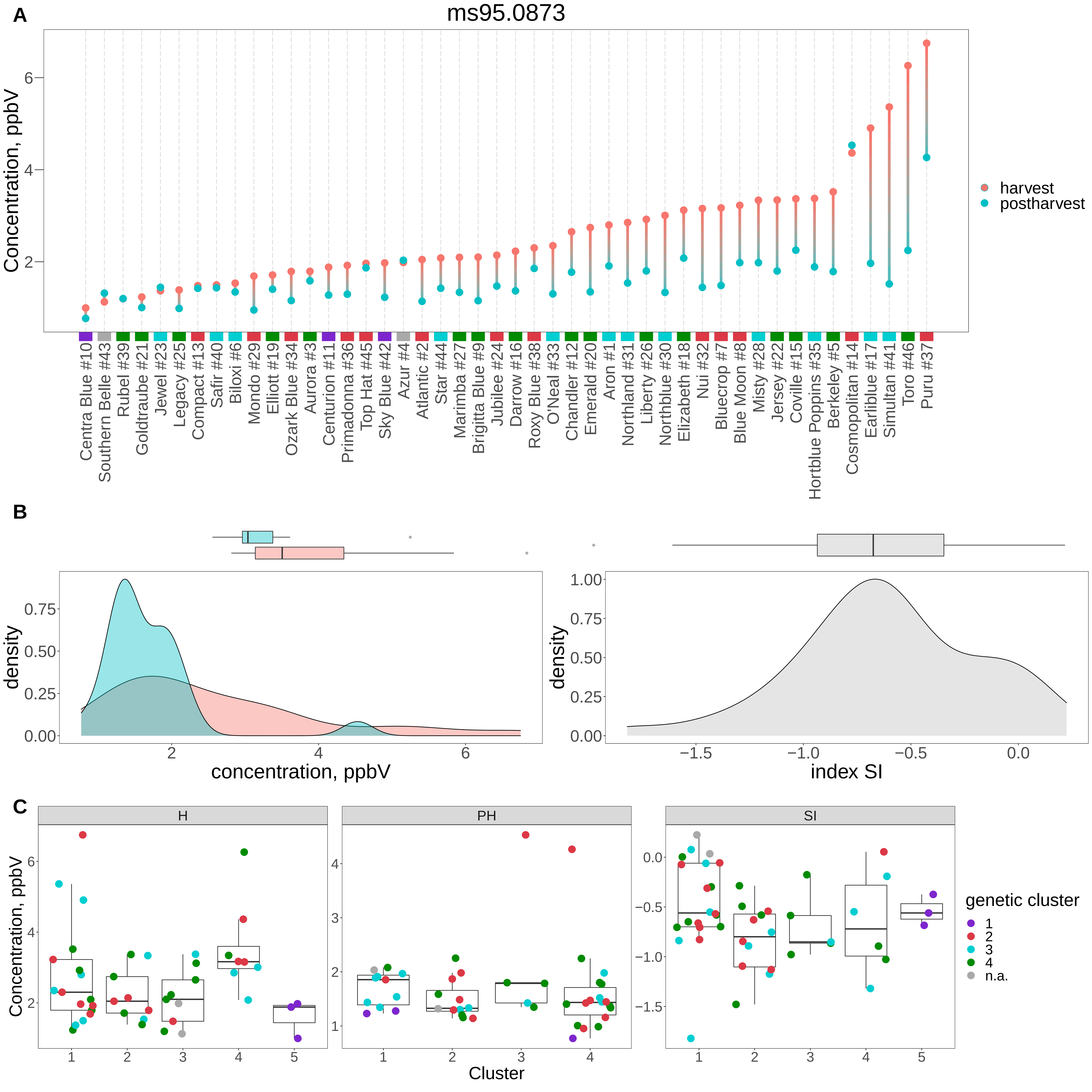

A
ms95.0873
Concentration, ppbV
harvest
postharvest
Centra Blue #10
Southern Belle #43
Rubel #39
Goldtraube #21
Jewel #23
Legacy #25
Compact #13
Safir #40
Biloxi #6
Mondo #29
Elliott #19
Ozark Blue #34
Aurora #3
Centurion #11
Primadonna #36
Top Hat #45
Sky Blue #42
Azur #4
Atlantic #2
Star #44
Marimba #27
Brigitta Blue #9
Jubilee #24
Darrow #16
Roxy Blue #38
O'Neal #33
Chandler #12
Emerald #20
Aron #1
Northland #31
Liberty #26
Northblue #30
Elizabeth #18
Nui #32
Bluecrop #7
Blue Moon #8
Misty #28
Jersey #22
Coville #15
Hortblue Poppins #35
Berkeley #5
Cosmopolitan #14
Earliblue #17
Simultan #41
Toro #46
Puru #37
B
density
concentration, ppbV
index SI
C
H
PH
SI
Concentration, ppbV
Cluster
genetic cluster
1
2
3
4
n.a.

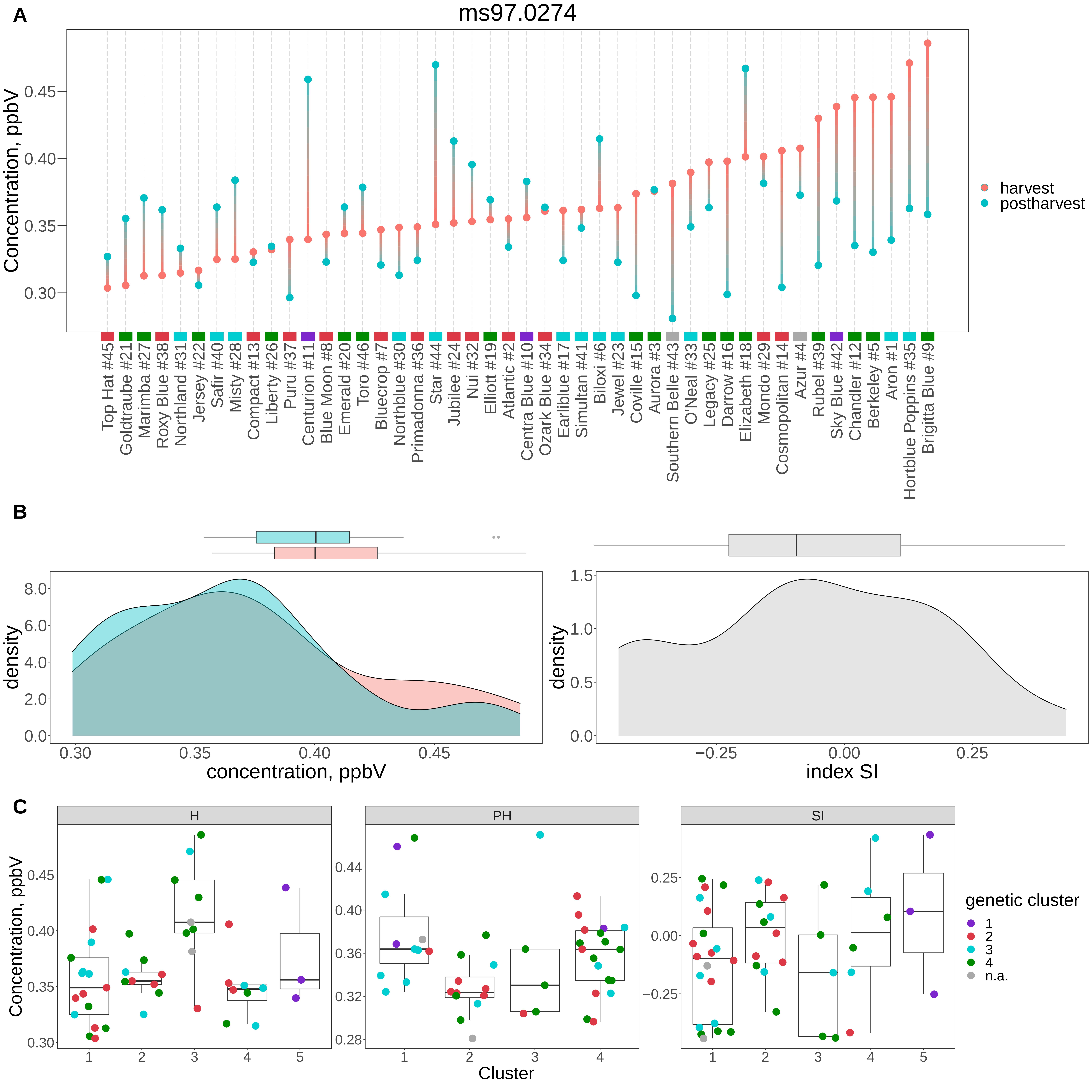
A
ms97.0274
Concentration, ppbV
harvest
postharvest
Top Hat #45
Goldtraube #21
Marimba #27
Roxy Blue #38
Northland #31
Jersey #22
Safir #40
Misty #28
Compact #13
Liberty #26
Puru #37
Centurion #11
Blue Moon #8
Emerald #20
Toro #46
Bluecrop #7
Northblue #30
Primadonna #36
Star #44
Jubilee #24
Nui #32
Elliott #19
Atlantic #2
Centra Blue #10
Ozark Blue #34
Earliblue #17
Simultan #41
Biloxi #6
Jewel #23
Coville #15
Aurora #3
Southern Belle #43
O'Neal #33
Legacy #25
Darrow #16
Elizabeth #18
Mondo #29
Cosmopolitan #14
Azur #4
Rubel #39
Sky Blue #42
Chandler #12
Berkeley #5
Aron #1
Hortblue Poppins #35
Brigitta Blue #9
B
density
concentration, ppbV
index SI
C
H
PH
SI
Cluster
genetic cluster
1
2
3
4
n.a.

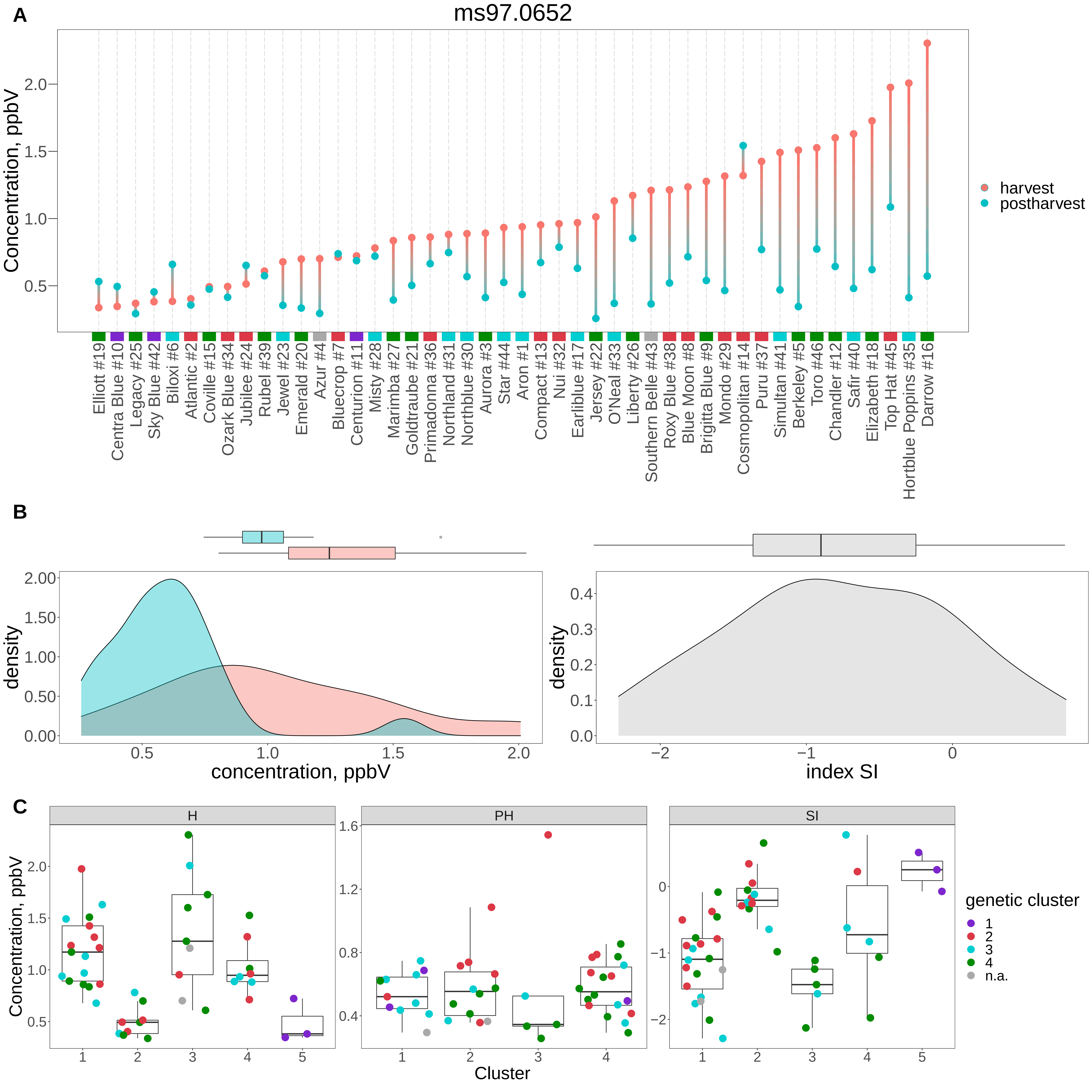

A
ms97.0652
Concentration, ppbV
harvest
postharvest
Elliott #19
Centra Blue #10
Legacy #25
Sky Blue #42
Biloxi #6
Atlantic #2
Coville #15
Ozark Blue #34
Jubilee #24
Rubel #39
Jewel #23
Emerald #20
Azur #4
Bluecrop #7
Centurion #11
Misty #28
Marimba #27
Goldtraube #21
Primadonna #36
Northland #31
Northblue #30
Aurora #3
Star #44
Aron #1
Compact #13
Nui #32
Earliblue #17
Jersey #22
O'Neal #33
Liberty #26
Southern Belle #43
Roxy Blue #38
Blue Moon #8
Brigitta Blue #9
Mondo #29
Cosmopolitan #14
Puru #37
Simultan #41
Berkeley #5
Toro #46
Chandler #12
Safir #40
Elizabeth #18
Top Hat #45
Hortblue Poppins #35
Darrow #16
B
density
concentration, ppbV
density
index SI
C
H
PH
SI
Concentration, ppbV
Cluster
genetic cluster
1
2
3
4
n.a.

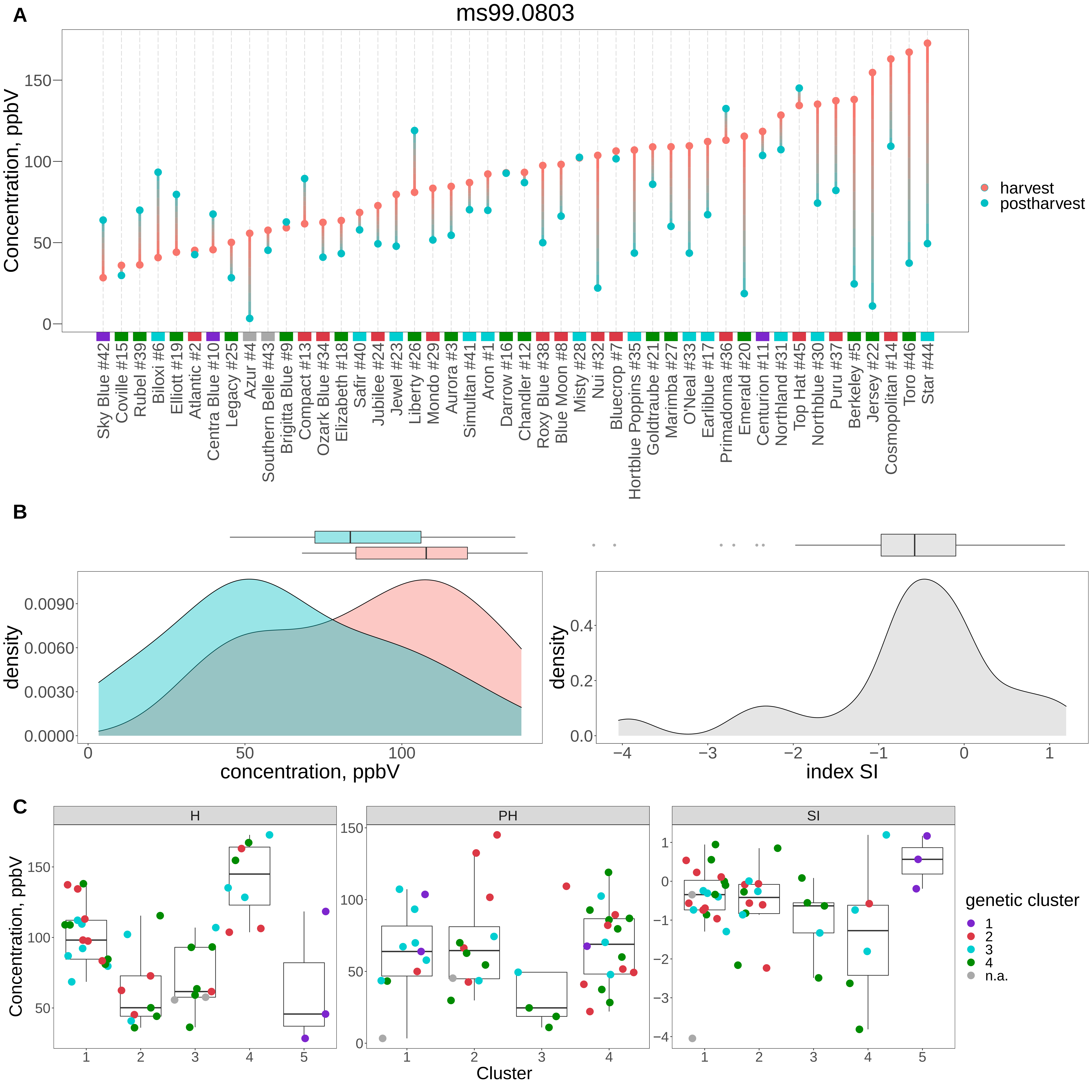
A
ms99.0803
Concentration, ppbV
harvest
postharvest
Sky Blue #42
Coville #15
Rubel #39
Biloxi #6
Elliott #19
Atlantic #2
Centra Blue #10
Legacy #25
Azur #4
Southern Belle #43
Brigitta Blue #9
Compact #13
Ozark Blue #34
Elizabeth #18
Safir #40
Jubilee #24
Jewel #23
Liberty #26
Mondo #29
Aurora #3
Simultan #41
Aron #1
Darrow #16
Chandler #12
Roxy Blue #38
Blue Moon #8
Misty #28
Nui #32
Bluecrop #7
Hortblue Poppins #35
Goldtraube #21
Marimba #27
O'Neal #33
Earliblue #17
Primadonna #36
Emerald #20
Centurion #11
Northland #31
Top Hat #45
Northblue #30
Puru #37
Berkeley #5
Jersey #22
Cosmopolitan #14
Toro #46
Star #44
B
density
concentration, ppbV
index SI
C
H
PH
SI
Concentration, ppbV
Cluster
genetic cluster
1
2
3
4
n.a.

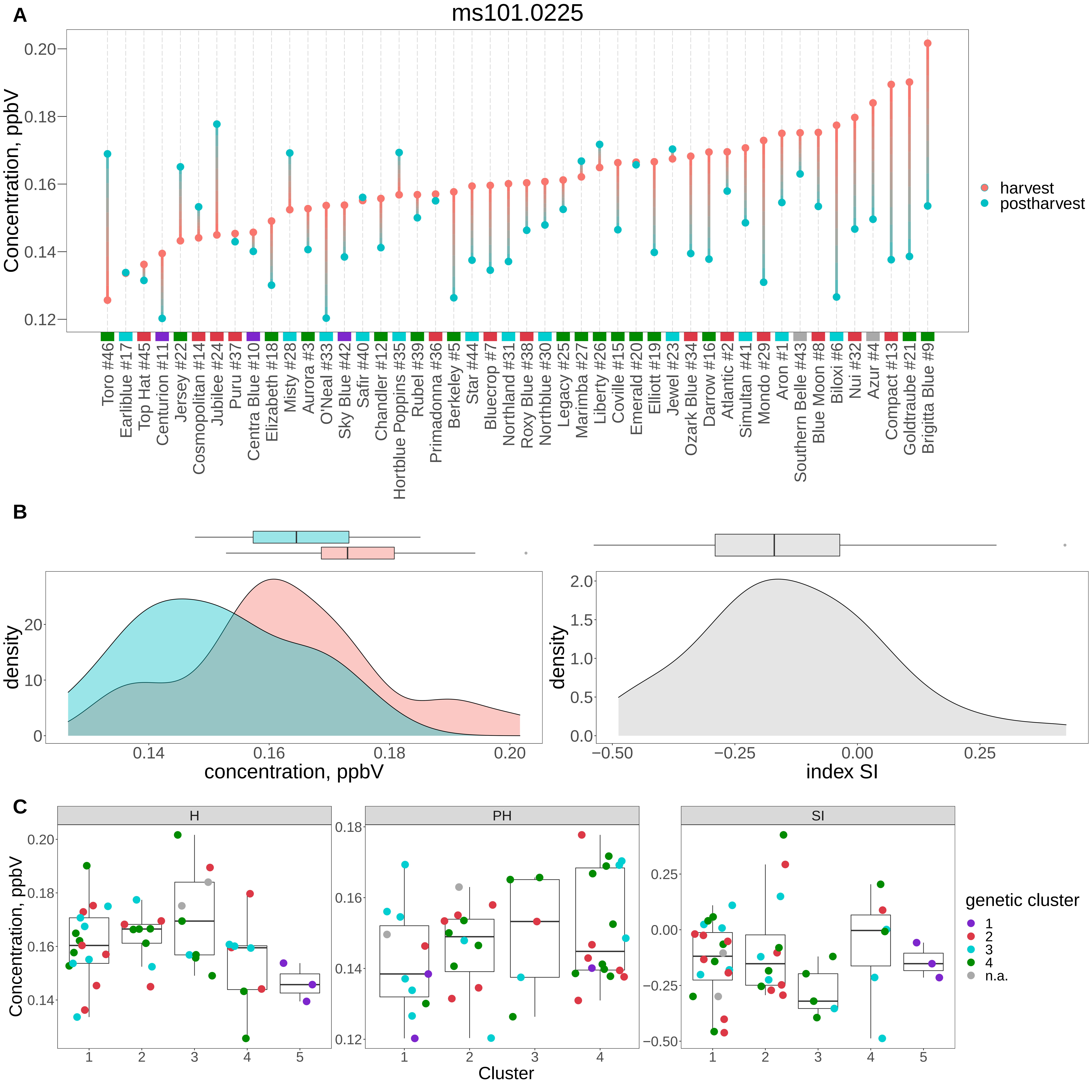
A
ms101.0225
Concentration, ppbV
harvest
postharvest
Toro #46
Earliblue #17
Top Hat #45
Centurion #11
Jersey #22
Cosmopolitan #14
Jubilee #24
Puru #37
Centra Blue #10
Elizabeth #18
Misty #28
Aurora #3
O'Neal #33
Sky Blue #42
Safir #40
Chandler #12
Hortblue Poppins #35
Rubel #39
Primadonna #36
Berkeley #5
Star #44
Bluecrop #7
Northland #31
Roxy Blue #38
Northblue #30
Legacy #25
Marimba #27
Liberty #26
Coville #15
Emerald #20
Elliott #19
Jewel #23
Ozark Blue #34
Darrow #16
Atlantic #2
Simultan #41
Mondo #29
Aron #1
Southern Belle #43
Blue Moon #8
Biloxi #6
Nui #32
Azur #4
Compact #13
Goldtraube #21
Brigitta Blue #9
B
density
concentration, ppbV
index SI
C
H
PH
SI
Concentration, ppbV
Cluster
genetic cluster
1
2
3
4
n.a.

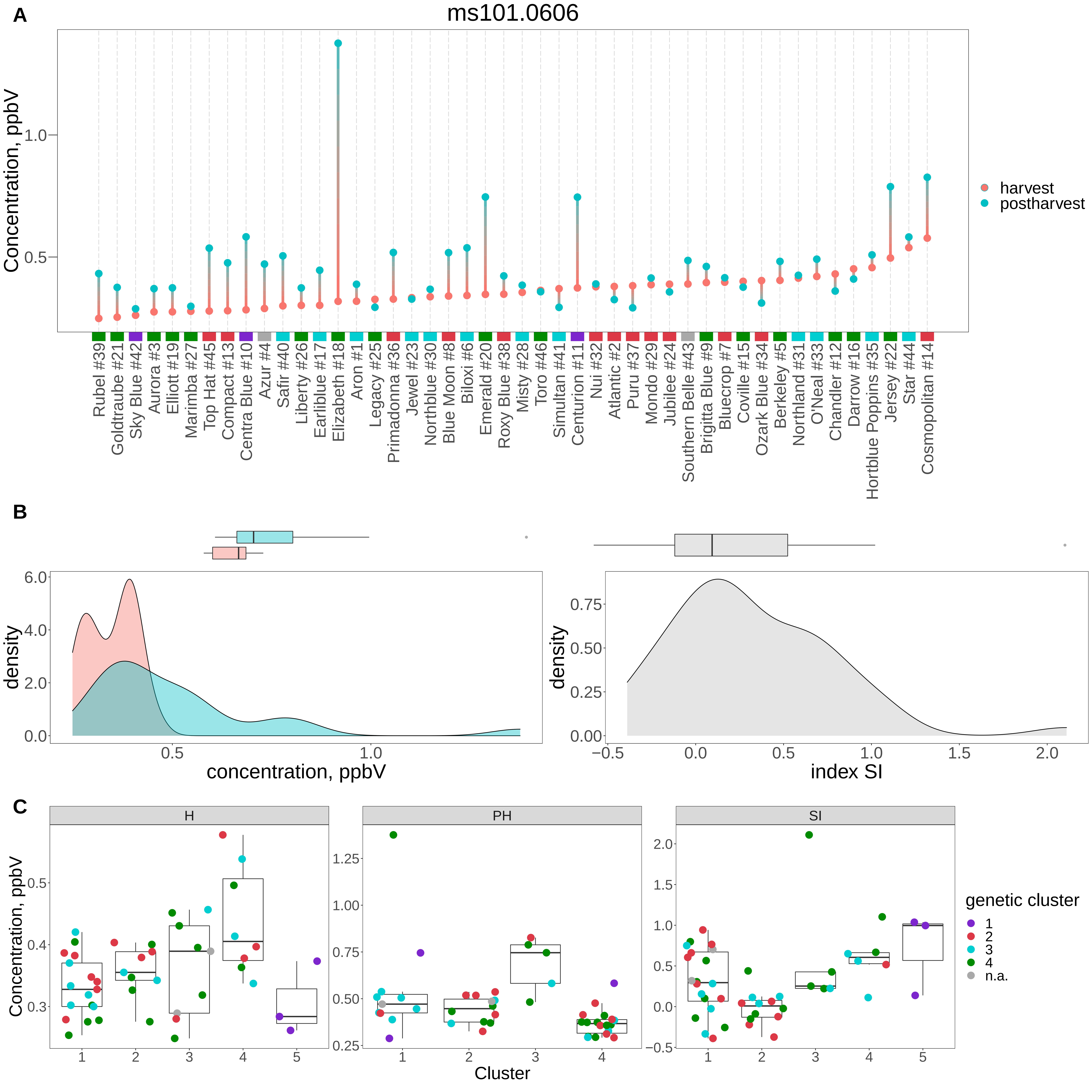
A
ms101.0606
Concentration, ppbV
harvest
postharvest
Rubel #39
Goldtraube #21
Sky Blue #42
Aurora #3
Elliott #19
Marimba #27
Top Hat #45
Compact #13
Centra Blue #10
Azur #4
Safir #40
Liberty #26
Earliblue #17
Elizabeth #18
Aron #1
Legacy #25
Primadonna #36
Jewel #23
Northblue #30
Blue Moon #8
Biloxi #6
Emerald #20
Roxy Blue #38
Misty #28
Toro #46
Simultan #41
Centurion #11
Nui #32
Atlantic #2
Puru #37
Mondo #29
Jubilee #24
Southern Belle #43
Brigitta Blue #9
Bluecrop #7
Coville #15
Ozark Blue #34
Berkeley #5
Northland #31
O'Neal #33
Chandler #12
Darrow #16
Hortblue Poppins #35
Jersey #22
Star #44
Cosmopolitan #14
B
density
concentration, ppbV
index SI
C
H
PH
SI
Concentration, ppbV
Cluster
genetic cluster
1
2
3
4
n.a.

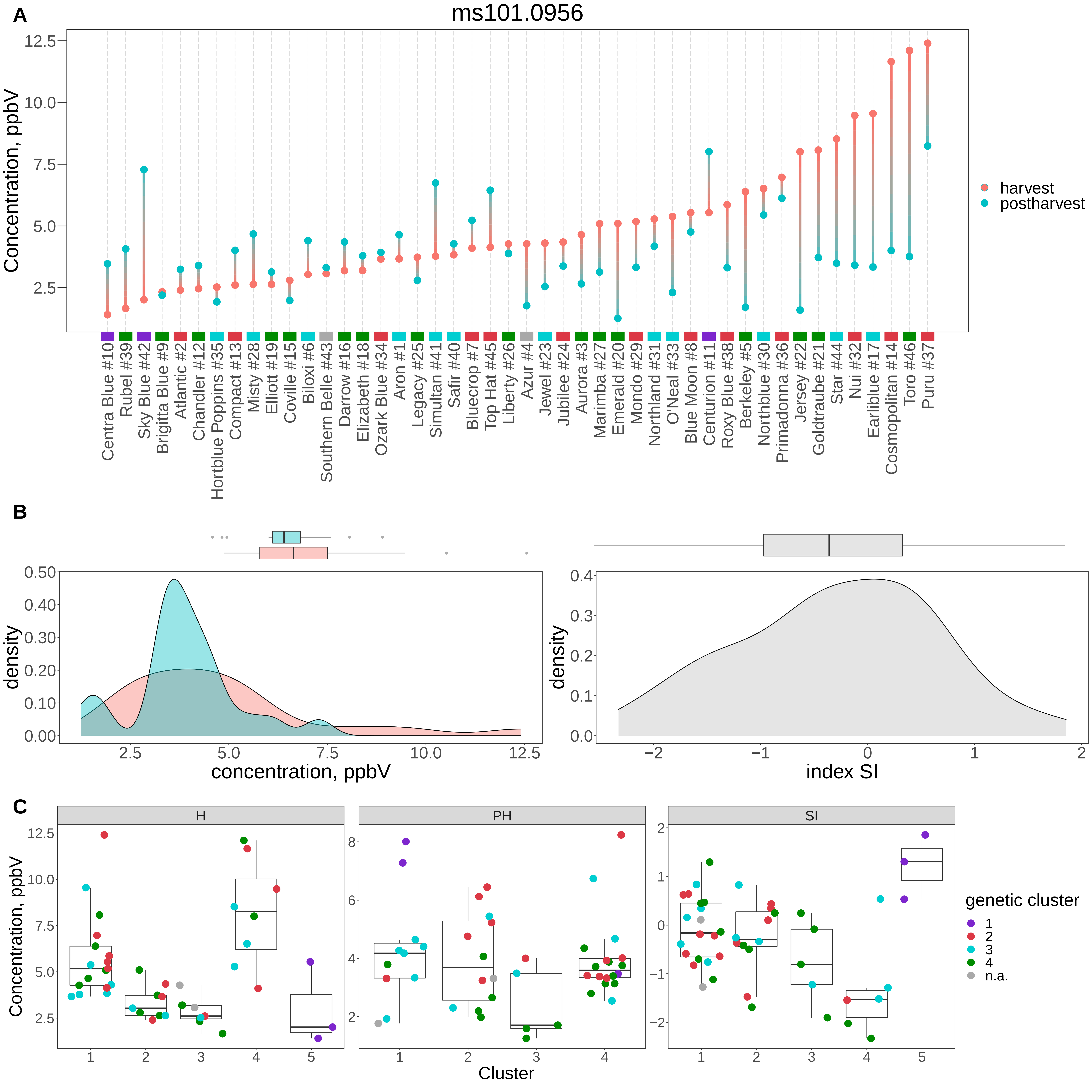
A
ms101.0956
Concentration, ppbV
harvest
postharvest
Centra Blue #10
Rubel #39
Sky Blue #42
Brigitta Blue #9
Atlantic #2
Chandler #12
Hortblue Poppins #35
Compact #13
Misty #28
Elliott #19
Coville #15
Biloxi #6
Southern Belle #43
Darrow #16
Elizabeth #18
Ozark Blue #34
Aron #1
Legacy #25
Simultan #41
Safir #40
Bluecrop #7
Top Hat #45
Liberty #26
Azur #4
Jewel #23
Jubilee #24
Aurora #3
Marimba #27
Emerald #20
Mondo #29
Northland #31
O'Neal #33
Blue Moon #8
Centurion #11
Roxy Blue #38
Berkeley #5
Northblue #30
Primadonna #36
Jersey #22
Goldtraube #21
Star #44
Nui #32
Earliblue #17
Cosmopolitan #14
Toro #46
Puru #37
B
density
concentration, ppbV
index SI
C
H
PH
SI
Cluster
genetic cluster
1
2
3
4
n.a.

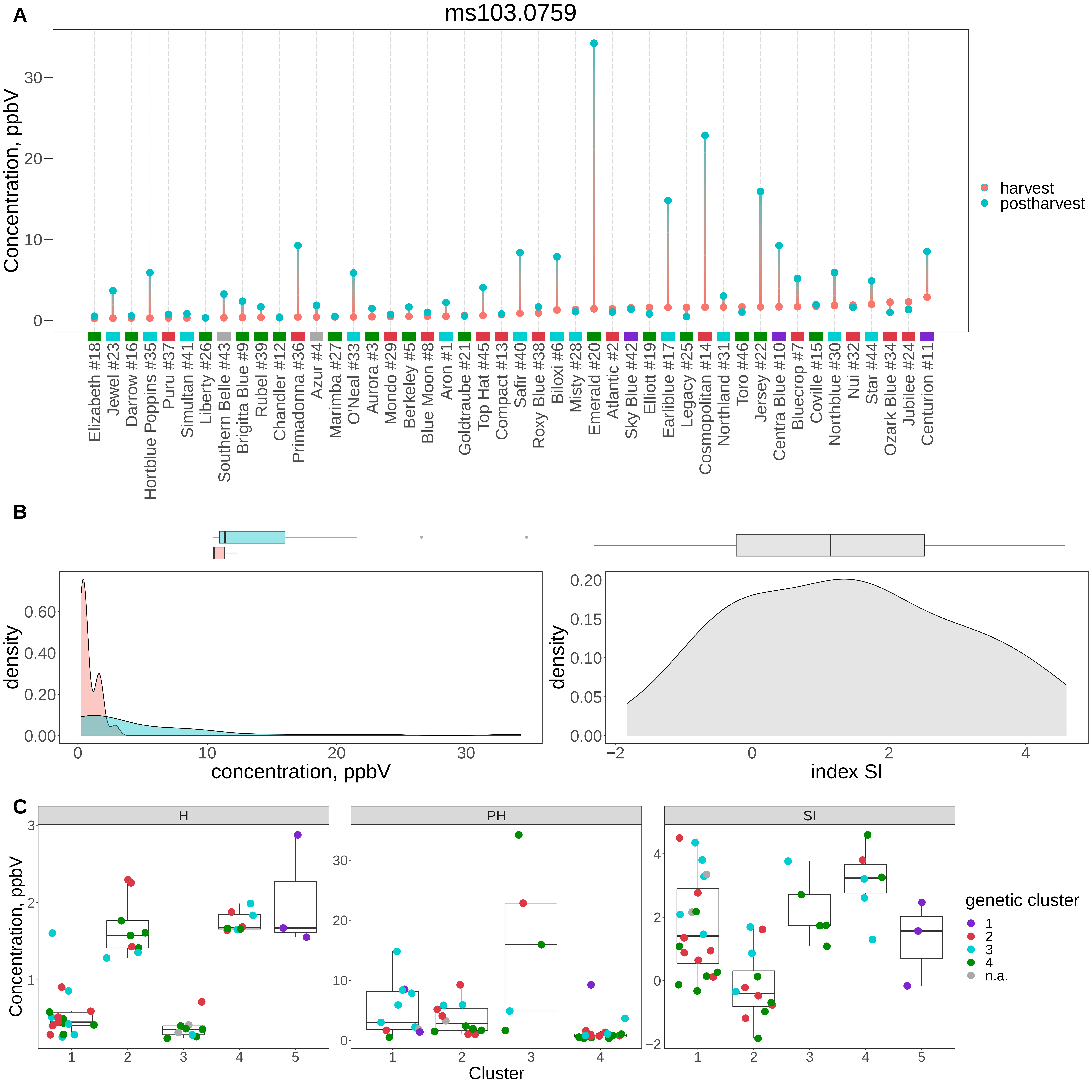
A
ms103.0759
Concentration, ppbV
harvest
postharvest
Elizabeth #18
Jewel #23
Darrow #16
Hortblue Poppins #35
Puru #37
Simultan #41
Liberty #26
Southern Belle #43
Brigitta Blue #9
Rubel #39
Chandler #12
Primadonna #36
Azur #4
Marimba #27
O'Neal #33
Aurora #3
Mondo #29
Berkeley #5
Blue Moon #8
Aron #1
Goldtraube #21
Top Hat #45
Compact #13
Safir #40
Roxy Blue #38
Biloxi #6
Misty #28
Emerald #20
Atlantic #2
Sky Blue #42
Elliott #19
Earliblue #17
Legacy #25
Cosmopolitan #14
Northland #31
Toro #46
Jersey #22
Centra Blue #10
Bluecrop #7
Coville #15
Northblue #30
Nui #32
Star #44
Ozark Blue #34
Jubilee #24
Centurion #11
B
density
concentration, ppbV
index SI
C
H
PH
SI
Cluster
genetic cluster
1
2
3
4
n.a.

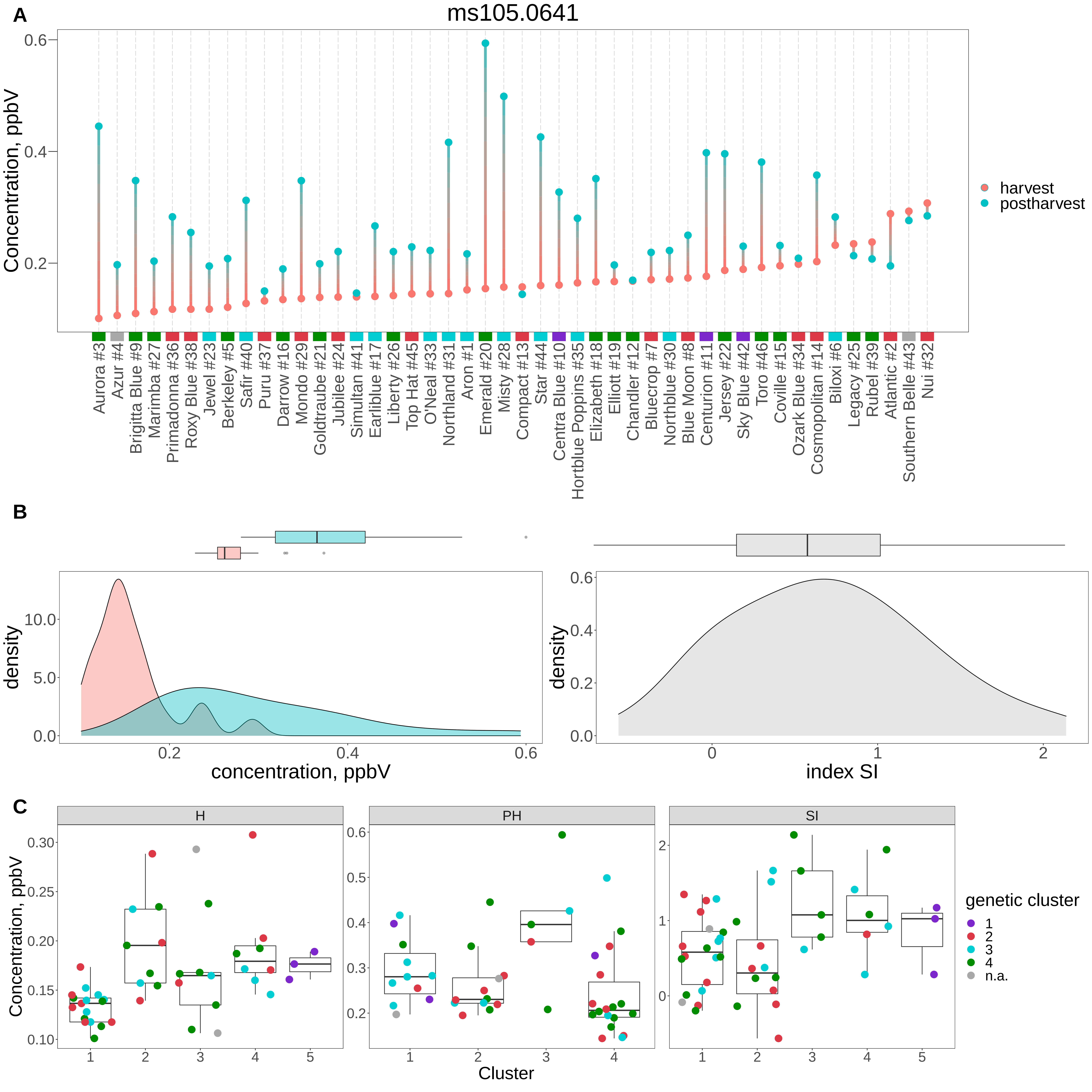
A
ms105.0641
Concentration, ppbV
harvest
postharvest
Aurora #3
Azur #4
Brigitta Blue #9
Marimba #27
Primadonna #36
Roxy Blue #38
Jewel #23
Berkeley #5
Safir #40
Puru #37
Darrow #16
Mondo #29
Goldtraube #21
Jubilee #24
Simultan #41
Earliblue #17
Liberty #26
Top Hat #45
O'Neal #33
Northland #31
Aron #1
Emerald #20
Misty #28
Compact #13
Star #44
Centra Blue #10
Hortblue Poppins #35
Elizabeth #18
Elliott #19
Chandler #12
Bluecrop #7
Northblue #30
Blue Moon #8
Centurion #11
Jersey #22
Sky Blue #42
Toro #46
Coville #15
Ozark Blue #34
Cosmopolitan #14
Biloxi #6
Legacy #25
Rubel #39
Atlantic #2
Southern Belle #43
Nui #32
B
density
concentration, ppbV
index SI
C
H
PH
SI
Concentration, ppbV
Cluster
genetic cluster
1
2
3
4
n.a.

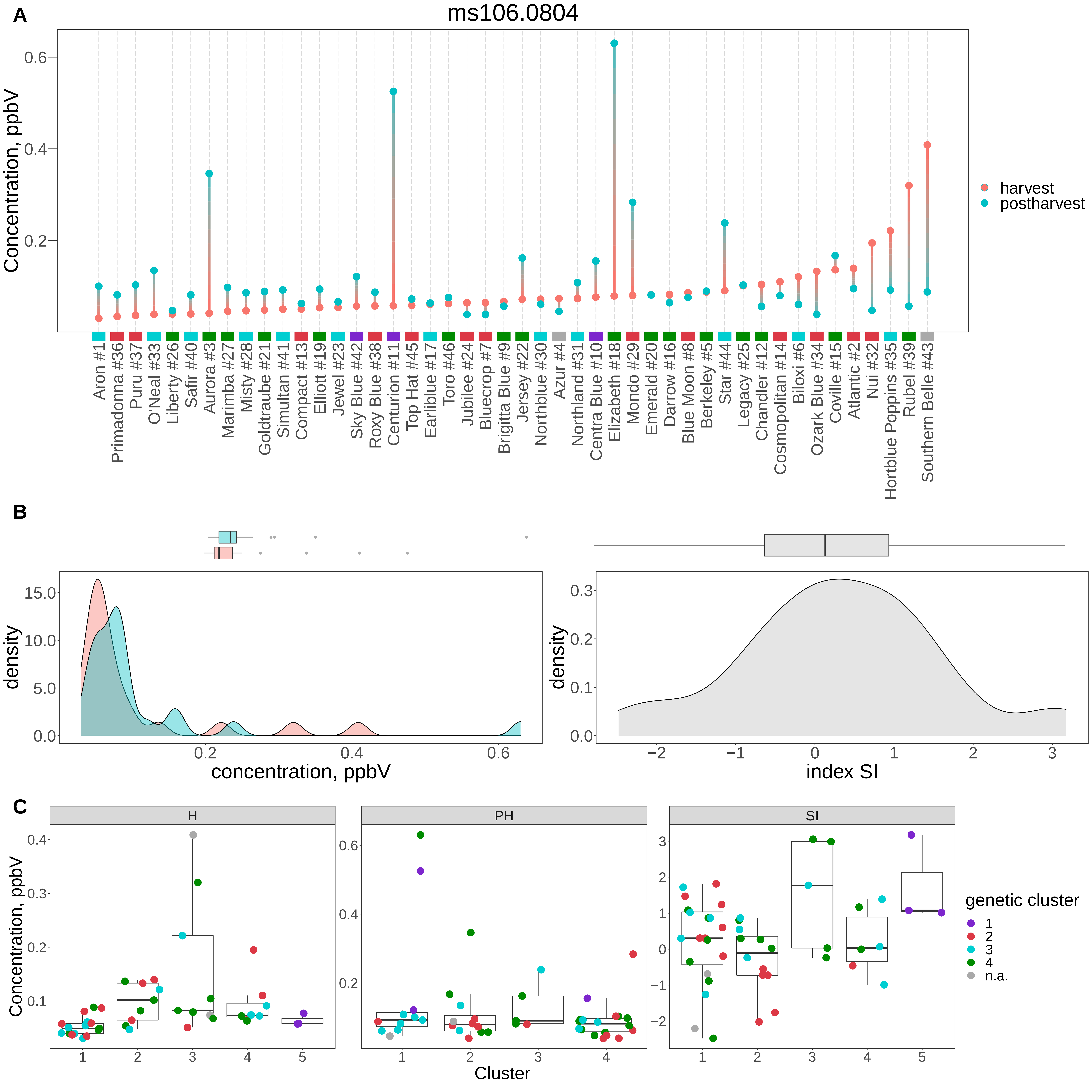

A
ms106.0804
Concentration, ppbV
0.6
0.4
0.2
harvest
postharvest
Aron #1
Primadonna #36
Puru #37
O'Neal #33
Liberty #26
Safir #40
Aurora #3
Marimba #27
Misty #28
Goldtraube #21
Simultan #41
Compact #13
Elliott #19
Jewel #23
Sky Blue #42
Roxy Blue #38
Centurion #11
Top Hat #45
Earliblue #17
Toro #46
Jubilee #24
Bluecrop #7
Brigitta Blue #9
Jersey #22
Northblue #30
Azur #4
Northland #31
Centra Blue #10
Elizabeth #18
Mondo #29
Emerald #20
Darrow #16
Blue Moon #8
Berkeley #5
Star #44
Legacy #25
Chandler #12
Cosmopolitan #14
Biloxi #6
Ozark Blue #34
Coville #15
Atlantic #2
Nui #32
Hortblue Poppins #35
Rubel #39
Southern Belle #43
B
density
15.0
10.0
5.0
0.0
0.2
0.4
0.6
concentration, ppbV
density
0.3
0.2
0.1
0.0
−2
−1
0
1
2
3
index SI
C
H
PH
SI
Concentration, ppbV
Cluster
genetic cluster
1
2
3
4
n.a.

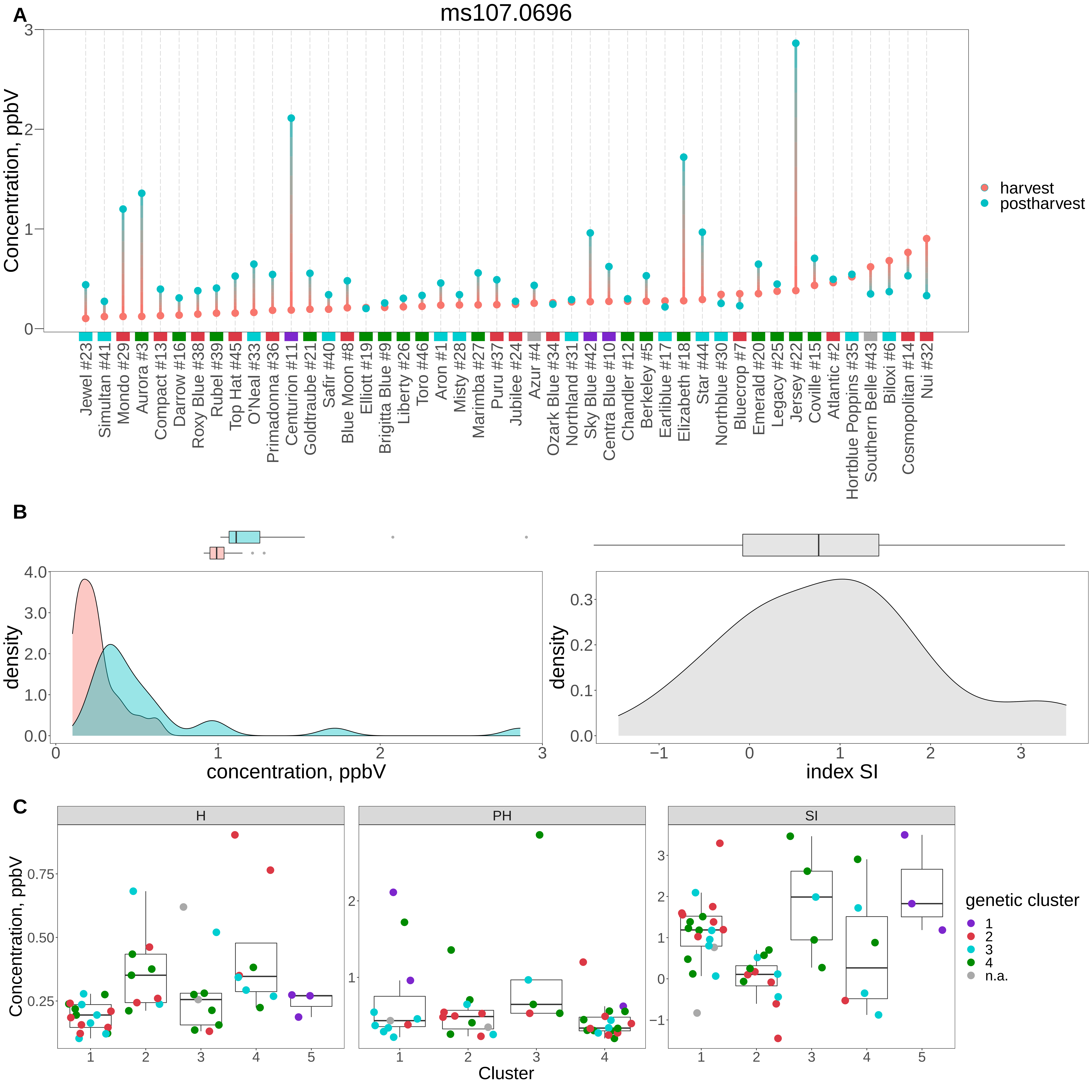
A
ms107.0696
Concentration, ppbV
harvest
postharvest
Jewel #23
Simultan #41
Mondo #29
Aurora #3
Compact #13
Darrow #16
Roxy Blue #38
Rubel #39
Top Hat #45
O'Neal #33
Primadonna #36
Centurion #11
Goldtraube #21
Safir #40
Blue Moon #8
Elliott #19
Brigitta Blue #9
Liberty #26
Toro #46
Aron #1
Misty #28
Marimba #27
Puru #37
Jubilee #24
Azur #4
Ozark Blue #34
Northland #31
Sky Blue #42
Centra Blue #10
Chandler #12
Berkeley #5
Earliblue #17
Elizabeth #18
Star #44
Northblue #30
Bluecrop #7
Emerald #20
Legacy #25
Jersey #22
Coville #15
Atlantic #2
Hortblue Poppins #35
Southern Belle #43
Biloxi #6
Cosmopolitan #14
Nui #32
B
density
concentration, ppbV
density
index SI
C
H
PH
SI
Concentration, ppbV
Cluster
genetic cluster
1
2
3
4
n.a.

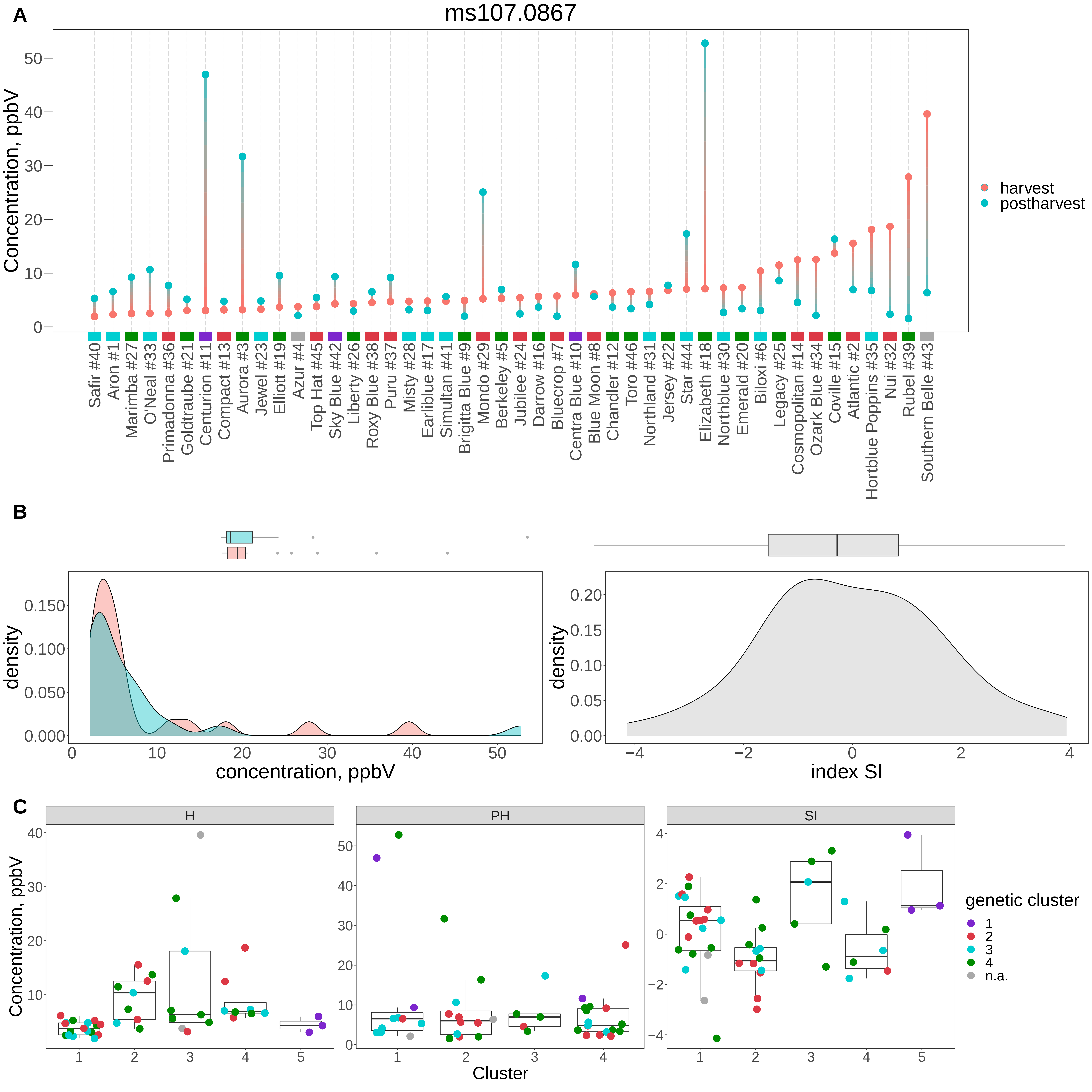

A
ms107.0867
Concentration, ppbV
0
10
20
30
40
50
harvest
postharvest
Safir #40
Aron #1
Marimba #27
O'Neal #33
Primadonna #36
Goldtraube #21
Centurion #11
Compact #13
Aurora #3
Jewel #23
Elliott #19
Azur #4
Top Hat #45
Sky Blue #42
Liberty #26
Roxy Blue #38
Puru #37
Misty #28
Earliblue #17
Simultan #41
Brigitta Blue #9
Mondo #29
Berkeley #5
Jubilee #24
Darrow #16
Bluecrop #7
Centra Blue #10
Blue Moon #8
Chandler #12
Toro #46
Northland #31
Jersey #22
Star #44
Elizabeth #18
Northblue #30
Emerald #20
Biloxi #6
Legacy #25
Cosmopolitan #14
Ozark Blue #34
Coville #15
Atlantic #2
Hortblue Poppins #35
Nui #32
Rubel #39
Southern Belle #43
B
density
0.000
0.050
0.100
0.150
0
10
20
30
40
50
concentration, ppbV
density
0.00
0.05
0.10
0.15
0.20
−4
−2
0
2
4
index SI
C
H
PH
SI
Concentration, ppbV
Cluster
genetic cluster
1
2
3
4
n.a.

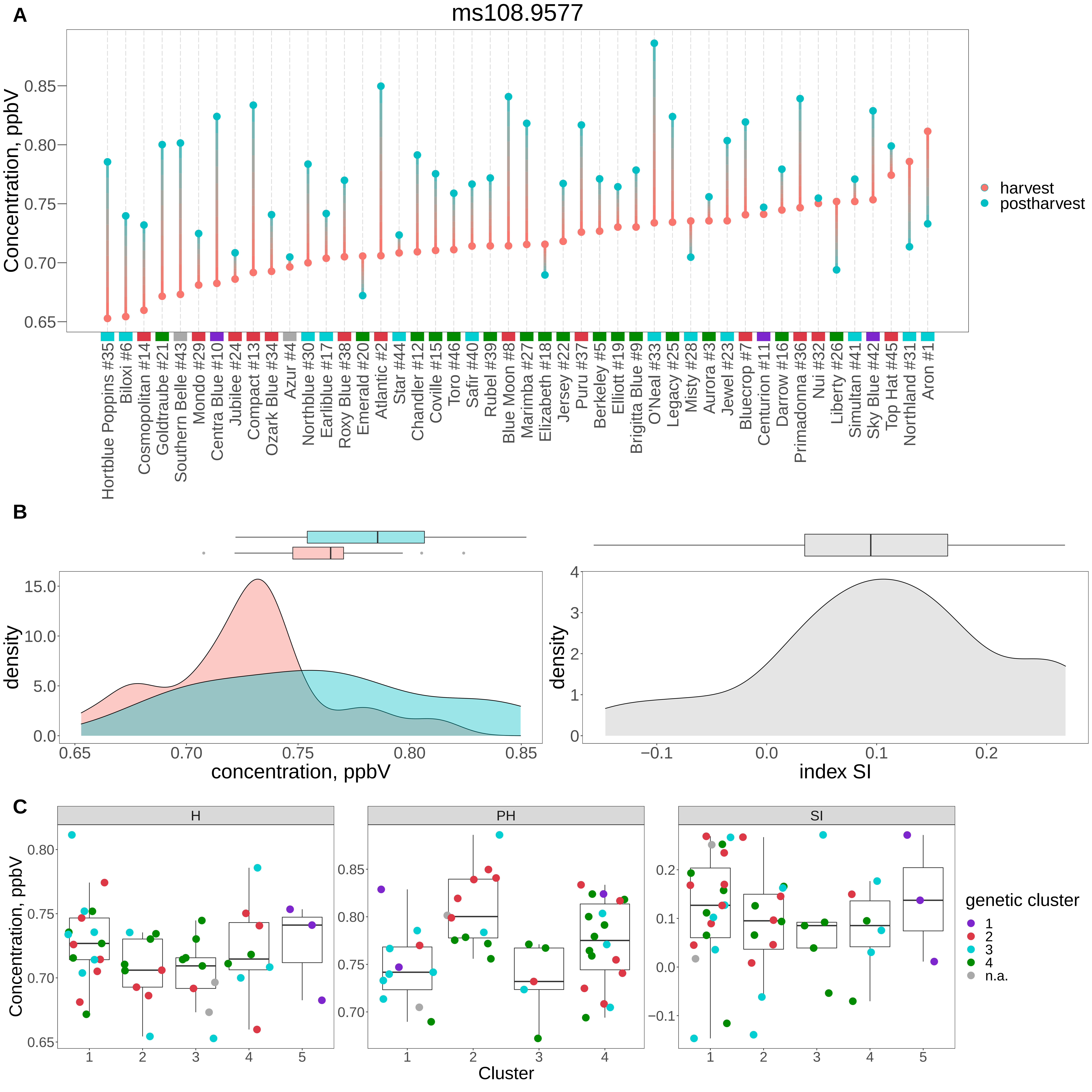

A
ms108.9577
Concentration, ppbV
harvest
postharvest
Hortblue Poppins #35
Biloxi #6
Cosmopolitan #14
Goldtraube #21
Southern Belle #43
Mondo #29
Centra Blue #10
Jubilee #24
Compact #13
Ozark Blue #34
Azur #4
Northblue #30
Earliblue #17
Roxy Blue #38
Emerald #20
Atlantic #2
Star #44
Chandler #12
Coville #15
Toro #46
Safir #40
Rubel #39
Blue Moon #8
Marimba #27
Elizabeth #18
Jersey #22
Puru #37
Berkeley #5
Elliott #19
Brigitta Blue #9
O'Neal #33
Legacy #25
Misty #28
Aurora #3
Jewel #23
Bluecrop #7
Centurion #11
Darrow #16
Primadonna #36
Nui #32
Liberty #26
Simultan #41
Sky Blue #42
Top Hat #45
Northland #31
Aron #1
B
density
concentration, ppbV
index SI
C
H
PH
SI
Concentration, ppbV
Cluster
genetic cluster
1
2
3
4
n.a.

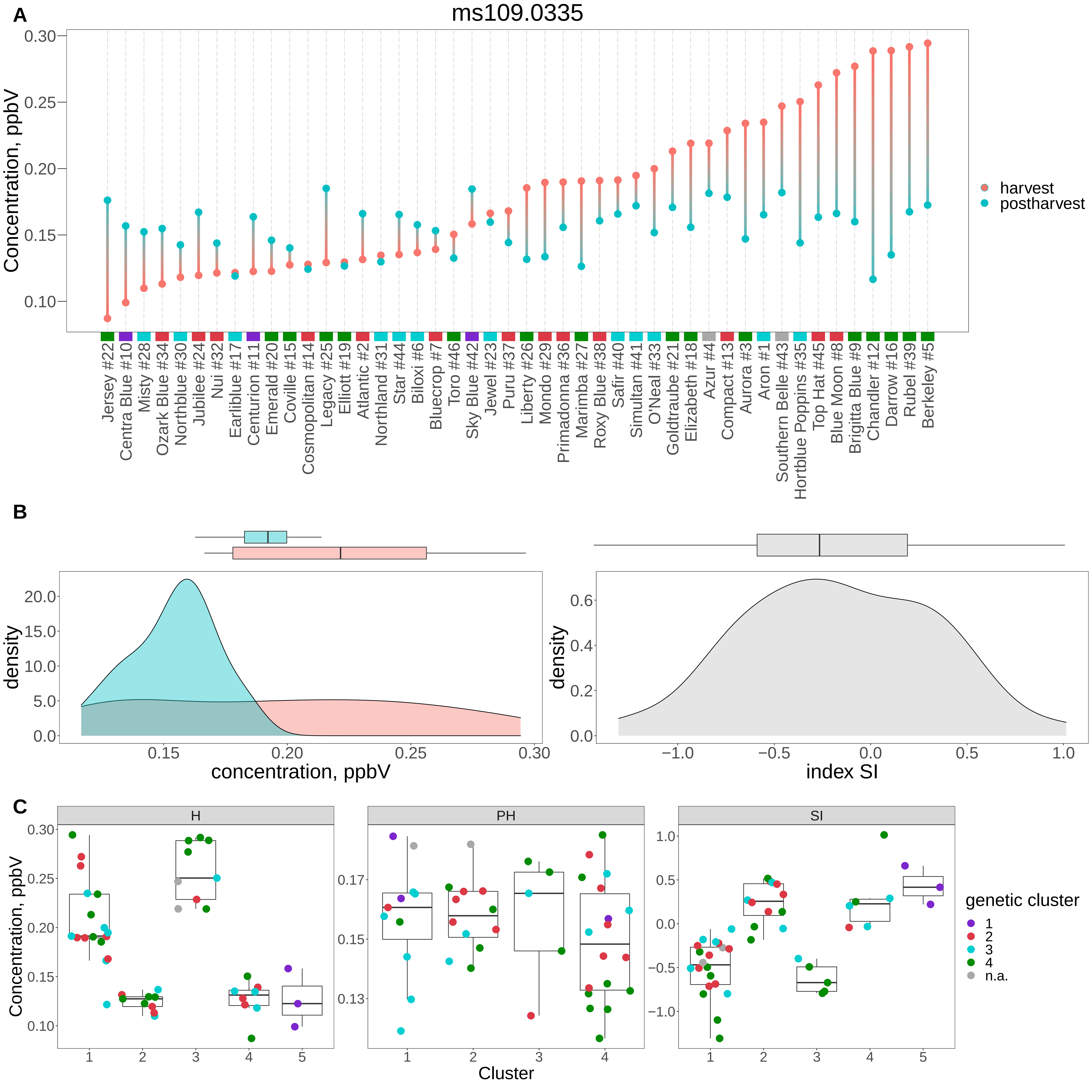

A
ms109.0335
Concentration, ppbV
harvest
postharvest
Jersey #22
Centra Blue #10
Misty #28
Ozark Blue #34
Northblue #30
Jubilee #24
Nui #32
Earliblue #17
Centurion #11
Emerald #20
Coville #15
Cosmopolitan #14
Legacy #25
Elliott #19
Atlantic #2
Northland #31
Star #44
Biloxi #6
Bluecrop #7
Toro #46
Sky Blue #42
Jewel #23
Puru #37
Liberty #26
Mondo #29
Primadonna #36
Marimba #27
Roxy Blue #38
Safir #40
Simultan #41
O'Neal #33
Goldtraube #21
Elizabeth #18
Azur #4
Compact #13
Aurora #3
Aron #1
Southern Belle #43
Hortblue Poppins #35
Top Hat #45
Blue Moon #8
Brigitta Blue #9
Chandler #12
Darrow #16
Rubel #39
Berkeley #5
B
density
concentration, ppbV
index SI
C
H
PH
SI
Concentration, ppbV
Cluster
genetic cluster
1
2
3
4
n.a.

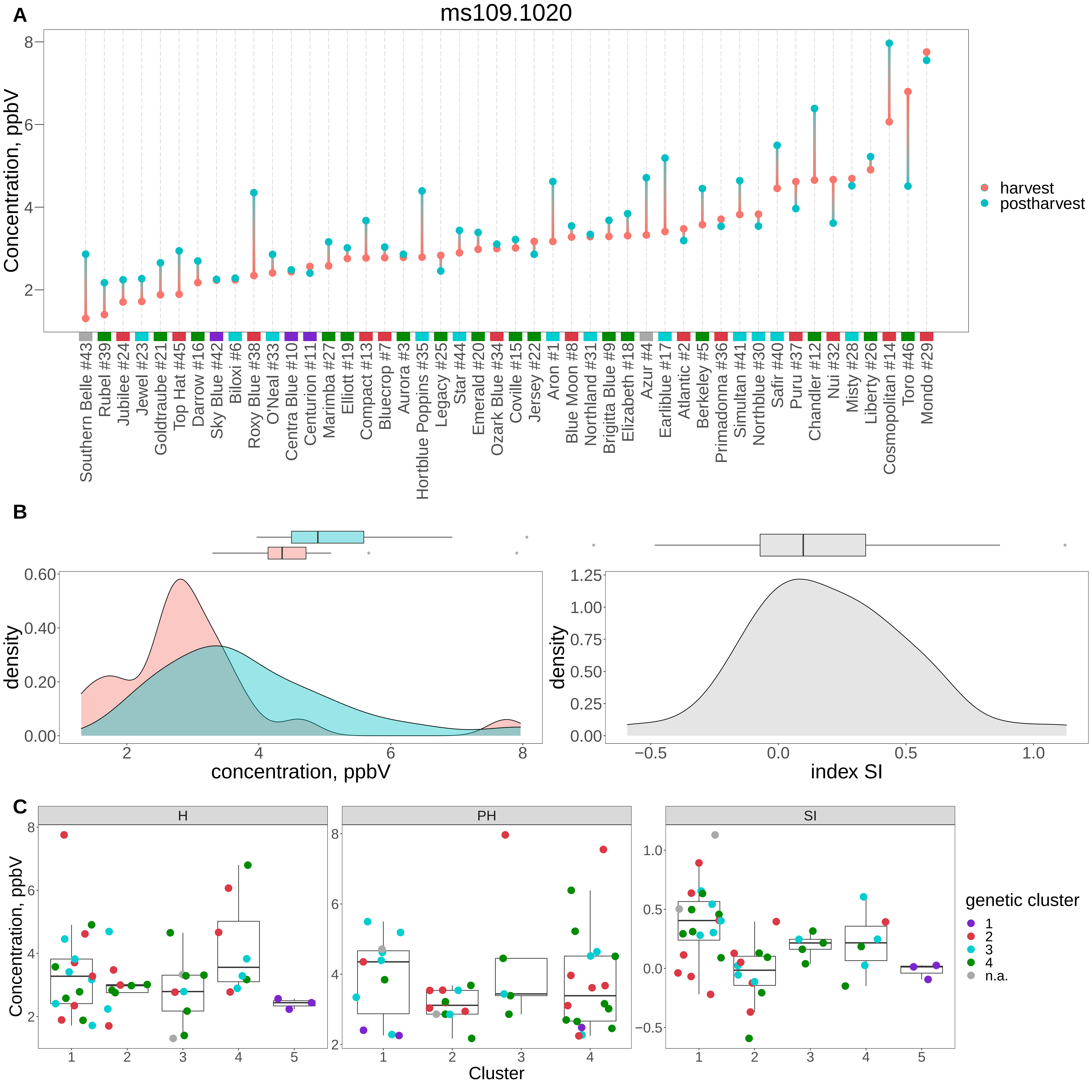
A
ms109.1020
Concentration, ppbV
harvest
postharvest
Southern Belle #43
Rubel #39
Jubilee #24
Jewel #23
Goldtraube #21
Top Hat #45
Darrow #16
Sky Blue #42
Biloxi #6
Roxy Blue #38
O'Neal #33
Centra Blue #10
Centurion #11
Marimba #27
Elliott #19
Compact #13
Bluecrop #7
Aurora #3
Hortblue Poppins #35
Legacy #25
Star #44
Emerald #20
Ozark Blue #34
Coville #15
Jersey #22
Aron #1
Blue Moon #8
Northland #31
Brigitta Blue #9
Elizabeth #18
Azur #4
Earliblue #17
Atlantic #2
Berkeley #5
Primadonna #36
Simultan #41
Northblue #30
Safir #40
Puru #37
Chandler #12
Nui #32
Misty #28
Liberty #26
Cosmopolitan #14
Toro #46
Mondo #29
B
density
concentration, ppbV
index SI
C
H
PH
SI
Cluster
genetic cluster
1
2
3
4
n.a.

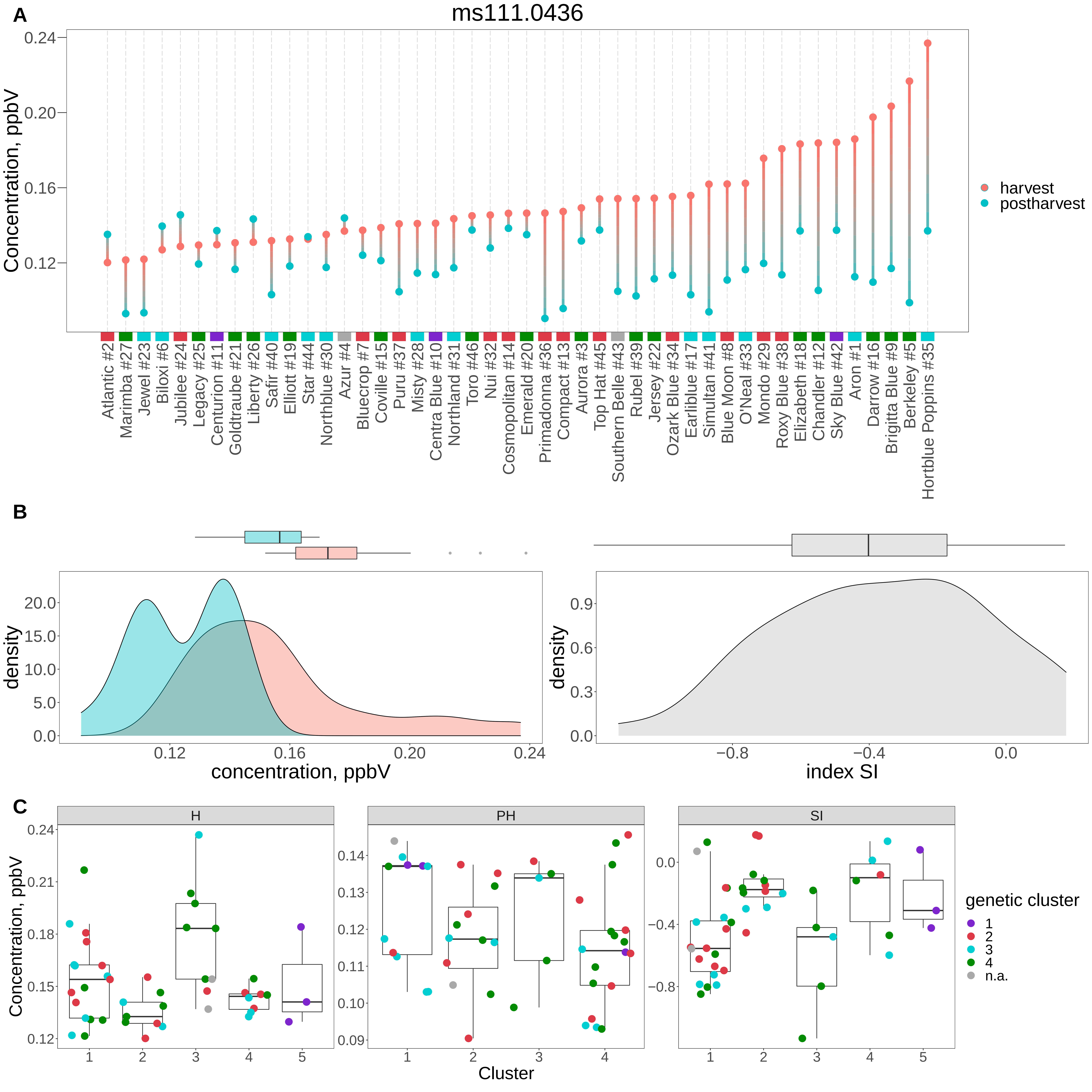

A
ms111.0436
Concentration, ppbV
harvest
postharvest
Atlantic #2
Marimba #27
Jewel #23
Biloxi #6
Jubilee #24
Legacy #25
Centurion #11
Goldtraube #21
Liberty #26
Safir #40
Elliott #19
Star #44
Northblue #30
Azur #4
Bluecrop #7
Coville #15
Puru #37
Misty #28
Centra Blue #10
Northland #31
Toro #46
Nui #32
Cosmopolitan #14
Emerald #20
Primadonna #36
Compact #13
Aurora #3
Top Hat #45
Southern Belle #43
Rubel #39
Jersey #22
Ozark Blue #34
Earliblue #17
Simultan #41
Blue Moon #8
O'Neal #33
Mondo #29
Roxy Blue #38
Elizabeth #18
Chandler #12
Sky Blue #42
Aron #1
Darrow #16
Brigitta Blue #9
Berkeley #5
Hortblue Poppins #35
B
density
concentration, ppbV
index SI
C
H
PH
SI
Concentration, ppbV
Cluster
genetic cluster
1
2
3
4
n.a.

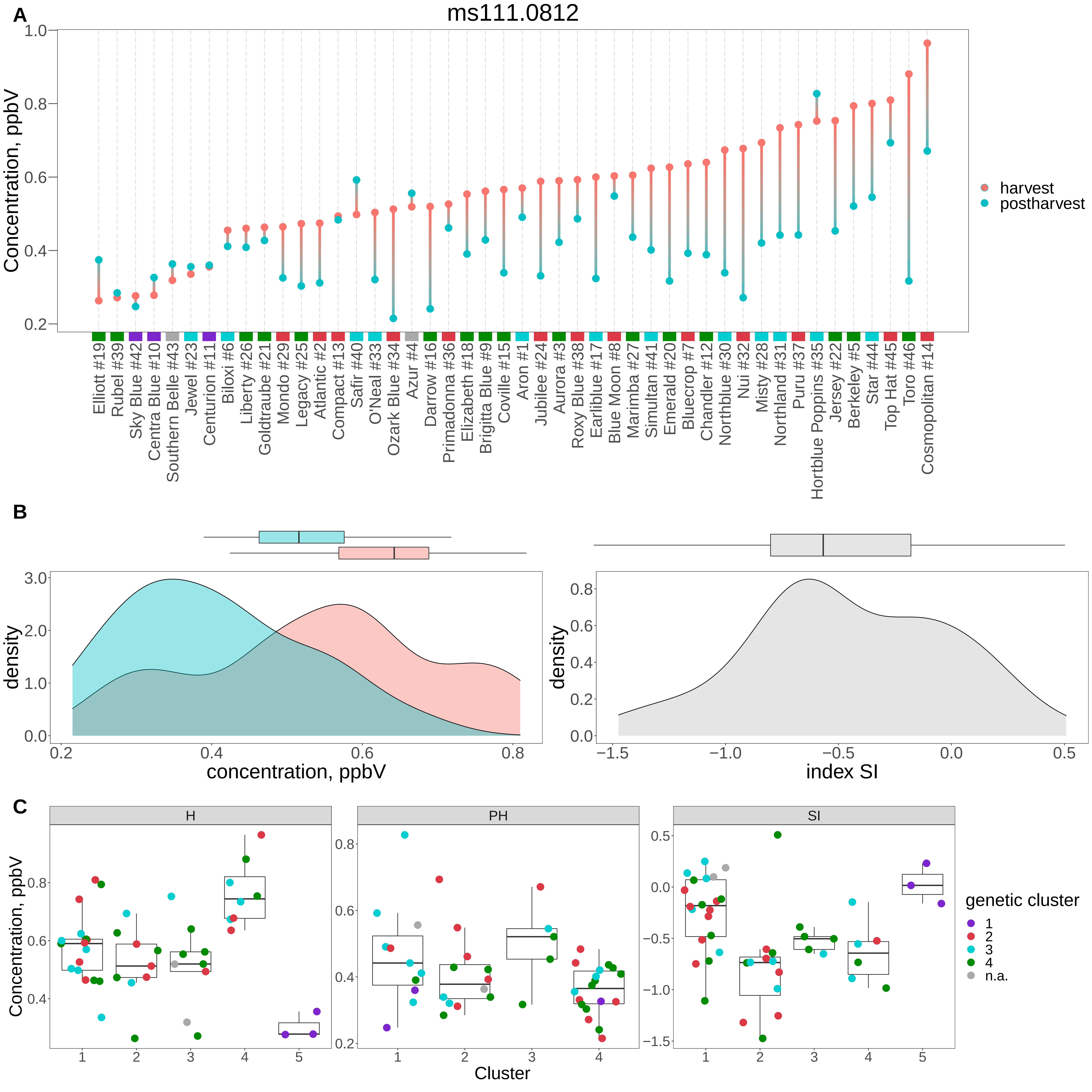
A
ms111.0812
Concentration, ppbV
harvest
postharvest
Elliott #19
Rubel #39
Sky Blue #42
Centra Blue #10
Southern Belle #43
Jewel #23
Centurion #11
Biloxi #6
Liberty #26
Goldtraube #21
Mondo #29
Legacy #25
Atlantic #2
Compact #13
Safir #40
O'Neal #33
Ozark Blue #34
Azur #4
Darrow #16
Primadonna #36
Elizabeth #18
Brigitta Blue #9
Coville #15
Aron #1
Jubilee #24
Aurora #3
Roxy Blue #38
Earliblue #17
Blue Moon #8
Marimba #27
Simultan #41
Emerald #20
Bluecrop #7
Chandler #12
Northblue #30
Nui #32
Misty #28
Northland #31
Puru #37
Hortblue Poppins #35
Jersey #22
Berkeley #5
Star #44
Top Hat #45
Toro #46
Cosmopolitan #14
B
density
concentration, ppbV
density
index SI
C
H
PH
SI
Concentration, ppbV
Cluster
genetic cluster
1
2
3
4
n.a.

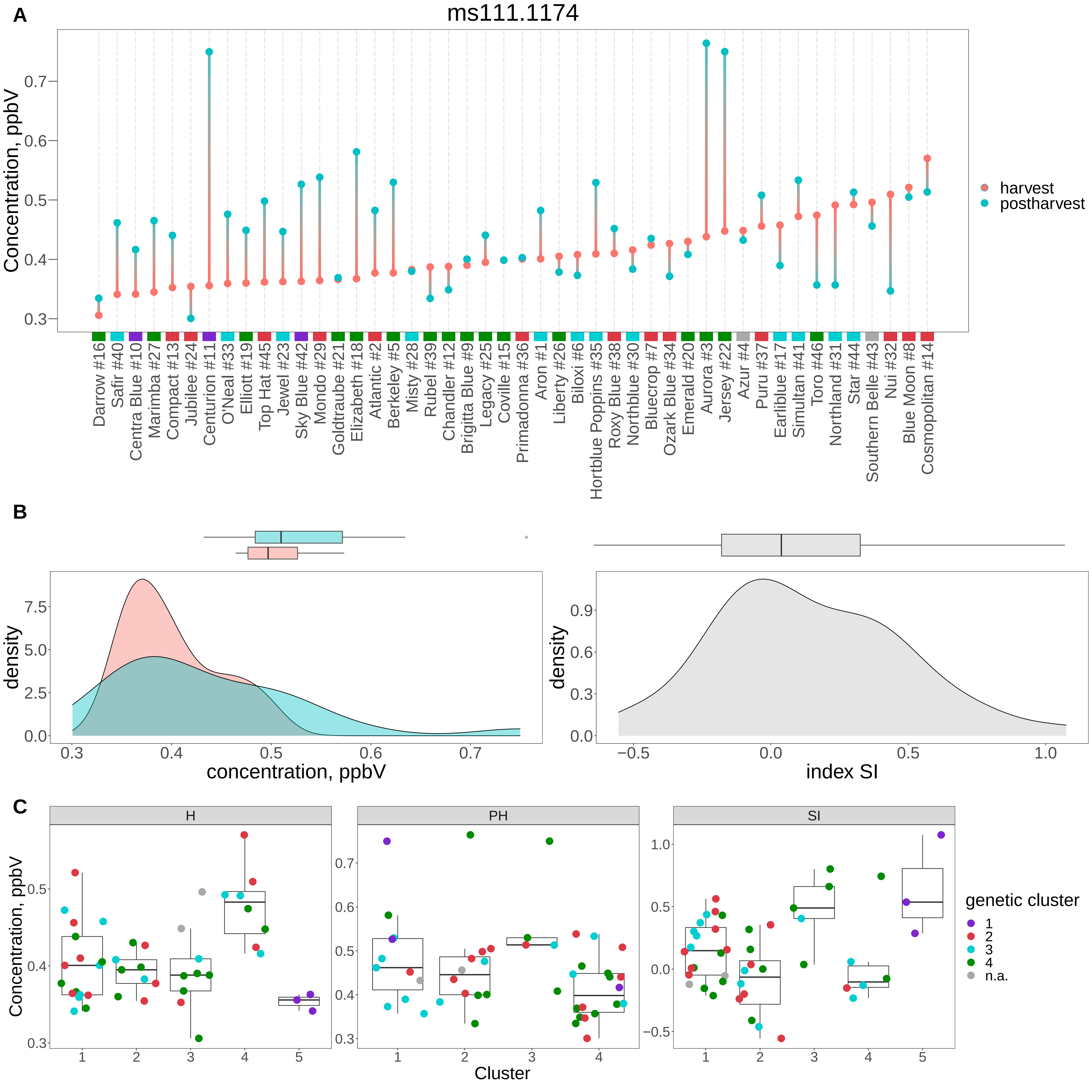
A
ms111.1174
Concentration, ppbV
harvest
postharvest
Darrow #16
Safir #40
Centra Blue #10
Marimba #27
Compact #13
Jubilee #24
Centurion #11
O'Neal #33
Elliott #19
Top Hat #45
Jewel #23
Sky Blue #42
Mondo #29
Goldtraube #21
Elizabeth #18
Atlantic #2
Berkeley #5
Misty #28
Rubel #39
Chandler #12
Brigitta Blue #9
Legacy #25
Coville #15
Primadonna #36
Aron #1
Liberty #26
Biloxi #6
Hortblue Poppins #35
Roxy Blue #38
Northblue #30
Bluecrop #7
Ozark Blue #34
Emerald #20
Aurora #3
Jersey #22
Azur #4
Puru #37
Earliblue #17
Simultan #41
Toro #46
Northland #31
Star #44
Southern Belle #43
Nui #32
Blue Moon #8
Cosmopolitan #14
B
density
concentration, ppbV
index SI
C
H
PH
SI
Concentration, ppbV
Cluster
genetic cluster
1
2
3
4
n.a.

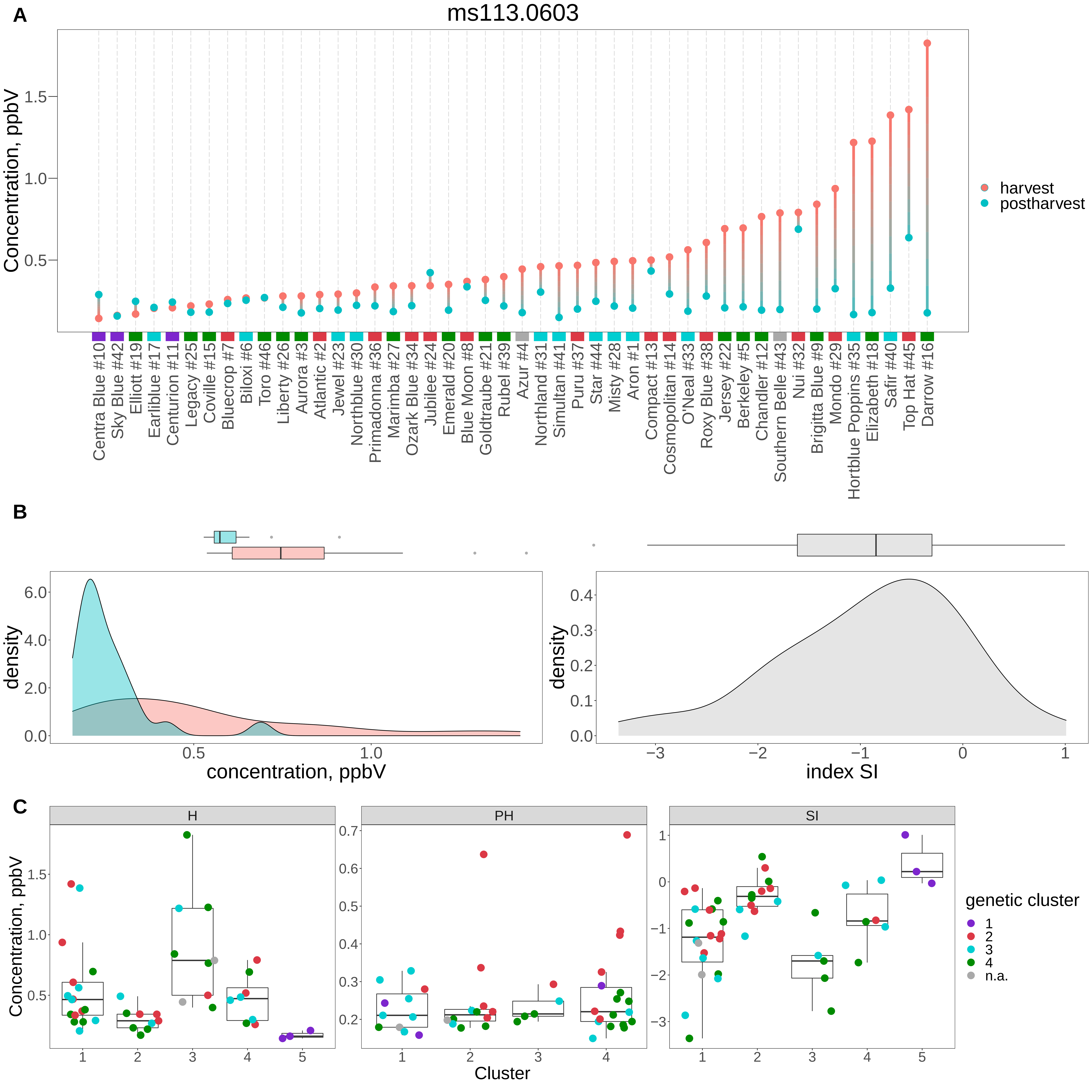

A
ms113.0603
Concentration, ppbV
harvest
postharvest
Centra Blue #10
Sky Blue #42
Elliott #19
Earliblue #17
Centurion #11
Legacy #25
Coville #15
Bluecrop #7
Biloxi #6
Toro #46
Liberty #26
Aurora #3
Atlantic #2
Jewel #23
Northblue #30
Primadonna #36
Marimba #27
Ozark Blue #34
Jubilee #24
Emerald #20
Blue Moon #8
Goldtraube #21
Rubel #39
Azur #4
Northland #31
Simultan #41
Puru #37
Star #44
Misty #28
Aron #1
Compact #13
Cosmopolitan #14
O'Neal #33
Roxy Blue #38
Jersey #22
Berkeley #5
Chandler #12
Southern Belle #43
Nui #32
Brigitta Blue #9
Mondo #29
Hortblue Poppins #35
Elizabeth #18
Safir #40
Top Hat #45
Darrow #16
B
density
concentration, ppbV
index SI
C
H
PH
SI
Concentration, ppbV
Cluster
genetic cluster
1
2
3
4
n.a.

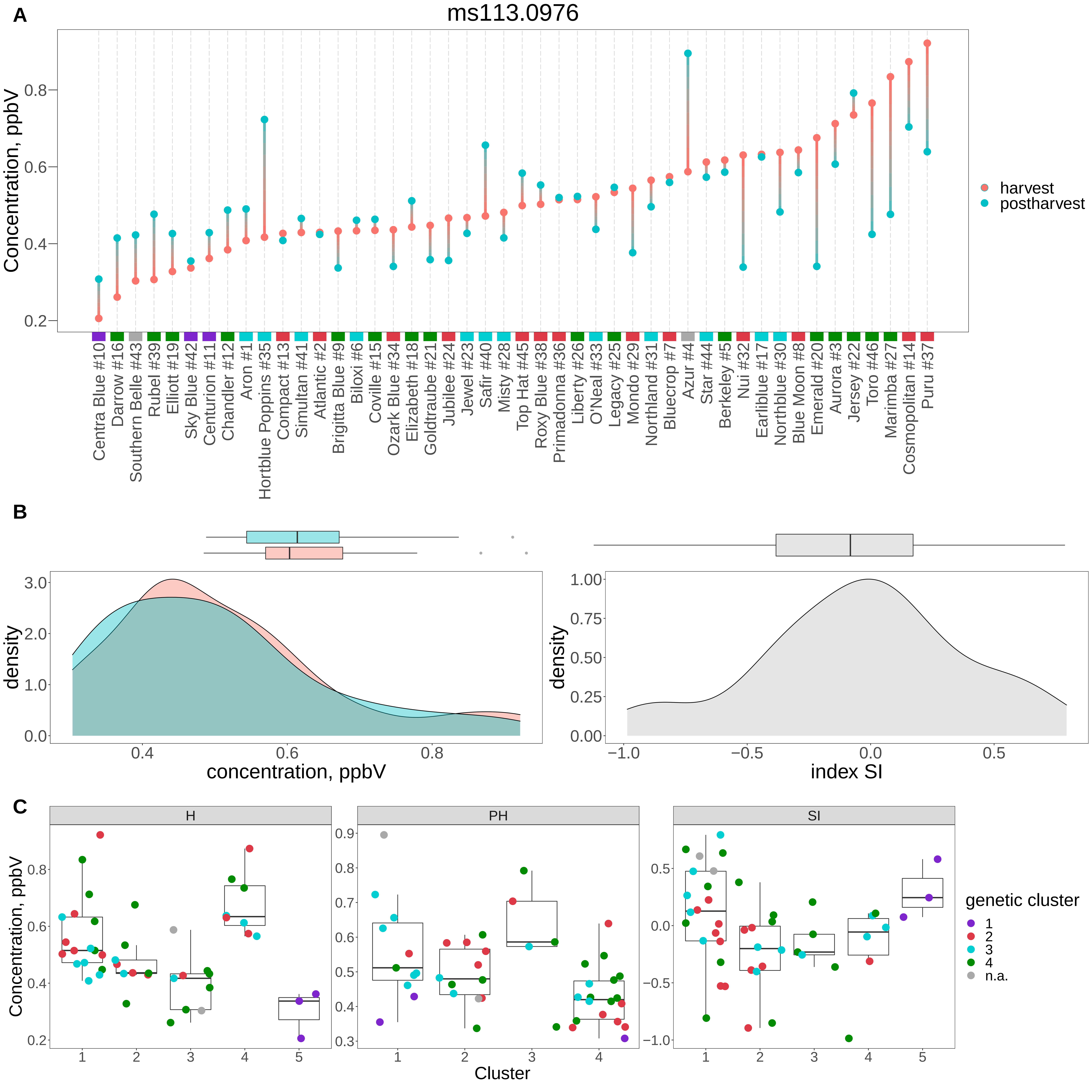
A
ms113.0976
Concentration, ppbV
harvest
postharvest
Centra Blue #10
Darrow #16
Southern Belle #43
Rubel #39
Elliott #19
Sky Blue #42
Centurion #11
Chandler #12
Aron #1
Hortblue Poppins #35
Compact #13
Simultan #41
Atlantic #2
Brigitta Blue #9
Biloxi #6
Coville #15
Ozark Blue #34
Elizabeth #18
Goldtraube #21
Jubilee #24
Jewel #23
Safir #40
Misty #28
Top Hat #45
Roxy Blue #38
Primadonna #36
Liberty #26
O'Neal #33
Legacy #25
Mondo #29
Northland #31
Bluecrop #7
Azur #4
Star #44
Berkeley #5
Nui #32
Earliblue #17
Northblue #30
Blue Moon #8
Emerald #20
Aurora #3
Jersey #22
Toro #46
Marimba #27
Cosmopolitan #14
Puru #37
B
density
concentration, ppbV
density
index SI
C
H
PH
SI
Concentration, ppbV
Cluster
genetic cluster
1
2
3
4
n.a.

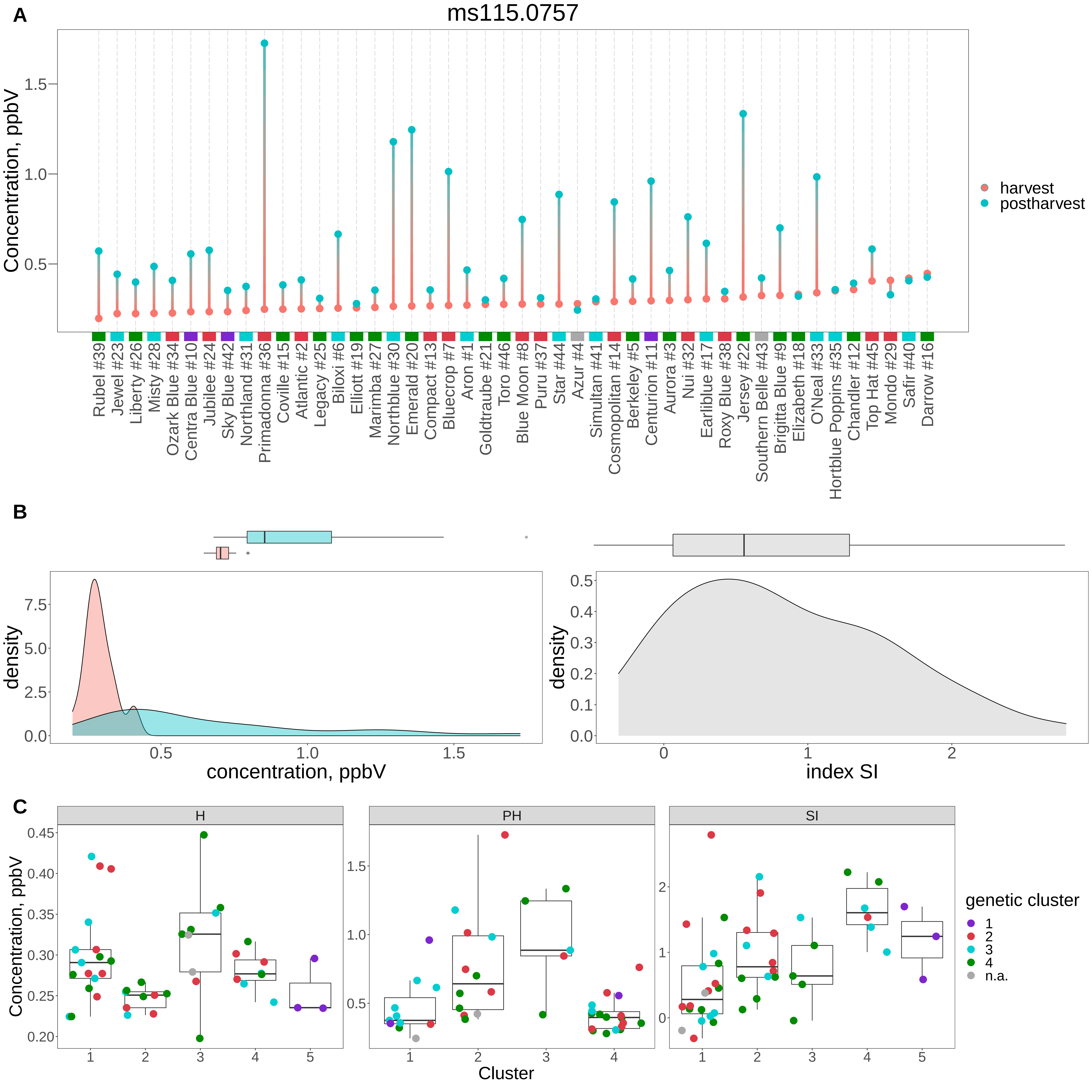
A
ms115.0757
Concentration, ppbV
harvest
postharvest
Rubel #39
Jewel #23
Liberty #26
Misty #28
Ozark Blue #34
Centra Blue #10
Jubilee #24
Sky Blue #42
Northland #31
Primadonna #36
Coville #15
Atlantic #2
Legacy #25
Biloxi #6
Elliott #19
Marimba #27
Northblue #30
Emerald #20
Compact #13
Bluecrop #7
Aron #1
Goldtraube #21
Toro #46
Blue Moon #8
Puru #37
Star #44
Azur #4
Simultan #41
Cosmopolitan #14
Berkeley #5
Centurion #11
Aurora #3
Nui #32
Earliblue #17
Roxy Blue #38
Jersey #22
Southern Belle #43
Brigitta Blue #9
Elizabeth #18
O'Neal #33
Hortblue Poppins #35
Chandler #12
Top Hat #45
Mondo #29
Safir #40
Darrow #16
B
density
concentration, ppbV
density
index SI
C
H
PH
SI
Concentration, ppbV
Cluster
genetic cluster
1
2
3
4
n.a.

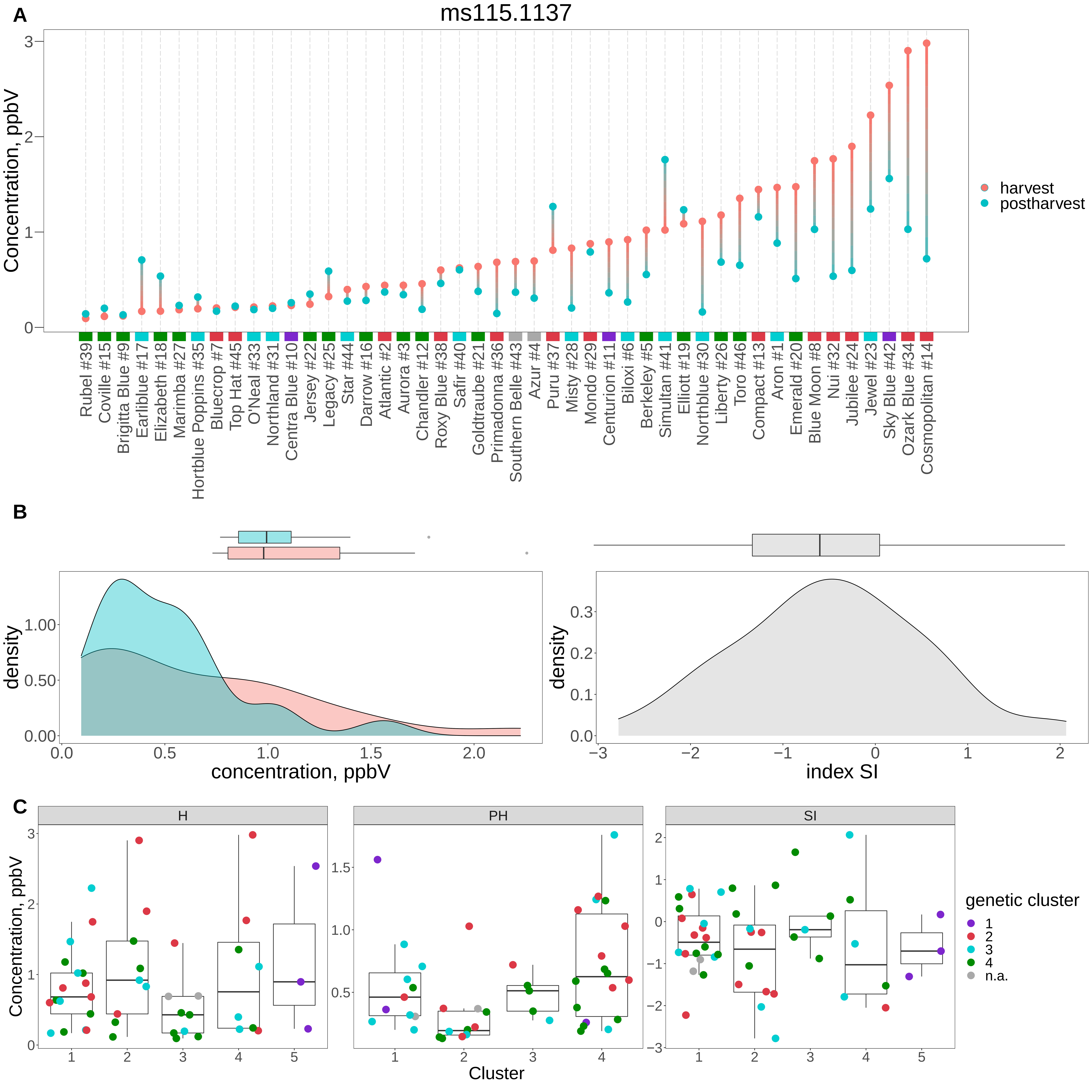

A
ms115.1137
Concentration, ppbV
harvest
postharvest
Rubel #39
Coville #15
Brigitta Blue #9
Earliblue #17
Elizabeth #18
Marimba #27
Hortblue Poppins #35
Bluecrop #7
Top Hat #45
O'Neal #33
Northland #31
Centra Blue #10
Jersey #22
Legacy #25
Star #44
Darrow #16
Atlantic #2
Aurora #3
Chandler #12
Roxy Blue #38
Safir #40
Goldtraube #21
Primadonna #36
Southern Belle #43
Azur #4
Puru #37
Misty #28
Mondo #29
Centurion #11
Biloxi #6
Berkeley #5
Simultan #41
Elliott #19
Northblue #30
Liberty #26
Toro #46
Compact #13
Aron #1
Emerald #20
Blue Moon #8
Nui #32
Jubilee #24
Jewel #23
Sky Blue #42
Ozark Blue #34
Cosmopolitan #14
B
density
concentration, ppbV
density
index SI
C
H
PH
SI
Concentration, ppbV
Cluster
genetic cluster
1
2
3
4
n.a.

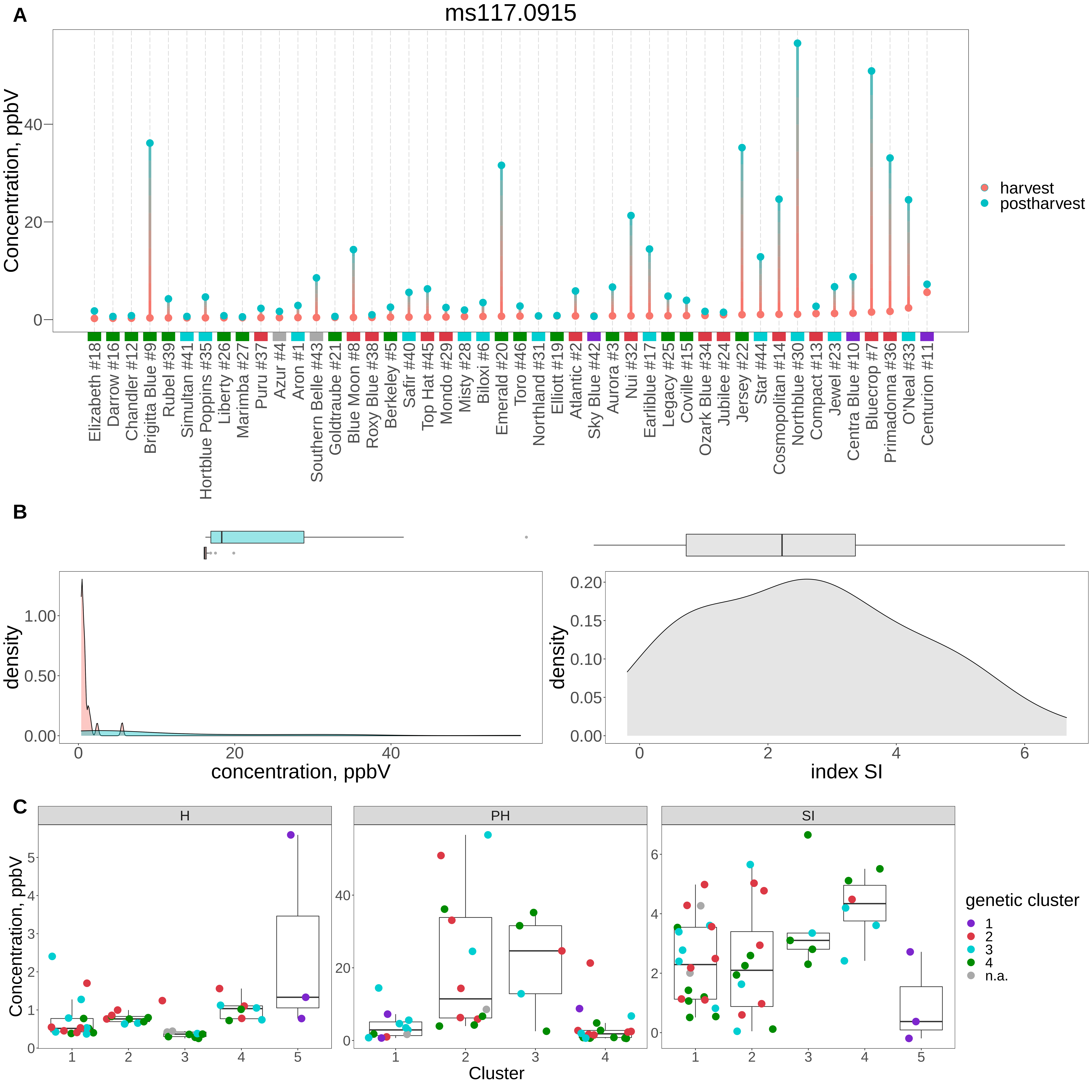
A
ms117.0915
Concentration, ppbV
harvest
postharvest
Elizabeth #18
Darrow #16
Chandler #12
Brigitta Blue #9
Rubel #39
Simultan #41
Hortblue Poppins #35
Liberty #26
Marimba #27
Puru #37
Azur #4
Aron #1
Southern Belle #43
Goldtraube #21
Blue Moon #8
Roxy Blue #38
Berkeley #5
Safir #40
Top Hat #45
Mondo #29
Misty #28
Biloxi #6
Emerald #20
Toro #46
Northland #31
Elliott #19
Atlantic #2
Sky Blue #42
Aurora #3
Nui #32
Earliblue #17
Legacy #25
Coville #15
Ozark Blue #34
Jubilee #24
Jersey #22
Star #44
Cosmopolitan #14
Northblue #30
Compact #13
Jewel #23
Centra Blue #10
Bluecrop #7
Primadonna #36
O'Neal #33
Centurion #11
B
density
concentration, ppbV
index SI
C
H
PH
SI
Concentration, ppbV
Cluster
genetic cluster
1
2
3
4
n.a.

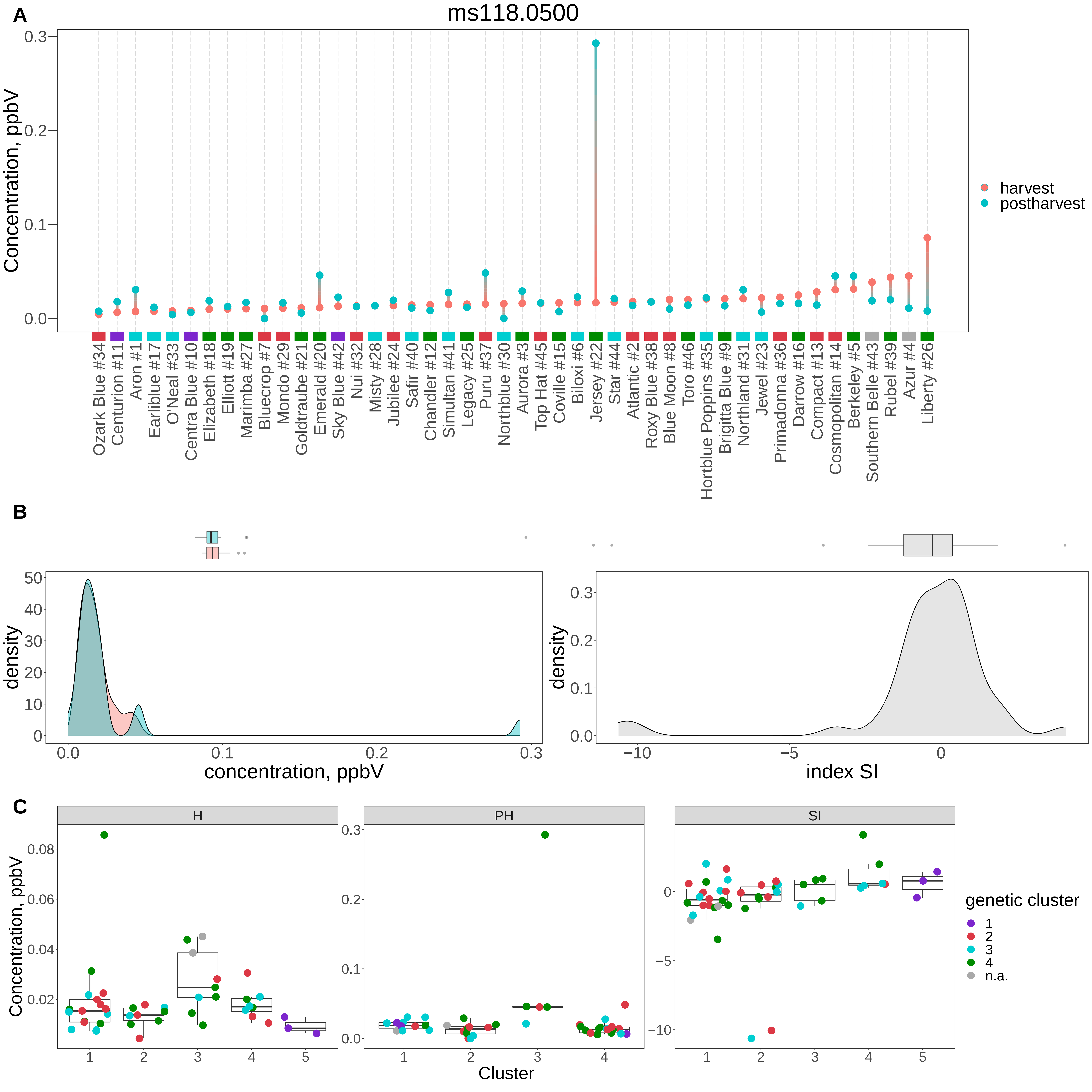

A
ms118.0500
Concentration, ppbV
harvest
postharvest
Ozark Blue #34
Centurion #11
Aron #1
Earliblue #17
O'Neal #33
Centra Blue #10
Elizabeth #18
Elliott #19
Marimba #27
Bluecrop #7
Mondo #29
Goldtraube #21
Emerald #20
Sky Blue #42
Nui #32
Misty #28
Jubilee #24
Safir #40
Chandler #12
Simultan #41
Legacy #25
Puru #37
Northblue #30
Aurora #3
Top Hat #45
Coville #15
Biloxi #6
Jersey #22
Star #44
Atlantic #2
Roxy Blue #38
Blue Moon #8
Toro #46
Hortblue Poppins #35
Brigitta Blue #9
Northland #31
Jewel #23
Primadonna #36
Darrow #16
Compact #13
Cosmopolitan #14
Berkeley #5
Southern Belle #43
Rubel #39
Azur #4
Liberty #26
B
density
concentration, ppbV
index SI
C
H
PH
SI
Concentration, ppbV
Cluster
genetic cluster
1
2
3
4
n.a.

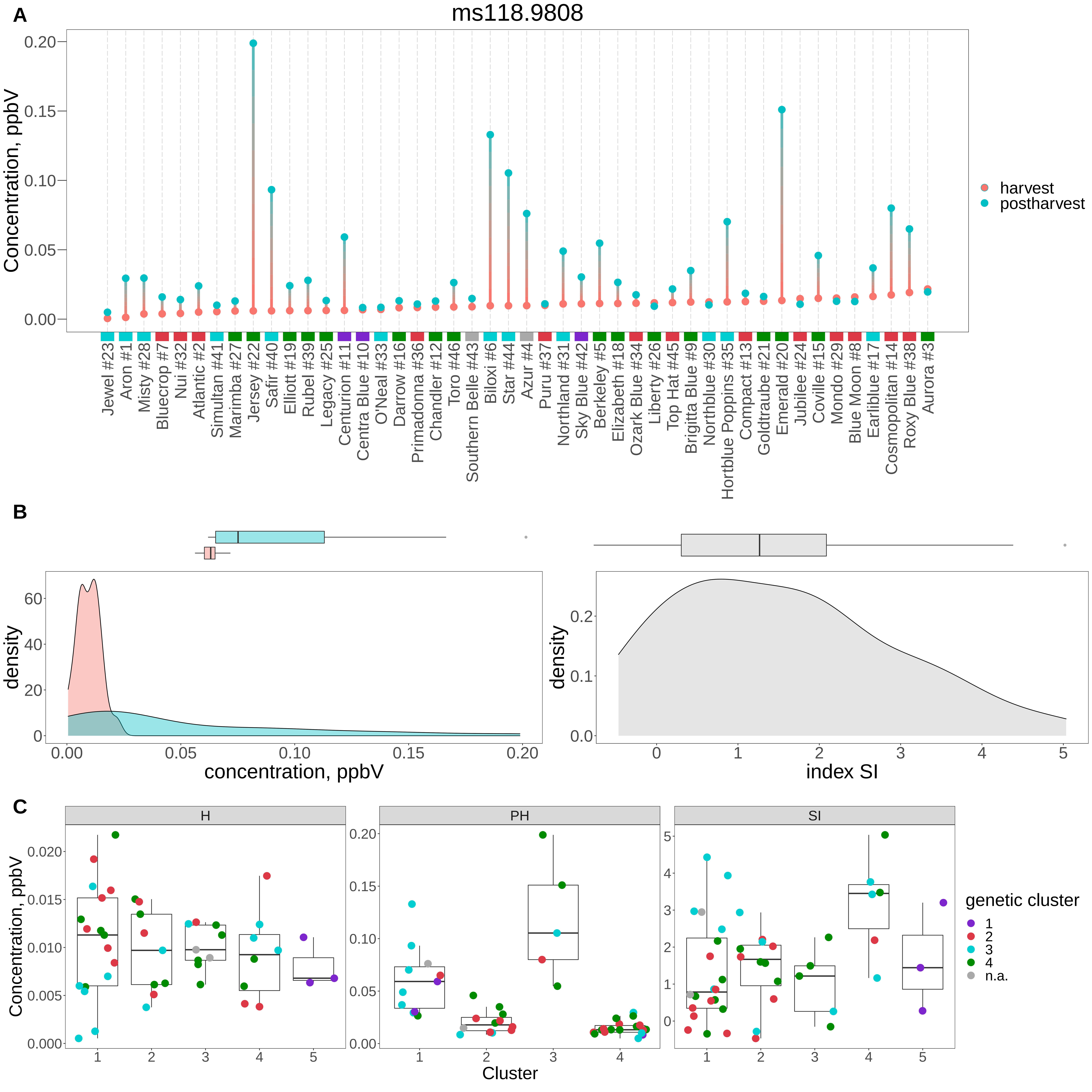

A
ms118.9808
Concentration, ppbV
harvest
postharvest
Jewel #23
Aron #1
Misty #28
Bluecrop #7
Nui #32
Atlantic #2
Simultan #41
Marimba #27
Jersey #22
Safir #40
Elliott #19
Rubel #39
Legacy #25
Centurion #11
Centra Blue #10
O'Neal #33
Darrow #16
Primadonna #36
Chandler #12
Toro #46
Southern Belle #43
Biloxi #6
Star #44
Azur #4
Puru #37
Northland #31
Sky Blue #42
Berkeley #5
Elizabeth #18
Ozark Blue #34
Liberty #26
Top Hat #45
Brigitta Blue #9
Northblue #30
Hortblue Poppins #35
Compact #13
Goldtraube #21
Emerald #20
Jubilee #24
Coville #15
Mondo #29
Blue Moon #8
Earliblue #17
Cosmopolitan #14
Roxy Blue #38
Aurora #3
B
density
concentration, ppbV
index SI
C
H
PH
SI
Concentration, ppbV
Cluster
genetic cluster
1
2
3
4
n.a.

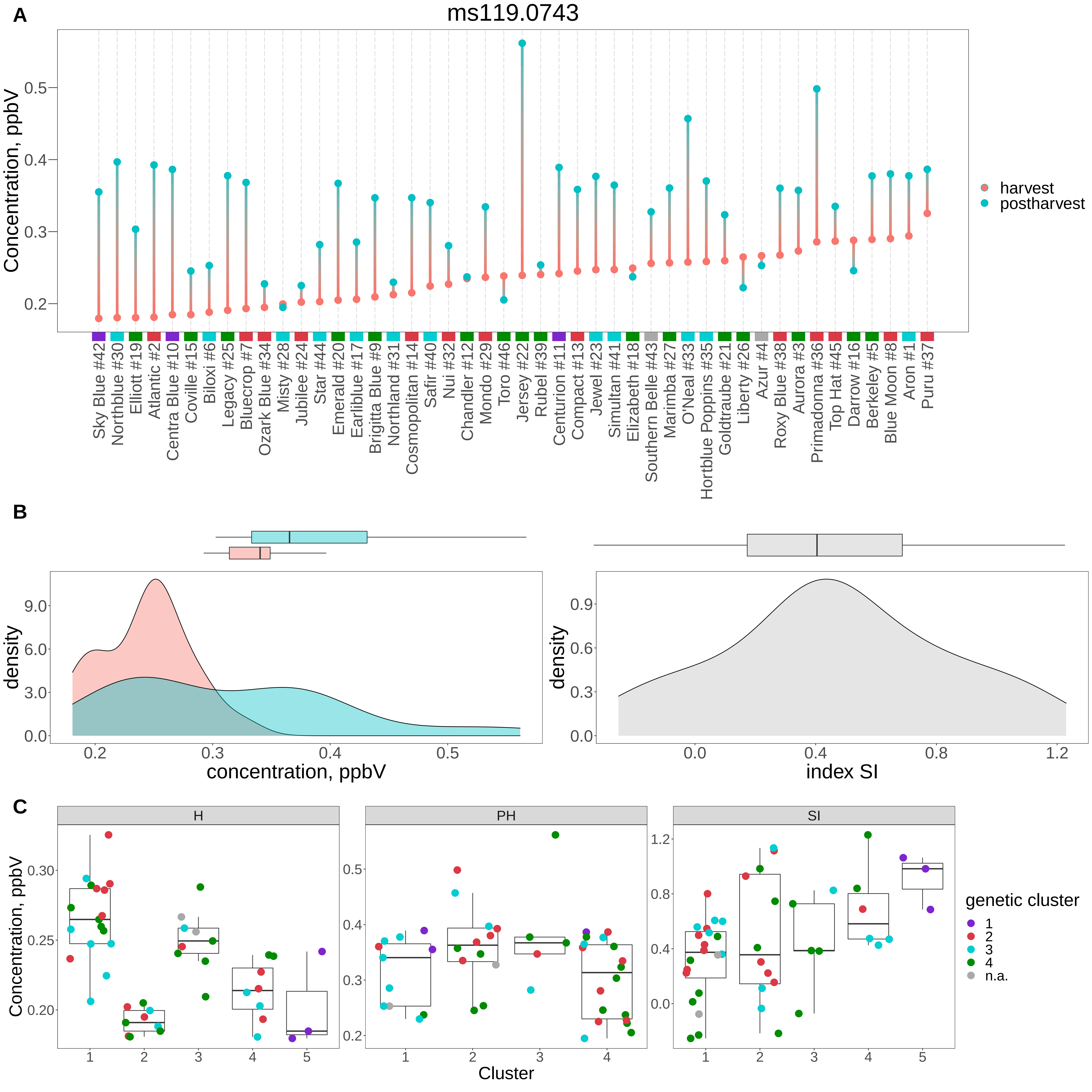
A
ms119.0743
Concentration, ppbV
harvest
postharvest
Sky Blue #42
Northblue #30
Elliott #19
Atlantic #2
Centra Blue #10
Coville #15
Biloxi #6
Legacy #25
Bluecrop #7
Ozark Blue #34
Misty #28
Jubilee #24
Star #44
Emerald #20
Earliblue #17
Brigitta Blue #9
Northland #31
Cosmopolitan #14
Safir #40
Nui #32
Chandler #12
Mondo #29
Toro #46
Jersey #22
Rubel #39
Centurion #11
Compact #13
Jewel #23
Simultan #41
Elizabeth #18
Southern Belle #43
Marimba #27
O'Neal #33
Hortblue Poppins #35
Goldtraube #21
Liberty #26
Azur #4
Roxy Blue #38
Aurora #3
Primadonna #36
Top Hat #45
Darrow #16
Berkeley #5
Blue Moon #8
Aron #1
Puru #37
B
density
concentration, ppbV
index SI
C
H
PH
SI
Concentration, ppbV
Cluster
genetic cluster
1
2
3
4
n.a.

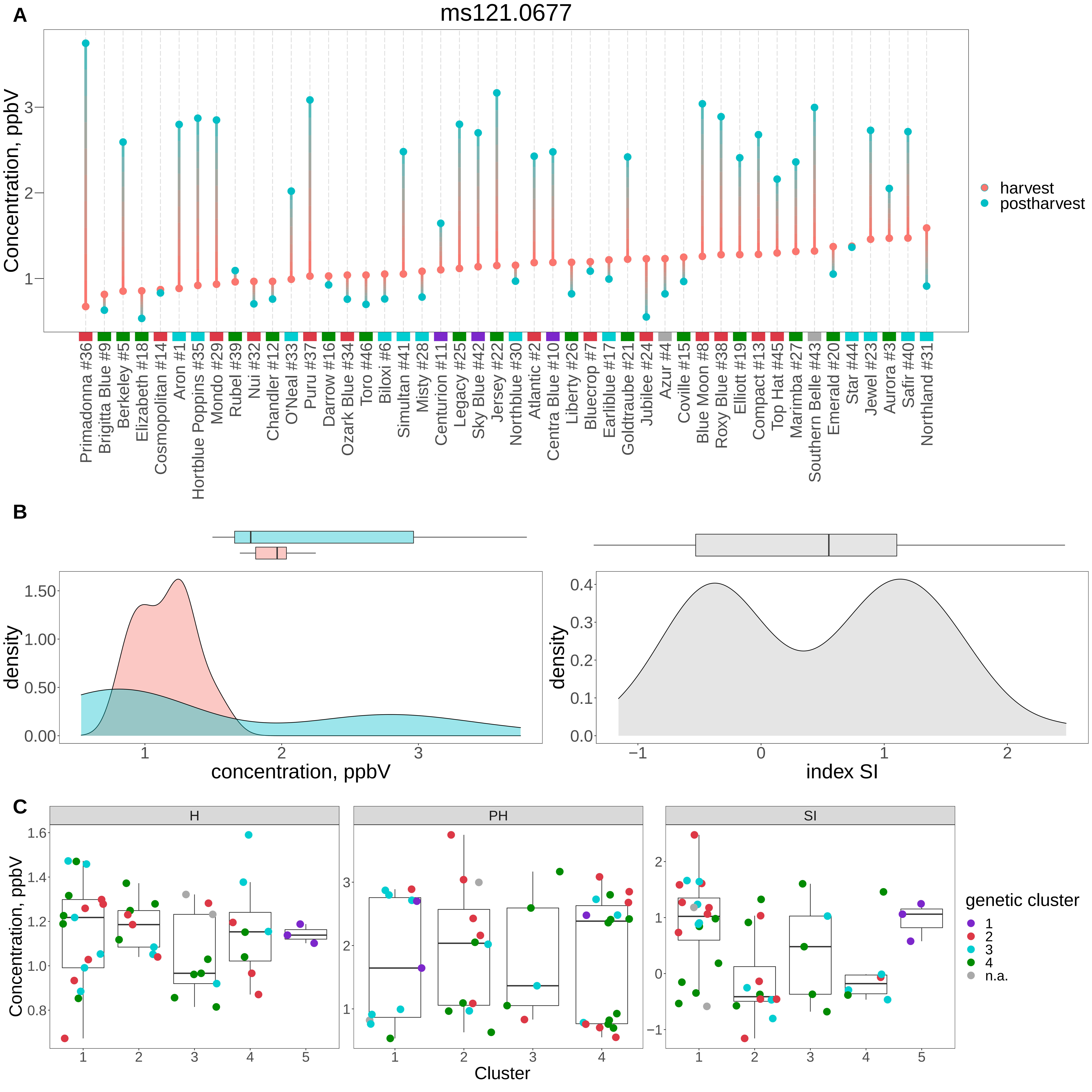
A
ms121.0677
Concentration, ppbV
harvest
postharvest
Primadonna #36
Brigitta Blue #9
Berkeley #5
Elizabeth #18
Cosmopolitan #14
Aron #1
Hortblue Poppins #35
Mondo #29
Rubel #39
Nui #32
Chandler #12
O'Neal #33
Puru #37
Darrow #16
Ozark Blue #34
Toro #46
Biloxi #6
Simultan #41
Misty #28
Centurion #11
Legacy #25
Sky Blue #42
Jersey #22
Northblue #30
Atlantic #2
Centra Blue #10
Liberty #26
Bluecrop #7
Earliblue #17
Goldtraube #21
Jubilee #24
Azur #4
Coville #15
Blue Moon #8
Roxy Blue #38
Elliott #19
Compact #13
Top Hat #45
Marimba #27
Southern Belle #43
Emerald #20
Star #44
Jewel #23
Aurora #3
Safir #40
Northland #31
B
density
concentration, ppbV
density
index SI
C
H
PH
SI
Concentration, ppbV
Cluster
genetic cluster
1
2
3
4
n.a.

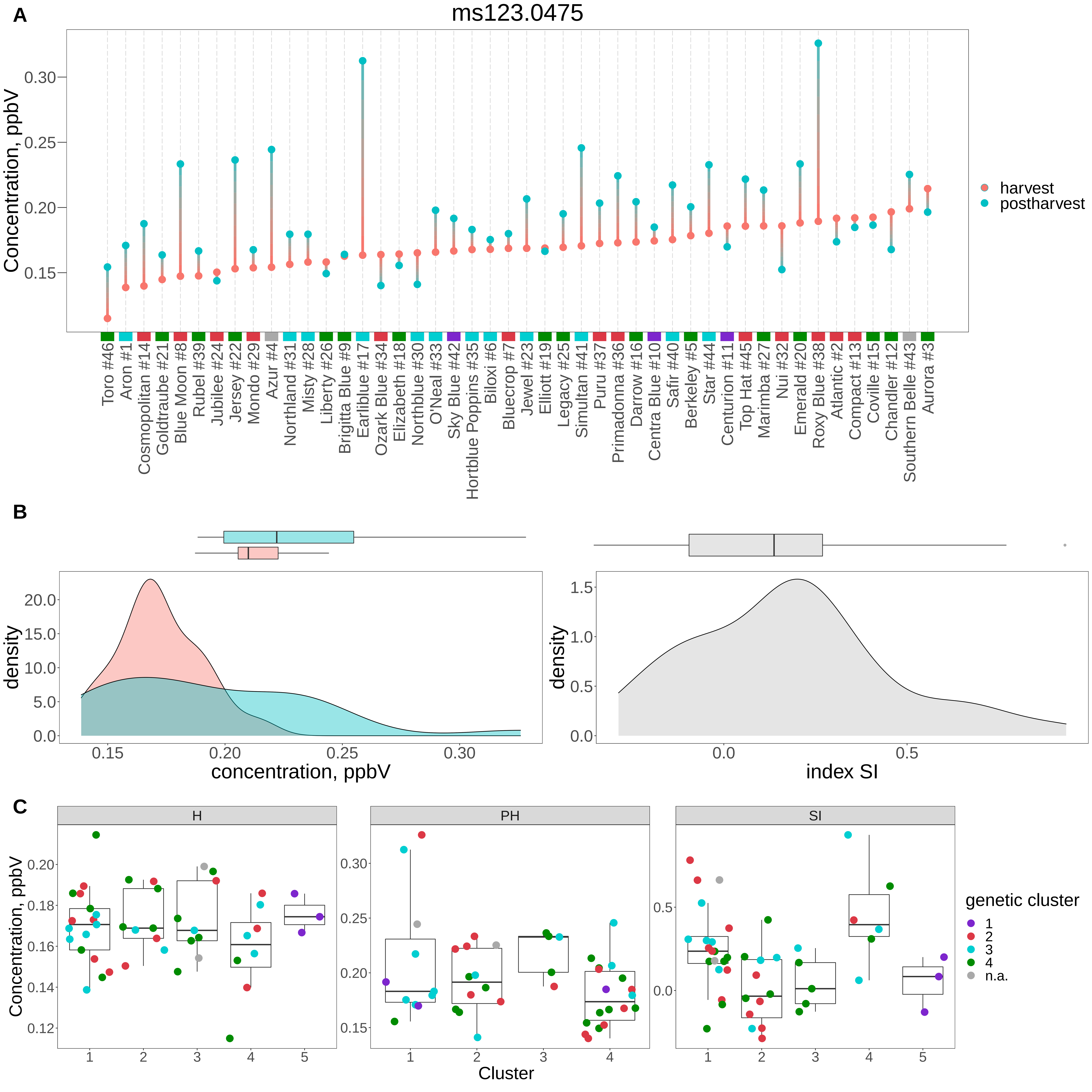
A
ms123.0475
Concentration, ppbV
harvest
postharvest
Toro #46
Aron #1
Cosmopolitan #14
Goldtraube #21
Blue Moon #8
Rubel #39
Jubilee #24
Jersey #22
Mondo #29
Azur #4
Northland #31
Misty #28
Liberty #26
Brigitta Blue #9
Earliblue #17
Ozark Blue #34
Elizabeth #18
Northblue #30
O'Neal #33
Sky Blue #42
Hortblue Poppins #35
Biloxi #6
Bluecrop #7
Jewel #23
Elliott #19
Legacy #25
Simultan #41
Puru #37
Primadonna #36
Darrow #16
Centra Blue #10
Safir #40
Berkeley #5
Star #44
Centurion #11
Top Hat #45
Marimba #27
Nui #32
Emerald #20
Roxy Blue #38
Atlantic #2
Compact #13
Coville #15
Chandler #12
Southern Belle #43
Aurora #3
B
density
concentration, ppbV
index SI
C
H
PH
SI
Concentration, ppbV
Cluster
genetic cluster
1
2
3
4
n.a.

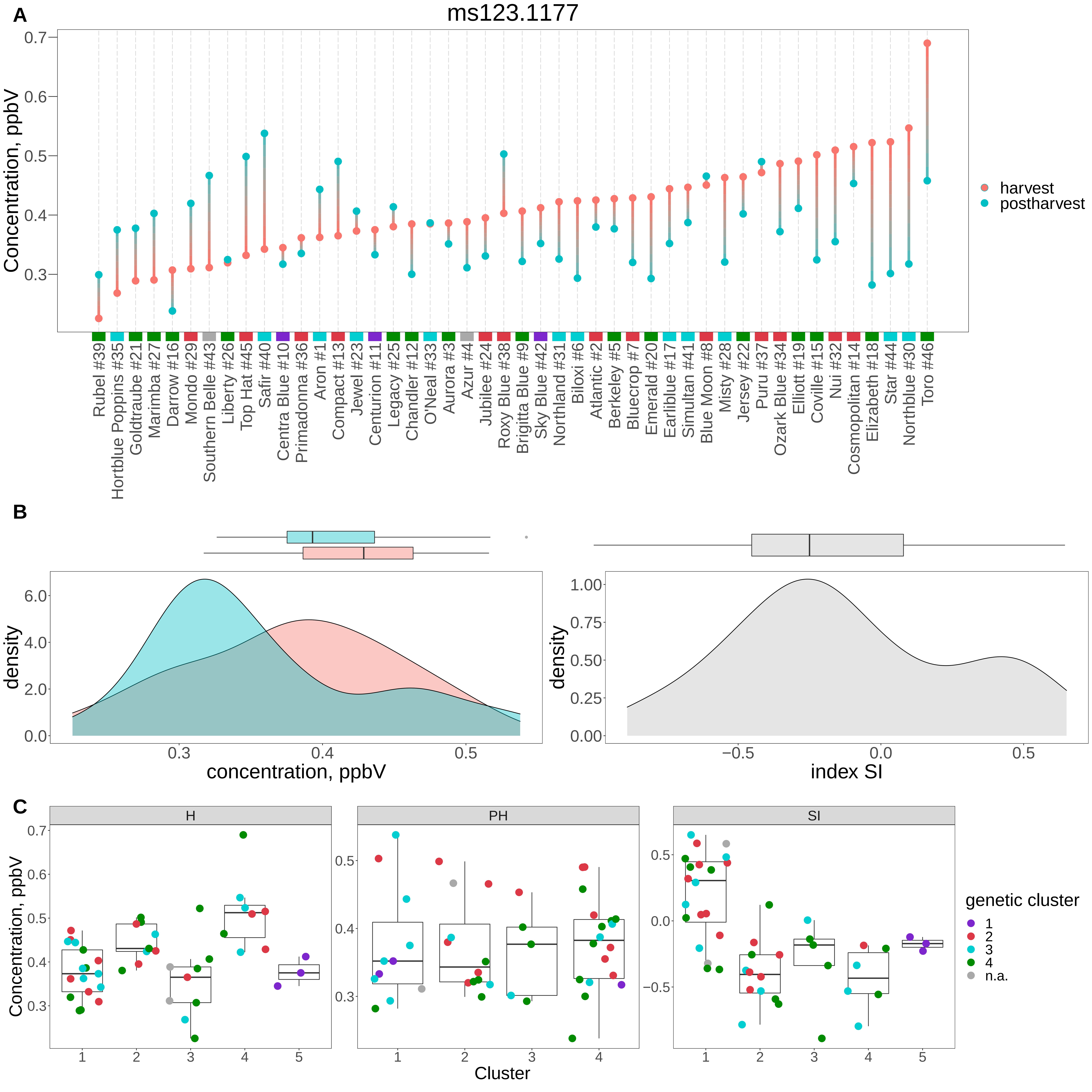
A
ms123.1177
Concentration, ppbV
harvest
postharvest
Rubel #39
Hortblue Poppins #35
Goldtraube #21
Marimba #27
Darrow #16
Mondo #29
Southern Belle #43
Liberty #26
Top Hat #45
Safir #40
Centra Blue #10
Primadonna #36
Aron #1
Compact #13
Jewel #23
Centurion #11
Legacy #25
Chandler #12
O'Neal #33
Aurora #3
Azur #4
Jubilee #24
Roxy Blue #38
Brigitta Blue #9
Sky Blue #42
Northland #31
Biloxi #6
Atlantic #2
Berkeley #5
Bluecrop #7
Emerald #20
Earliblue #17
Simultan #41
Blue Moon #8
Misty #28
Jersey #22
Puru #37
Ozark Blue #34
Elliott #19
Coville #15
Nui #32
Cosmopolitan #14
Elizabeth #18
Star #44
Northblue #30
Toro #46
B
density
concentration, ppbV
index SI
C
H
PH
SI
Concentration, ppbV
Cluster
genetic cluster
1
2
3
4
n.a.

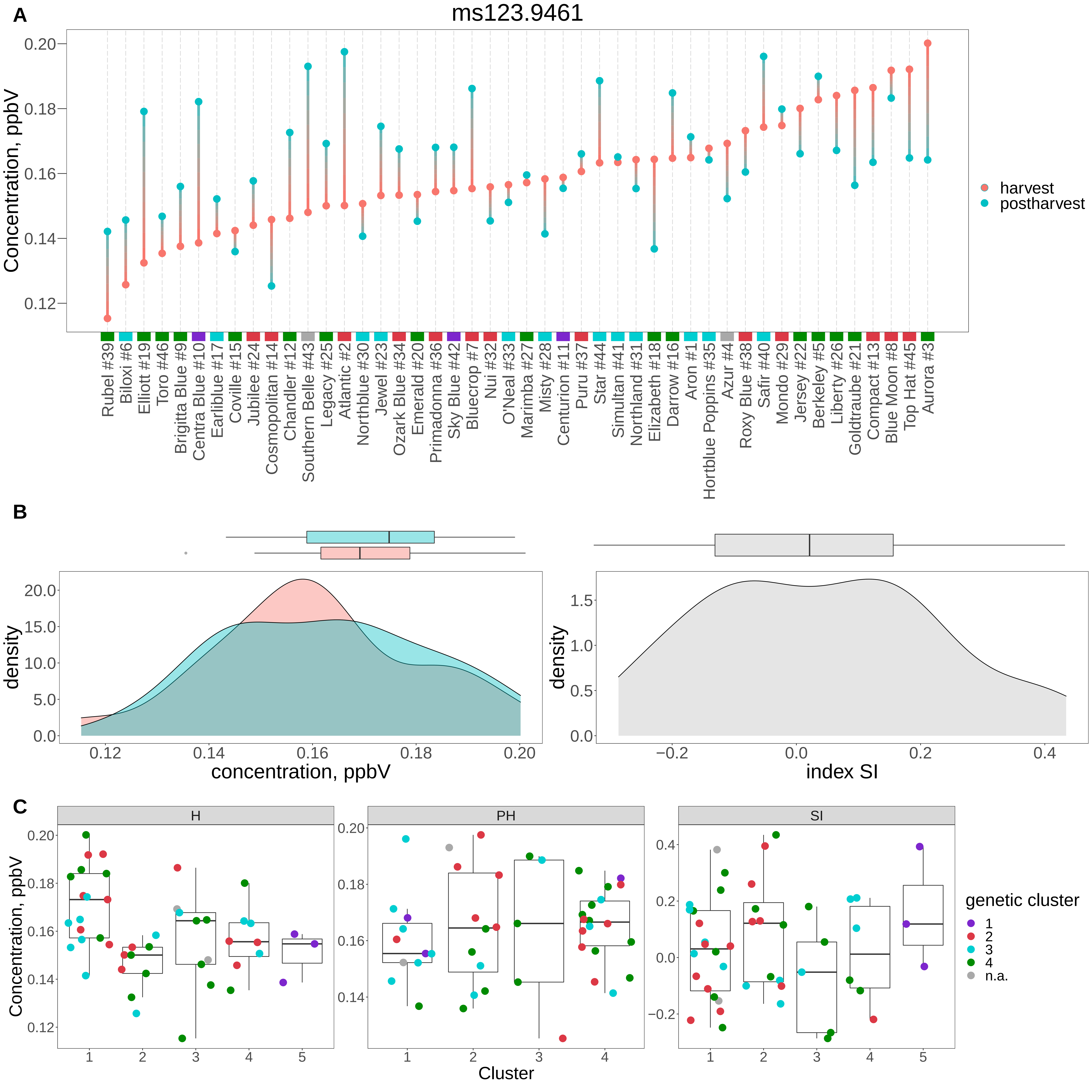
A
ms123.9461
Concentration, ppbV
0.20
0.18
0.16
0.14
0.12
harvest
postharvest
Rubel #39
Biloxi #6
Elliott #19
Toro #46
Brigitta Blue #9
Centra Blue #10
Earliblue #17
Coville #15
Jubilee #24
Cosmopolitan #14
Chandler #12
Southern Belle #43
Legacy #25
Atlantic #2
Northblue #30
Jewel #23
Ozark Blue #34
Emerald #20
Primadonna #36
Sky Blue #42
Bluecrop #7
Nui #32
O'Neal #33
Marimba #27
Misty #28
Centurion #11
Puru #37
Star #44
Simultan #41
Northland #31
Elizabeth #18
Darrow #16
Aron #1
Hortblue Poppins #35
Azur #4
Roxy Blue #38
Safir #40
Mondo #29
Jersey #22
Berkeley #5
Liberty #26
Goldtraube #21
Compact #13
Blue Moon #8
Top Hat #45
Aurora #3
B
density
20.0
15.0
10.0
5.0
0.0
0.12
0.14
0.16
0.18
0.20
concentration, ppbV
1.5
1.0
0.5
0.0
−0.2
0.0
0.2
0.4
index SI
C
H
PH
SI
Concentration, ppbV
Cluster
genetic cluster
1
2
3
4
5
n.a.

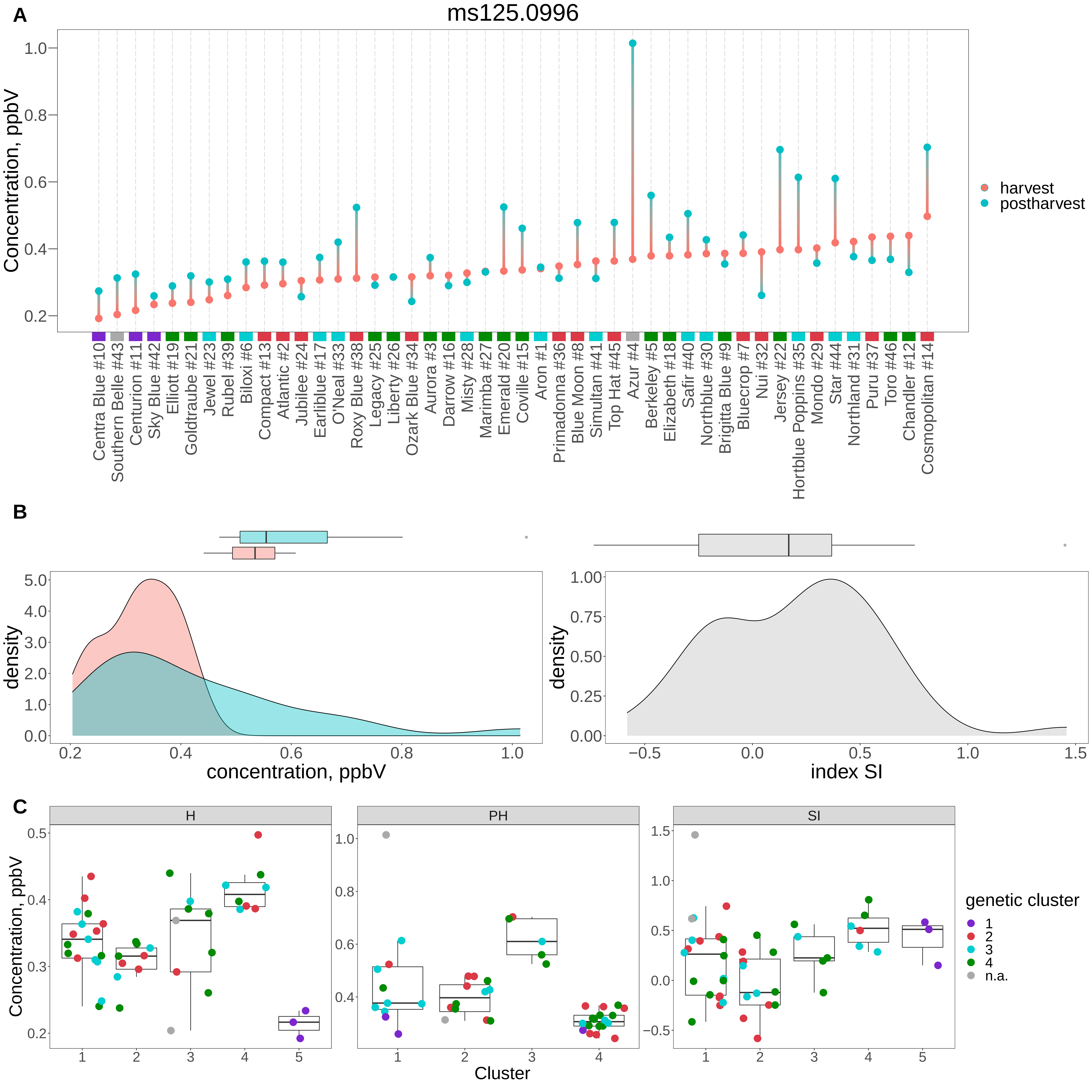
A
ms125.0996
Concentration, ppbV
harvest
postharvest
Centra Blue #10
Southern Belle #43
Centurion #11
Sky Blue #42
Elliott #19
Goldtraube #21
Jewel #23
Rubel #39
Biloxi #6
Compact #13
Atlantic #2
Jubilee #24
Earliblue #17
O'Neal #33
Roxy Blue #38
Legacy #25
Liberty #26
Ozark Blue #34
Aurora #3
Darrow #16
Misty #28
Marimba #27
Emerald #20
Coville #15
Aron #1
Primadonna #36
Blue Moon #8
Simultan #41
Top Hat #45
Azur #4
Berkeley #5
Elizabeth #18
Safir #40
Northblue #30
Brigitta Blue #9
Bluecrop #7
Nui #32
Jersey #22
Hortblue Poppins #35
Mondo #29
Star #44
Northland #31
Puru #37
Toro #46
Chandler #12
Cosmopolitan #14
B
density
concentration, ppbV
index SI
C
H
PH
SI
Concentration, ppbV
Cluster
genetic cluster
1
2
3
4
n.a.

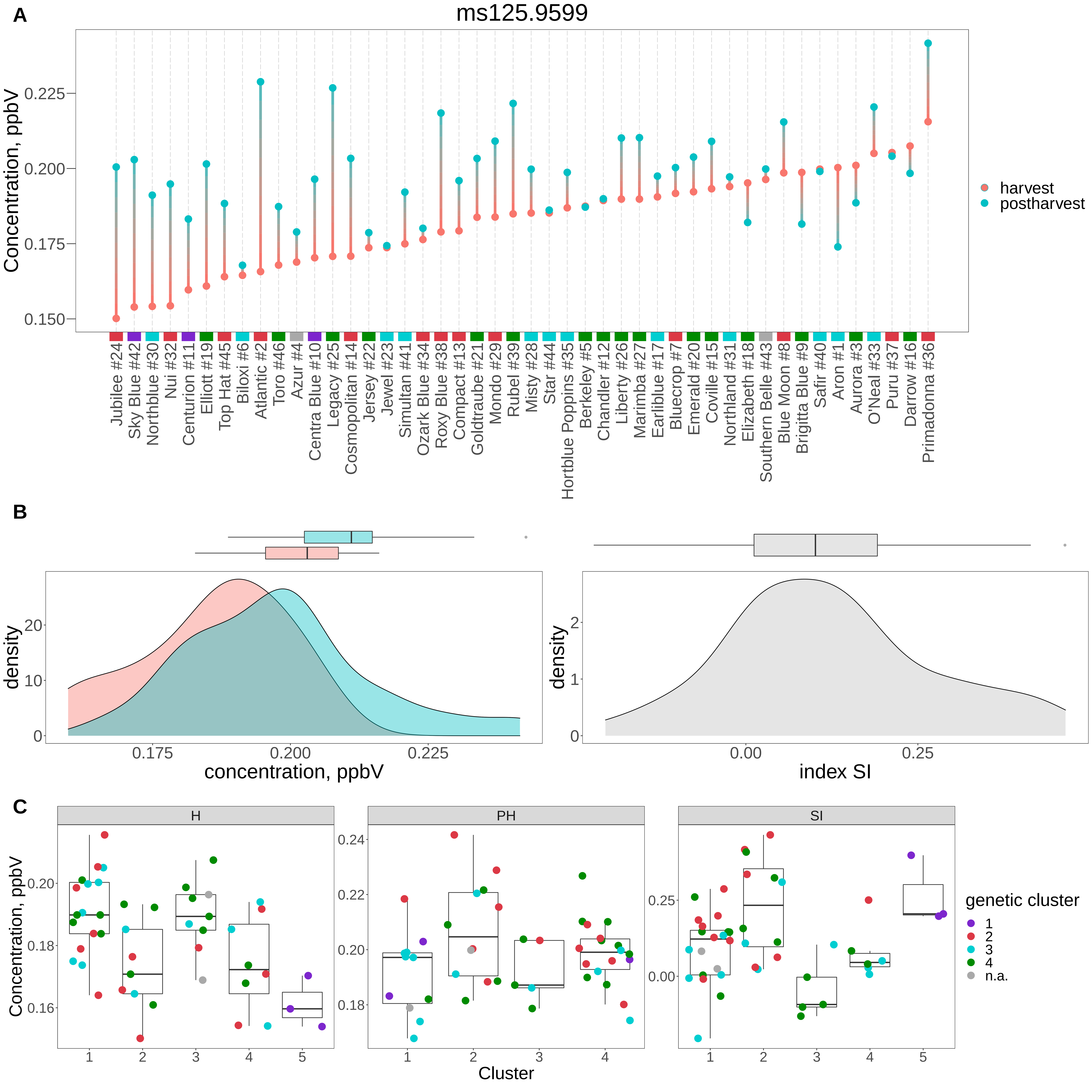
A
ms125.9599
Concentration, ppbV
0.150
0.175
0.200
0.225
harvest
postharvest
Jubilee #24
Sky Blue #42
Northblue #30
Nui #32
Centurion #11
Elliott #19
Top Hat #45
Biloxi #6
Atlantic #2
Toro #46
Azur #4
Centra Blue #10
Legacy #25
Cosmopolitan #14
Jersey #22
Jewel #23
Simultan #41
Ozark Blue #34
Roxy Blue #38
Compact #13
Goldtraube #21
Mondo #29
Rubel #39
Misty #28
Star #44
Hortblue Poppins #35
Berkeley #5
Chandler #12
Liberty #26
Marimba #27
Earliblue #17
Bluecrop #7
Emerald #20
Coville #15
Northland #31
Elizabeth #18
Southern Belle #43
Blue Moon #8
Brigitta Blue #9
Safir #40
Aron #1
Aurora #3
O'Neal #33
Puru #37
Darrow #16
Primadonna #36
B
density
0
10
20
0.175
0.200
0.225
concentration, ppbV
density
0
1
2
0.00
0.25
index SI
C
H
PH
SI
Concentration, ppbV
0.16
0.18
0.20
0.22
0.24
0.00
0.25
1
2
3
4
5
Cluster
genetic cluster
1
2
3
4
n.a.

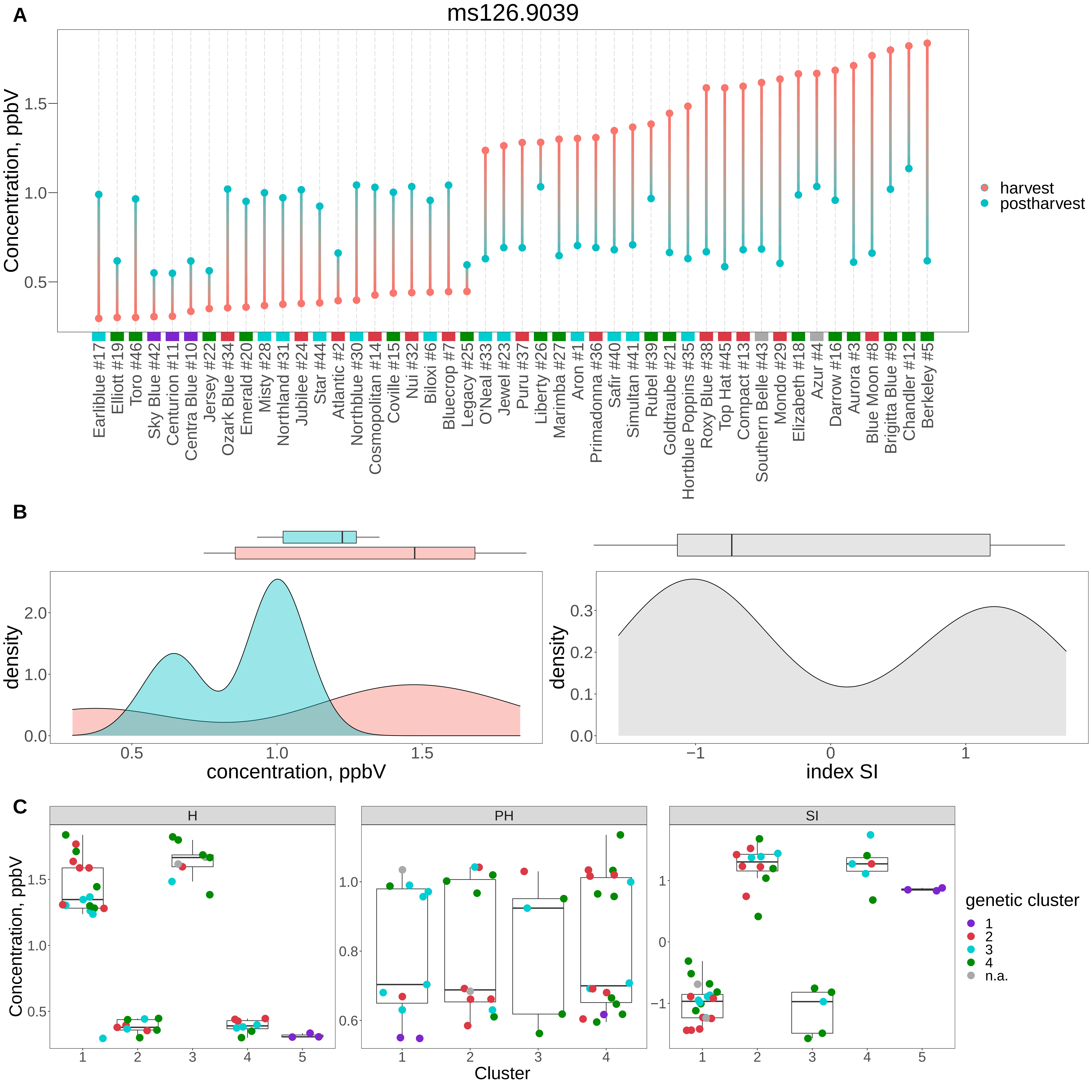
A
ms126.9039
Concentration, ppbV
harvest
postharvest
Earliblue #17
Elliott #19
Toro #46
Sky Blue #42
Centurion #11
Centra Blue #10
Jersey #22
Ozark Blue #34
Emerald #20
Misty #28
Northland #31
Jubilee #24
Star #44
Atlantic #2
Northblue #30
Cosmopolitan #14
Coville #15
Nui #32
Biloxi #6
Bluecrop #7
Legacy #25
O'Neal #33
Jewel #23
Puru #37
Liberty #26
Marimba #27
Aron #1
Primadonna #36
Safir #40
Simultan #41
Rubel #39
Goldtraube #21
Hortblue Poppins #35
Roxy Blue #38
Top Hat #45
Compact #13
Southern Belle #43
Mondo #29
Elizabeth #18
Azur #4
Darrow #16
Aurora #3
Blue Moon #8
Brigitta Blue #9
Chandler #12
Berkeley #5
B
density
concentration, ppbV
index SI
C
H
PH
SI
Concentration, ppbV
Cluster
genetic cluster
1
2
3
4
n.a.

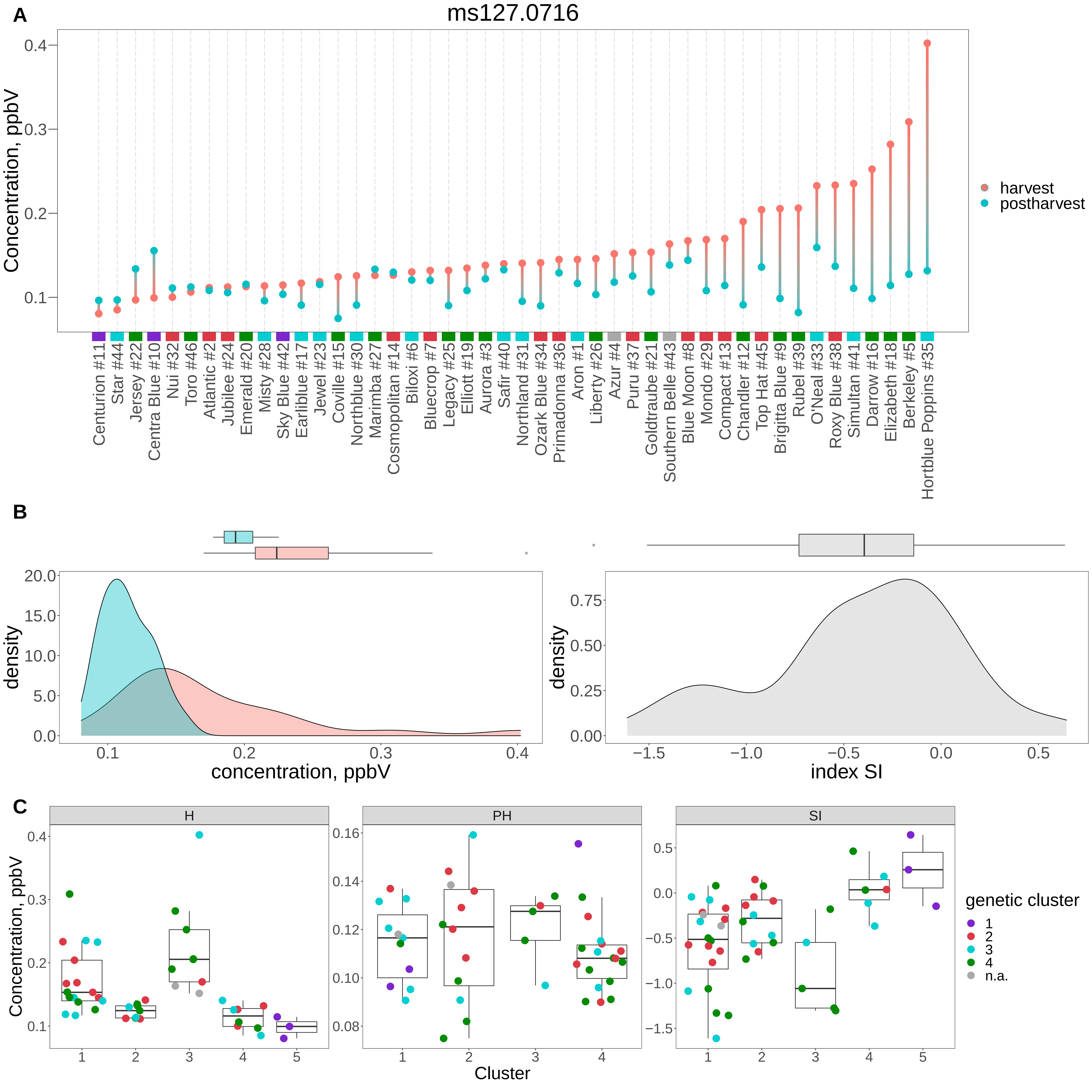

A
ms127.0716
Concentration, ppbV
harvest
postharvest
Centurion #11
Star #44
Jersey #22
Centra Blue #10
Nui #32
Toro #46
Atlantic #2
Jubilee #24
Emerald #20
Misty #28
Sky Blue #42
Earliblue #17
Jewel #23
Coville #15
Northblue #30
Marimba #27
Cosmopolitan #14
Biloxi #6
Bluecrop #7
Legacy #25
Elliott #19
Aurora #3
Safir #40
Northland #31
Ozark Blue #34
Primadonna #36
Aron #1
Liberty #26
Azur #4
Puru #37
Goldtraube #21
Southern Belle #43
Blue Moon #8
Mondo #29
Compact #13
Chandler #12
Top Hat #45
Brigitta Blue #9
Rubel #39
O'Neal #33
Roxy Blue #38
Simultan #41
Darrow #16
Elizabeth #18
Berkeley #5
Hortblue Poppins #35
B
density
concentration, ppbV
index SI
C
H
PH
SI
Cluster
genetic cluster
1
2
3
4
n.a.

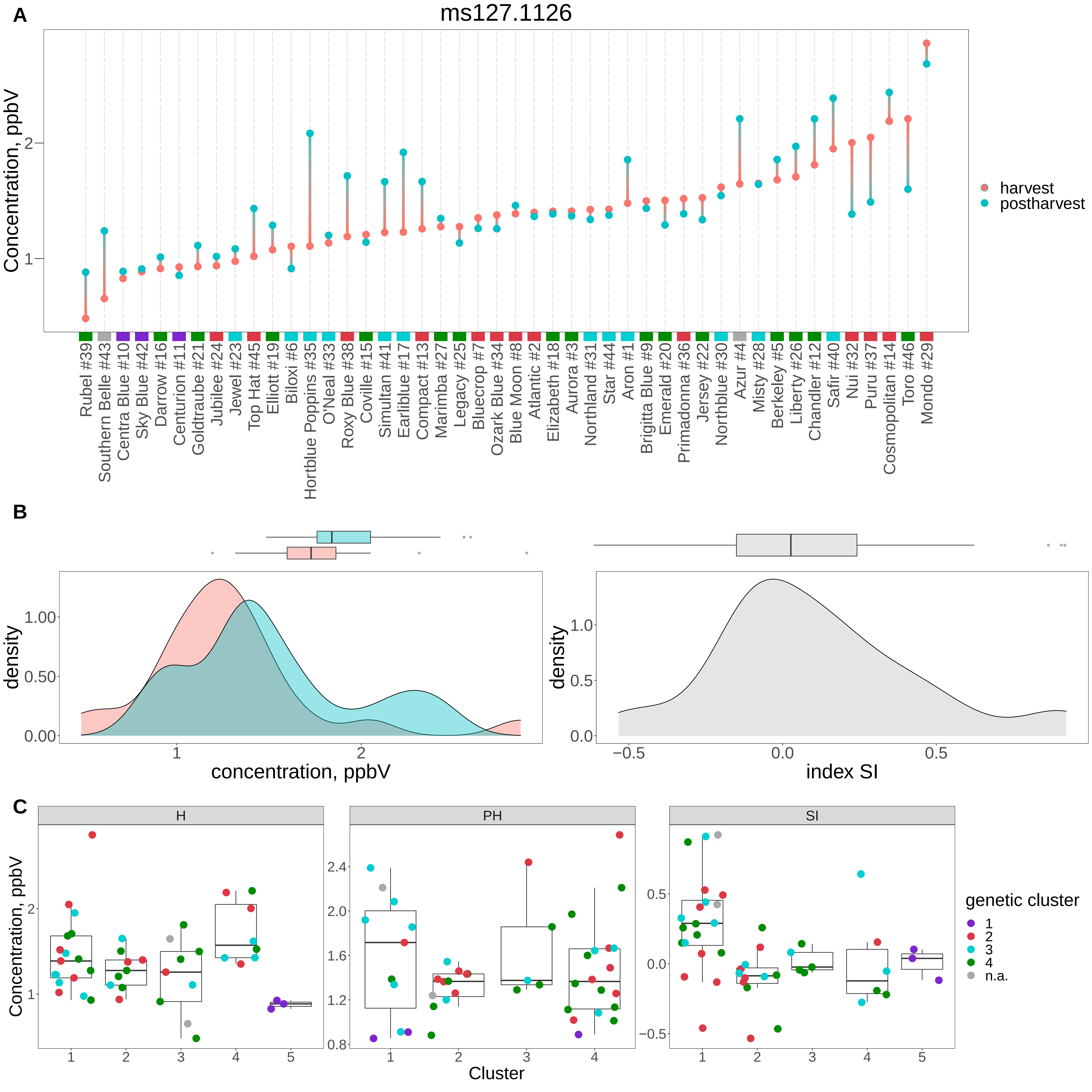
A
ms127.1126
Concentration, ppbV
harvest
postharvest
Rubel #39
Southern Belle #43
Centra Blue #10
Sky Blue #42
Darrow #16
Centurion #11
Goldtraube #21
Jubilee #24
Jewel #23
Top Hat #45
Elliott #19
Biloxi #6
Hortblue Poppins #35
O'Neal #33
Roxy Blue #38
Coville #15
Simultan #41
Earliblue #17
Compact #13
Marimba #27
Legacy #25
Bluecrop #7
Ozark Blue #34
Blue Moon #8
Atlantic #2
Elizabeth #18
Aurora #3
Northland #31
Star #44
Aron #1
Brigitta Blue #9
Emerald #20
Primadonna #36
Jersey #22
Northblue #30
Azur #4
Misty #28
Berkeley #5
Liberty #26
Chandler #12
Safir #40
Nui #32
Puru #37
Cosmopolitan #14
Toro #46
Mondo #29
B
density
concentration, ppbV
index SI
C
H
PH
SI
Concentration, ppbV
Cluster
genetic cluster
1
2
3
4
n.a.

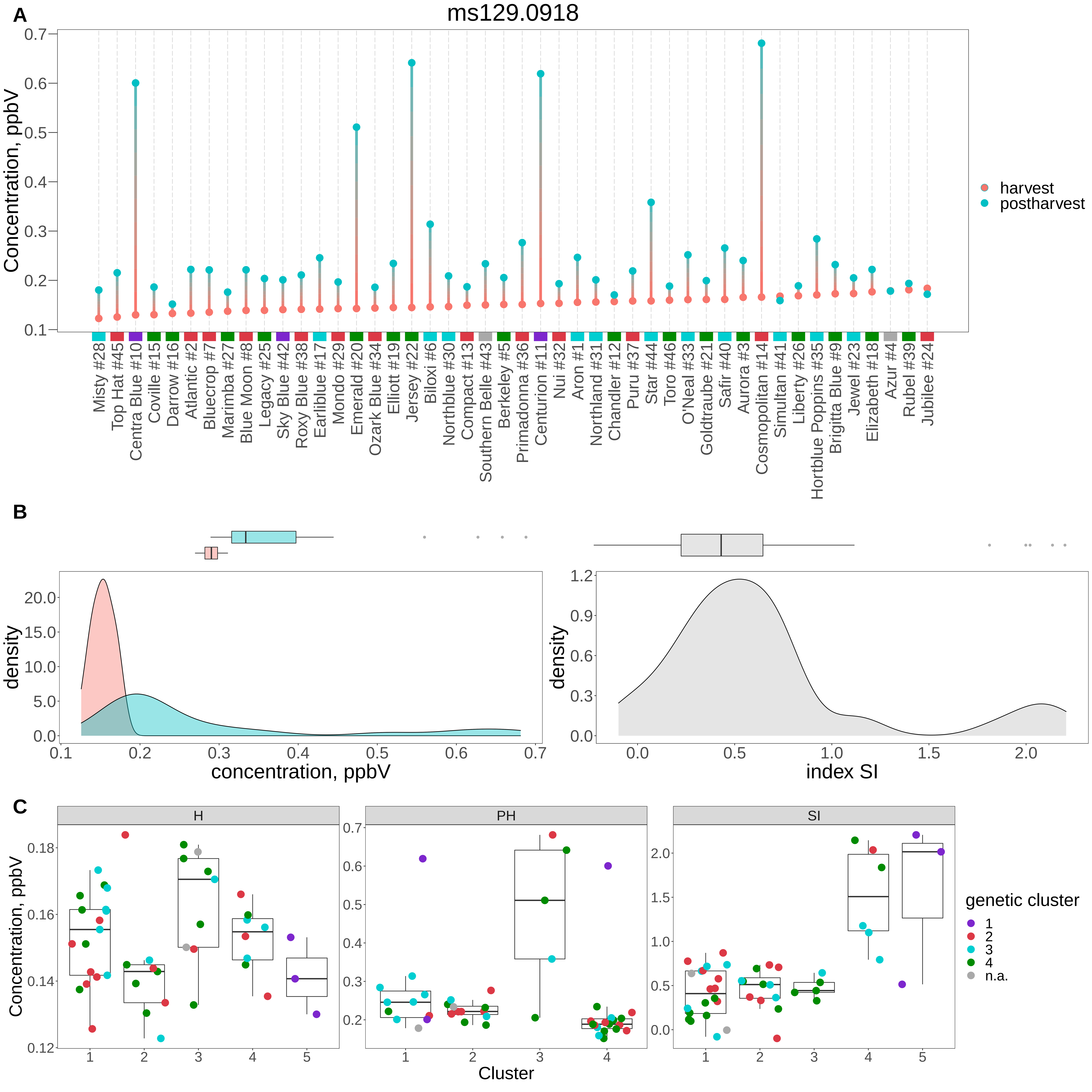
A
ms129.0918
Concentration, ppbV
harvest
postharvest
Misty #28
Top Hat #45
Centra Blue #10
Coville #15
Darrow #16
Atlantic #2
Bluecrop #7
Marimba #27
Blue Moon #8
Legacy #25
Sky Blue #42
Roxy Blue #38
Earliblue #17
Mondo #29
Emerald #20
Ozark Blue #34
Elliott #19
Jersey #22
Biloxi #6
Northblue #30
Compact #13
Southern Belle #43
Berkeley #5
Primadonna #36
Centurion #11
Nui #32
Aron #1
Northland #31
Chandler #12
Puru #37
Star #44
Toro #46
O'Neal #33
Goldtraube #21
Safir #40
Aurora #3
Cosmopolitan #14
Simultan #41
Liberty #26
Hortblue Poppins #35
Brigitta Blue #9
Jewel #23
Elizabeth #18
Azur #4
Rubel #39
Jubilee #24
B
density
concentration, ppbV
index SI
C
H
PH
SI
Concentration, ppbV
Cluster
genetic cluster
1
2
3
4
n.a.

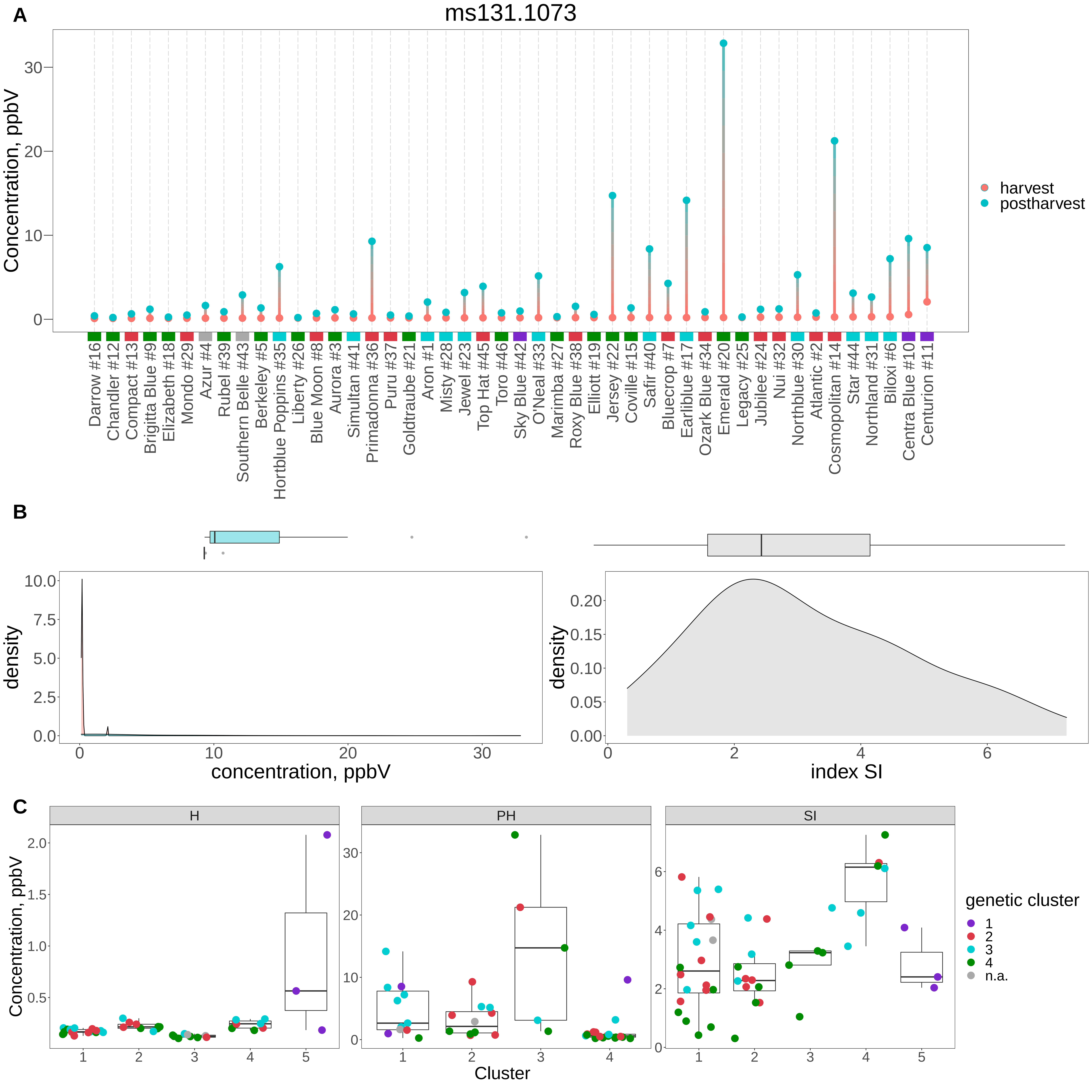

A
ms131.1073
Concentration, ppbV
harvest
postharvest
Darrow #16
Chandler #12
Compact #13
Brigitta Blue #9
Elizabeth #18
Mondo #29
Azur #4
Rubel #39
Southern Belle #43
Berkeley #5
Hortblue Poppins #35
Liberty #26
Blue Moon #8
Aurora #3
Simultan #41
Primadonna #36
Puru #37
Goldtraube #21
Aron #1
Misty #28
Jewel #23
Top Hat #45
Toro #46
Sky Blue #42
O'Neal #33
Marimba #27
Roxy Blue #38
Elliott #19
Jersey #22
Coville #15
Safir #40
Bluecrop #7
Earliblue #17
Ozark Blue #34
Emerald #20
Legacy #25
Jubilee #24
Nui #32
Northblue #30
Atlantic #2
Cosmopolitan #14
Star #44
Northland #31
Biloxi #6
Centra Blue #10
Centurion #11
B
density
concentration, ppbV
index SI
C
H
PH
SI
Cluster
genetic cluster
1
2
3
4
n.a.

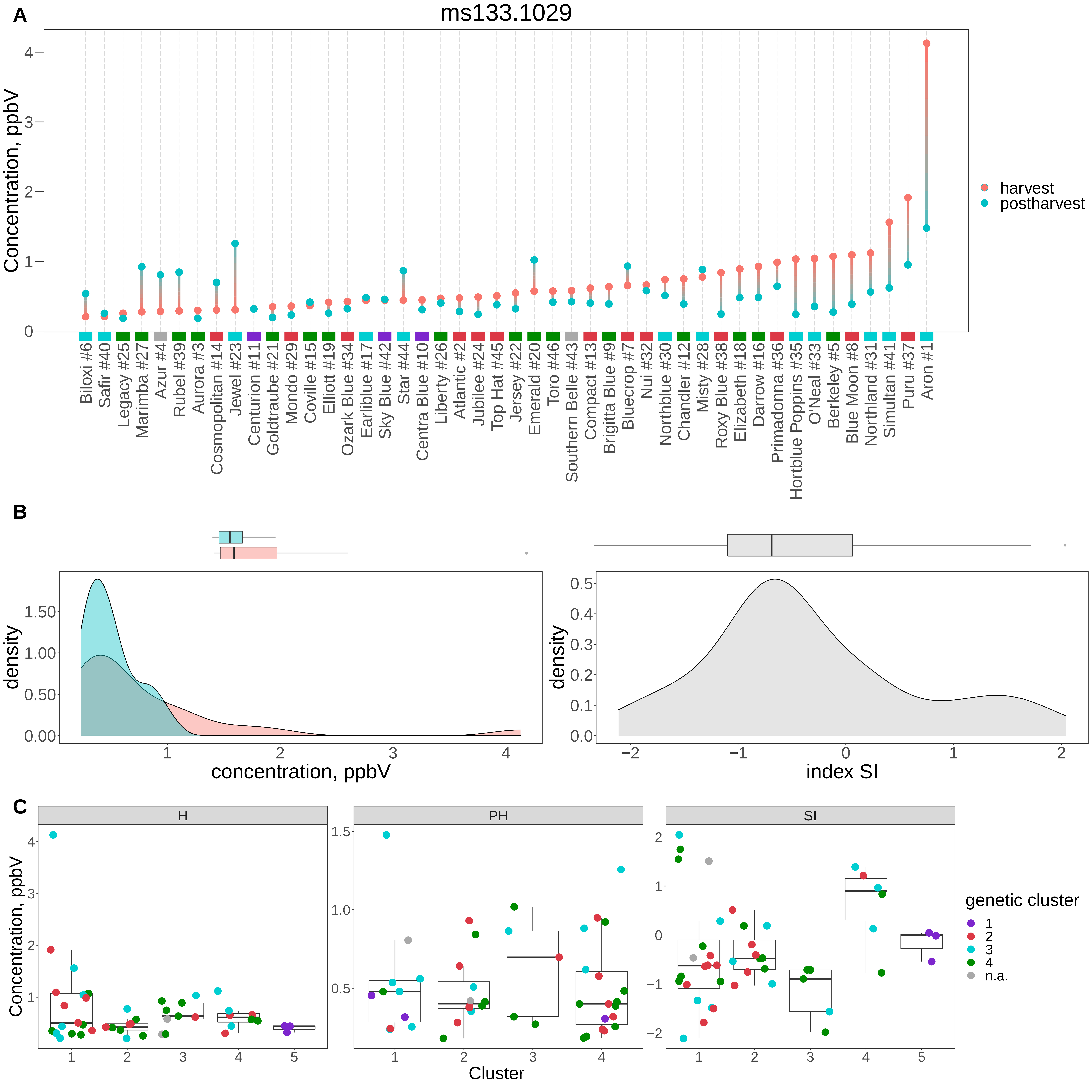

A
ms133.1029
Concentration, ppbV
harvest
postharvest
Biloxi #6
Safir #40
Legacy #25
Marimba #27
Azur #4
Rubel #39
Aurora #3
Cosmopolitan #14
Jewel #23
Centurion #11
Goldtraube #21
Mondo #29
Coville #15
Elliott #19
Ozark Blue #34
Earliblue #17
Sky Blue #42
Star #44
Centra Blue #10
Liberty #26
Atlantic #2
Jubilee #24
Top Hat #45
Jersey #22
Emerald #20
Toro #46
Southern Belle #43
Compact #13
Brigitta Blue #9
Bluecrop #7
Nui #32
Northblue #30
Chandler #12
Misty #28
Roxy Blue #38
Elizabeth #18
Darrow #16
Primadonna #36
Hortblue Poppins #35
O'Neal #33
Berkeley #5
Blue Moon #8
Northland #31
Simultan #41
Puru #37
Aron #1
B
density
concentration, ppbV
density
index SI
C
H
PH
SI
Concentration, ppbV
Cluster
genetic cluster
1
2
3
4
n.a.

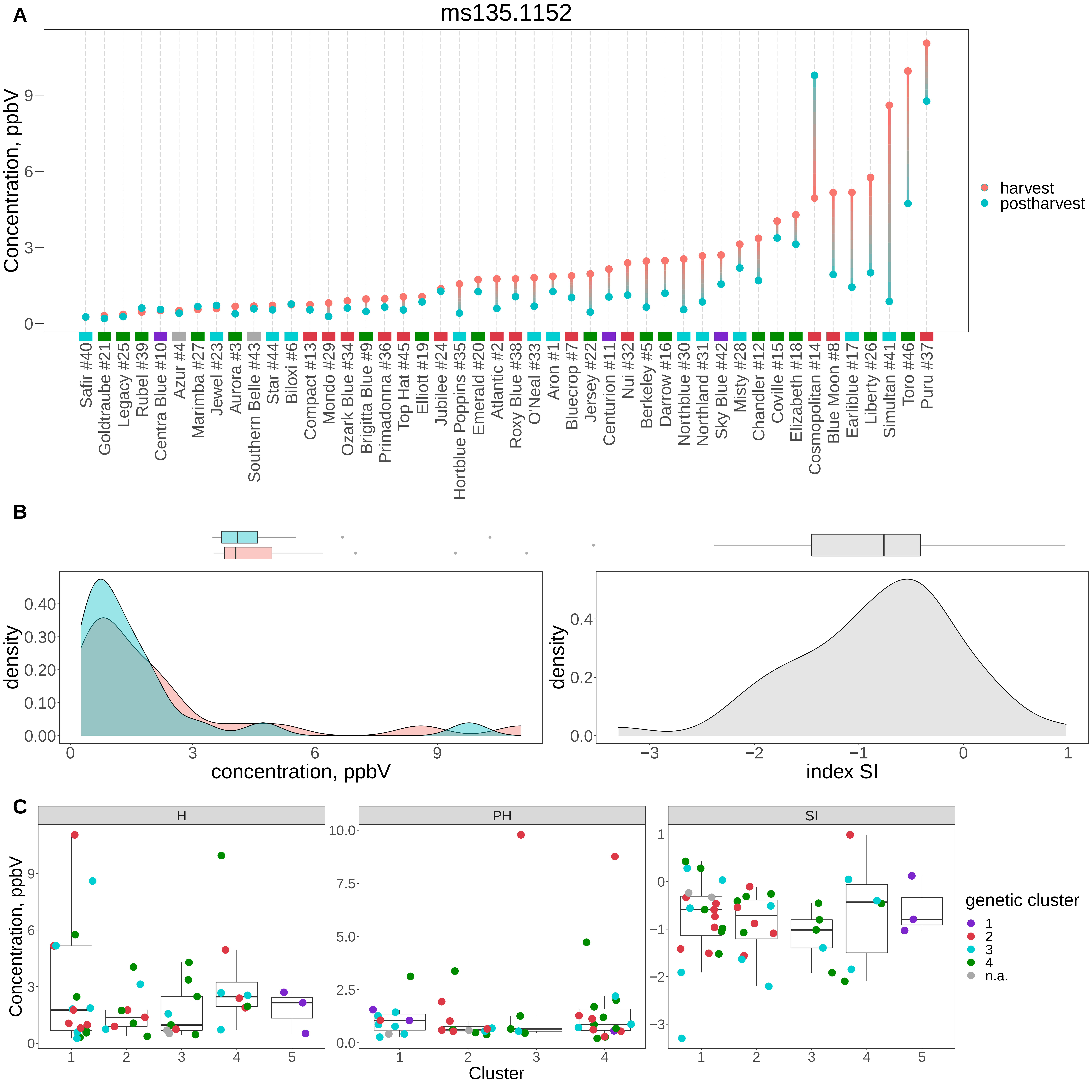
A
ms135.1152
Concentration, ppbV
harvest
postharvest
Safir #40
Goldtraube #21
Legacy #25
Rubel #39
Centra Blue #10
Azur #4
Marimba #27
Jewel #23
Aurora #3
Southern Belle #43
Star #44
Biloxi #6
Compact #13
Mondo #29
Ozark Blue #34
Brigitta Blue #9
Primadonna #36
Top Hat #45
Elliott #19
Jubilee #24
Hortblue Poppins #35
Emerald #20
Atlantic #2
Roxy Blue #38
O'Neal #33
Aron #1
Bluecrop #7
Jersey #22
Centurion #11
Nui #32
Berkeley #5
Darrow #16
Northblue #30
Northland #31
Sky Blue #42
Misty #28
Chandler #12
Coville #15
Elizabeth #18
Cosmopolitan #14
Blue Moon #8
Earliblue #17
Liberty #26
Simultan #41
Toro #46
Puru #37
B
density
concentration, ppbV
index SI
C
H
PH
SI
Cluster
genetic cluster
1
2
3
4
n.a.

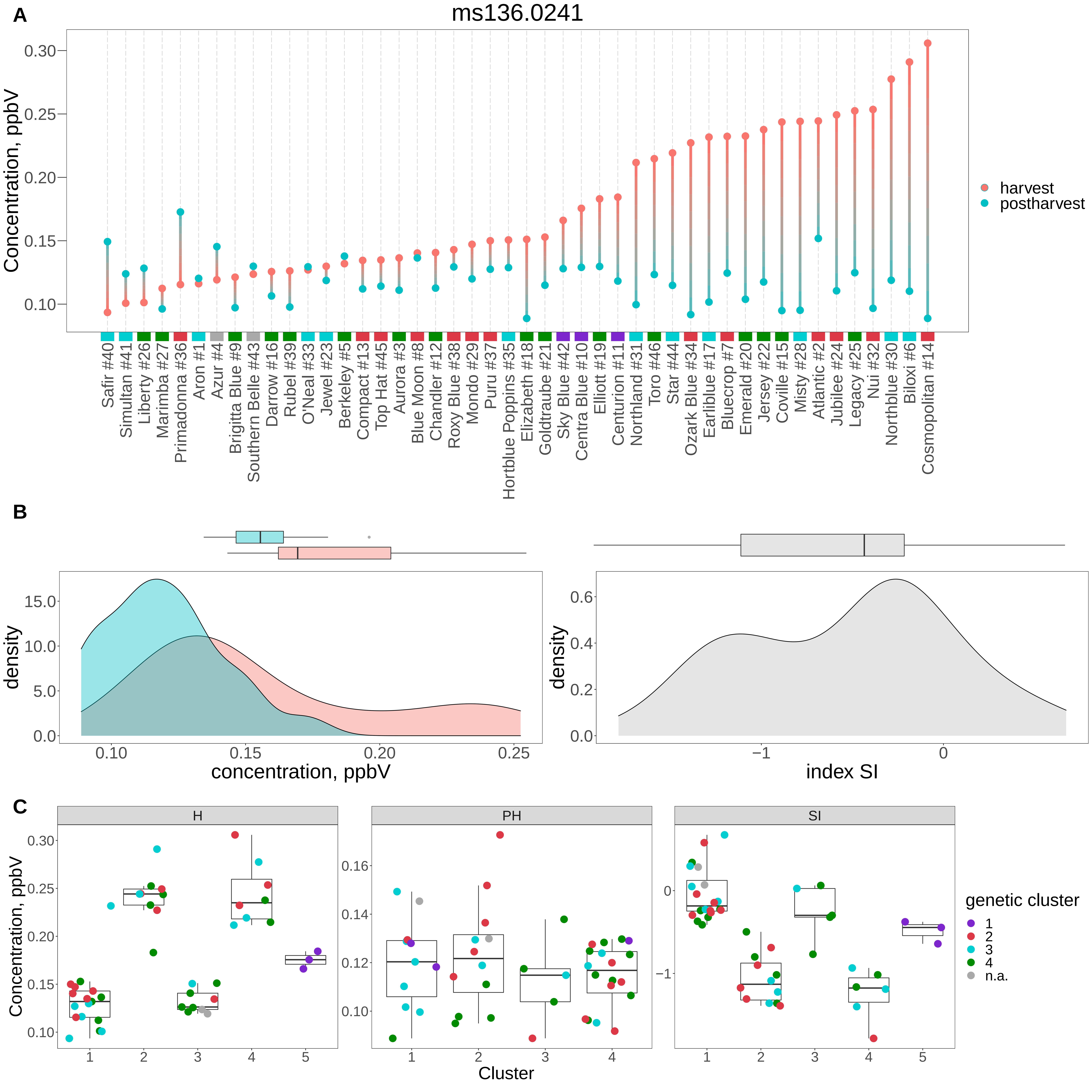

A
ms136.0241
Concentration, ppbV
0.30
0.25
0.20
0.15
0.10
harvest
postharvest
Safir #40
Simultan #41
Liberty #26
Marimba #27
Primadonna #36
Aron #1
Azur #4
Brigitta Blue #9
Southern Belle #43
Darrow #16
Rubel #39
O'Neal #33
Jewel #23
Berkeley #5
Compact #13
Top Hat #45
Aurora #3
Blue Moon #8
Chandler #12
Roxy Blue #38
Mondo #29
Puru #37
Hortblue Poppins #35
Elizabeth #18
Goldtraube #21
Sky Blue #42
Centra Blue #10
Elliott #19
Centurion #11
Northland #31
Toro #46
Star #44
Ozark Blue #34
Earliblue #17
Bluecrop #7
Emerald #20
Jersey #22
Coville #15
Misty #28
Atlantic #2
Jubilee #24
Legacy #25
Nui #32
Northblue #30
Biloxi #6
Cosmopolitan #14
B
density
15.0
10.0
5.0
0.0
0.10
0.15
0.20
0.25
concentration, ppbV
density
0.6
0.4
0.2
0.0
−1
0
index SI
C
H
PH
SI
Concentration, ppbV
0.30
0.25
0.20
0.15
0.10
0.16
0.14
0.12
0.10
0
−1
1
2
3
4
5
Cluster
genetic cluster
1
2
3
4
n.a.

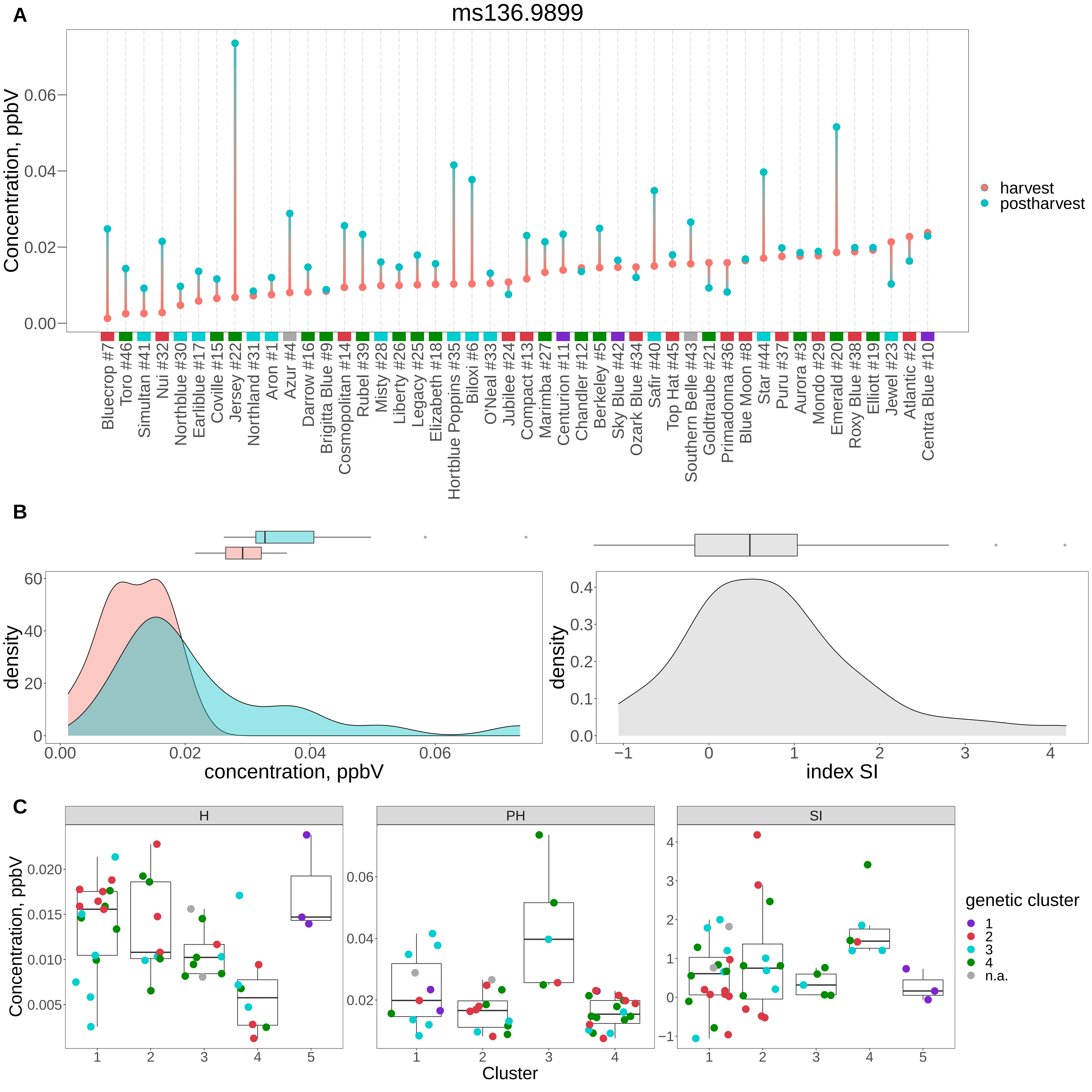
ms136.9899
A
B
C
Concentration, ppbV
harvest
postharvest
Bluecrop #7
Toro #46
Simultan #41
Nui #32
Northblue #30
Earliblue #17
Coville #15
Jersey #22
Northland #31
Aron #1
Azur #4
Darrow #16
Brigitta Blue #9
Cosmopolitan #14
Rubel #39
Misty #28
Liberty #26
Legacy #25
Elizabeth #18
Hortblue Poppins #35
Biloxi #6
O'Neal #33
Jubilee #24
Compact #13
Marimba #27
Centurion #11
Chandler #12
Berkeley #5
Sky Blue #42
Ozark Blue #34
Safir #40
Top Hat #45
Southern Belle #43
Goldtraube #21
Primadonna #36
Blue Moon #8
Star #44
Puru #37
Aurora #3
Mondo #29
Emerald #20
Roxy Blue #38
Elliott #19
Jewel #23
Atlantic #2
Centra Blue #10
density
concentration, ppbV
index SI
H
PH
SI
Cluster
genetic cluster
1
2
3
4
n.a.

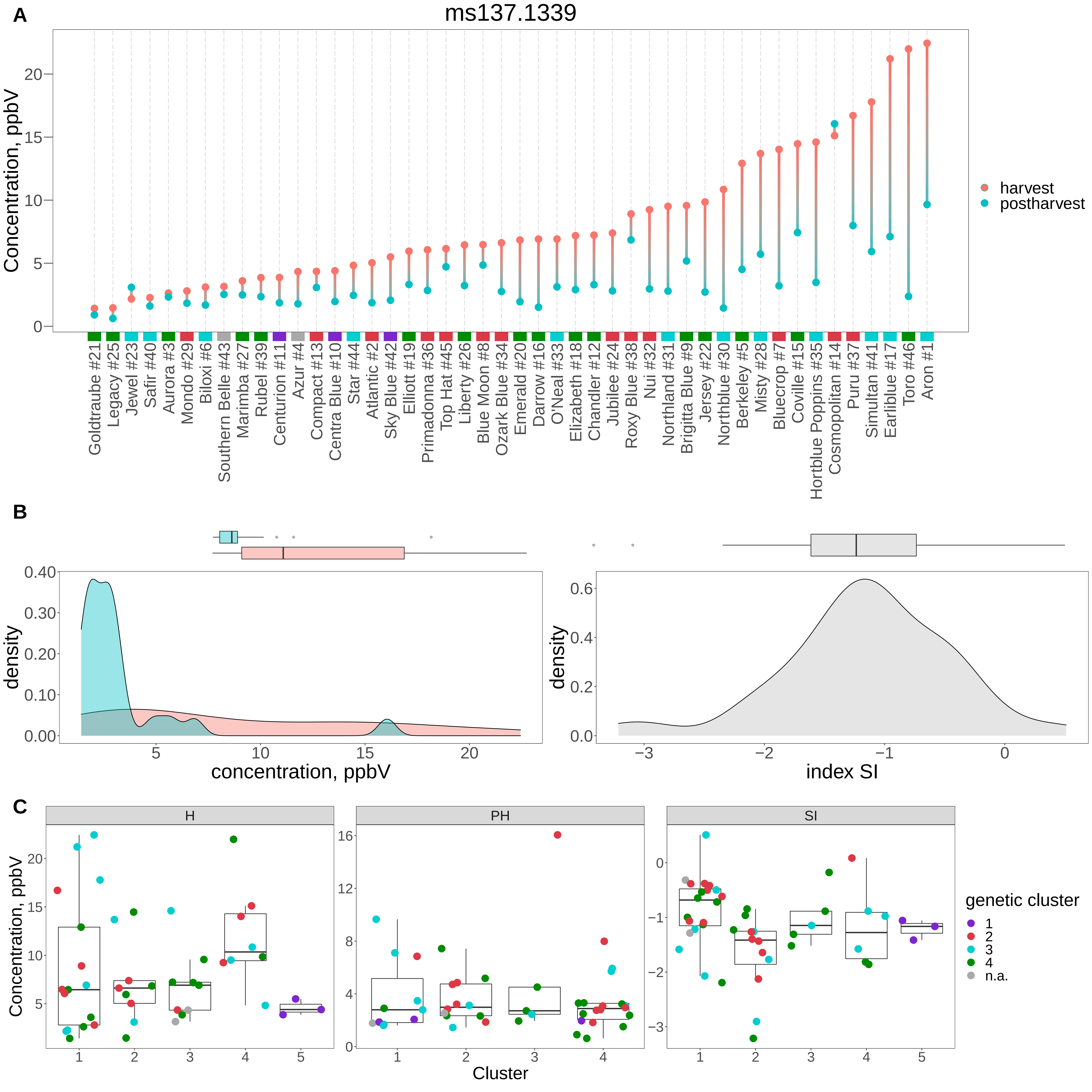

A
ms137.1339
Concentration, ppbV
harvest
postharvest
Goldtraube #21
Legacy #25
Jewel #23
Safir #40
Aurora #3
Mondo #29
Biloxi #6
Southern Belle #43
Marimba #27
Rubel #39
Centurion #11
Azur #4
Compact #13
Centra Blue #10
Star #44
Atlantic #2
Sky Blue #42
Elliott #19
Primadonna #36
Top Hat #45
Liberty #26
Blue Moon #8
Ozark Blue #34
Emerald #20
Darrow #16
O'Neal #33
Elizabeth #18
Chandler #12
Jubilee #24
Roxy Blue #38
Nui #32
Northland #31
Brigitta Blue #9
Jersey #22
Northblue #30
Berkeley #5
Misty #28
Bluecrop #7
Coville #15
Hortblue Poppins #35
Cosmopolitan #14
Puru #37
Simultan #41
Earliblue #17
Toro #46
Aron #1
B
density
concentration, ppbV
index SI
C
H
PH
SI
Concentration, ppbV
Cluster
genetic cluster
1
2
3
4
n.a.

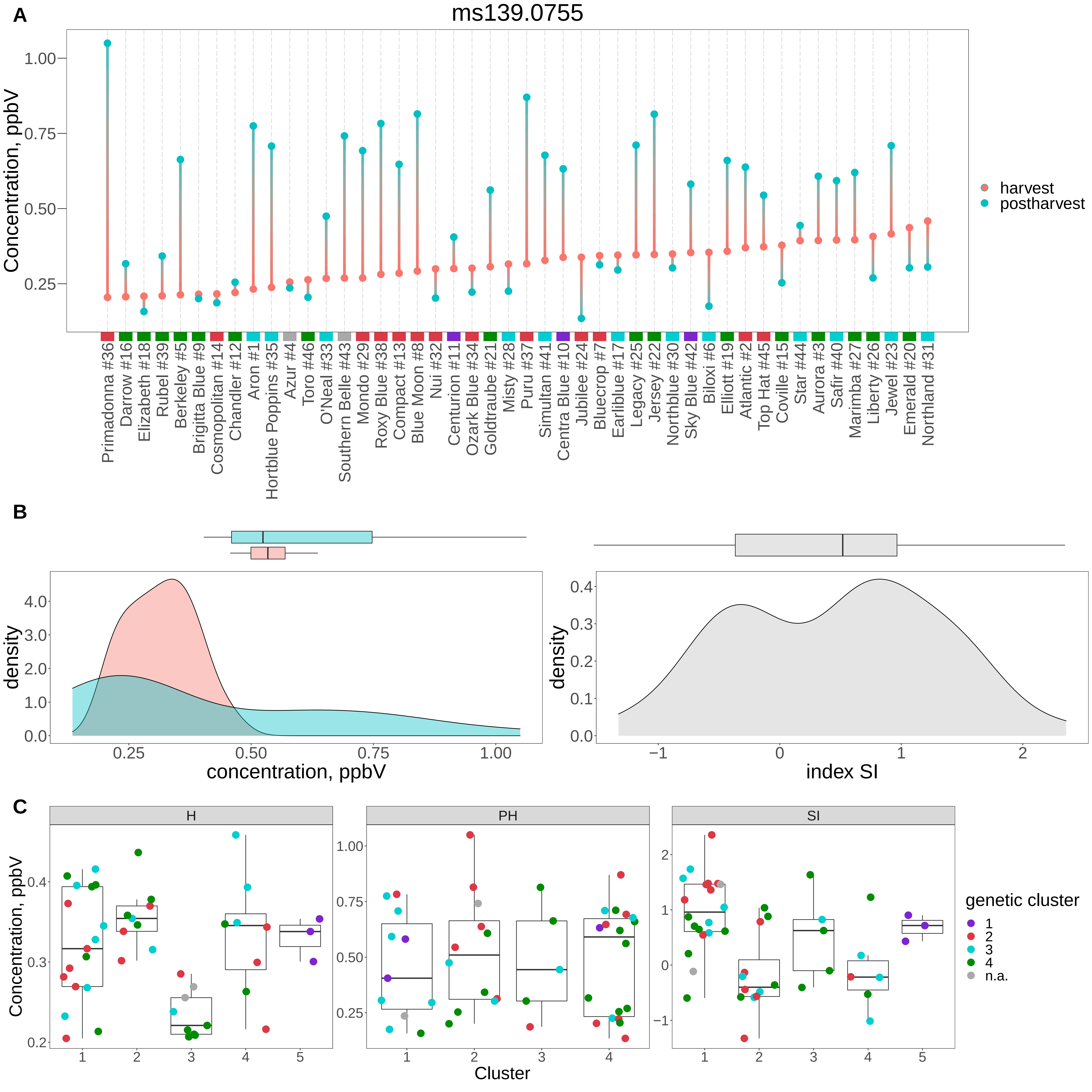
A
ms139.0755
Concentration, ppbV
harvest
postharvest
Primadonna #36
Darrow #16
Elizabeth #18
Rubel #39
Berkeley #5
Brigitta Blue #9
Cosmopolitan #14
Chandler #12
Aron #1
Hortblue Poppins #35
Azur #4
Toro #46
O'Neal #33
Southern Belle #43
Mondo #29
Roxy Blue #38
Compact #13
Blue Moon #8
Nui #32
Centurion #11
Ozark Blue #34
Goldtraube #21
Misty #28
Puru #37
Simultan #41
Centra Blue #10
Jubilee #24
Bluecrop #7
Earliblue #17
Legacy #25
Jersey #22
Northblue #30
Sky Blue #42
Biloxi #6
Elliott #19
Atlantic #2
Top Hat #45
Coville #15
Star #44
Aurora #3
Safir #40
Marimba #27
Liberty #26
Jewel #23
Emerald #20
Northland #31
B
density
concentration, ppbV
index SI
C
H
PH
SI
Concentration, ppbV
Cluster
genetic cluster
n.a.

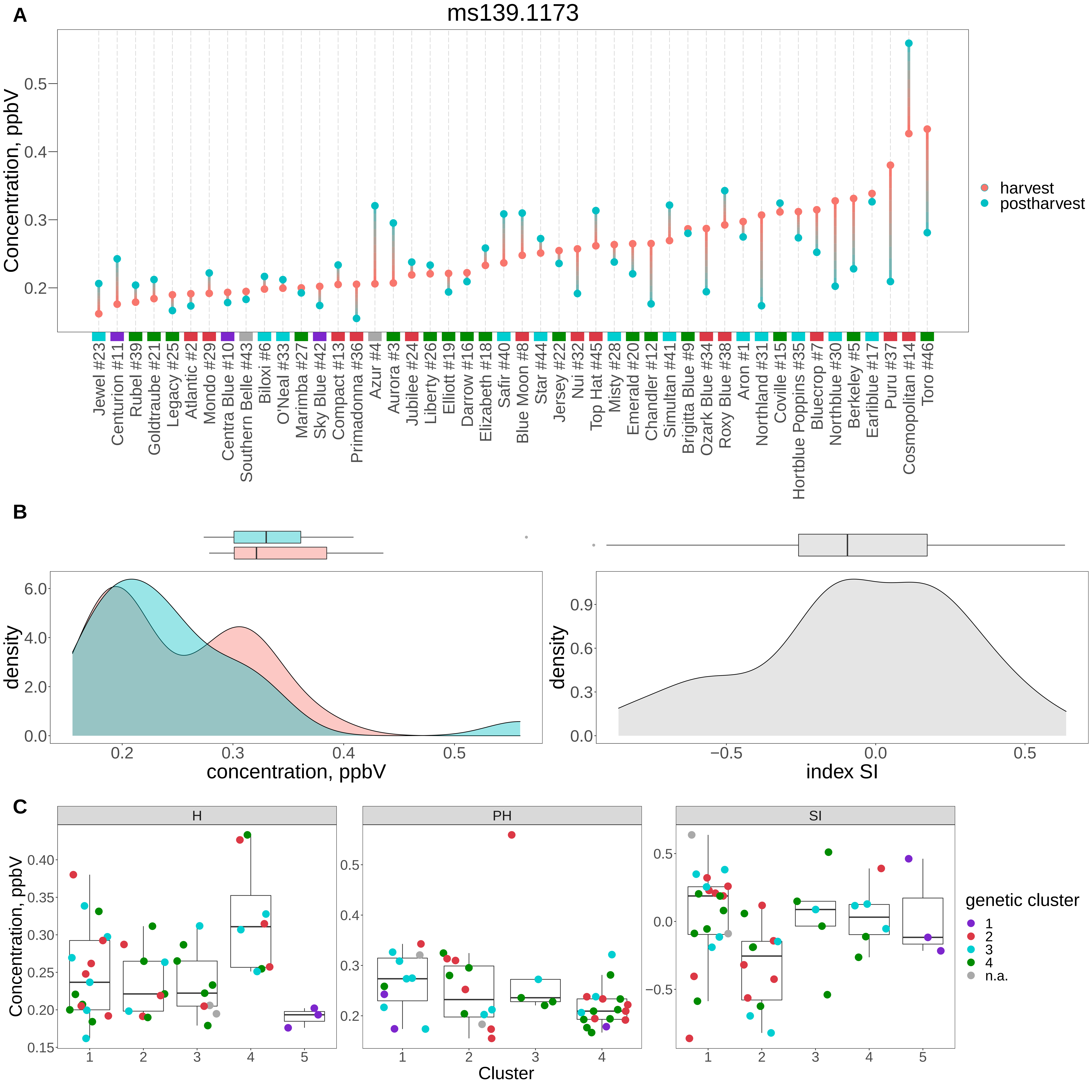
A
ms139.1173
Concentration, ppbV
harvest
postharvest
Jewel #23
Centurion #11
Rubel #39
Goldtraube #21
Legacy #25
Atlantic #2
Mondo #29
Centra Blue #10
Southern Belle #43
Biloxi #6
O'Neal #33
Marimba #27
Sky Blue #42
Compact #13
Primadonna #36
Azur #4
Aurora #3
Jubilee #24
Liberty #26
Elliott #19
Darrow #16
Elizabeth #18
Safir #40
Blue Moon #8
Star #44
Jersey #22
Nui #32
Top Hat #45
Misty #28
Emerald #20
Chandler #12
Simultan #41
Brigitta Blue #9
Ozark Blue #34
Roxy Blue #38
Aron #1
Northland #31
Coville #15
Hortblue Poppins #35
Bluecrop #7
Northblue #30
Berkeley #5
Earliblue #17
Puru #37
Cosmopolitan #14
Toro #46
B
density
concentration, ppbV
index SI
C
H
PH
SI
Concentration, ppbV
Cluster
genetic cluster
1
2
3
4
n.a.

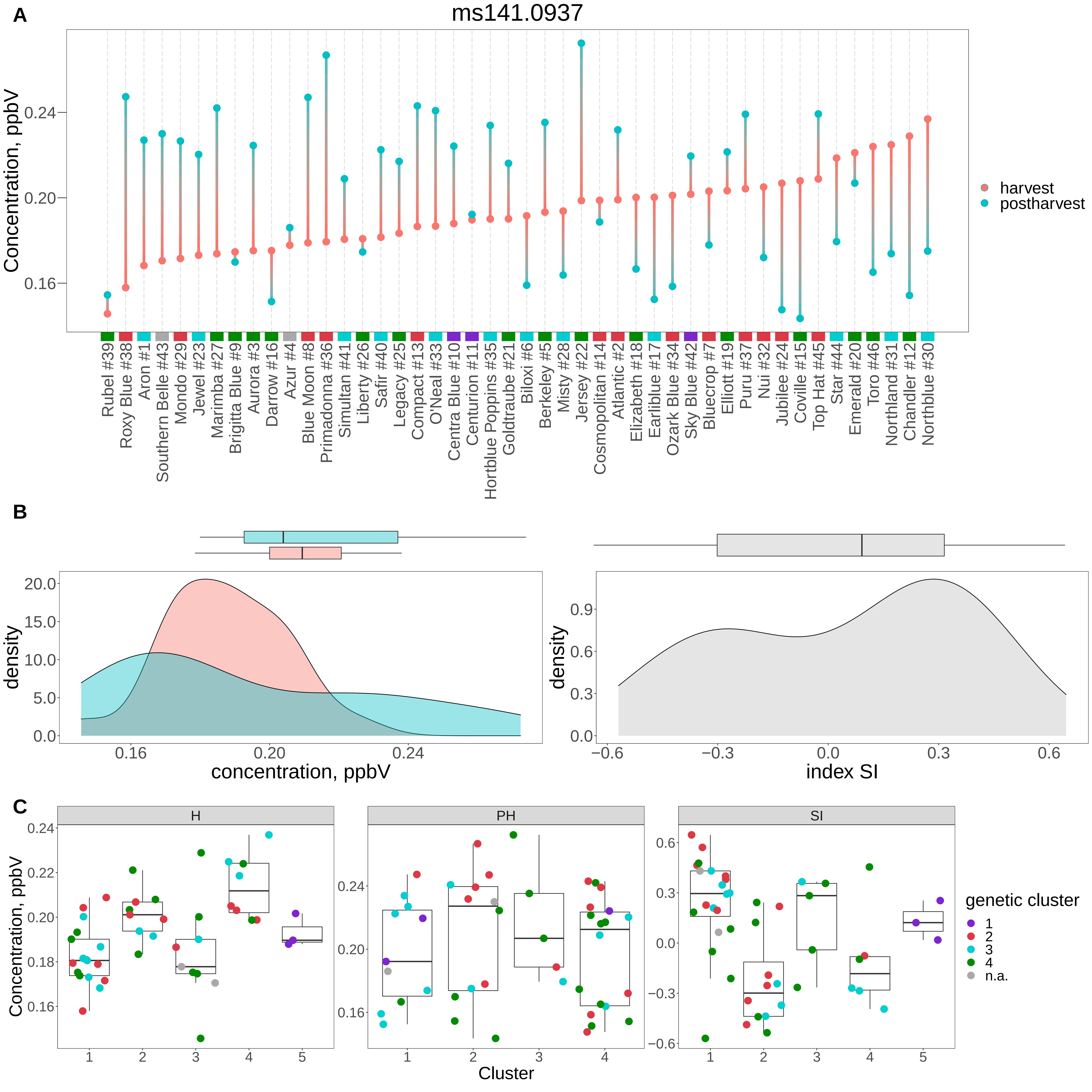
A
ms141.0937
Concentration, ppbV
harvest
postharvest
Rubel #39
Roxy Blue #38
Aron #1
Southern Belle #43
Mondo #29
Jewel #23
Marimba #27
Brigitta Blue #9
Aurora #3
Darrow #16
Azur #4
Blue Moon #8
Primadonna #36
Simultan #41
Liberty #26
Safir #40
Legacy #25
Compact #13
O'Neal #33
Centra Blue #10
Centurion #11
Hortblue Poppins #35
Goldtraube #21
Biloxi #6
Berkeley #5
Misty #28
Jersey #22
Cosmopolitan #14
Atlantic #2
Elizabeth #18
Earliblue #17
Ozark Blue #34
Sky Blue #42
Bluecrop #7
Elliott #19
Puru #37
Nui #32
Jubilee #24
Coville #15
Top Hat #45
Star #44
Emerald #20
Toro #46
Northland #31
Chandler #12
Northblue #30
B
density
concentration, ppbV
index SI
C
H
PH
SI
Concentration, ppbV
Cluster
genetic cluster
1
2
3
4
n.a.

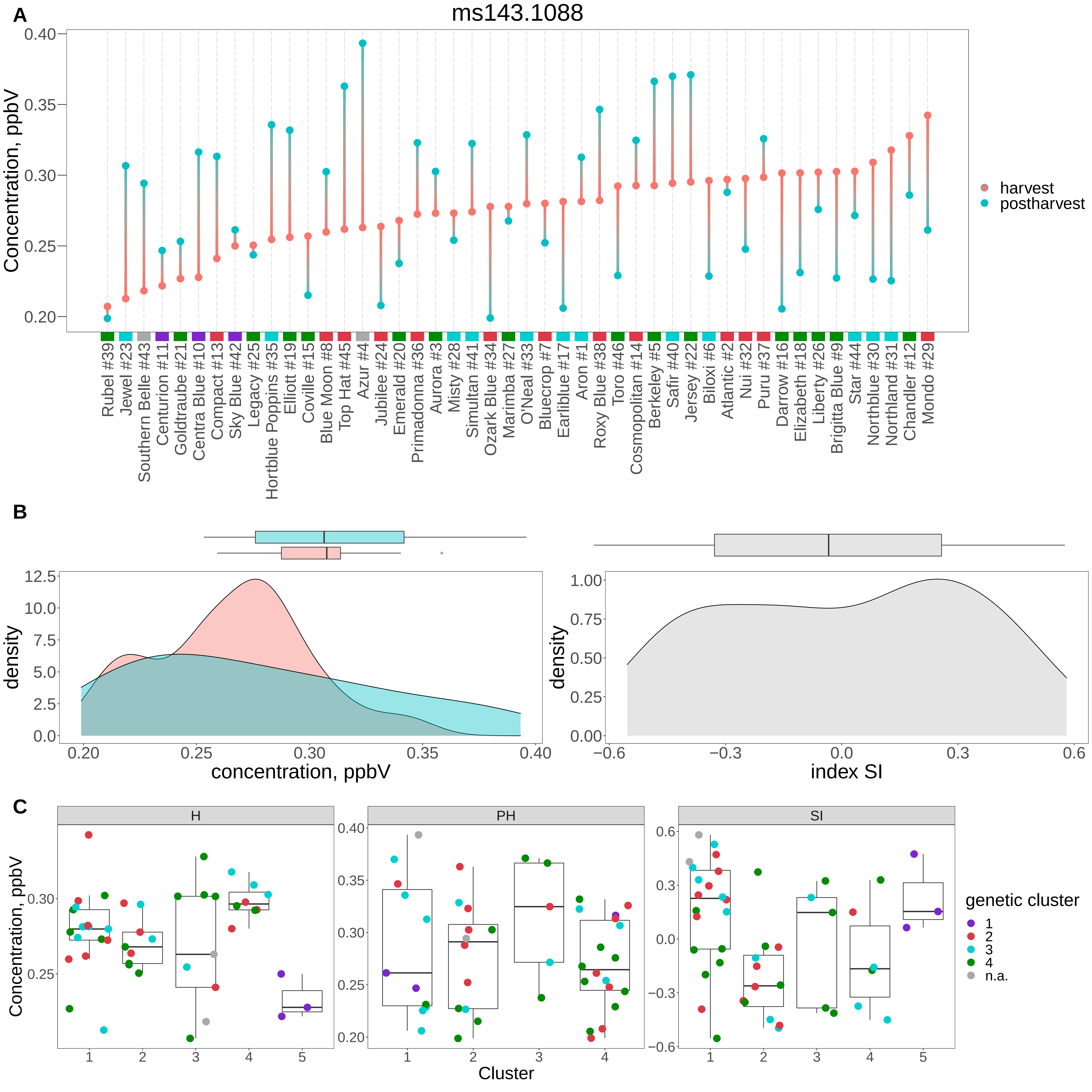
A
ms143.1088
Concentration, ppbV
harvest
postharvest
Rubel #39
Jewel #23
Southern Belle #43
Centurion #11
Goldtraube #21
Centra Blue #10
Compact #13
Sky Blue #42
Legacy #25
Hortblue Poppins #35
Elliott #19
Coville #15
Blue Moon #8
Top Hat #45
Azur #4
Jubilee #24
Emerald #20
Primadonna #36
Aurora #3
Misty #28
Simultan #41
Ozark Blue #34
Marimba #27
O'Neal #33
Bluecrop #7
Earliblue #17
Aron #1
Roxy Blue #38
Toro #46
Cosmopolitan #14
Berkeley #5
Safir #40
Jersey #22
Biloxi #6
Atlantic #2
Nui #32
Puru #37
Darrow #16
Elizabeth #18
Liberty #26
Brigitta Blue #9
Star #44
Northblue #30
Northland #31
Chandler #12
Mondo #29
B
density
concentration, ppbV
index SI
C
H
PH
SI
Concentration, ppbV
Cluster
genetic cluster
1
2
3
4
n.a.

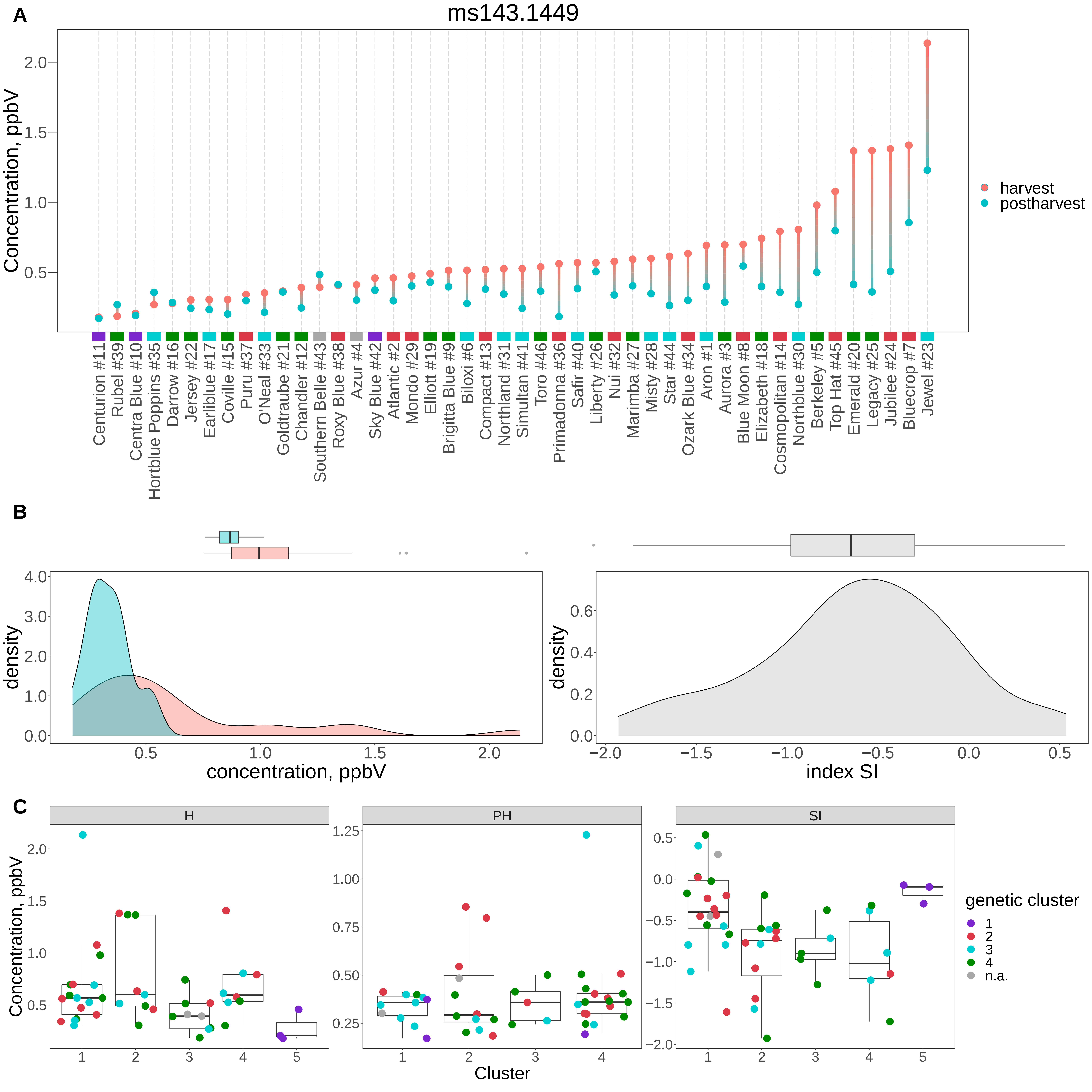

A
ms143.1449
Concentration, ppbV
harvest
postharvest
Centurion #11
Rubel #39
Centra Blue #10
Hortblue Poppins #35
Darrow #16
Jersey #22
Earliblue #17
Coville #15
Puru #37
O'Neal #33
Goldtraube #21
Chandler #12
Southern Belle #43
Roxy Blue #38
Azur #4
Sky Blue #42
Atlantic #2
Mondo #29
Elliott #19
Brigitta Blue #9
Biloxi #6
Compact #13
Northland #31
Simultan #41
Toro #46
Primadonna #36
Safir #40
Liberty #26
Nui #32
Marimba #27
Misty #28
Star #44
Ozark Blue #34
Aron #1
Aurora #3
Blue Moon #8
Elizabeth #18
Cosmopolitan #14
Northblue #30
Berkeley #5
Top Hat #45
Emerald #20
Legacy #25
Jubilee #24
Bluecrop #7
Jewel #23
B
density
concentration, ppbV
density
index SI
C
H
PH
SI
Concentration, ppbV
Cluster
genetic cluster
1
2
3
4
n.a.

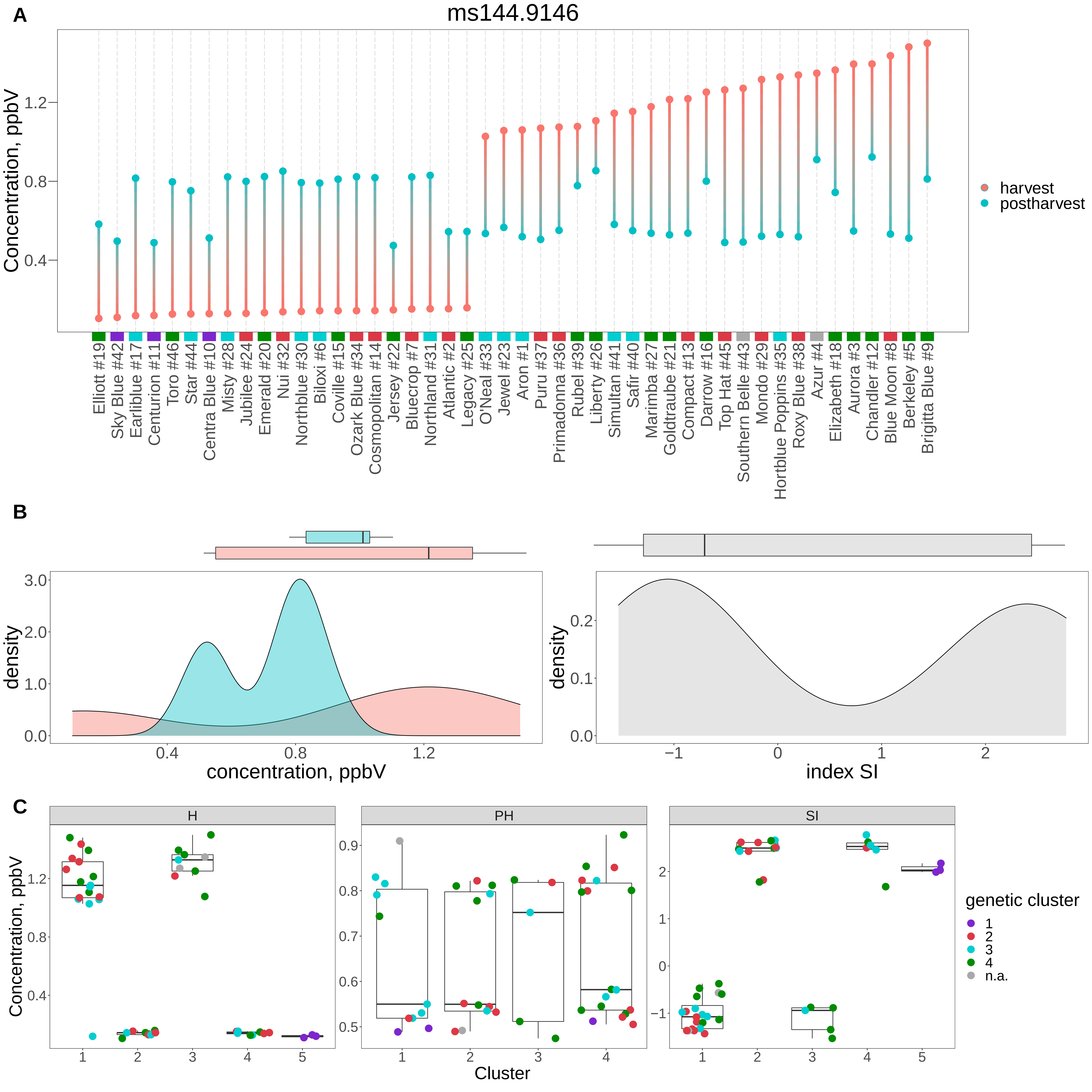
A
ms144.9146
Concentration, ppbV
harvest
postharvest
Elliott #19
Sky Blue #42
Earliblue #17
Centurion #11
Toro #46
Star #44
Centra Blue #10
Misty #28
Jubilee #24
Emerald #20
Nui #32
Northblue #30
Biloxi #6
Coville #15
Ozark Blue #34
Cosmopolitan #14
Jersey #22
Bluecrop #7
Northland #31
Atlantic #2
Legacy #25
O'Neal #33
Jewel #23
Aron #1
Puru #37
Primadonna #36
Rubel #39
Liberty #26
Simultan #41
Safir #40
Marimba #27
Goldtraube #21
Compact #13
Darrow #16
Top Hat #45
Southern Belle #43
Mondo #29
Hortblue Poppins #35
Roxy Blue #38
Azur #4
Elizabeth #18
Aurora #3
Chandler #12
Blue Moon #8
Berkeley #5
Brigitta Blue #9
B
density
concentration, ppbV
index SI
C
H
PH
SI
Cluster
genetic cluster
1
2
3
4
n.a.

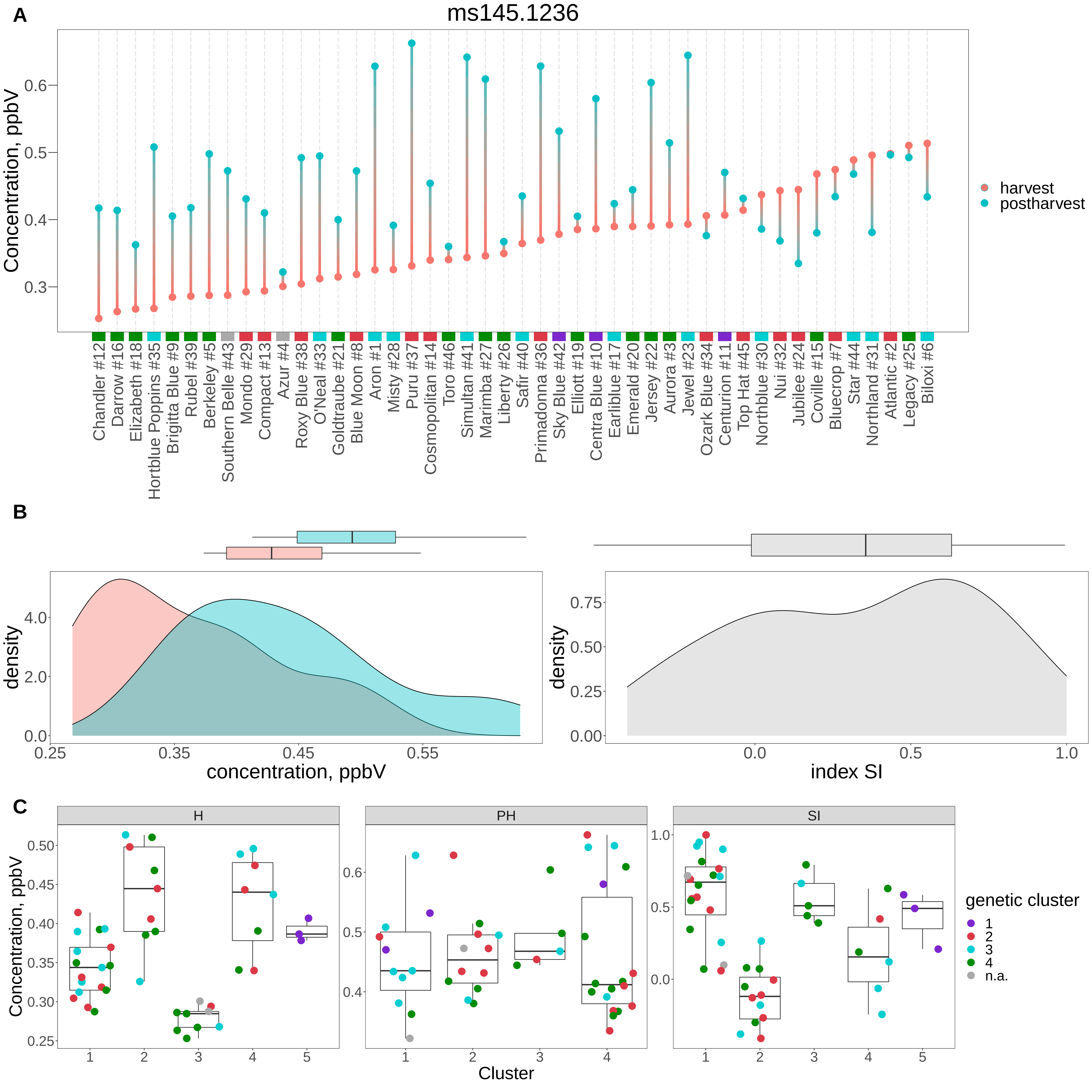

A
ms145.1236
Concentration, ppbV
harvest
postharvest
Chandler #12
Darrow #16
Elizabeth #18
Hortblue Poppins #35
Brigitta Blue #9
Rubel #39
Berkeley #5
Southern Belle #43
Mondo #29
Compact #13
Azur #4
Roxy Blue #38
O'Neal #33
Goldtraube #21
Blue Moon #8
Aron #1
Misty #28
Puru #37
Cosmopolitan #14
Toro #46
Simultan #41
Marimba #27
Liberty #26
Safir #40
Primadonna #36
Sky Blue #42
Elliott #19
Centra Blue #10
Earliblue #17
Emerald #20
Jersey #22
Aurora #3
Jewel #23
Ozark Blue #34
Centurion #11
Top Hat #45
Northblue #30
Nui #32
Jubilee #24
Coville #15
Bluecrop #7
Star #44
Northland #31
Atlantic #2
Legacy #25
Biloxi #6
B
density
concentration, ppbV
index SI
C
H
PH
SI
Concentration, ppbV
Cluster
genetic cluster
1
2
3
4
n.a.

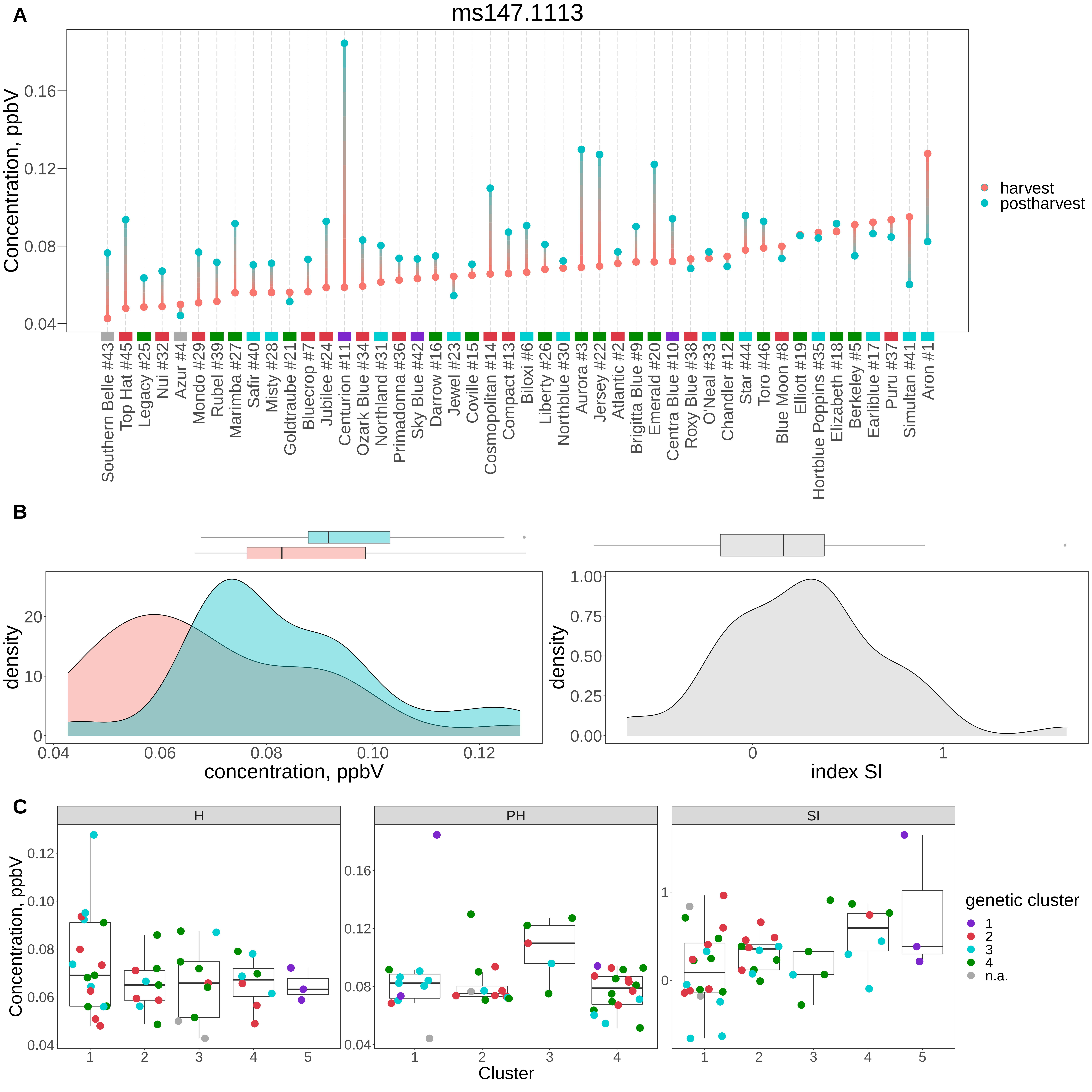

A
ms147.1113
Concentration, ppbV
harvest
postharvest
Southern Belle #43
Top Hat #45
Legacy #25
Nui #32
Azur #4
Mondo #29
Rubel #39
Marimba #27
Safir #40
Misty #28
Goldtraube #21
Bluecrop #7
Jubilee #24
Centurion #11
Ozark Blue #34
Northland #31
Primadonna #36
Sky Blue #42
Darrow #16
Jewel #23
Coville #15
Cosmopolitan #14
Compact #13
Biloxi #6
Liberty #26
Northblue #30
Aurora #3
Jersey #22
Atlantic #2
Brigitta Blue #9
Emerald #20
Centra Blue #10
Roxy Blue #38
O'Neal #33
Chandler #12
Star #44
Toro #46
Blue Moon #8
Elliott #19
Hortblue Poppins #35
Elizabeth #18
Berkeley #5
Earliblue #17
Puru #37
Simultan #41
Aron #1
B
density
concentration, ppbV
index SI
C
H
PH
SI
Concentration, ppbV
Cluster
genetic cluster
1
2
3
4
n.a.

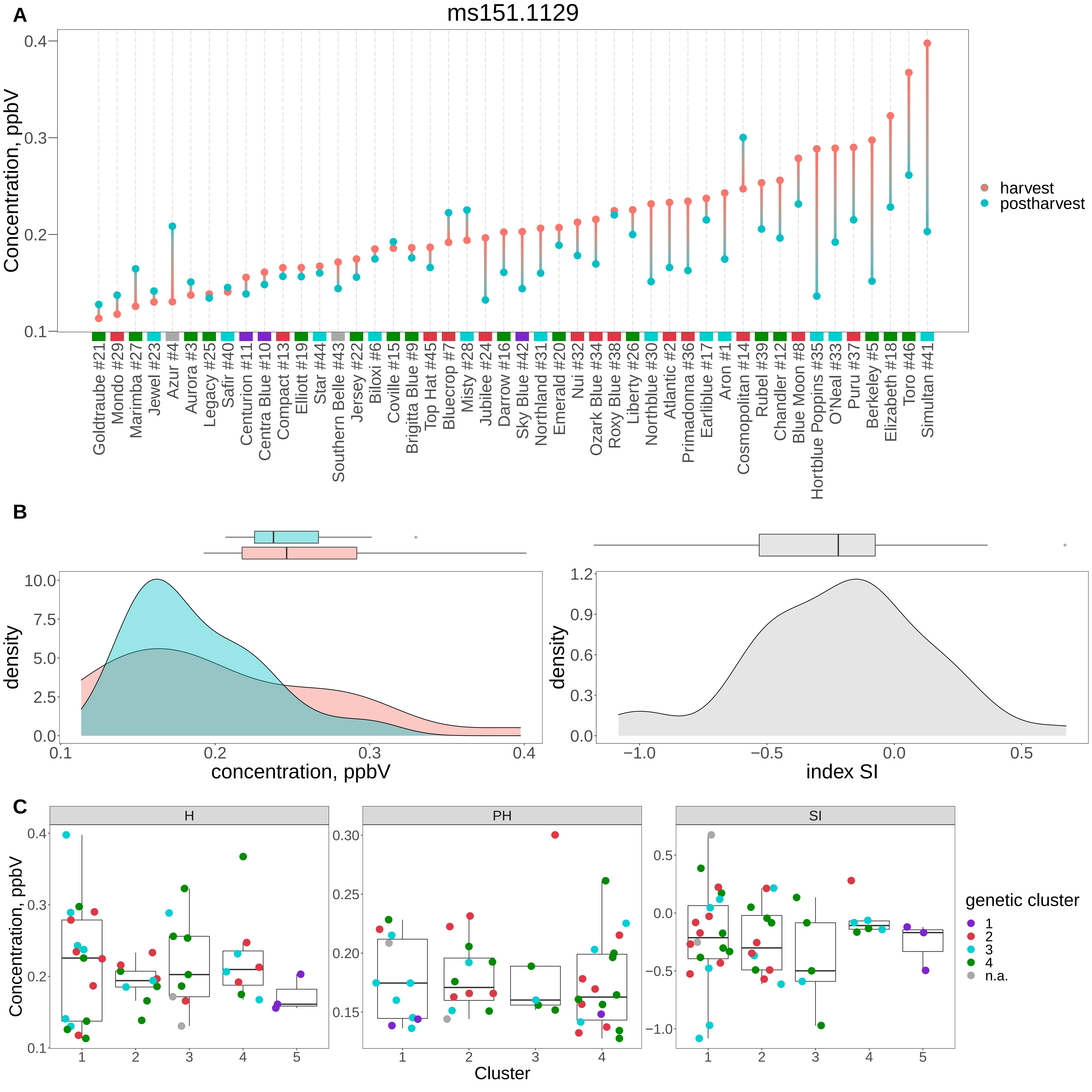
A
ms151.1129
Concentration, ppbV
harvest
postharvest
Goldtraube #21
Mondo #29
Marimba #27
Jewel #23
Azur #4
Aurora #3
Legacy #25
Safir #40
Centurion #11
Centra Blue #10
Compact #13
Elliott #19
Star #44
Southern Belle #43
Jersey #22
Biloxi #6
Coville #15
Brigitta Blue #9
Top Hat #45
Bluecrop #7
Misty #28
Jubilee #24
Darrow #16
Sky Blue #42
Northland #31
Emerald #20
Nui #32
Ozark Blue #34
Roxy Blue #38
Liberty #26
Northblue #30
Atlantic #2
Primadonna #36
Earliblue #17
Aron #1
Cosmopolitan #14
Rubel #39
Chandler #12
Blue Moon #8
Hortblue Poppins #35
O'Neal #33
Puru #37
Berkeley #5
Elizabeth #18
Toro #46
Simultan #41
B
density
concentration, ppbV
index SI
C
H
PH
SI
Cluster
genetic cluster
1
2
3
4
n.a.

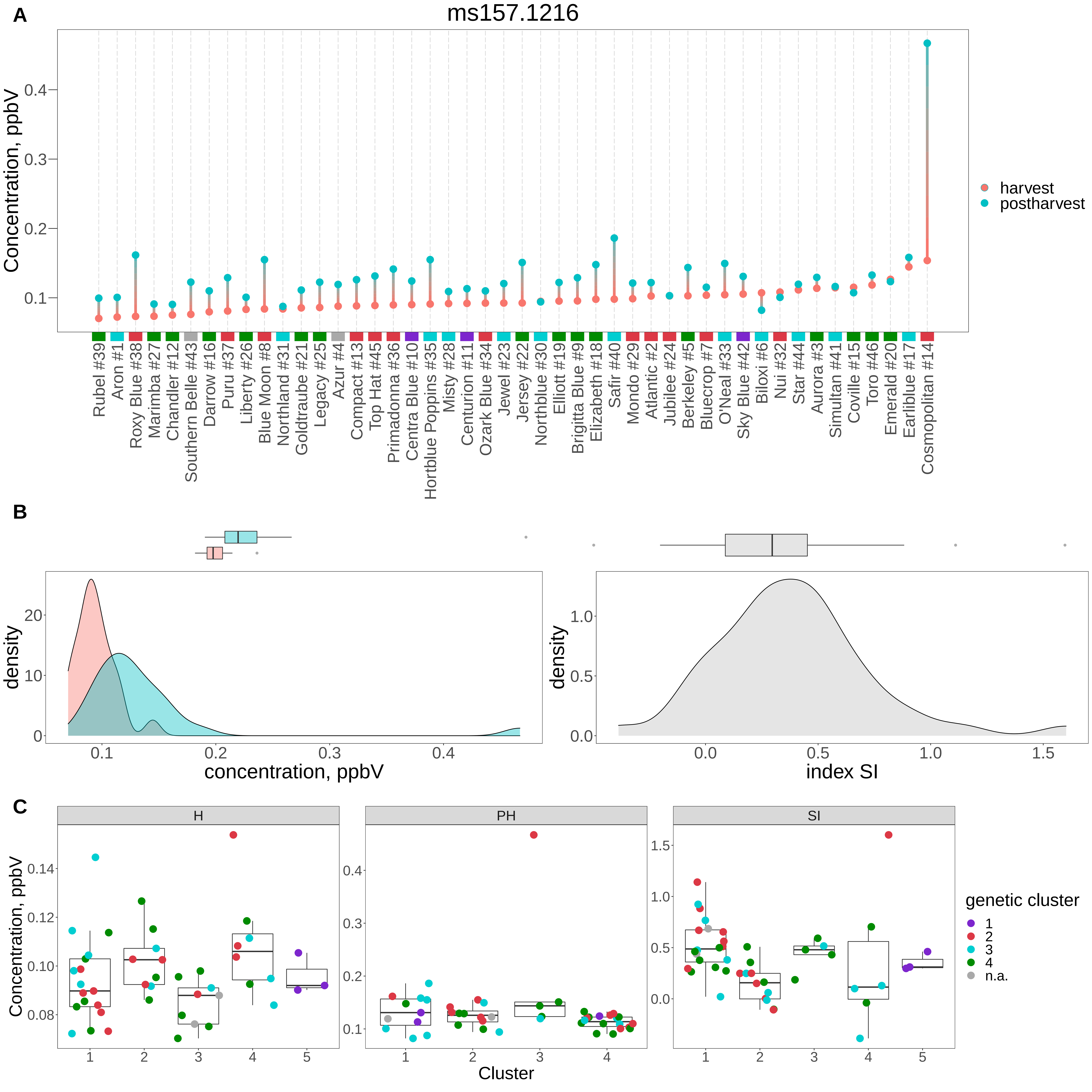

A
ms157.1216
Concentration, ppbV
harvest
postharvest
Rubel #39
Aron #1
Roxy Blue #38
Marimba #27
Chandler #12
Southern Belle #43
Darrow #16
Puru #37
Liberty #26
Blue Moon #8
Northland #31
Goldtraube #21
Legacy #25
Azur #4
Compact #13
Top Hat #45
Primadonna #36
Centra Blue #10
Hortblue Poppins #35
Misty #28
Centurion #11
Ozark Blue #34
Jewel #23
Jersey #22
Northblue #30
Elliott #19
Brigitta Blue #9
Elizabeth #18
Safir #40
Mondo #29
Atlantic #2
Jubilee #24
Berkeley #5
Bluecrop #7
O'Neal #33
Sky Blue #42
Biloxi #6
Nui #32
Star #44
Aurora #3
Simultan #41
Coville #15
Toro #46
Emerald #20
Earliblue #17
Cosmopolitan #14
B
density
concentration, ppbV
index SI
C
H
PH
SI
Concentration, ppbV
Cluster
genetic cluster
1
2
3
4
n.a.

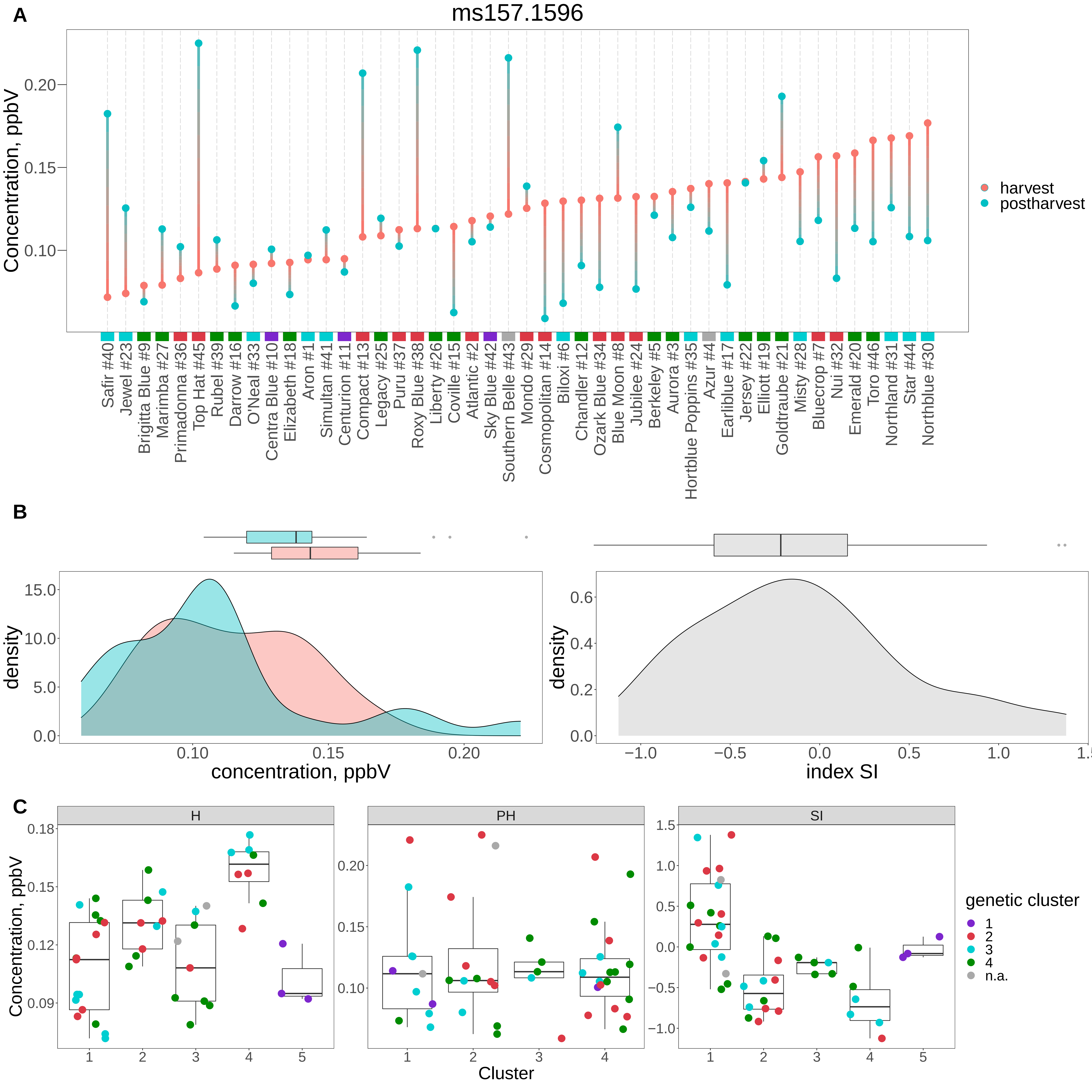
A
ms157.1596
Concentration, ppbV
0.20
0.15
0.10
harvest
postharvest
Safir #40
Jewel #23
Brigitta Blue #9
Marimba #27
Primadonna #36
Top Hat #45
Rubel #39
Darrow #16
O'Neal #33
Centra Blue #10
Elizabeth #18
Aron #1
Simultan #41
Centurion #11
Compact #13
Legacy #25
Puru #37
Roxy Blue #38
Liberty #26
Coville #15
Atlantic #2
Sky Blue #42
Southern Belle #43
Mondo #29
Cosmopolitan #14
Biloxi #6
Chandler #12
Ozark Blue #34
Blue Moon #8
Jubilee #24
Berkeley #5
Aurora #3
Hortblue Poppins #35
Azur #4
Earliblue #17
Jersey #22
Elliott #19
Goldtraube #21
Misty #28
Bluecrop #7
Nui #32
Emerald #20
Toro #46
Northland #31
Star #44
Northblue #30
B
density
15.0
10.0
5.0
0.0
0.10
0.15
0.20
concentration, ppbV
density
0.6
0.4
0.2
0.0
−1.0
−0.5
0.0
0.5
1.0
index SI
C
H
PH
SI
Concentration, ppbV
0.18
0.15
0.12
0.09
0.20
0.15
0.10
1.5
1.0
0.5
0.0
−0.5
−1.0
1
2
3
4
5
Cluster
genetic cluster
1
2
3
4
n.a.

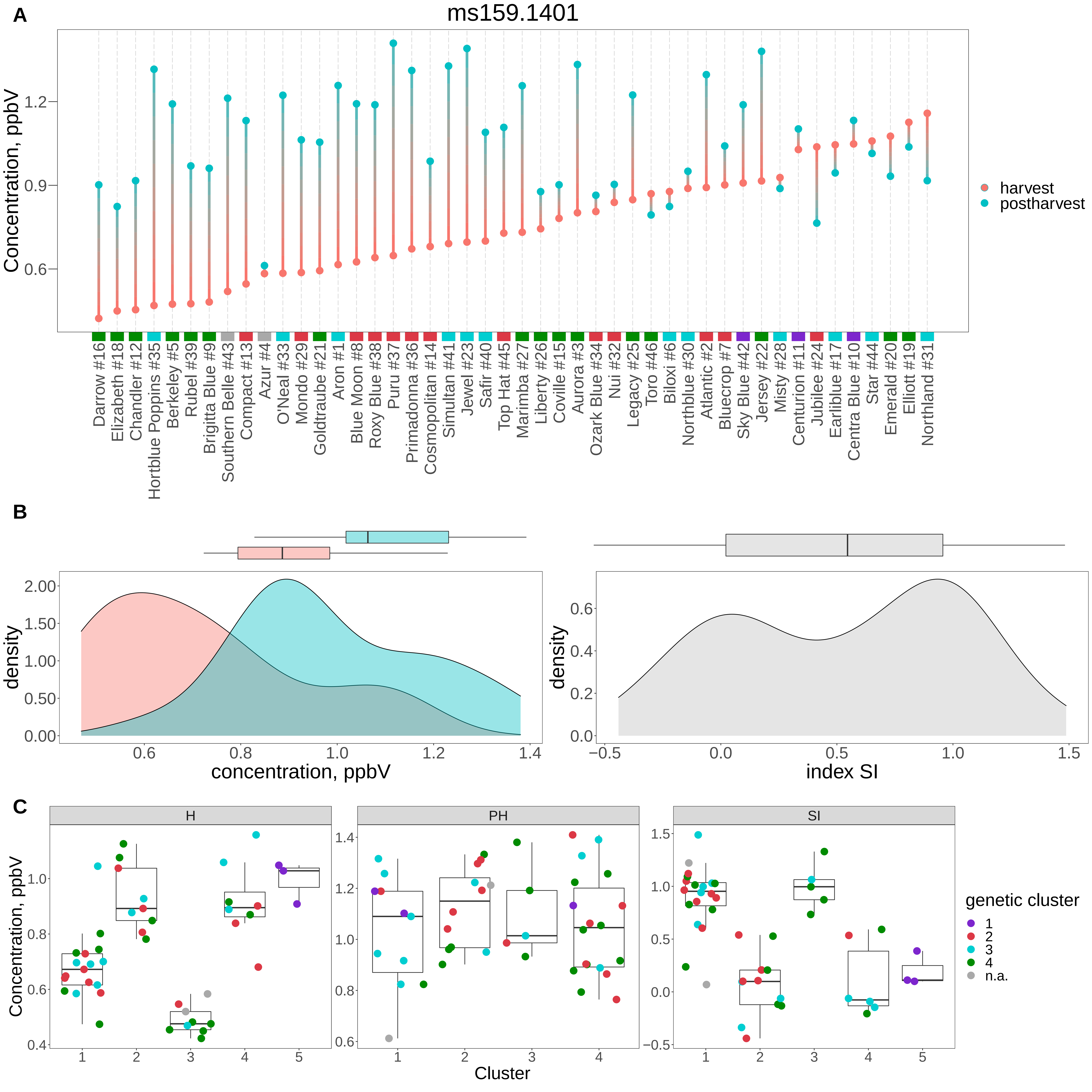

A
ms159.1401
Concentration, ppbV
harvest
postharvest
Darrow #16
Elizabeth #18
Chandler #12
Hortblue Poppins #35
Berkeley #5
Rubel #39
Brigitta Blue #9
Southern Belle #43
Compact #13
Azur #4
O'Neal #33
Mondo #29
Goldtraube #21
Aron #1
Blue Moon #8
Roxy Blue #38
Puru #37
Primadonna #36
Cosmopolitan #14
Simultan #41
Jewel #23
Safir #40
Top Hat #45
Marimba #27
Liberty #26
Coville #15
Aurora #3
Ozark Blue #34
Nui #32
Legacy #25
Toro #46
Biloxi #6
Northblue #30
Atlantic #2
Bluecrop #7
Sky Blue #42
Jersey #22
Misty #28
Centurion #11
Jubilee #24
Earliblue #17
Centra Blue #10
Star #44
Emerald #20
Elliott #19
Northland #31
B
density
concentration, ppbV
index SI
C
H
PH
SI
Concentration, ppbV
Cluster
genetic cluster
1
2
3
4
n.a.

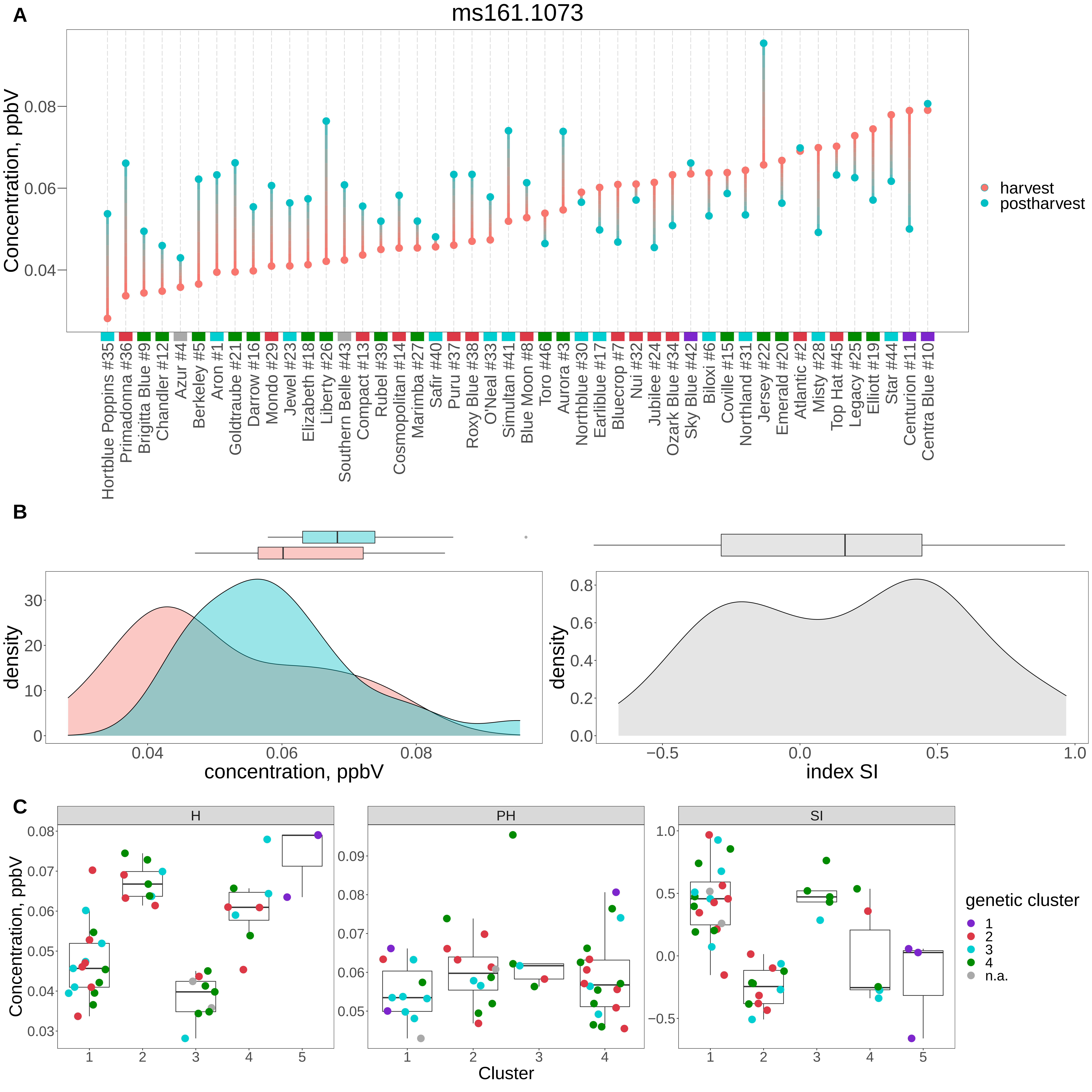
A
ms161.1073
Concentration, ppbV
harvest
postharvest
Hortblue Poppins #35
Primadonna #36
Brigitta Blue #9
Chandler #12
Azur #4
Berkeley #5
Aron #1
Goldtraube #21
Darrow #16
Mondo #29
Jewel #23
Elizabeth #18
Liberty #26
Southern Belle #43
Compact #13
Rubel #39
Cosmopolitan #14
Marimba #27
Safir #40
Puru #37
Roxy Blue #38
O'Neal #33
Simultan #41
Blue Moon #8
Toro #46
Aurora #3
Northblue #30
Earliblue #17
Bluecrop #7
Nui #32
Jubilee #24
Ozark Blue #34
Sky Blue #42
Biloxi #6
Coville #15
Northland #31
Jersey #22
Emerald #20
Atlantic #2
Misty #28
Top Hat #45
Legacy #25
Elliott #19
Star #44
Centurion #11
Centra Blue #10
B
density
concentration, ppbV
index SI
C
H
PH
SI
Concentration, ppbV
Cluster
genetic cluster
1
2
3
4
n.a.

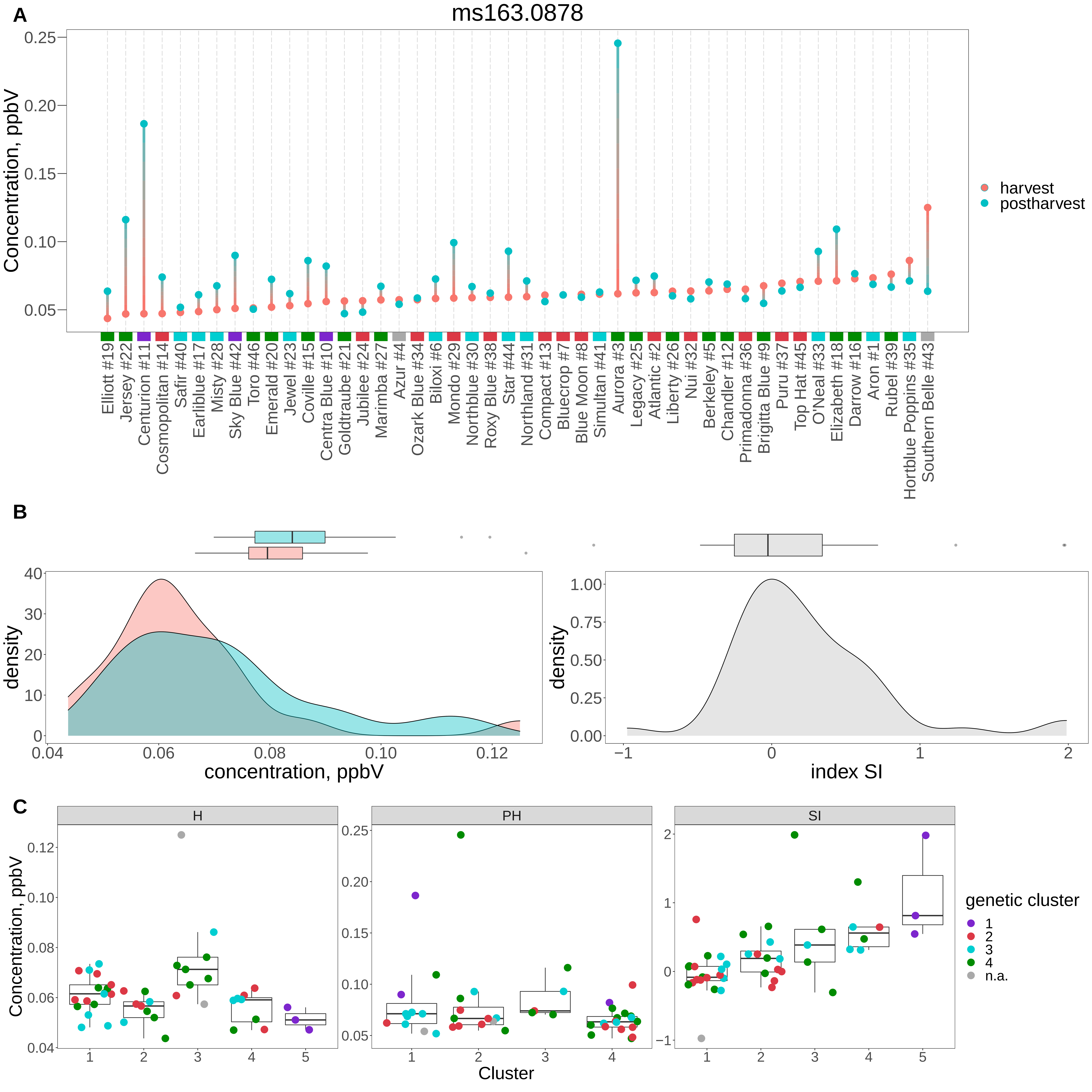

A
ms163.0878
Concentration, ppbV
harvest
postharvest
Elliott #19
Jersey #22
Centurion #11
Cosmopolitan #14
Safir #40
Earliblue #17
Misty #28
Sky Blue #42
Toro #46
Emerald #20
Jewel #23
Coville #15
Centra Blue #10
Goldtraube #21
Jubilee #24
Marimba #27
Azur #4
Ozark Blue #34
Biloxi #6
Mondo #29
Northblue #30
Roxy Blue #38
Star #44
Northland #31
Compact #13
Bluecrop #7
Blue Moon #8
Simultan #41
Aurora #3
Legacy #25
Atlantic #2
Liberty #26
Nui #32
Berkeley #5
Chandler #12
Primadonna #36
Brigitta Blue #9
Puru #37
Top Hat #45
O'Neal #33
Elizabeth #18
Darrow #16
Aron #1
Rubel #39
Hortblue Poppins #35
Southern Belle #43
B
density
concentration, ppbV
index SI
C
H
PH
SI
Concentration, ppbV
Cluster
genetic cluster
1
2
3
4
n.a.

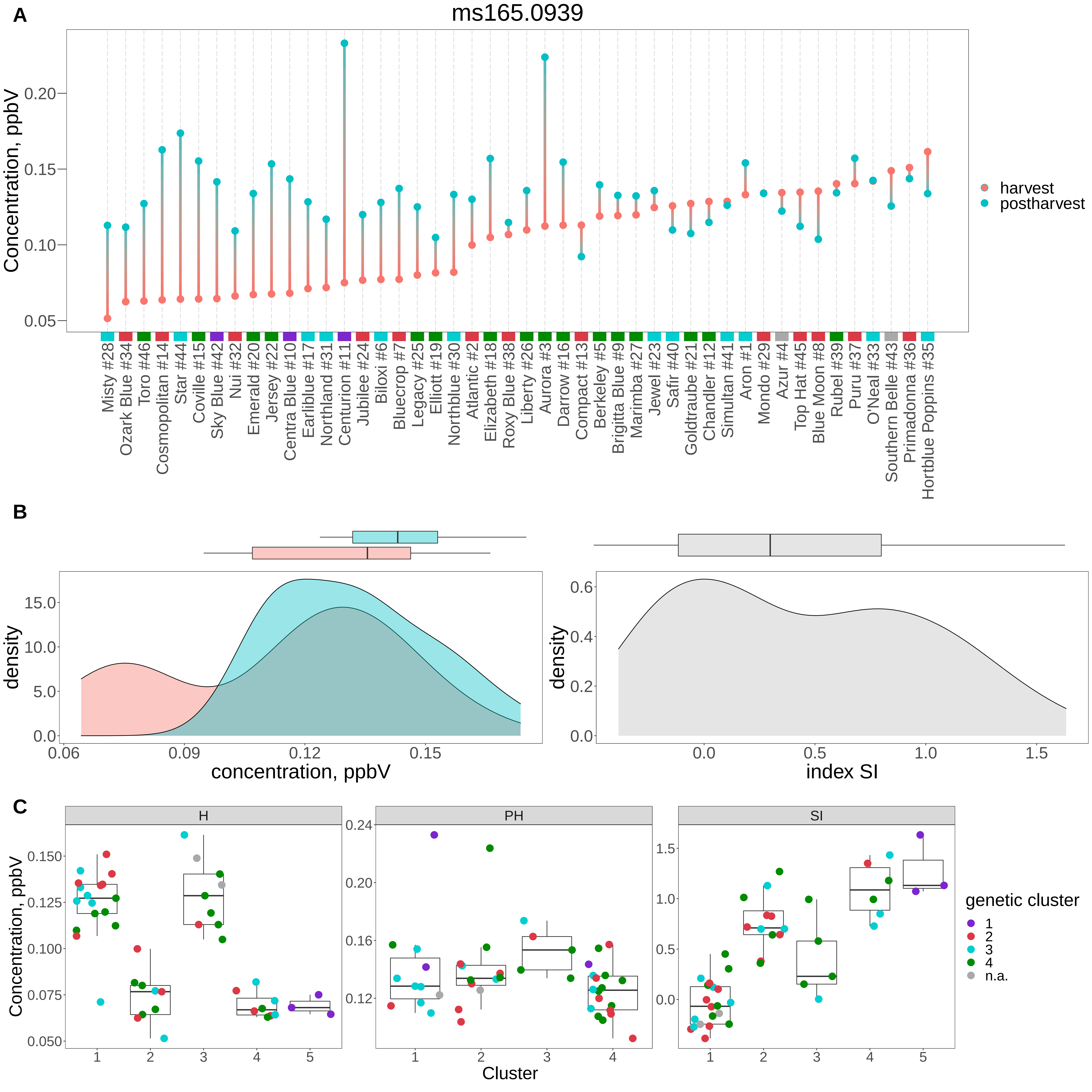

A
ms165.0939
Concentration, ppbV
harvest
postharvest
Misty #28
Ozark Blue #34
Toro #46
Cosmopolitan #14
Star #44
Coville #15
Sky Blue #42
Nui #32
Emerald #20
Jersey #22
Centra Blue #10
Earliblue #17
Northland #31
Centurion #11
Jubilee #24
Biloxi #6
Bluecrop #7
Legacy #25
Elliott #19
Northblue #30
Atlantic #2
Elizabeth #18
Roxy Blue #38
Liberty #26
Aurora #3
Darrow #16
Compact #13
Berkeley #5
Brigitta Blue #9
Marimba #27
Jewel #23
Safir #40
Goldtraube #21
Chandler #12
Simultan #41
Aron #1
Mondo #29
Azur #4
Top Hat #45
Blue Moon #8
Rubel #39
Puru #37
O'Neal #33
Southern Belle #43
Primadonna #36
Hortblue Poppins #35
B
density
concentration, ppbV
index SI
C
H
PH
SI
Concentration, ppbV
Cluster
genetic cluster
1
2
3
4
n.a.

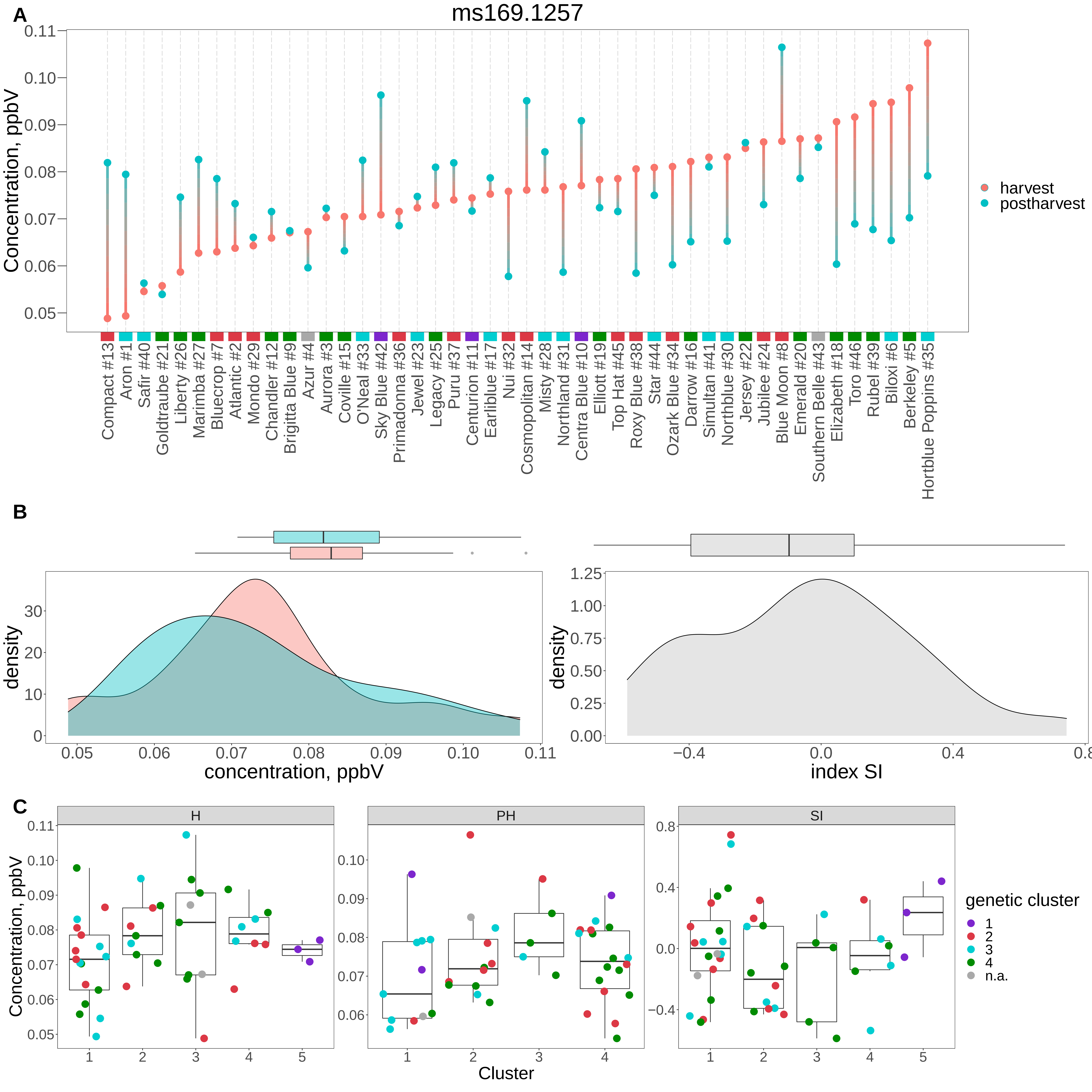

A
ms169.1257
Concentration, ppbV
harvest
postharvest
Compact #13
Aron #1
Safir #40
Goldtraube #21
Liberty #26
Marimba #27
Bluecrop #7
Atlantic #2
Mondo #29
Chandler #12
Brigitta Blue #9
Azur #4
Aurora #3
Coville #15
O'Neal #33
Sky Blue #42
Primadonna #36
Jewel #23
Legacy #25
Puru #37
Centurion #11
Earliblue #17
Nui #32
Cosmopolitan #14
Misty #28
Northland #31
Centra Blue #10
Elliott #19
Top Hat #45
Roxy Blue #38
Star #44
Ozark Blue #34
Darrow #16
Simultan #41
Northblue #30
Jersey #22
Jubilee #24
Blue Moon #8
Emerald #20
Southern Belle #43
Elizabeth #18
Toro #46
Rubel #39
Biloxi #6
Berkeley #5
Hortblue Poppins #35
B
density
concentration, ppbV
index SI
C
H
PH
SI
Cluster
genetic cluster
1
2
3
4
n.a.

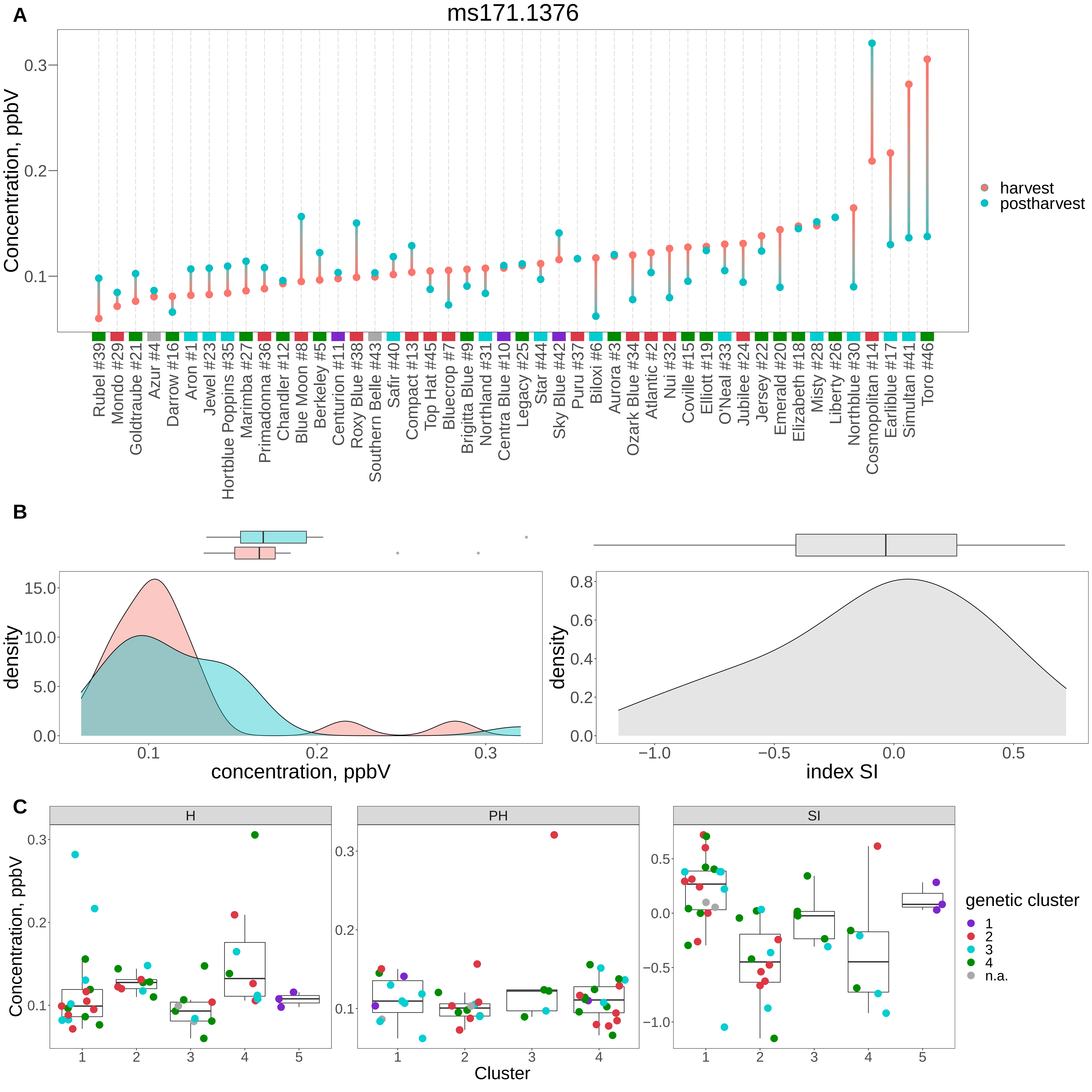

A
ms171.1376
Concentration, ppbV
harvest
postharvest
Rubel #39
Mondo #29
Goldtraube #21
Azur #4
Darrow #16
Aron #1
Jewel #23
Hortblue Poppins #35
Marimba #27
Primadonna #36
Chandler #12
Blue Moon #8
Berkeley #5
Centurion #11
Roxy Blue #38
Southern Belle #43
Safir #40
Compact #13
Top Hat #45
Bluecrop #7
Brigitta Blue #9
Northland #31
Centra Blue #10
Legacy #25
Star #44
Sky Blue #42
Puru #37
Biloxi #6
Aurora #3
Ozark Blue #34
Atlantic #2
Nui #32
Coville #15
Elliott #19
O'Neal #33
Jubilee #24
Jersey #22
Emerald #20
Elizabeth #18
Misty #28
Liberty #26
Northblue #30
Cosmopolitan #14
Earliblue #17
Simultan #41
Toro #46
B
density
concentration, ppbV
index SI
C
H
PH
SI
Cluster
genetic cluster
1
2
3
4
n.a.

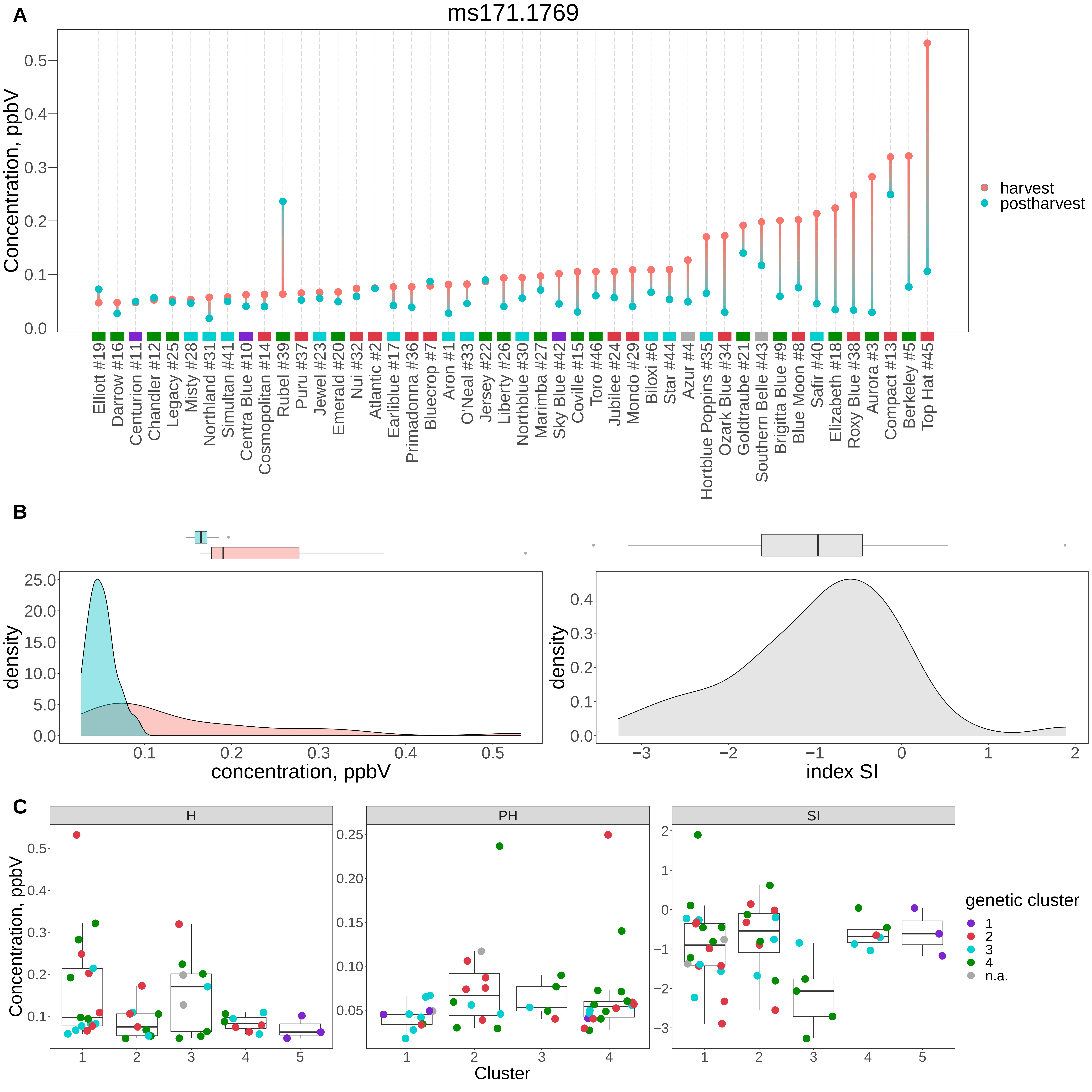
A
ms171.1769
Concentration, ppbV
harvest
postharvest
Elliott #19
Darrow #16
Centurion #11
Chandler #12
Legacy #25
Misty #28
Northland #31
Simultan #41
Centra Blue #10
Cosmopolitan #14
Rubel #39
Puru #37
Jewel #23
Emerald #20
Nui #32
Atlantic #2
Earliblue #17
Primadonna #36
Bluecrop #7
Aron #1
O'Neal #33
Jersey #22
Liberty #26
Northblue #30
Marimba #27
Sky Blue #42
Coville #15
Toro #46
Jubilee #24
Mondo #29
Biloxi #6
Star #44
Azur #4
Hortblue Poppins #35
Ozark Blue #34
Goldtraube #21
Southern Belle #43
Brigitta Blue #9
Blue Moon #8
Safir #40
Elizabeth #18
Roxy Blue #38
Aurora #3
Compact #13
Berkeley #5
Top Hat #45
B
density
concentration, ppbV
index SI
C
H
PH
SI
Concentration, ppbV
Cluster
genetic cluster
1
2
3
4
n.a.

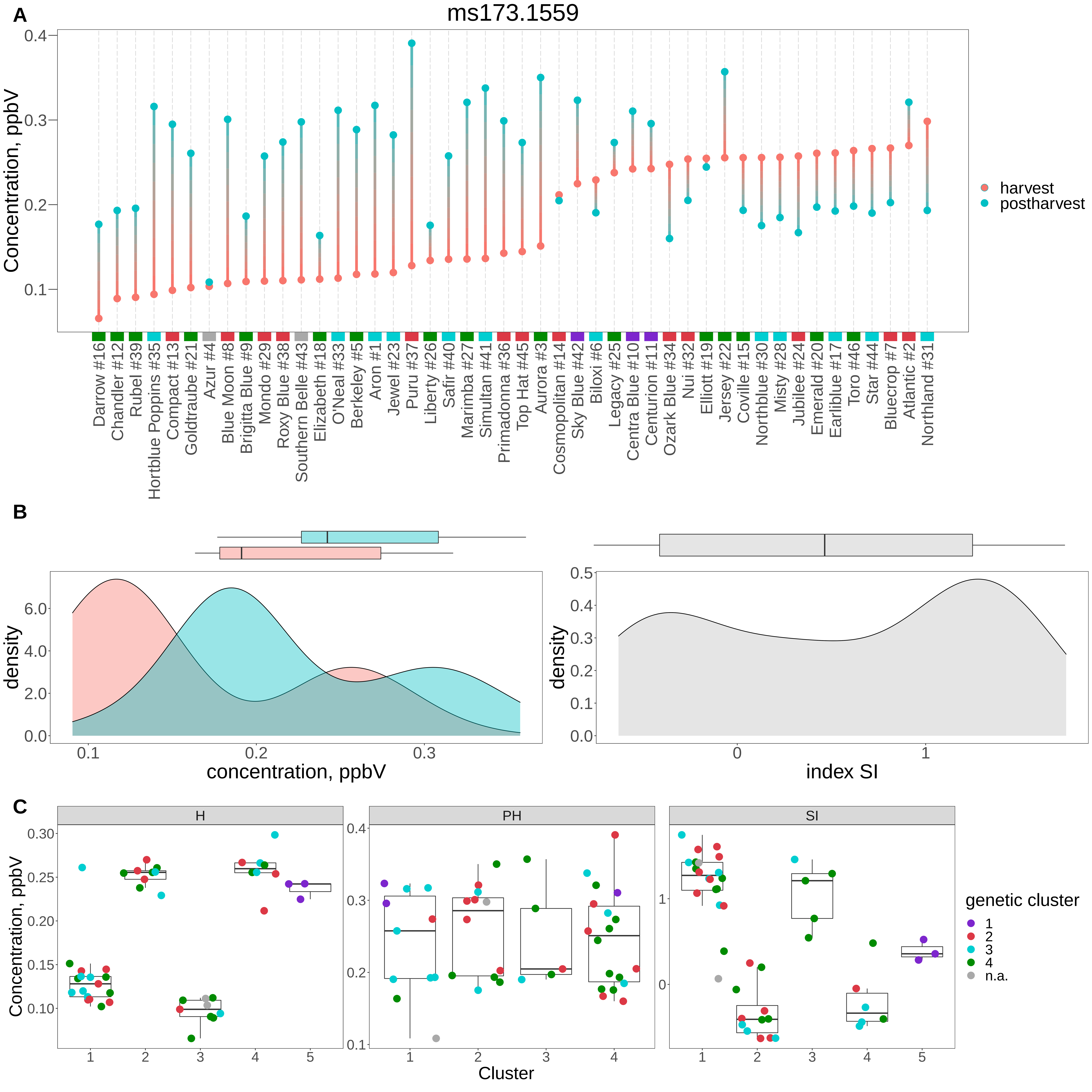
A
ms173.1559
Concentration, ppbV
harvest
postharvest
Darrow #16
Chandler #12
Rubel #39
Hortblue Poppins #35
Compact #13
Goldtraube #21
Azur #4
Blue Moon #8
Brigitta Blue #9
Mondo #29
Roxy Blue #38
Southern Belle #43
Elizabeth #18
O'Neal #33
Berkeley #5
Aron #1
Jewel #23
Puru #37
Liberty #26
Safir #40
Marimba #27
Simultan #41
Primadonna #36
Top Hat #45
Aurora #3
Cosmopolitan #14
Sky Blue #42
Biloxi #6
Legacy #25
Centra Blue #10
Centurion #11
Ozark Blue #34
Nui #32
Elliott #19
Jersey #22
Coville #15
Northblue #30
Misty #28
Jubilee #24
Emerald #20
Earliblue #17
Toro #46
Star #44
Bluecrop #7
Atlantic #2
Northland #31
B
density
concentration, ppbV
index SI
C
H
PH
SI
Concentration, ppbV
Cluster
genetic cluster
1
2
3
4
n.a.

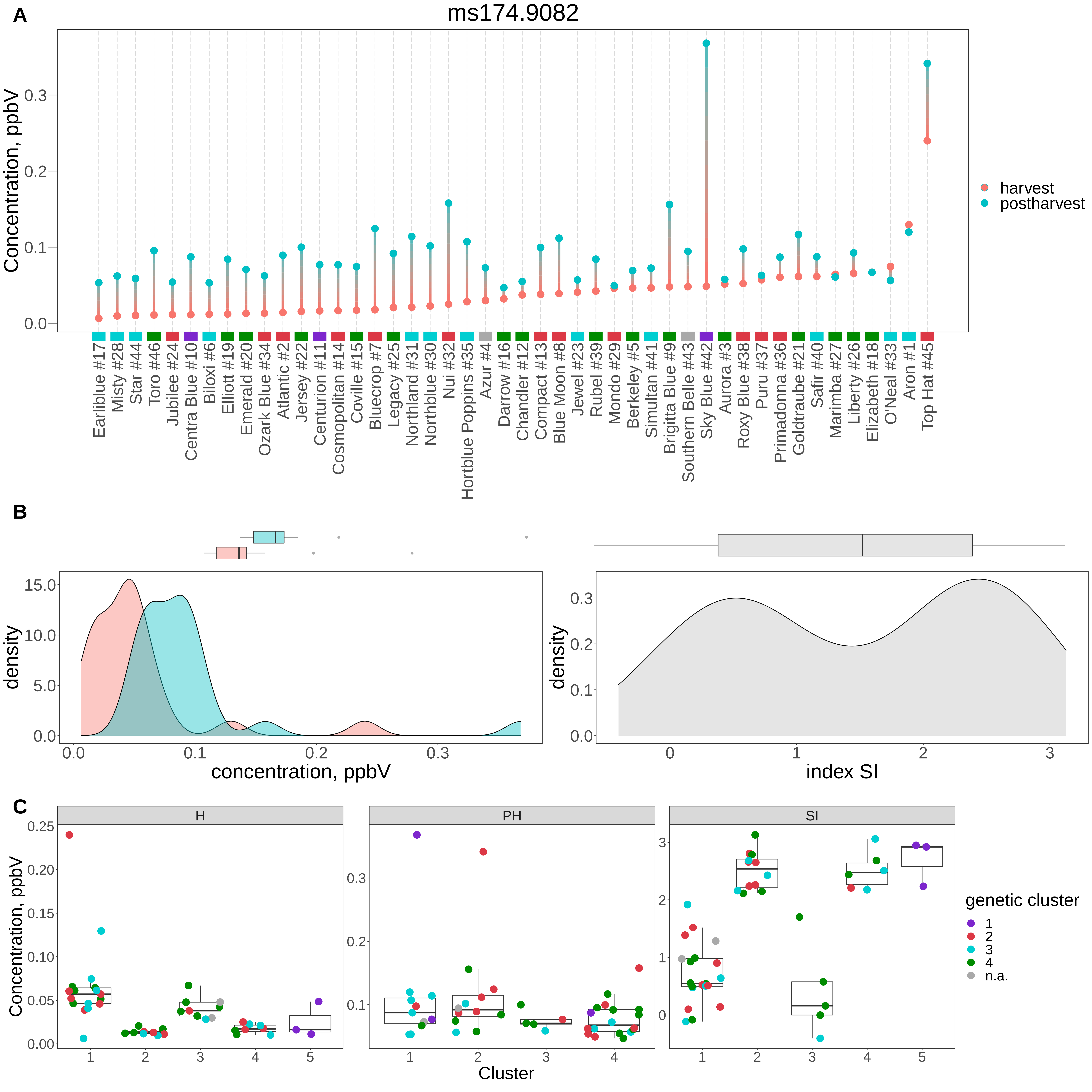

A
ms174.9082
Concentration, ppbV
harvest
postharvest
Earliblue #17
Misty #28
Star #44
Toro #46
Jubilee #24
Centra Blue #10
Biloxi #6
Elliott #19
Emerald #20
Ozark Blue #34
Atlantic #2
Jersey #22
Centurion #11
Cosmopolitan #14
Coville #15
Bluecrop #7
Legacy #25
Northland #31
Northblue #30
Nui #32
Hortblue Poppins #35
Azur #4
Darrow #16
Chandler #12
Compact #13
Blue Moon #8
Jewel #23
Rubel #39
Mondo #29
Berkeley #5
Simultan #41
Brigitta Blue #9
Southern Belle #43
Sky Blue #42
Aurora #3
Roxy Blue #38
Puru #37
Primadonna #36
Goldtraube #21
Safir #40
Marimba #27
Liberty #26
Elizabeth #18
O'Neal #33
Aron #1
Top Hat #45
B
density
concentration, ppbV
index SI
C
H
PH
SI
Concentration, ppbV
Cluster
genetic cluster
1
2
3
4
n.a.

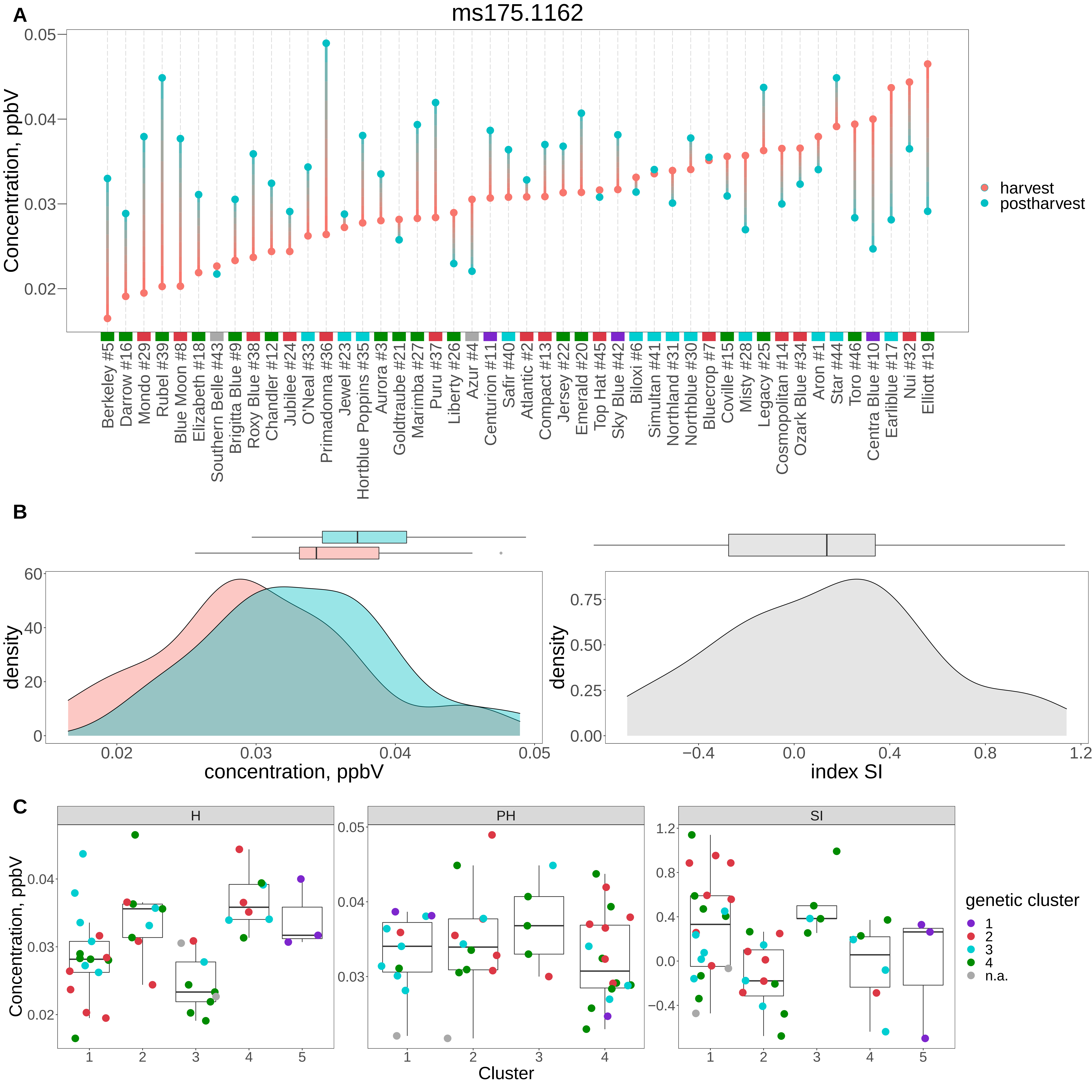

A
ms175.1162
Concentration, ppbV
0.05
0.04
0.03
0.02
harvest
postharvest
Berkeley #5
Darrow #16
Mondo #29
Rubel #39
Blue Moon #8
Elizabeth #18
Southern Belle #43
Brigitta Blue #9
Roxy Blue #38
Chandler #12
Jubilee #24
O'Neal #33
Primadonna #36
Jewel #23
Hortblue Poppins #35
Aurora #3
Goldtraube #21
Marimba #27
Puru #37
Liberty #26
Azur #4
Centurion #11
Safir #40
Atlantic #2
Compact #13
Jersey #22
Emerald #20
Top Hat #45
Sky Blue #42
Biloxi #6
Simultan #41
Northland #31
Northblue #30
Bluecrop #7
Coville #15
Misty #28
Legacy #25
Cosmopolitan #14
Ozark Blue #34
Aron #1
Star #44
Toro #46
Centra Blue #10
Earliblue #17
Nui #32
Elliott #19
B
density
60
40
20
0
0.02
0.03
0.04
0.05
concentration, ppbV
density
0.75
0.50
0.25
0.00
−0.4
0.0
0.4
0.8
1.2
index SI
C
H
PH
SI
Concentration, ppbV
Cluster
genetic cluster
1
2
3
4
n.a.

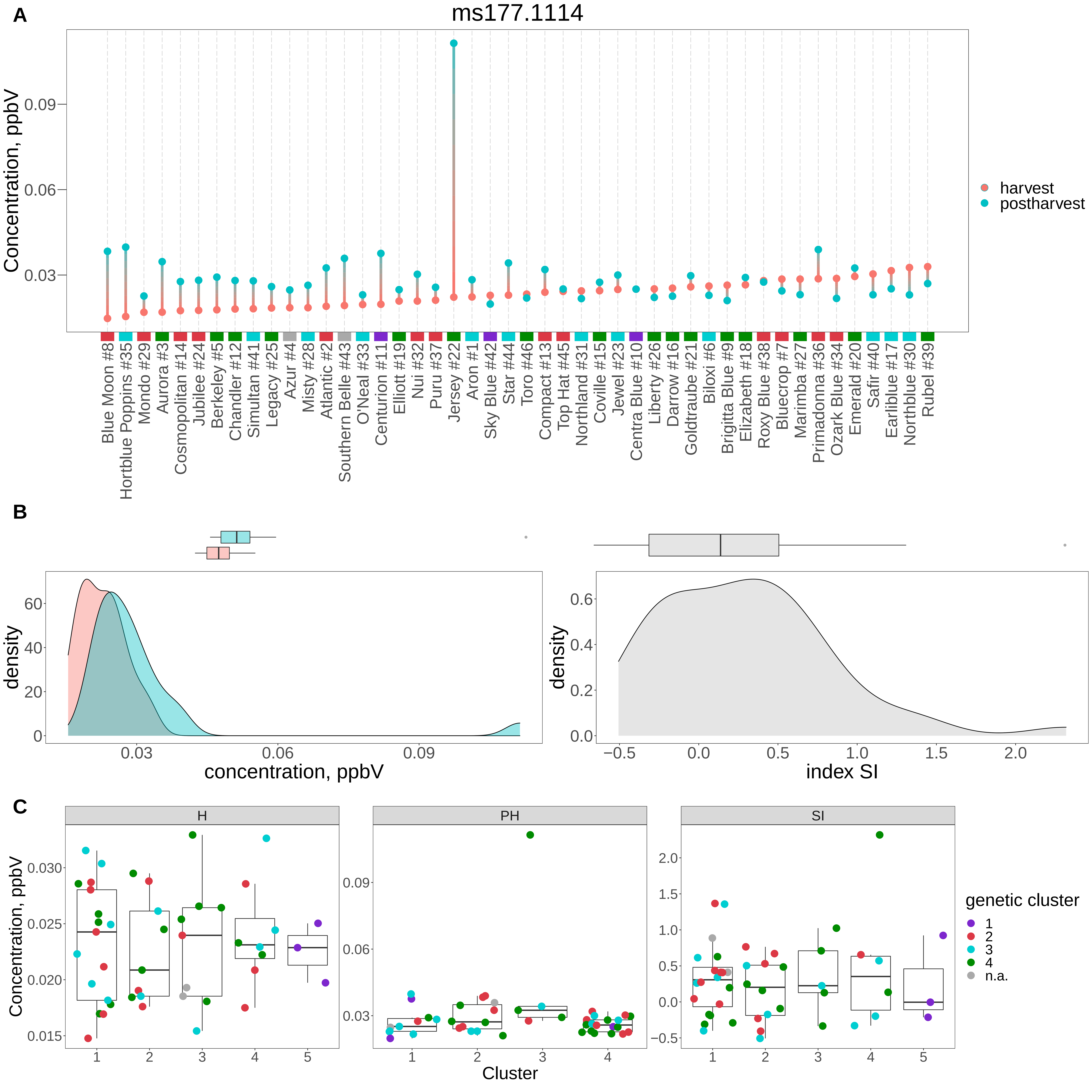
A
ms177.1114
Concentration, ppbV
0.09
0.06
0.03
harvest
postharvest
Blue Moon #8
Hortblue Poppins #35
Mondo #29
Aurora #3
Cosmopolitan #14
Jubilee #24
Berkeley #5
Chandler #12
Simultan #41
Legacy #25
Azur #4
Misty #28
Atlantic #2
Southern Belle #43
O'Neal #33
Centurion #11
Elliott #19
Nui #32
Puru #37
Jersey #22
Aron #1
Sky Blue #42
Star #44
Toro #46
Compact #13
Top Hat #45
Northland #31
Coville #15
Jewel #23
Centra Blue #10
Liberty #26
Darrow #16
Goldtraube #21
Biloxi #6
Brigitta Blue #9
Elizabeth #18
Roxy Blue #38
Bluecrop #7
Marimba #27
Primadonna #36
Ozark Blue #34
Emerald #20
Safir #40
Earliblue #17
Northblue #30
Rubel #39
B
density
concentration, ppbV
index SI
C
H
PH
SI
Concentration, ppbV
Cluster
genetic cluster
1
2
3
4
n.a.

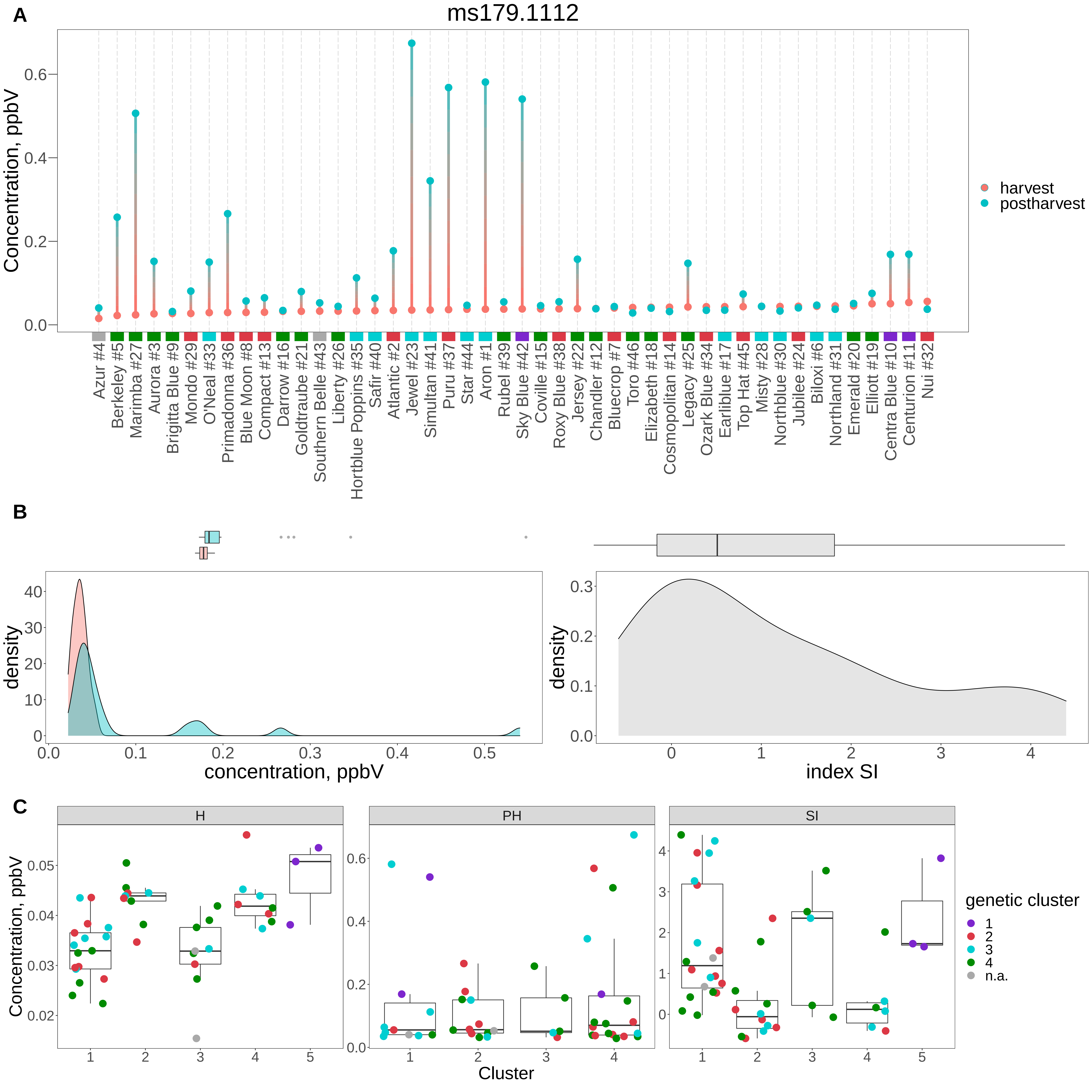

A
ms179.1112
Concentration, ppbV
harvest
postharvest
Azur #4
Berkeley #5
Marimba #27
Aurora #3
Brigitta Blue #9
Mondo #29
O'Neal #33
Primadonna #36
Blue Moon #8
Compact #13
Darrow #16
Goldtraube #21
Southern Belle #43
Liberty #26
Hortblue Poppins #35
Safir #40
Atlantic #2
Jewel #23
Simultan #41
Puru #37
Star #44
Aron #1
Rubel #39
Sky Blue #42
Coville #15
Roxy Blue #38
Jersey #22
Chandler #12
Bluecrop #7
Toro #46
Elizabeth #18
Cosmopolitan #14
Legacy #25
Ozark Blue #34
Earliblue #17
Top Hat #45
Misty #28
Northblue #30
Jubilee #24
Biloxi #6
Northland #31
Emerald #20
Elliott #19
Centra Blue #10
Centurion #11
Nui #32
B
density
concentration, ppbV
density
index SI
C
H
PH
SI
Concentration, ppbV
Cluster
genetic cluster
1
2
3
4
n.a.

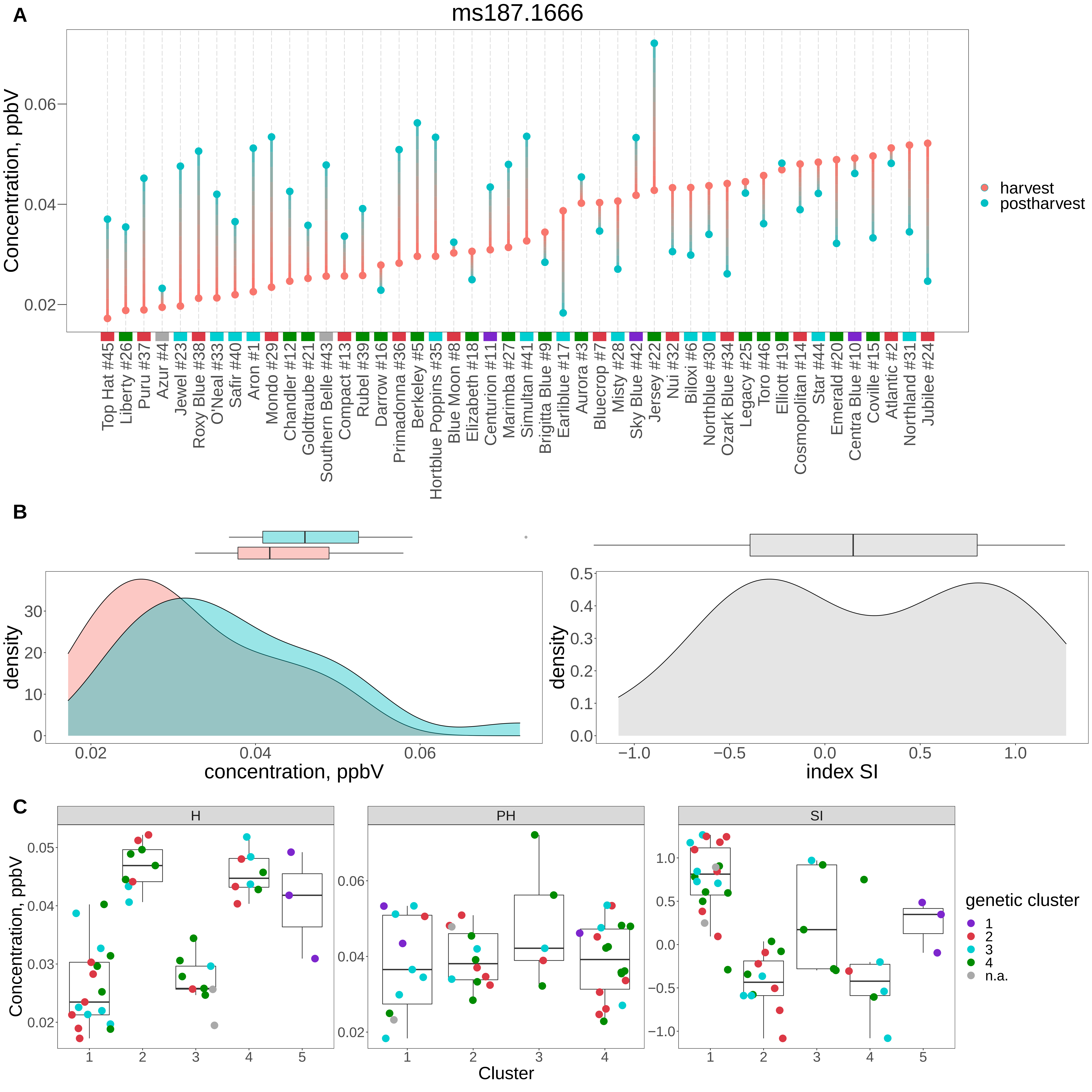

A
ms187.1666
Concentration, ppbV
harvest
postharvest
Top Hat #45
Liberty #26
Puru #37
Azur #4
Jewel #23
Roxy Blue #38
O'Neal #33
Safir #40
Aron #1
Mondo #29
Chandler #12
Goldtraube #21
Southern Belle #43
Compact #13
Rubel #39
Darrow #16
Primadonna #36
Berkeley #5
Hortblue Poppins #35
Blue Moon #8
Elizabeth #18
Centurion #11
Marimba #27
Simultan #41
Brigitta Blue #9
Earliblue #17
Aurora #3
Bluecrop #7
Misty #28
Sky Blue #42
Jersey #22
Nui #32
Biloxi #6
Northblue #30
Ozark Blue #34
Legacy #25
Toro #46
Elliott #19
Cosmopolitan #14
Star #44
Emerald #20
Centra Blue #10
Coville #15
Atlantic #2
Northland #31
Jubilee #24
B
density
concentration, ppbV
index SI
C
H
PH
SI
Concentration, ppbV
Cluster
genetic cluster
1
2
3
4
n.a.
