# Supplementary figures and images for: Development of a Novel Phenotypic Roadmap to Improve Blueberry Quality and Storability
Source: Front Plant Sci. 2020 Aug 14;11:1140. doi: 10.3389/fpls.2020.01140 (PMC7456834; doi:10.3389/fpls.2020.01140)

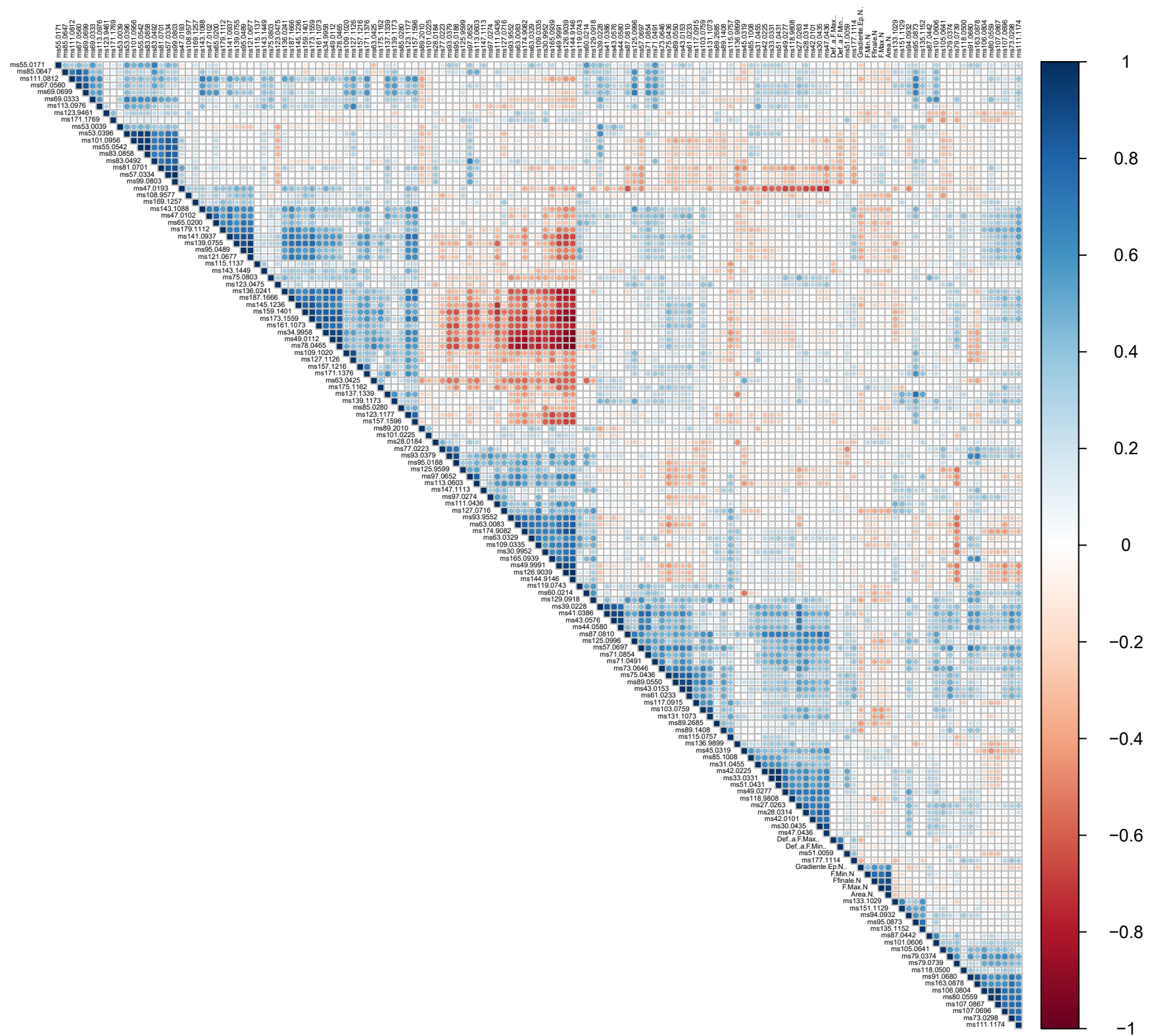

**Figure S5.** Correlation heat map of the storage index values of all quality traits

Supplement: Supplementary file 5 [file DataSheet_5.pdf]
