# Supplementary material for: Development of a Novel Phenotypic Roadmap to Improve Blueberry Quality and Storability
Source: Front Plant Sci. 2020 Aug 14;11:1140. doi: 10.3389/fpls.2020.01140 (PMC7456834; doi:10.3389/fpls.2020.01140)
Supplement: Supplementary file 6 [file DataSheet_6.pdf]

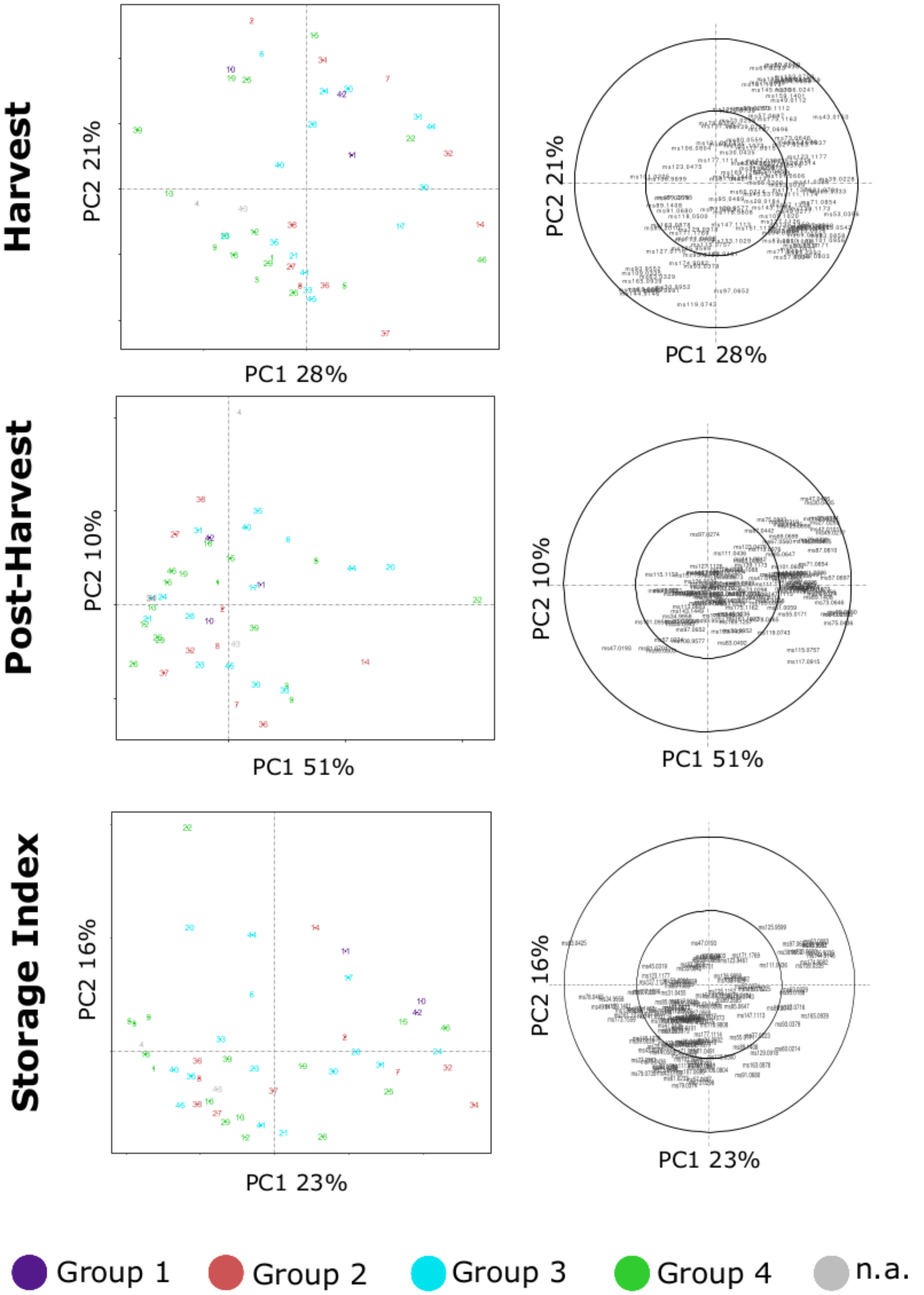

Figure S6. PCA and loading plots of the VOC profile of 46 *Vaccinium* spp. cultivars, based on VOC analysis at harvest and postharvest, and on the calculated storage index. Colours indicate the genetic clusters presented in figure 1 and table S3.
