# Supplementary material for: Development of a Novel Phenotypic Roadmap to Improve Blueberry Quality and Storability
Source: Front Plant Sci. 2020 Aug 14;11:1140. doi: 10.3389/fpls.2020.01140 (PMC7456834; doi:10.3389/fpls.2020.01140)

Figure S7. Print screens of the Shiny web application (Qualysort 1.0) with six examples of selected texture and VOC traits. This Shiny web application is interactive and gives the opportunity to choose an xlsx file with the dataset of phenotypic traits. It is possible to select sample types (in the example H – harvest, PH – postharvest and SI – storage index) and parameters of quality traits. Each parameter is classified according to the quartile it belongs to (0-25%, 25-50%, 50-75%, 75-100%) for each type separately. Button “Calculate” plots the heatmap based on the selected classified data with the hierarchical clustering (Gower's distance and clustering method – average) by samples (rows).

Example #1

QualySort 1.0

Choose xlsx file

Browse... database_H_PH_SI_selected.xlsx

Upload complete

Calculate

Select All

Choose types

- [x] H
- [ ] PH
- [ ] SI

Select All

Choose parameters

- [x] Gradiente Ep N/%
- [ ] Def. a F.Max %
- [x] F.Max N
- [ ] 33.0331 CH4OH+ Methanol
- [ ] 34.9958 H2SH+ Hydrogen sulfide
- [ ] 45.0319 C2H4OH+ Acetaldehyde
- [ ] 47.0436 C2H6OH+ Ethanol
- [ ] 49.0112 CH4SH+ Methanethiol
- [ ] 57.0697 C4H9+ 1-Octanol. high alcohol fragment
- [ ] 61.0233 C2H4O2H+ Acetic acid. common ester fragment
- [ ] 63.0329 C2H6SH+ Dimethyl sulfide. Ethanethiol
- [ ] 71.0491 C4H6OH+ Butenal
- [ ] 71.0854 C5H11+ 3-methyl-1-butanol + 2-methyl-1-butanol. Pentanol
- [ ] 73.0646 C4H8OH+ Butanale. isobutyraldehyde
- [ ] 75.0436 C3H6O2H+ Methyl acetate
- [x] 83.0858 C6H11+ (E)-3-Hexen-1-ol. (Z)-3-Hexen-1-ol. (Z)-2-Hexen-1-ol. Hexanal. 2-Hexanone
- [ ] 85.0647 C5H8OH+ (E)-2-Pentenal
- [x] 85.1008 C6H13+ Hexanol
- [ ] 87.0442 C4H6O2H+ Butyrolactone
- [ ] 87.0810 C5H10OH+ 2-methyl butanal+3-methyl butanal
- [ ] 89.0550 C4H8O2H+ Ethyl acetate
- [ ] 91.0680 C7H7+ Benzyl Alcohol
- [ ] 93.0379 C3H8OSH+ 2-(Methylthio)ethanol
- [ ] 95.0873 C7H11+ (E)-2-Heptenal; Monoterpene fragment
- [x] 99.0803 C6H10OH+ (Z)-3-Hexenal. (E)-2-Hexenal
- [ ] 101.0956 C6H12OH+ Hexanal
- [ ] 103.0759 C5H10O2H+ Ethyl Propanoate
- [ ] 107.0696 C7H6OH+ Benzaldehyde
- [ ] 107.0867 C8H10H+ Ethyl Benzene. p-Xylene. m-Xylene
- [ ] 109.1020 C8H13+ 2-Octenal (E)
- [ ] 115.0757 C6H10O2H+ Ethyl Crotonate (Ethyl (2E)-2-butenoate)
- [x] 117.0915 C6H12O2H+ Ethyl Isobutanoate. Methyl-2-methyl butanoate. Methyl Isovalerate. Ethyl Butyrate. Hexanoic Acid
- [ ] 121.0677 C8H8OH+ Acetophenone. Phenylacetaldehyde
- [ ] 127.1126 C8H14OH+ 1-octen-3-one. 6-methyl-5-hepten-2-one. (E)-2-Octenal
- [x] 131.1073 C7H14O2H+ Ethyl-2-methyl butanoate. Ethyl Isovalerate
- [ ] 133.1029 C10H15+ Thymol
- [ ] 135.1152 C10H15+ HO-Trienol
- [x] 137.1339 C10H17+ 1.8-cineole. Linalool. 4-Terpineol. alpha Terpineol. Nerol. Geraniol. Beta myrcene. Limonene. (E)-Beta Ocimene. Alpha Terpinolene
- [ ] 143.1449 C9H18OH+ 2-Nonanone. Nonanal
- [ ] 145.1236 C8H16O2H+ Ethyl Hexanoate. Hexyl Acetate. Octanoic Acid
- [ ] 159.1401 C9H18O2H+ Nonanoic Acid

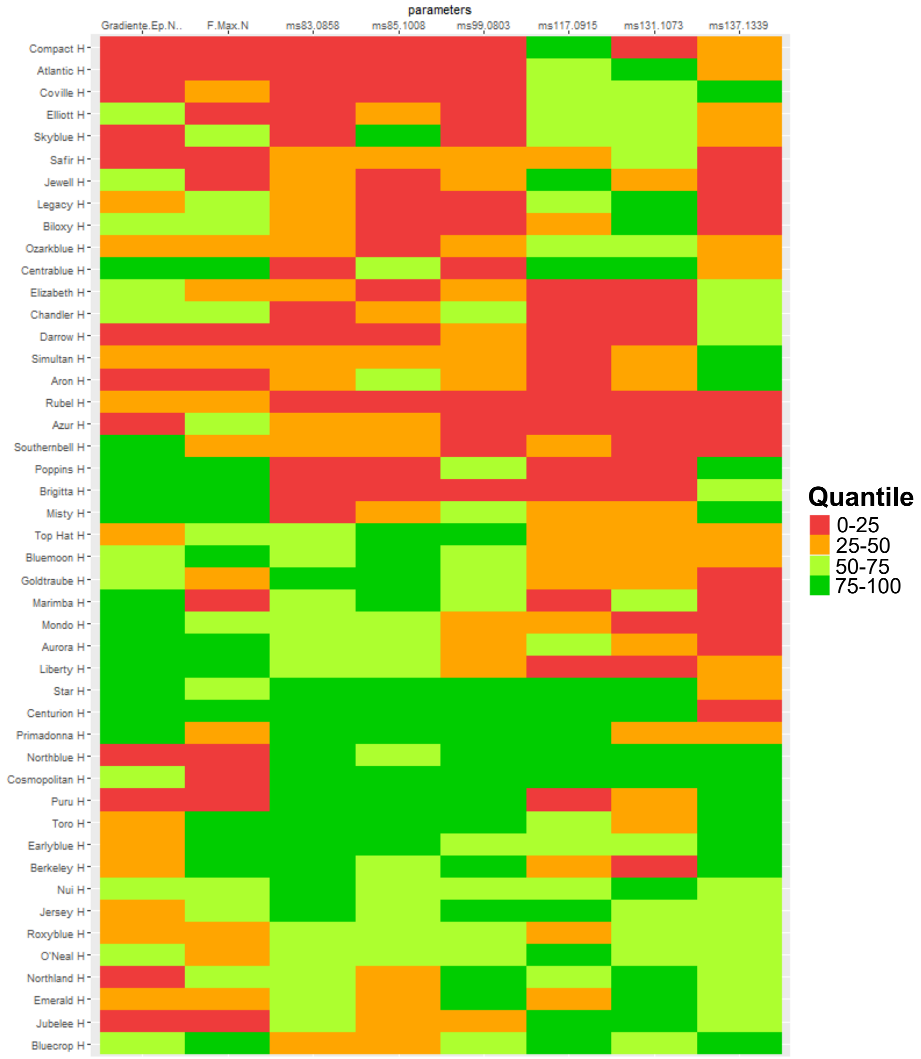

Example #2

QualySort 1.0

Choose xlsx file

Browse... database_H_PH_SI_selected.xlsx

Upload complete

Calculate

Select All

Choose types

- [ ] H
- [x] PH
- [ ] SI

Select All

Choose parameters

- [x] Gradiente Ep N/%
- [ ] Def. a F.Max %
- [x] F.Max N
- [ ] 33.0331 CH4OH+ Methanol
- [ ] 34.9958 H2SH+ Hydrogen sulfide
- [ ] 45.0319 C2H4OH+ Acetaldehyde
- [ ] 47.0436 C2H6OH+ Ethanol
- [ ] 49.0112 CH4SH+ Methanethiol
- [ ] 57.0697 C4H9+ 1-Octanol. high alcohol fragment
- [ ] 61.0233 C2H4O2H+ Acetic acid. common ester fragment
- [ ] 63.0329 C2H6SH+ Dimethyl sulfide. Ethanethiol
- [ ] 71.0491 C4H6OH+ Butenal
- [ ] 71.0854 C5H11+ 3-methyl-1-butanol + 2-methyl-1-butanol. Pentanol
- [ ] 73.0646 C4H8OH+ Butanale. isobutyraldehyde
- [ ] 75.0436 C3H6O2H+ Methyl acetate
- [x] 83.0858 C6H11+ (E)-3-Hexen-1-ol. (Z)-3-Hexen-1-ol. (Z)-2-Hexen-1-ol. Hexanal. 2-Hexanone
- [ ] 85.0647 C5H8OH+ (E)-2-Pentenal
- [x] 85.1008 C6H13+ Hexanol
- [ ] 87.0442 C4H6O2H+ Butyrolactone
- [ ] 87.0810 C5H10OH+ 2-methyl butanal+3-methyl butanal
- [ ] 89.0550 C4H8O2H+ Ethyl acetate
- [ ] 91.0680 C7H7+ Benzyl Alcohol
- [ ] 93.0379 C3H8OSH+ 2-(Methylthio)ethanol
- [ ] 95.0873 C7H11+ (E)-2-Heptenal; Monoterpene fragment
- [x] 99.0803 C6H10OH+ (Z)-3-Hexenal. (E)-2-Hexenal
- [ ] 101.0956 C6H12OH+ Hexanal
- [ ] 103.0759 C5H10O2H+ Ethyl Propanoate
- [ ] 107.0696 C7H6OH+ Benzaldehyde
- [ ] 107.0867 C8H10H+ Ethyl Benzene. p-Xylene. m-Xylene
- [ ] 109.1020 C8H13+ 2-Octenal (E)
- [ ] 115.0757 C6H10O2H+ Ethyl Crotonate (Ethyl (2E)-2-butenoate)
- [x] 117.0915 C6H12O2H+ Ethyl Isobutanoate. Methyl-2-methyl butanoate. Methyl Isovalerate. Ethyl Butyrate. Hexanoic Acid
- [ ] 121.0677 C8H8OH+ Acetophenone. Phenylacetaldehyde
- [ ] 127.1126 C8H14OH+ 1-octen-3-one. 6-methyl-5-hepten-2-one. (E)-2-Octenal
- [x] 131.1073 C7H14O2H+ Ethyl-2-methyl butanoate. Ethyl Isovalerate
- [ ] 133.1029 C10H15+ Thymol
- [ ] 135.1152 C10H15+ HO-Trienol
- [x] 137.1339 C10H17+ 1.8-cineole. Linalool. 4-Terpineol. alpha Terpineol. Nerol. Geraniol. Beta myrcene. Limonene. (E)-Beta Ocimene. Alpha Terpinolene
- [ ] 143.1449 C9H18OH+ 2-Nonanone. Nonanal
- [ ] 145.1236 C8H16O2H+ Ethyl Hexanoate. Hexyl Acetate. Octanoic Acid
- [ ] 159.1401 C9H18O2H+ Nonanoic Acid

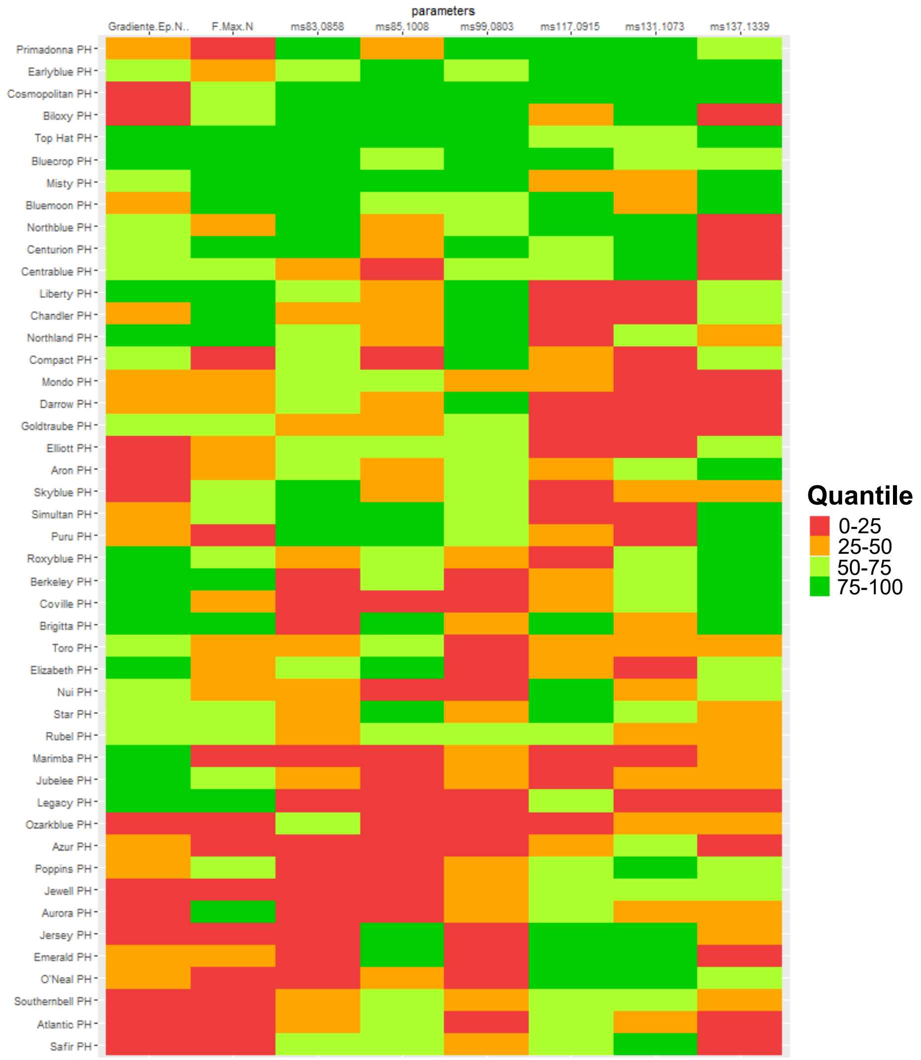

Example #3

QualySort 1.0

Choose xlsx file

Browse... database_H_PH_SI_selected.xlsx

Upload complete

Calculate

Select All

Choose types

- [ ] H
- [x] PH
- [ ] SI

Select All

Choose parameters

- [x] Gradiente Ep N/%
- [ ] Def. a F.Max %
- [x] F.Max N
- [x] 33.0331 CH4OH+ Methanol
- [ ] 34.9958 H2SH+ Hydrogen sulfide
- [x] 45.0319 C2H4OH+ Acetaldehyde
- [x] 47.0436 C2H6OH+ Ethanol
- [ ] 49.0112 CH4SH+ Methanethiol
- [ ] 57.0697 C4H9+ 1-Octanol. high alcohol fragment
- [ ] 61.0233 C2H4O2H+ Acetic acid. common ester fragment
- [ ] 63.0329 C2H6SH+ Dimethyl sulfide. Ethanethiol
- [ ] 71.0491 C4H6OH+ Butenal
- [ ] 71.0854 C5H11+ 3-methyl-1-butanol + 2-methyl-1-butanol. Pentanol
- [ ] 73.0646 C4H8OH+ Butanale. isobutyraldehyde
- [ ] 75.0436 C3H6O2H+ Methyl acetate
- [ ] 83.0858 C6H11+ (E)-3-Hexen-1-ol. (Z)-3-Hexen-1-ol. (Z)-2-Hexen-1-ol. Hexanal. 2-Hexanone
- [ ] 85.0647 C5H8OH+ (E)-2-Pentenal
- [ ] 85.1008 C6H13+ Hexanol
- [ ] 87.0442 C4H6O2H+ Butyrolactone
- [ ] 87.0810 C5H10OH+ 2-methyl butanal+3-methyl butanal
- [ ] 89.0550 C4H8O2H+ Ethyl acetate
- [ ] 91.0680 C7H7+ Benzyl Alcohol
- [ ] 93.0379 C3H8OSH+ 2-(Methylthio)ethanol
- [ ] 95.0873 C7H11+ (E)-2-Heptenal; Monoterpene fragment
- [ ] 99.0803 C6H10OH+ (Z)-3-Hexenal. (E)-2-Hexenal
- [ ] 101.0956 C6H12OH+ Hexanal
- [ ] 103.0759 C5H10O2H+ Ethyl Propanoate
- [ ] 107.0696 C7H6OH+ Benzaldehyde
- [ ] 107.0867 C8H10H+ Ethyl Benzene. p-Xylene. m-Xylene
- [ ] 109.1020 C8H13+ 2-Octenal (E)
- [ ] 115.0757 C6H10O2H+ Ethyl Crotonate (Ethyl (2E)-2-butenoate)
- [ ] 117.0915 C6H12O2H+ Ethyl Isobutanoate. Methyl-2-methyl butanoate. Methyl Isovalerate. Ethyl Butyrate. Hexanoic Acid
- [ ] 121.0677 C8H8OH+ Acetophenone. Phenylacetaldehyde
- [ ] 127.1126 C8H14OH+ 1-octen-3-one. 6-methyl-5-hepten-2-one. (E)-2-Octenal
- [ ] 131.1073 C7H14O2H+ Ethyl-2-methyl butanoate. Ethyl Isovalerate
- [ ] 133.1029 C10H15+ Thymol
- [ ] 135.1152 C10H15+ HO-Trienol
- [ ] 137.1339 C10H17+ 1.8-cineole. Linalool. 4-Terpineol. alpha Terpineol. Nerol. Geraniol. Beta myrcene. Limonene. (E)-Beta Ocimene. Alpha Terpinolene
- [ ] 143.1449 C9H18OH+ 2-Nonanone. Nonanal
- [ ] 145.1236 C8H16O2H+ Ethyl Hexanoate. Hexyl Acetate. Octanoic Acid
- [ ] 159.1401 C9H18O2H+ Nonanoic Acid

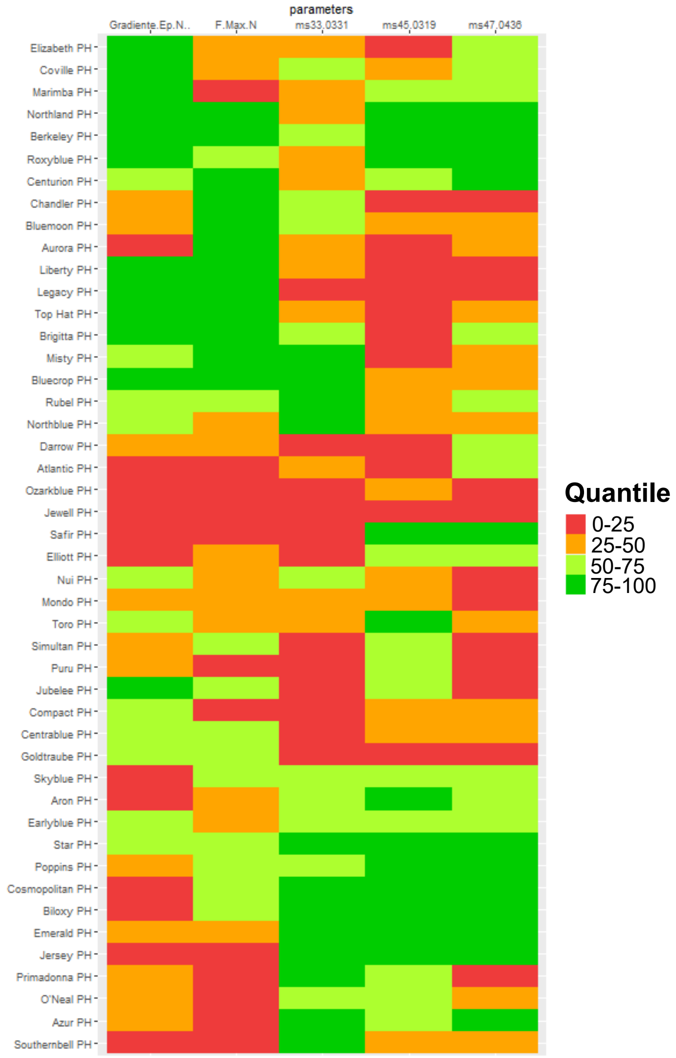

QualySort 1.0

Choose xlsx file

Browse... database_H_PH_SI_selected.xlsx

Upload complete

Calculate

Select All

Choose types

- [ ] H
- [x] PH
- [ ] SI

Select All

Choose parameters

- [ ] Gradiente Ep N/%
- [ ] Def. a F.Max %
- [ ] F.Max N
- [ ] 33.0331 CH4OH+ Methanol
- [ ] 34.9958 H2SH+ Hydrogen sulfide
- [ ] 45.0319 C2H4OH+ Acetaldehyde
- [ ] 47.0436 C2H6OH+ Ethanol
- [ ] 49.0112 CH4SH+ Methanethiol
- [ ] 57.0697 C4H9+ 1-Octanol. high alcohol fragment
- [x] 61.0233 C2H4O2H+ Acetic acid. common ester fragment
- [ ] 63.0329 C2H6SH+ Dimethyl sulfide. Ethanethiol
- [ ] 71.0491 C4H6OH+ Butenal
- [ ] 71.0854 C5H11+ 3-methyl-1-butanol + 2-methyl-1-butanol. Pentanol
- [ ] 73.0646 C4H8OH+ Butanale. isobutyraldehyde
- [x] 75.0436 C3H6O2H+ Methyl acetate
- [ ] 83.0858 C6H11+ (E)-3-Hexen-1-ol. (Z)-3-Hexen-1-ol. (Z)-2-Hexen-1-ol. Hexanal. 2-Hexanone
- [ ] 85.0647 C5H8OH+ (E)-2-Pentenal
- [ ] 85.1008 C6H13+ Hexanol
- [ ] 87.0442 C4H6O2H+ Butyrolactone
- [ ] 87.0810 C5H10OH+ 2-methyl butanal+3-methyl butanal
- [x] 89.0550 C4H8O2H+ Ethyl acetate
- [ ] 91.0680 C7H7+ Benzyl Alcohol
- [ ] 93.0379 C3H8OSH+ 2-(Methylthio)ethanol
- [ ] 95.0873 C7H11+ (E)-2-Heptenal; Monoterpene fragment
- [ ] 99.0803 C6H10OH+ (Z)-3-Hexenal. (E)-2-Hexenal
- [ ] 101.0956 C6H12OH+ Hexanal
- [x] 103.0759 C5H10O2H+ Ethyl Propanoate
- [ ] 107.0696 C7H6OH+ Benzaldehyde
- [ ] 107.0867 C8H10H+ Ethyl Benzene. p-Xylene. m-Xylene
- [ ] 109.1020 C8H13+ 2-Octenal (E)
- [x] 115.0757 C6H10O2H+ Ethyl Crotonate (Ethyl (2E)-2-butenoate)
- [x] 117.0915 C6H12O2H+ Ethyl Isobutanoate. Methyl-2-methyl butanoate. Methyl Isovalerate. Ethyl Butyrate. Hexanoic Acid
- [ ] 121.0677 C8H8OH+ Acetophenone. Phenylacetaldehyde
- [ ] 127.1126 C8H14OH+ 1-octen-3-one. 6-methyl-5-hepten-2-one. (E)-2-Octenal
- [x] 131.1073 C7H14O2H+ Ethyl-2-methyl butanoate. Ethyl Isovalerate
- [ ] 133.1029 C10H15+ Thymol
- [ ] 135.1152 C10H15+ HO-Trienol
- [ ] 137.1339 C10H17+ 1.8-cineole. Linalool. 4-Terpineol. alpha Terpineol. Nerol. Geraniol. Beta myrcene. Limonene. (E)-Beta Ocimene. Alpha Terpinolene
- [ ] 143.1449 C9H18OH+ 2-Nonanone. Nonanal
- [x] 145.1236 C8H16O2H+ Ethyl Hexanoate. Hexyl Acetate. Octanoic Acid
- [x] 159.1401 C9H18O2H+ Nonanoic Acid

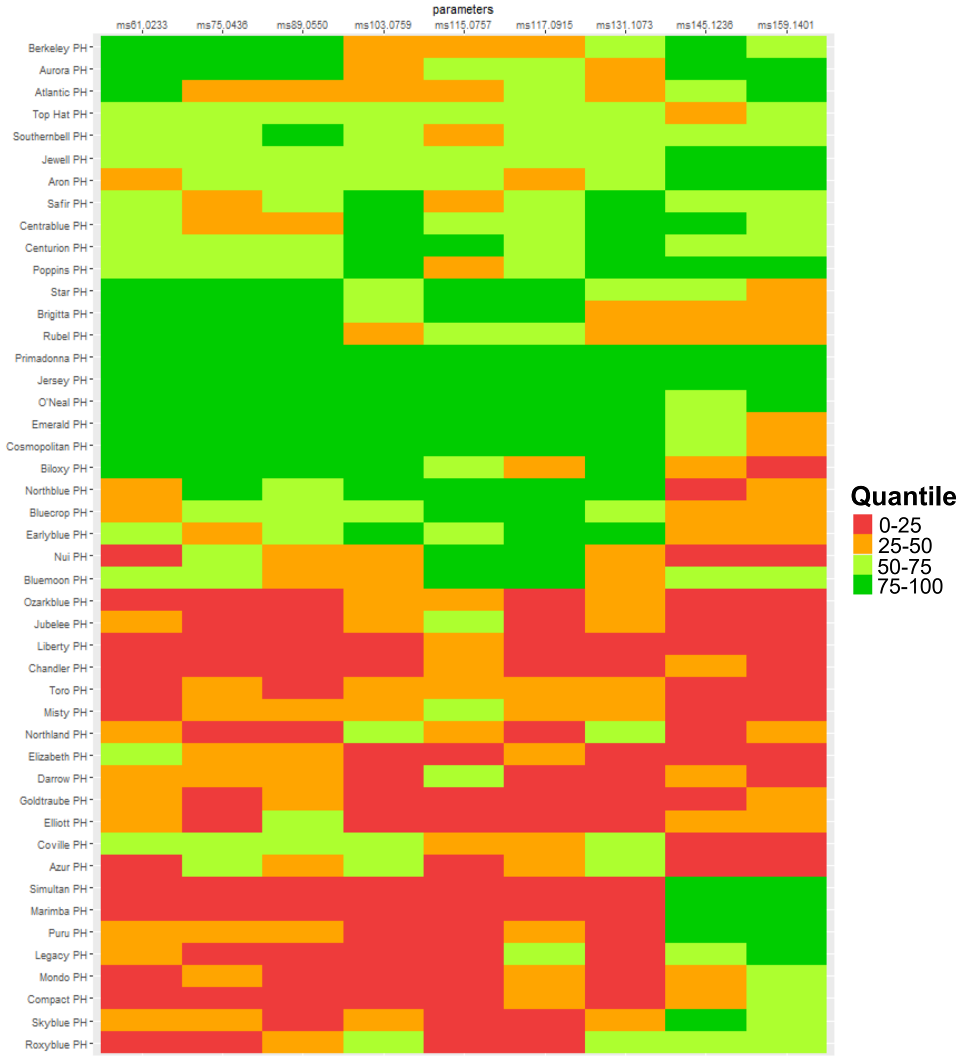

Example #5

QualySort 1.0

Choose xlsx file

Browse... | database_H_PH_SI_selected.xlsx

Upload complete

Calculate

Select All

Choose types

- [x] H
- [ ] PH
- [ ] SI

Select All

Choose parameters

- [ ] Gradiente Ep N/%
- [ ] Def. a F.Max %
- [ ] F.Max N
- [ ] 33.0331 CH4OH+ Methanol
- [x] 34.9958 H2SH+ Hydrogen sulfide
- [ ] 45.0319 C2H4OH+ Acetaldehyde
- [ ] 47.0436 C2H6OH+ Ethanol
- [x] 49.0112 CH4SH+ Methanethiol
- [ ] 57.0697 C4H9+ 1-Octanol. high alcohol fragment
- [ ] 61.0233 C2H4O2H+ Acetic acid. common ester fragment
- [x] 63.0329 C2H6SH+ Dimethyl sulfide. Ethanethiol
- [ ] 71.0491 C4H6OH+ Butenal
- [ ] 71.0854 C5H11+ 3-methyl-1-butanol + 2-methyl-1-butanol. Pentanol
- [ ] 73.0646 C4H8OH+ Butanale. isobutyraldehyde
- [ ] 75.0436 C3H6O2H+ Methyl acetate
- [ ] 83.0858 C6H11+ (E)-3-Hexen-1-ol. (Z)-3-Hexen-1-ol. (Z)-2-Hexen-1-ol. Hexanal. 2-Hexanone
- [ ] 85.0647 C5H8OH+ (E)-2-Pentenal
- [ ] 85.1008 C6H13+ Hexanol
- [ ] 87.0442 C4H6O2H+ Butyrolactone
- [ ] 87.0810 C5H10OH+ 2-methyl butanal+3-methyl butanal
- [ ] 89.0550 C4H8O2H+ Ethyl acetate
- [ ] 91.0680 C7H7+ Benzyl Alcohol
- [x] 93.0379 C3H8OSH+ 2-(Methylthio)ethanol
- [ ] 95.0873 C7H11+ (E)-2-Heptenal; Monoterpene fragment
- [ ] 99.0803 C6H10OH+ (Z)-3-Hexenal. (E)-2-Hexenal
- [ ] 101.0956 C6H12OH+ Hexanal
- [ ] 103.0759 C5H10O2H+ Ethyl Propanoate
- [ ] 107.0696 C7H6OH+ Benzaldehyde
- [ ] 107.0867 C8H10H+ Ethyl Benzene. p-Xylene. m-Xylene
- [ ] 109.1020 C8H13+ 2-Octenal (E)
- [ ] 115.0757 C6H10O2H+ Ethyl Crotonate (Ethyl (2E)-2-butenoate)
- [ ] 117.0915 C6H12O2H+ Ethyl Isobutanoate. Methyl-2-methyl butanoate. Methyl Isovalerate. Ethyl Butyrate. Hexanoic Acid
- [ ] 121.0677 C8H8OH+ Acetophenone. Phenylacetaldehyde
- [ ] 127.1126 C8H14OH+ 1-octen-3-one. 6-methyl-5-hepten-2-one. (E)-2-Octenal
- [ ] 131.1073 C7H14O2H+ Ethyl-2-methyl butanoate. Ethyl Isovalerate
- [ ] 133.1029 C10H15+ Thymol
- [ ] 135.1152 C10H15+ HO-Trienol
- [ ] 137.1339 C10H17+ 1.8-cineole. Linalool. 4-Terpineol. alpha Terpineol. Nerol. Geraniol. Beta myrcene. Limonene. (E)-Beta Ocimene. Alpha Terpinolene
- [ ] 143.1449 C9H18OH+ 2-Nonanone. Nonanal
- [ ] 145.1236 C8H16O2H+ Ethyl Hexanoate. Hexyl Acetate. Octanoic Acid
- [ ] 159.1401 C9H18O2H+ Nonanoic Acid

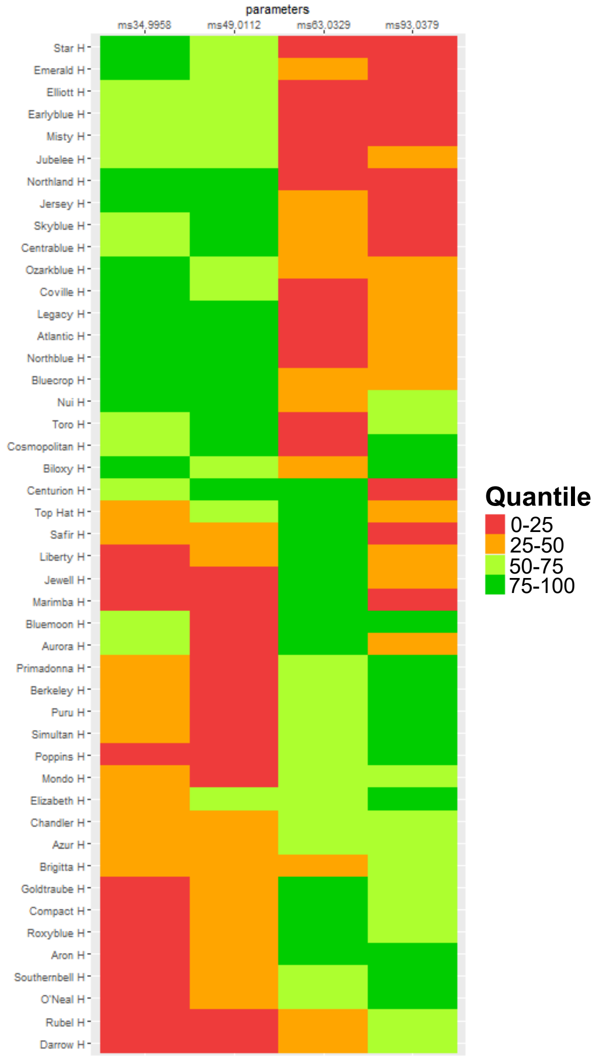

Example #6

QualySort 1.0

Choose xlsx file

Browse... database_H_PH_SI_selected.xlsx

Upload complete

Calculate

Select All

Choose types

- [ ] H
- [x] PH
- [ ] SI

Select All

Choose parameters

- [ ] Gradiente Ep N/%
- [ ] Def. a F.Max %
- [ ] F.Max N
- [ ] 33.0331 CH4OH+ Methanol
- [x] 34.9958 H2SH+ Hydrogen sulfide
- [ ] 45.0319 C2H4OH+ Acetaldehyde
- [ ] 47.0436 C2H6OH+ Ethanol
- [x] 49.0112 CH4SH+ Methanethiol
- [ ] 57.0697 C4H9+ 1-Octanol. high alcohol fragment
- [ ] 61.0233 C2H4O2H+ Acetic acid. common ester fragment
- [x] 63.0329 C2H6SH+ Dimethyl sulfide. Ethanethiol
- [ ] 71.0491 C4H6OH+ Butenal
- [ ] 71.0854 C5H11+ 3-methyl-1-butanol + 2-methyl-1-butanol. Pentanol
- [ ] 73.0646 C4H8OH+ Butanale. isobutyraldehyde
- [ ] 75.0436 C3H6O2H+ Methyl acetate
- [ ] 83.0858 C6H11+ (E)-3-Hexen-1-ol. (Z)-3-Hexen-1-ol. (Z)-2-Hexen-1-ol. Hexanal. 2-Hexanone
- [ ] 85.0647 C5H8OH+ (E)-2-Pentenal
- [ ] 85.1008 C6H13+ Hexanol
- [ ] 87.0442 C4H6O2H+ Butyrolactone
- [ ] 87.0810 C5H10OH+ 2-methyl butanal+3-methyl butanal
- [ ] 89.0550 C4H8O2H+ Ethyl acetate
- [ ] 91.0680 C7H7+ Benzyl Alcohol
- [x] 93.0379 C3H8OSH+ 2-(Methylthio)ethanol
- [ ] 95.0873 C7H11+ (E)-2-Heptenal; Monoterpene fragment
- [ ] 99.0803 C6H10OH+ (Z)-3-Hexenal. (E)-2-Hexenal
- [ ] 101.0956 C6H12OH+ Hexanal
- [ ] 103.0759 C5H10O2H+ Ethyl Propanoate
- [ ] 107.0696 C7H6OH+ Benzaldehyde
- [ ] 107.0867 C8H10H+ Ethyl Benzene. p-Xylene. m-Xylene
- [ ] 109.1020 C8H13+ 2-Octenal (E)
- [ ] 115.0757 C6H10O2H+ Ethyl Crotonate (Ethyl (2E)-2-butenoate)
- [ ] 117.0915 C6H12O2H+ Ethyl Isobutanoate. Methyl-2-methyl butanoate. Methyl Isovalerate. Ethyl Butyrate. Hexanoic Acid
- [ ] 121.0677 C8H8OH+ Acetophenone. Phenylacetaldehyde
- [ ] 127.1126 C8H14OH+ 1-octen-3-one. 6-methyl-5-hepten-2-one. (E)-2-Octenal
- [ ] 131.1073 C7H14O2H+ Ethyl-2-methyl butanoate. Ethyl Isovalerate
- [ ] 133.1029 C10H15+ Thymol
- [ ] 135.1152 C10H15+ HO-Trienol
- [ ] 137.1339 C10H17+ 1.8-cineole. Linalool. 4-Terpineol. alpha Terpineol. Nerol. Geraniol. Beta myrcene. Limonene. (E)-Beta Ocimene. Alpha Terpinolene
- [ ] 143.1449 C9H18OH+ 2-Nonanone. Nonanal
- [ ] 145.1236 C8H16O2H+ Ethyl Hexanoate. Hexyl Acetate. Octanoic Acid
- [ ] 159.1401 C9H18O2H+ Nonanoic Acid

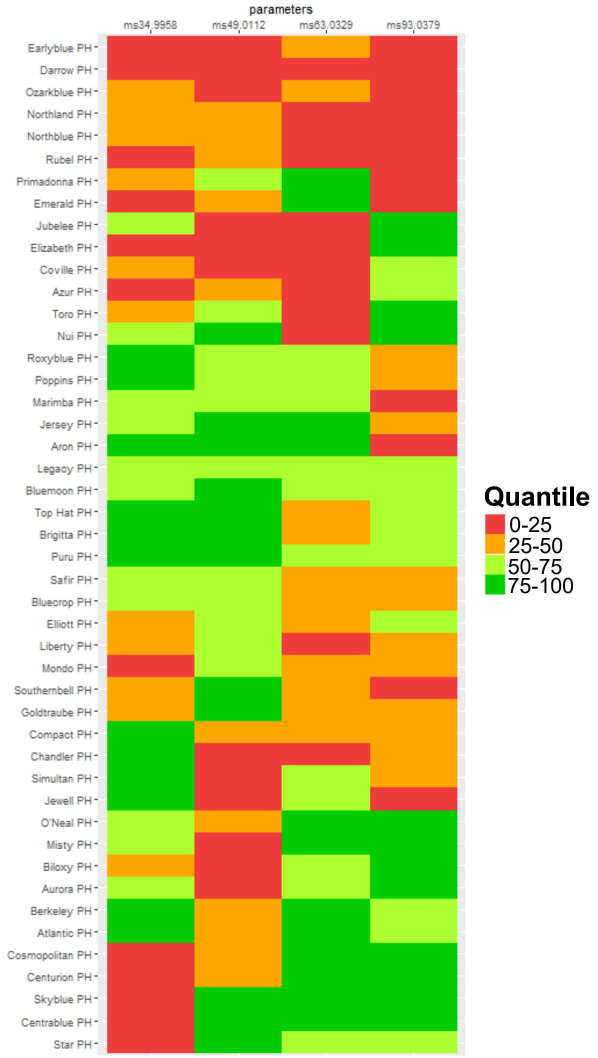

Supplement: Supplementary file 7 [file DataSheet_7.pdf]
