# Supplementary material for: Development of a Novel Phenotypic Roadmap to Improve Blueberry Quality and Storability
Source: Front Plant Sci. 2020 Aug 14;11:1140. doi: 10.3389/fpls.2020.01140 (PMC7456834; doi:10.3389/fpls.2020.01140)
Supplement: Supplementary file 8 [file DataSheet_8.pdf]

Supplementary table 1

Primer sequence of the 6 SSR Markers used for the genotyping

Multiplex	Locus	Primer name	Primer sequence 5'-3'
MVA	CA23	CA23F_m13f_fw	TGTAAAACGACGGCCAGTGAGAGGGTTTCGAGGAGGAG
		CA23F_rw	GTTTAGAAACGGGACTGTGAGACG
	CA169F	CA169F_d12_fw	ACCAACCTAGGAAACACAGTAGTGGAGGGTTTTGCTTGG
		CA169F_rw	GTTTATCGAAGCGAAGGTCAAAGA
	CA855F	CA855F_m13f_fw	TGTAAAACGACGGCCAGTCGCGTGAAAAACGACCTAAT
		CA855F_rw	GTTTACTCGATCCCTCCACCTG
MVB	CA94F	CA94F_m13f_fw	TGTAAAACGACGGCCAGTCACCCATTTCACGGAATCTC
		CA94F_rw	GTTTACTTGGTCGGGTGTTGTCTC
	NA398	NA398_m13r_fw	CAGGAAACAGCTATGACCTCCTTGCTCCAGTCCTATGC
		NA398_rw	GTTTCCTTCCACTCCAAGATGC
	CA961F	CA961F_t7_fw	TAATACGACTCACTATAGGGTCAGACATGATTGGGGAGGT
		CA961F_rw	GTTTGGAATAATAGAGGCGGTGGA
