# Supplementary material for: Development of a Novel Phenotypic Roadmap to Improve Blueberry Quality and Storability
Source: Front Plant Sci. 2020 Aug 14;11:1140. doi: 10.3389/fpls.2020.01140 (PMC7456834; doi:10.3389/fpls.2020.01140)
Supplement: Supplementary file 9 [file DataSheet_9.pdf]

Supplementary table 2

Locus-Dye-labeled universal primer combination in each multiplex reaction used for the genotyping

Multiplex	Locus	Universal Primer	Dye
MVA	CA23F	M13F	6-FAM™
	CA169F	D12S1090F	VIC®
	CA855F	M13F	6-FAM™
MVB	CA94F	M13F	6-FAM™
	NA398	M13R	NED™
	CA961F	T7	PET™
