# Supplementary material for: Development of a Novel Phenotypic Roadmap to Improve Blueberry Quality and Storability
Source: Front Plant Sci. 2020 Aug 14;11:1140. doi: 10.3389/fpls.2020.01140 (PMC7456834; doi:10.3389/fpls.2020.01140)
Supplement: Supplementary file 10 [file DataSheet_10.pdf]

Supplementary table 3

Cluster classification of blueberry accessions based on genetic and phenotypic (texture and VOCs) assessments.

		GENETIC	TEXTURE			VOCS		
**CODE**	**Name cv**	**cluster**	**cluster_H**	**cluster_PH**	**cluster_SI**	**cluster_H**	**cluster_PH**	**cluster_SI**
**1**	Aron	3	1	1	1	1	1	1
**2**	Atlantic	2	1	1	2	2	2	2
**3**	Aurora	4	2	2	1	1	2	3
**4**	Azur	n.a.	1	1	2	3	1	1
**5**	Berkeley	4	3	2	3	1	3	3
**6**	Biloxi	3	3	3	2	2	1	4
**7**	Bluecrop	2	3	2	3	4	2	2
**8**	Blue Moon	2	3	2	1	1	2	1
**9**	Brigitta Blue	4	3	2	3	3	2	3
**10**	Centra Blue	1	2	2	2	5	4	5
**11**	Centurion	1	2	2	2	5	1	5
**12**	Chandler	4	4	2	1	3	4	1
**13**	Compact	2	1	3	3	3	4	1
**14**	Cosmopolitan	2	4	2	1	4	3	4
**15**	Coville	4	1	2	3	2	2	2
**16**	Darrow	4	1	3	1	3	4	1
**17**	Earliblue	3	3	3	2	1	1	4
**18**	Elizabeth	4	4	2	3	3	1	3
**19**	Elliott	4	4	3	1	2	4	2
**20**	Emerald	4	1	3	1	2	3	4
**21**	Goldtraube	4	4	3	1	1	4	1
**22**	Jersey	4	3	1	2	4	3	4
**23**	Jewel	3	1	1	2	1	4	1
**24**	Jubilee	2	1	2	3	2	4	2
**25**	Legacy	4	4	2	3	2	4	2
**26**	Liberty	4	2	2	1	1	4	1
**27**	Marimba	4	4	3	2	1	4	1
**28**	Misty	3	3	2	1	2	4	2
**29**	Mondo	2	4	3	1	1	4	1
**30**	Northblue	3	1	3	3	4	2	2
**31**	Northland	3	3	2	3	4	1	2
**32**	Nui	2	4	3	1	4	4	2
**33**	O'Neal	3	4	3	2	1	2	3
**34**	Ozark Blue	2	1	3	2	2	4	2
**35**	Hortblue Poppins	3	3	3	2	3	1	1
**36**	Primadonna	2	4	3	2	1	2	1
**37**	Puru	2	1	3	1	1	4	1
**38**	Roxy Blue	2	1	2	3	1	1	1
**39**	Rubel	4	3	2	1	3	2	1
**40**	Safir	3	1	1	2	1	1	1
**41**	Simultan	3	1	3	1	1	4	1
**42**	Sky Blue	1	3	3	1	5	1	5
**43**	Southern Belle	n.a.	4	3	2	3	2	1
**44**	Star	3	4	2	1	4	3	4
**45**	Top Hat	2	3	2	3	1	2	1
**46**	Toro	4	3	3	2	4	4	2
